# Supplementary material for: Electrochemical C–N bond activation for deaminative reductive coupling of Katritzky salts
Source: Nat Commun. 2021 Nov 19;12:6745. doi: 10.1038/s41467-021-27060-7 (PMC8604921; doi:10.1038/s41467-021-27060-7)
Supplement: Supplementary file 1 — Supplementary Information [file 41467_2021_27060_MOESM1_ESM.pdf]

Supplementary Information

of

Electrochemical C–N Bond Activation for Deaminative Reductive Coupling of Katritzky Salts

This PDF file includes:

Supplementary Tables 1-5
Supplementary Methods 1-2
Supplementary Discussion
Supplementary Figures 1-150
Supplementary Notes

Supplementary Table 1. Optimization of cross-coupling with trifluoromethyl alkenes.

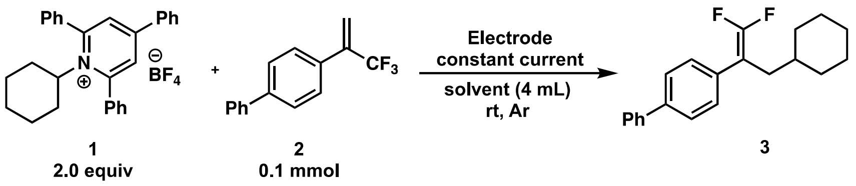

Entry	Solvent	Electrode	Current	Time	Yield[b]
1	DMSO	Zn(+)/FNE(-)	5 mA	4 h	84(79)[c]
2	dry DMSO	Zn(+)/FNE(-)	5 mA	4 h	75
3	DMA	Zn(+)/FNE(-)	5 mA	4 h	68
4	DMF	Zn(+)/FNE(-)	5 mA	4 h	62
5	NMP	Zn(+)/FNE(-)	5 mA	4 h	73
6	MeCN	Zn(+)/FNE(-)	5 mA	4 h	21
7	DMSO	Zn(+)/FNE(-)	5 mA	4 h	35 (in air)
8	DMSO	C(+)/FNE(-)	5 mA	4 h	0
9	DMSO	Fe(+)/FNE(-)	5 mA	4 h	68
10	DMSO	FNE(+)/FNE(-)	5 mA	4 h	0
11	DMSO	Zn(+)/C(-)	5 mA	4 h	78
12	DMSO	Zn(+)/Pt(-)	5 mA	4 h	65
13	DMSO	Zn(+)/FNE(-)	2.5 mA	8 h	78
14	DMSO	Zn(+)/FNE(-)	10 mA	2 h	76
15	DMSO	Zn(+)/FNE(-)	20 mA	1 h	67
16[d]	DMSO	-	-	4 h	24

[a]Reaction conditions: 1a (0.2 mmol), 2a (0.1 mmol), solvent (4 mL), under argon atmosphere for 4 h. [b]Determined by GC using 1,3,5-trimethoxybenzne as internal standard. [c]Isolated yield. [d] add 2 equiv. Zn powder, r.t..

Supplementary Table 2. Optimization of cross-coupling with disulfides.

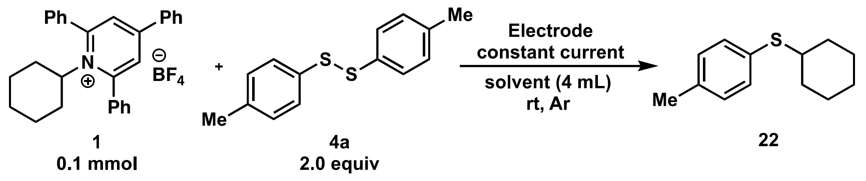

Entry	Solvent	Electrode	Current	Time	Yield[b] (%)
1	DMSO	Zn(+)/FNE(-)	5 mA	4 h	62(57)[c]
2	Acetone	Zn(+)/FNE(-)	5 mA	4 h	12
3	MeOH	Zn(+)/FNE(-)	5 mA	4 h	21
4	DMA	Zn(+)/FNE(-)	5 mA	4 h	39
5	DMF	Zn(+)/FNE(-)	5 mA	4 h	38
6	NMP	Zn(+)/FNE(-)	5 mA	4 h	36
7	MeCN	Zn(+)/FNE(-)	5 mA	4 h	23
8	Dioxane	Zn(+)/FNE(-)	5 mA	4 h	n.r.
9(in air)	DMSO	Zn(+)/FNE(-)	5 mA	4 h	12
10	DMSO	Zn(+)/C(-)	5 mA	4 h	16
11	DMSO	Zn(+)/RVC(-)	5 mA	4 h	30
12	DMSO	Zn(+)/Pt(-)	5 mA	4 h	30
13	DMSO	Fe(+)/FNE(-)	5 mA	4 h	31
14	DMSO	Mg(+)/FNE(-)	5 mA	4 h	50
15	DMSO	Al(+)/FNE(-)	5 mA	4 h	31
16	DMSO	Zn(+)/FNE(-)	2.5 mA	8 h	54
17	DMSO	Zn(+)/FNE(-)	10 mA	2 h	50
18	DMSO	Zn(+)/FNE(-)	20 mA	1 h	48
19[d]	DMSO	-	-	4 h	20

[a]Reaction conditions: 1a (0.1 mmol), 4a (0.2 mmol), solvent (4 mL), under argon atmosphere for 4 h. [b]Determined by GC using 1,3,5-trimethoxybenzne as internal standard. [c]Isolated yield. [d] add 2 equiv. Zn powder, r.t..

Supplementary Table 3. Optimization of cross-coupling with alkynyl p-tolylsulfones.

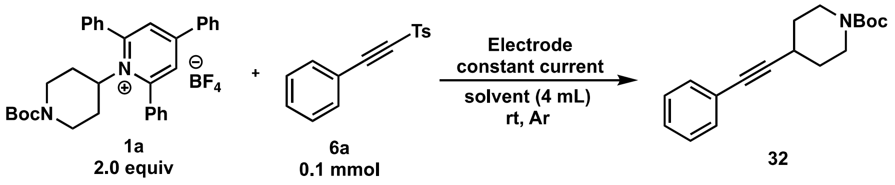

Entry	Solvent	Electrode	Current	Time	Yield[b] (%)
1	DMF	Fe(+)/FNE(-)	5 mA	4 h	57(54)[c]
2	DMA	Zn(+)/FNE(-)	5 mA	4 h	23
3	MeOH	Zn(+)/FNE(-)	5 mA	4 h	30
4	DMSO	Zn(+)/FNE(-)	5 mA	4 h	trace
5	MeCN	Zn(+)/FNE(-)	5 mA	4 h	24
6	NMP	Zn(+)/FNE(-)	5 mA	4 h	30
7	DMF	Zn (+)/FNE(-)	5 mA	4 h	32
8	DMF	Mg(+)/FNE(-)	5 mA	4 h	0
9	DMF	Al(+)/FNE(-)	5 mA	4 h	0
10	DMF	Fe(+)/C(-)	5 mA	4 h	39
11	DMF	Fe(+)/RVC(-)	5 mA	4 h	15
12	DMF	Fe(+)/Pt(-)	5 mA	4 h	47
13	DMF	Fe(+)/Ni(-)	5 mA	4 h	33
14	DMF	Fe(+)/Cu(-)	5 mA	4 h	29

[a]Reaction conditions: 1a (0.2 mmol), 6a (0.1 mmol), solvent (4 mL), under argon atmosphere for 4 h. [b]Determined by GC using 1,3,5-trimethoxybenzne as internal standard. [c]Isolated yield.

Supplementary Table 4. Control Experiments.

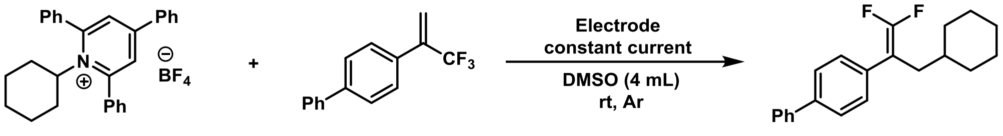

1 2.0 equiv. 2 0.1 mmol 3

Entry	Katritzky salts	$^{n}Bu_4NPF_6$	Yield[b] (%)
1	2 equiv.	0	84
2	1 equiv.	0	51
3	1 equiv.	1 equiv.	50

[a]Reaction conditions: 1a (0.2 mmol), 2a (0.1 mmol), solvent (4 mL), under argon atmosphere for 4 h. [b]Determined by GC using 1,3,5-trimethoxybenzne as internal standard. [c]Isolated yield.

Supplementary Table 5. Optimization of cross-coupling with trifluoromethyl alkenes using electrolytes.

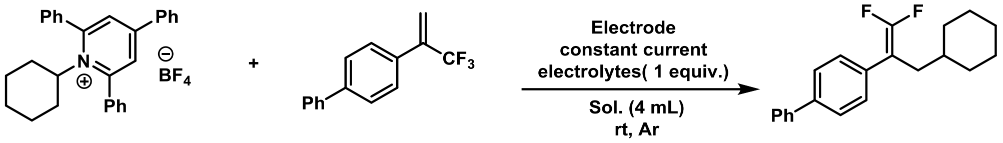

1 1.0 equiv. 2 0.1 mmol 3

Entry	Solvent	Electrolytes	Electolytes	Electricity	Yield[b]
1	DMSO	Zn(+)/FNE(-)	$^{n}Bu_4NPF_6$	5 mA /4 h	50
2	DMSO	Zn(+)/FNE(-)	$^{n}Bu_4NBF_6$	5 mA /4 h	52
3	DMSO	Zn(+)/FNE(-)	$LiClO_4$	5 mA /4 h	46
4	DMSO	Zn(+)/FNE(-)	$^{n}Bu_4NBr$	5 mA /4 h	11
5	DMSO	Zn(+)/FNE(-)	$^{n}Bu_4NOAc$	5 mA /4 h	29
6	MeCN	Zn(+)/FNE(-)	$^{n}Bu_4NPF_6$	5 mA /4 h	21
7	DMSO	Fe(+)/FNE(-)	$^{n}Bu_4NPF_6$	5 mA /4 h	17
8	DMSO	Zn (+)/FNE(-)	$^{n}Bu_4NPF_6$	2.5 mA /8 h	46
9	DMSO	Zn (+)/FNE(-)	$^{n}Bu_4NPF_6$	10 mA /2 h	44
10[c]	DMSO	Zn (+)/FNE(-)	$^{n}Bu_4NPF_6$	5 mA /4 h	53

[a]Reaction conditions: 1a (0.1 mmol), 2a (0.1 mmol), solvent (4 mL), under argon atmosphere for 4 h. [b]Determined by GC using 1,3,5-trimethoxybenzne as internal standard. [c] 50 °C

Supplementary Method 1

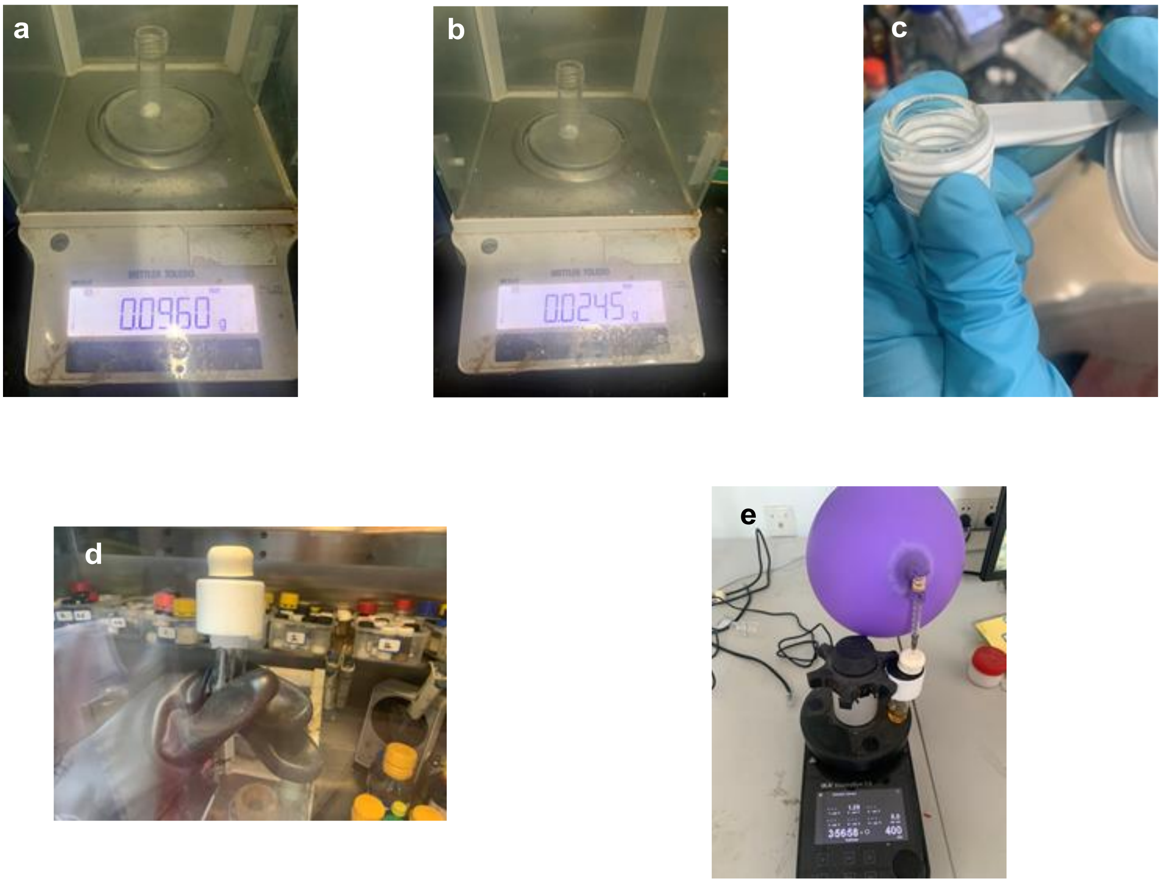

Supplementary Figure 1. a) 0.2 mmol **1a**. b) 0.1 mmol **2a**. c) The vessel was wraped with teflon tape. d) Solvent was added and the vessel was sealed in the glove box. e) A balloon filled with nitrogen was connected to the vessel.

Supplementary Method 2

The continuous-flow electrochemical reactor used was the Asia Flux Module purchased from Syrris as shown in Supplementary Figure 2 and 3.

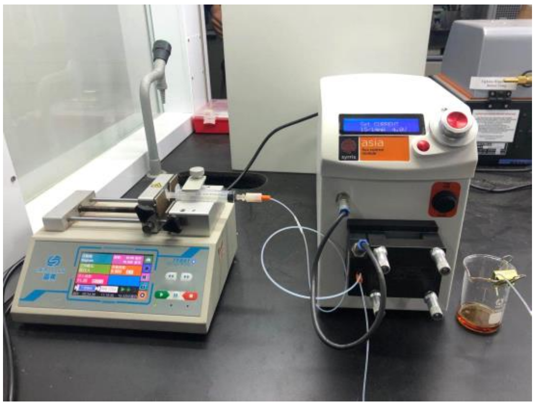

Supplementary Figure 2 The setup of the Asia Flux Module

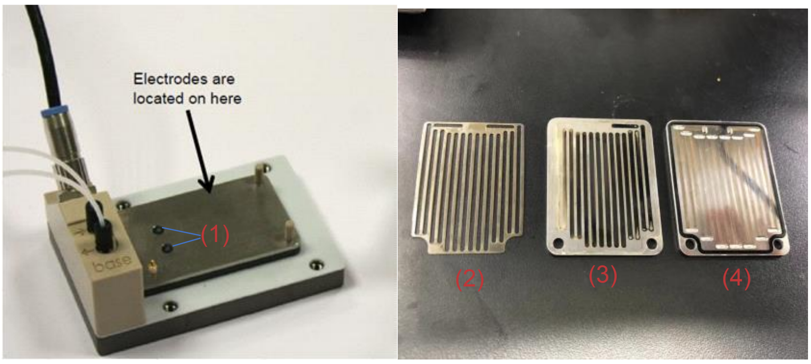

Supplementary Figure 3 The components of the reactor.
(1): inlet and outlet; (2): channel reator; (3): anode; (4): cathode.

The result of reaction in continuous-flow electrochemical reactor as shown in Supplementary Figure 4. We found that using SS304 as anode and platinum as cathode, with a current set to10 mA the flow rate set to 22.5 uL/min, our target product could be obtained in 26% yield after 10 minute under air.

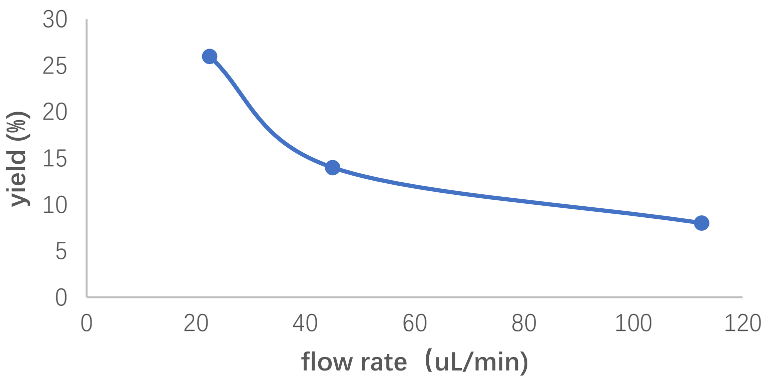

Supplementary Figure 4 The result of reaction in continuous-flow electrochemical reactor

Supplementary Disscussion

For demonstrated the possible reaction mechanism, electron paramagnetic resonance (EPR) experiments with *N*-tert-butyl-α-phenylnitrone (PBN) as the electron-spin trapping reagent were carried out. A significant EPR signal was observed when the reaction was charged with 5.0 mA current. Combining the above results indicating that the reaction probably proceeded via a radical process.

i） **PBN**, no EPR signal

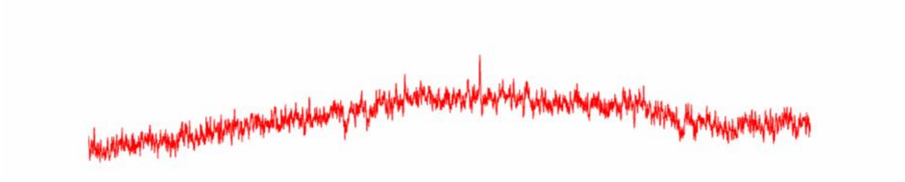

ii） **1+2+PBN** in DMSO, (+)Zn / (-)NFE, I = 5.0 mA

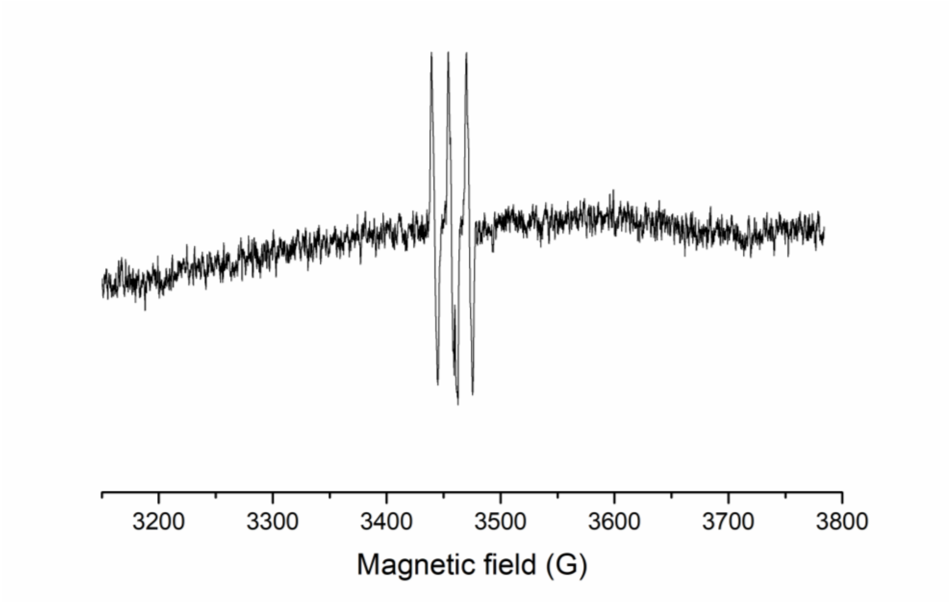

Supplementary Figure 5 EPR experiments

Characterization Data

Compound 3

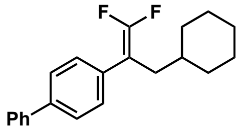

Purification by flash column chromatography (silica gel, petroleum ether) afforded 24.7mg (79%) of the title compound **3**.
Physical State: white solid.
R_f = 0.75 (petroleum ether).
^{1}H NMR (400 MHz, $CDCl_3$): δ 7.68-7.56 (m, 4H), 7.52-7.32 (m, 5H), 2.35-2.30 (m, 2H), 1.76-1.62 (m, 5H), 1.37-1.30 (m, 1H), 1.21-1.10 (m, 3H), 1.03-0.91 (m, 2H) ppm.
^{13}C NMR (126 MHz, $CDCl_3$): δ 154.2 (dd, J = 290.6, 286.3 Hz), 140.7, 140.0, 133.2 (dd, J = 4.6, 4.2 Hz), 128.9, 128.8 (t, J = 3.3 Hz), 127.5, 127.2, 127.1, 90.9 (dd, J = 22.3, 12.3 Hz), 35.9 (t, J = 2.4 Hz), 35.3, 33.0, 26.6, 26.2 ppm.
^{19}F NMR (376 MHz, $CDCl_3$): δ -90.67 (d, J = 43.5 Hz), -91.22 (d, J = 43.2 Hz) ppm.
All data matched that reported in the literature[1]

Compound 4

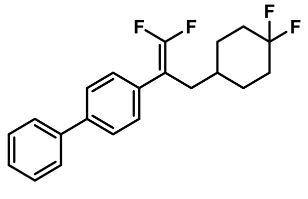

Purification by pTLC (Petroleum ether) afforded 25.4 mg (73%) of the title compound **4**
Physical State: colorless oil.
R_f = 0.50 (petroleum ether).
^{1}H NMR (500 MHz, $CDCl_3$): δ 7.64 – 7.57 (m, 4H), 7.50 – 7.42 (m, 2H), 7.41 – 7.34 (m, 3H), 2.46 – 2.32 (m, 2H), 2.12 – 2.00 (m, 2H), 1.82 – 1.73 (m, 2H), 1.71 – 1.52 (m, 2H), 1.45 – 1.39 (m, 1H), 1.38 – 1.30 (m, 2H).
^{13}C NMR (126 MHz, $CDCl_3$): δ 154.29 (dd, J = 291.6, 287.0 Hz), 140.57, 140.37, 132.58 (d, J = 7.5 Hz), 128.99, 128.67 (t, J = 3.5 Hz), 127.64, 127.38, 127.15, 90.68 (dd, J = 22.0, 13.3 Hz), 34.08, 33.73, 33.38 (dd, J = 25.1, 22.8 Hz), 29.86, 28.69 (d, J = 9.2 Hz).
^{19}F NMR (471 MHz, $CDCl_3$): δ -90.06 (d, J = 42.0 Hz), -90.52 (d, J = 41.0 Hz), -92.13 (d, J = 233.6 Hz), -101.86 (d, J = 233.7 Hz).
HRMS (ESI-TOF): calc'd for $C_{21}H_{21}F_4$ $[M+H]^+$: 349.1574, found: 349.1572.

Compound 5

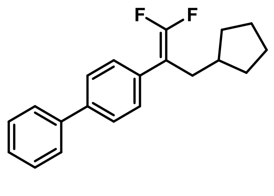

Purification by pTLC (Petroleum ether) afforded 15.2 mg (Three parallel experiments) (17%) of the title compound **5**.
Physical State: white solid.
R_f = 0.80 (petroleum ether).
^{1}H NMR (500 MHz, $CDCl_3$): δ 7.65 – 7.56 (m, 4H), 7.49 – 7.41 (m, 3H), 7.43 – 7.37 (m, 2H), 7.39 – 7.31 (m, 2H), 2.44 (dt, *J* = 7.6, 2.4 Hz, 2H), 1.93 – 1.80 (m, 1H), 1.75 – 1.65 (m, 2H), 1.66 – 1.58 (m, 2H), 1.55 – 1.44 (m, 2H), 1.25 – 1.13 (m, 2H).
^{13}C NMR (126 MHz, $CDCl_3$): δ 154.12 (dd, *J* = 290.7, 286.1 Hz), 140.77, 140.09, 133.14 (dd, *J* = 4.6, 2.9 Hz), 128.94, 128.86 (t, *J* = 3.2 Hz), 127.50, 127.19, 127.16, 92.20 (dd, *J* = 22.0, 12.1 Hz), 38.44 (t, *J* = 2.6 Hz), 33.63, 32.31, 25.13.
^{19}F NMR (471 MHz, $CDCl_3$): δ -91.54 (d, *J* = 44.8 Hz), -91.94 (d, *J* = 44.8 Hz).
All data matched that reported in the literature [10]

Compound 6

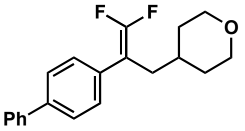

Purification by pTLC (30:1 petroleum ether : EtOAc) afforded 22.0 mg (70%) of the title compound **6**.
Physical State: yellow solid.
R_f = 0.25 (30:1 petroleum ether : EtOAc).
^{1}H NMR (400 MHz, $CDCl_3$): δ 7.67-7.54 (m, 4H), 7.51-7.32 (m, 5H), 3.97-3.89 (m, 2H), 3.32-3.25 (m, 2H), 2.45-2.34 (m, 2H), 1.64-1.50 (m, 3H), 1.38-1.26 (m, 2H) ppm.
^{13}C NMR (101 MHz, $CDCl_3$): δ 154.3 (dd, *J* = 291.3, 286.9 Hz), 140.6, 140.3, 133.2 (dd, *J* = 4.6, 4.2 Hz), 129.0, 128.7 (t, *J* = 3.4 Hz), 127.6, 127.3, 127.1, 90.9 (dd, *J* = 22.3, 12.3 Hz), 67.9, 34.8, 33.4 (t, *J* = 2.7 Hz), 32.8 ppm.
^{19}F NMR (376 MHz, $CDCl_3$): δ -90.14 (d, *J* = 42.2 Hz), -90.56 (d, *J* = 41.4 Hz) ppm.
HRMS (ESI-TOF): calc'd for $C_{20}H_{20}F_2O$ $[M+H]^+$: 315.1555, found: 315.1554.

Compound 7

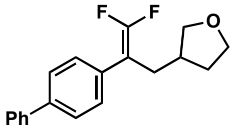

Purification by pTLC (30:1 petroleum ether : EtOAc) afforded 18.6 mg (62%) of the title compound **7**.
Physical State: yellow oil.
R_f = 0.25 (30:1 petroleum ether : EtOAc).
^{1}H NMR (400 MHz, $CDCl_3$): δ 7.65-7.56 (m, 4H), 7.50-7.41 (m, 2H), 7.43-7.31 (m, 3H), 3.89 (td, *J* = 8.2, 5.1 Hz, 1H), 3.80 (dd, *J* = 8.5, 7.0 Hz, 1H), 3.72 (dt, *J* = 8.5, 7.5 Hz, 1H), 3.43 (dd, *J* = 8.5, 6.4 Hz, 1H), 2.59-2.49 (m, 2H), 2.33-2.22 (m, 1H), 2.01-1.93 (m, 1H), 1.64-1.55 (m, 1H) ppm.
^{13}C NMR (126 MHz, $CDCl_3$): δ 154.2 (dd, *J* = 290.7, 287.4 Hz), 140.6, 140.5, 132.3 (dd, *J* = 3.6, 3.4 Hz), 129.0, 128.8 (t, *J* = 3.2 Hz), 127.6, 127.4, 127.1, 91.4 (dd, *J* = 21.3, 14.1 Hz), 72.8, 67.9,

37.8 (t, J = 2.5 Hz), 31.9, 31.2 ppm.
^{19}F NMR (376 MHz, CDCl$_3$): δ -90.63 (d, J = 42.2 Hz), -90.87 (d, J = 42.5 Hz) ppm.
HRMS (ESI-TOF): calc'd for $C_{19}H_{18}F_2O$ [M+H]$^+$: 301.1398, found: 301.1398.

Compound 8

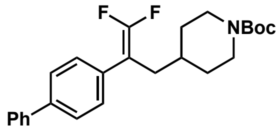

Purification by pTLC (20:1 petroleum ether : EtOAc) afforded 33.1 mg (80%) of the title compound **8**.
Physical State: colorless oil.
R$_f$ = 0.40 (20:1 petroleum ether : EtOAc).
^{1}H NMR (400 MHz, CDCl$_3$): δ 7.66-7.57 (m, 4H), 7.53-7.42 (m, 2H), 7.41-7.32 (m, 3H), 4.05 (s, 2H), 2.60 (t, J = 12.6 Hz, 2H), 2.38 (dt, J = 7.3, 2.5 Hz, 2H), 1.70-1.62 (m, 2H), 1.50-1.42 (m, 10H), 1.20-1.10 (m, 2H) ppm.
^{13}C NMR (101 MHz, CDCl$_3$): δ 154.9, 154.3 (dd, J = 291.4, 286.9 Hz), 140.6, 140.3, 132.7 (dd, J = 4.0, 3.8 Hz), 129.0, 128.7 (t, J = 3.3 Hz), 127.6, 127.3, 127.1, 90.4 (dd, J = 22.0, 13.0 Hz), 79.4, 43.9 (br s), 34.4, 31.9, 28.6 ppm.
^{19}F NMR (376 MHz, CDCl$_3$): δ -90.06 (d, J = 42.2 Hz), -90.53 (d, J = 42.3 Hz) ppm.
All data matched that reported in the literature [S1]

Compound 9

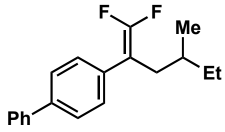

Purification by flash column chromatography (silica gel, petroleum ether) afforded 20.9 mg (73%) of the title compound **9**.
Physical State: colorless oil.
R$_f$ = 0.75 (petroleum ether).
^{1}H NMR (500 MHz, CDCl$_3$): δ 7.64-7.57 (m, 4H), 7.47-7.44 (m, 2H), 7.42-7.32 (m, 3H), 2.47-2.42 (m, 1H), 2.29-2.20 (m, 1H), 1.47-1.35 (m, 2H), 1.25-1.12 (m, 1H), 0.91-0.84 (m, 6H) ppm.
^{13}C NMR (126 MHz, CDCl$_3$): δ 154.2 (dd, J = 290.3, 286.2 Hz), 140.7, 140.0, 133.1 (dd, J = 4.0, 3.8 Hz), 128.9, 128.8 (t, J = 3.4 Hz), 127.5, 127.2, 127.2, 91.4 (dd, J = 21.9, 12.5 Hz), 34.6, 32.8 (t, J = 2.4 Hz), 29.2, 18.8, 11.4 ppm.
^{19}F NMR (471 MHz, CDCl$_3$): δ -91.01 (d, J = 43.5 Hz), -91.34 (d, J = 43.8 Hz) ppm.
HRMS (EI-TOF): calc'd for $C_{19}H_{20}F_2$ [M+Na]$^+$: 286.1533, found: 286.1525.
All data matched that reported in the literature [10]

Compound 10

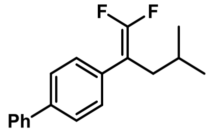

Purification by pTLC (Petroleum ether) afforded 16.8 mg (62%) of the title compound **10**.
Physical State: colorless oil.
R_f = 0.80 (petroleum ether).
^{1}H NMR (400 MHz, CDCl$_3$): δ 7.62 – 7.56 (m, 4H), 7.47 – 7.43(m, 2H), 7.42 – 7.33 (m, 3H), 2.31 (ddd, *J* = 7.3, 2.9, 2.0 Hz, 2H), 1.65 (tdd, *J* = 14.1, 7.1, 1.0 Hz, 1H), 0.91 (d, *J* = 6.6 Hz, 6H).
^{13}C NMR (101 MHz, CDCl$_3$): δ 154.27 (dd, *J* = 290.2, 286.6 Hz), 140.75, 140.08, 133.09 (dd, *J* = 4.5, 3.2 Hz), 128.94, 128.79 (t, *J* = 3.2 Hz), 127.50, 127.21, 127.16, 91.52 (dd, *J* = 22.0, 13.0 Hz), 36.67, 26.61 (t, *J* = 2.5 Hz), 22.23.
^{19}F NMR (376 MHz, CDCl$_3$): δ -91.00 (d, *J* = 43.6 Hz), -91.51 (d, *J* = 42.9 Hz).
All data matched that reported in the literature [10]

Compound 11

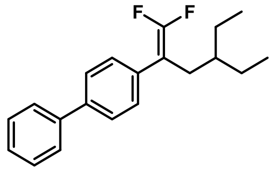

Purification by pTLC (Petroleum ether) afforded 14.7 mg (49%) of the title compound **11**.
Physical State: white solid.
R_f = 0.80 (petroleum ether).
^{1}H NMR (400 MHz, CDCl$_3$): δ 7.64 – 7.56 (m, 4H), 7.49 – 7.40 (m, 2H), 7.41 – 7.32 (m, 3H), 2.47 – 2.30 (m, 2H), 1.35 – 1.26 (m, 5H), 0.84 (t, *J* = 7.3 Hz, 6H).
^{13}C NMR (101 MHz, CDCl$_3$): δ 154.13 (t, *J* = 288.1 Hz), 140.75, 140.02, 133.06, 128.94, 128.79, 127.50, 127.19, 127.16, 91.58 (dd, *J* = 23.0, 12.3 Hz), 38.36, 31.30, 24.94, 10.61.
^{19}F NMR (376 MHz, CDCl$_3$): δ -91.21.
All data matched that reported in the literature [10]

Compound 12

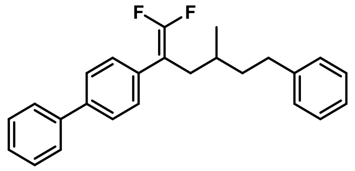

Purification by pTLC (Petroleum ether) afforded 13.4 mg (37%) of the title compound **12**
Physical State: colorless oil.
R_f = 0.80 (petroleum ether).
^{1}H NMR (500 MHz, CDCl$_3$): δ 7.63 – 7.59 (m, 2H), 7.58 – 7.54 (m, 2H), 7.48 – 7.42 (m, 2H), 7.36 – 7.32 (m, 2H), 7.26 – 7.21 (m, 2H), 7.18 – 7.13 (m, 1H), 7.12 – 7.09 (m, 2H), 2.64 (ddd, *J* = 13.6, 10.8, 5.6 Hz, 1H), 2.57 – 2.44 (m, 2H), 2.29 (dddd, *J* = 14.3, 8.1, 2.7, 1.4 Hz, 1H), 1.73 – 1.63 (m, 1H), 1.52 – 1.44 (m, 1H), 1.36 – 1.29 (m, 1H), 0.95 (d, *J* = 6.6 Hz, 3H).
^{13}C NMR (101 MHz, CDCl$_3$): δ 154.24 (dd, *J* = 290.2, 286.6 Hz), 142.71, 140.75, 140.14, 128.95, 128.81 (t, *J* = 3.2 Hz), 128.44, 127.52, 127.24, 127.17, 126.85, 125.79, 125.72, 113.99, 91.25 (dd, *J* = 21.5, 13.4 Hz), 38.34, 34.80, 33.36, 30.97 (t, *J* = 2.5 Hz), 19.36.
^{19}F NMR (376 MHz, CDCl$_3$): δ -90.88 (d, *J* = 43.6 Hz), -91.16 (d, *J* = 43.6 Hz).

HRMS (ESI-TOF): calc'd for $C_{25}H_{25}F_2$ [M+ H]$^+$: 363.1919, found: 363.1918.

Compound 13

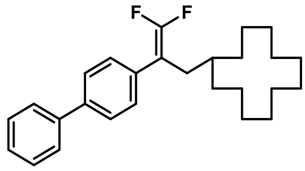

Purification by pTLC (Petroleum ether) afforded 13.8 mg (35%) of the title compound **13**.
Physical State: white solid.
R_f = 0.80 (petroleum ether).
^{1}H NMR (400 MHz, CDCl$_3$): δ 7.66 – 7.54 (m, 4H), 7.44 (tt, *J* = 8.3, 2.0 Hz, 2H), 7.40 – 7.30 (m, 3H), 2.33 (dt, *J* = 7.2, 2.4 Hz, 2H), 1.50 – 1.40 (m, 1H), 1.36 – 1.14 (m, 22H).
^{13}C NMR (126 MHz, CDCl$_3$): δ 154.10 (dd, *J* = 295.5, 285.3 Hz), 140.84, 140.06, 133.08 (d, *J* = 1.7 Hz), 128.94, 128.85 (t, *J* = 3.2 Hz), 127.48, 127.17, 91.58 (dd, *J* = 18.5, 15.6 Hz), 32.83, 32.17 (d, *J* = 2.3 Hz), 28.94, 24.78, 24.04, 23.56, 23.33, 21.67.
^{19}F NMR (376 MHz, CDCl$_3$): δ -91.32.
HRMS (ESI-TOF): calc'd for $C_{27}H_{35}F_2$ [M+ H]$^+$: 397.2701, found: 397.2700.

Compound 14

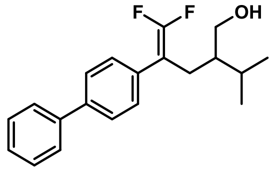

Purification by pTLC (10:1 petroleum ether : EtOAc) afforded 12.7 mg (40%) of the title compound **14**
Physical State: colorless oil.
R_f = 0.20 (10:1 petroleum ether : EtOAc).
^{1}H NMR (400 MHz, CDCl$_3$): δ 7.63 – 7.57 (m, 4H), 7.48 – 7.42 (m, 2H), 7.42 – 7.32 (m, 3H), 3.64 – 3.52 (m, 2H), 2.55 – 2.46 (m, 2H), 1.85 (ddd, *J* = 13.8, 6.8, 4.6 Hz, 1H), 1.47 – 1.36 (m, 1H), 0.93 (d, *J* = 6.8 Hz, 6H)
^{13}C NMR (101 MHz, CDCl$_3$): δ 154.99(dd, *J* = 290.3, 285.1 Hz), 140.65, 140.26, 132.52 (d, *J* = 3.6 Hz), 128.95, 128.76 (t, *J* = 3.2 Hz), 127.57, 127.33, 127.15, 91.32 (dd, *J* = 21.8, 13.2 Hz), 62.92, 44.53 (t, *J* = 2.3 Hz), 27.83, 26.06.
^{19}F NMR (376 MHz, CDCl$_3$): δ -90.40 (d, *J* = 43.6 Hz), -90.73 (d, *J* = 40.9 Hz).
HRMS (ESI-TOF): calc'd for $C_{20}H_{22}F_2O$ [M+ H]$^+$: 317.1711, found: 317.1710.

Compound 15

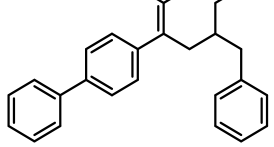

Purification by pTLC (10:1 petroleum ether : EtOAc) afforded 26.2 mg(Three parallel experiments) (24%) of the title compound **15**.

Physical State: colorless oil.

***R**$_f$* = 0.30 (10:1 petroleum ether : EtOAc).

^{1}H NMR (400 MHz, $CDCl_3$): δ 7.63 – 7.52 (m, 4H), 7.44 (ddd, *J* = 7.9, 6.3, 1.4 Hz, 2H), 7.38 – 7.32 (m, 1H), 7.31 – 7.26 (m, 2H), 7.24 (dd, *J* = 5.4, 2.0 Hz, 2H), 7.21 – 7.15 (m, 1H), 7.11 – 7.05 (m, 2H), 3.51 (qd, *J* = 10.8, 4.8 Hz, 2H), 2.74 – 2.63 (m, 2H), 2.59 (ddt, *J* = 14.5, 7.8, 2.3 Hz, 1H), 2.44 (ddt, *J* = 14.7, 6.7, 2.7 Hz, 1H), 1.92 – 1.76 (m, 1H).

^{13}C NMR (101 MHz, $CDCl_3$): δ 154.15 (dd, *J* = 291.7, 284.8 Hz), 140.60, 140.25, 132.29, 129.74, 129.23, 128.96, 128.68, 128.51, 127.58, 127.30, 127.14, 126.22, 90.85 (dd, *J* = 22.1, 13.1 Hz), 64.27, 40.85, 37.43, 28.88.

^{19}F NMR (376 MHz, $CDCl_3$): δ -90.04 (d, *J* = 10.9 Hz).

HRMS (ESI-TOF): calc'd for $C_{24}H_{22}F_2NaO$ [M+ Na]$^+$: 387.1531, found: 387.1531.

Compound 16-1

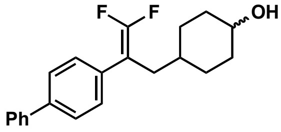

Purification by pTLC (4:1 petroleum ether : EtOAc) afforded 12.5 mg (38%) of the title compound **16-1**.

Physical State: white solid.

***R**$_f$* = 0.35 (4:1 petroleum ether : EtOAc).

^{1}H NMR (400 MHz, $CDCl_3$): δ 7.64-7.55 (m, 4H), 7.47-7.42 (m, 2H), 7.42-7.32 (m, 3H), 3.98-3.90 (m, 1H), 2.44-2.34 (m, 2H), 1.75-1.66 (m, 2H), 1.54-1.38 (m, 8H) ppm.

^{13}C NMR (101 MHz, $CDCl_3$): δ154.3 (dd, *J* = 290.8, 286.7 Hz), 140.7, 140.1, 133.0 (dd, *J* = 4.0, 3.0 Hz), 128.9, 128.7 (t, *J* = 3.4 Hz), 127.5, 127.2, 127.1, 90.8 (dd, *J* = 22.2, 12.5 Hz), 67.0, 34.4, 34.0, 32.2, 26.7 ppm.

^{19}F NMR (376 MHz, $CDCl_3$): δ -90.40 (d, *J* = 42.8 Hz), -90.98 (d, *J* = 42.6 Hz) ppm.

HRMS (ESI-TOF): calc'd for $C_{21}H_{22}F_2O$ [M+ Na]$^+$: 351.1531, found: 351.1529.

Compound 16-2

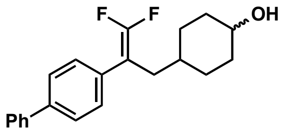

Purification by pTLC (4:1 petroleum ether : EtOAc) afforded 12.1 mg (37%) of the title compound **16-2**.

Physical State: white solid.

***R**$_f$* = 0.40 (4:1 petroleum ether : EtOAc).

^{1}H NMR (500 MHz, $CDCl_3$): δ 7.64-7.55 (m, 4H), 7.48-7.43 (m, 2H), 7.42-7.32 (m, 3H), 3.57-3.51 (m, 1H), 2.33 (dt, *J* = 7.3, 2.5 Hz, 2H), 1.99-1.90 (m, 2H), 1.82-1.73 (m, 2H), 1.59 (s, 1H),1.32-1.28 (m, 1H), 1.20-1.13 (m, 2H), 1.07-0.99 (m, 2H) ppm.

^{13}C NMR (126 MHz, CDCl$_3$): δ 154.2 (dd, *J* = 291.2, 286.6 Hz), 140.6, 140.2, 132.9 (dd, *J* = 4.0, 3.6 Hz), 128.9, 128.7 (t, *J* = 3.3 Hz), 127.6, 127.3, 127.1, 90.9 (dd, *J* = 22.2, 12.6 Hz), 71.0, 35.4, 35.0 (t, *J* = 2.3 Hz), 34.5, 30.9 ppm.
^{19}F NMR (376 MHz, CDCl$_3$): δ -90.41 (d, *J* = 42.7 Hz), -90.94 (d, *J* = 42.5 Hz) ppm.
HRMS (ESI-TOF): calc'd for $C_{21}H_{22}F_2O$ $[M+Na]^+$: 351.1531, found: 351.1530.

Compound 17

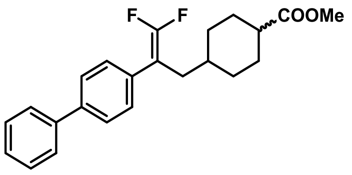

Purification by pTLC (20:1 petroleum ether : EtOAc) afforded 26.0 mg (70%) of the title compound **17 (**d.r. = 1:1).
Physical State: colorless oil.
R$_f$ = 0.20 (20:1 petroleum ether : EtOAc).
^{1}H NMR (500 MHz, CDCl$_3$): δ 7.64 – 7.58 (m, 7H), 7.49 – 7.43 (m, 5H), 7.41 – 7.35 (m, 6H), 3.69 (s, 3H), 2.51 (p, *J* = 5.0 Hz, 1H), 2.38 (dt, *J* = 7.5, 2.5 Hz, 2H), 2.22 (tt, *J* = 12.2, 3.7 Hz, 1H), 2.04 – 1.92 (m, 4H), 1.64 – 1.50 (m, 4H).
^{13}C NMR (126 MHz, CDCl$_3$): (mixed)
^{19}F NMR (471 MHz, CDCl$_3$): δ -90.41 (d, *J* = 42.9 Hz), -90.86 (d, *J* = 42.9 Hz).
HRMS (ESI-TOF): calc'd for $C_{24}H_{25}F_2O_2$ $[M+H]^+$: 371.1817, found: 371.1810.

Compound 18

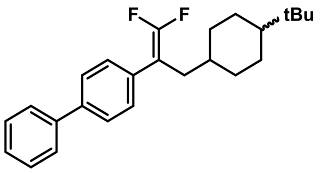

Purification by pTLC (Petroleum ether) afforded 29.4 mg (81%) of the title compound **18 (**d.r. = 4:3).
Physical State: white solid.
R$_f$ = 0.80 (petroleum ether).
^{1}H NMR (500 MHz, CDCl$_3$): δ 7.61 – 7.58 (m, 4H), 7.48 – 7.44 (m, 3H), 7.41 – 7.37 (m, 2H), 2.51 (ddd, *J* = 7.9, 2.7, 2.1 Hz, 2H), 1.78 – 1.72 (m, 2H), 1.64 (ddd, *J* = 13.6, 2.9, 1.7 Hz, 2H), 1.59 – 1.48 (m, 2H), 1.45 – 1.36 (m, 2H), 1.30 – 1.23 (m, 1H), 0.88 (s, 9H).
^{13}C NMR (126 MHz, CDCl$_3$): δ 154.20 (dd, *J* = 295.9, 286.1 Hz), 140.76, 140.09, 132.92 (dd,*J* = 4.4, 2.7 Hz), 128.94, 128.86 (t, *J* = 3.5 Hz), 127.50, 127.20, 127.16, 91.61 (dd, *J* = 22.3, 12.4 Hz), 48.52, 36.12 (t, *J* = 2.3 Hz), 35.24, 33.52, 30.12, 27.63, 21.75.
^{19}F NMR (471 MHz, CDCl$_3$): δ -91.16 (d, *J* = 43.9 Hz), -91.52 (d, *J* = 44.8 Hz).
HRMS (ESI-TOF): calc'd for $C_{25}H_{31}F_2$ $[M+H]^+$: 369.2388, found: 369.2384.

Compound 19

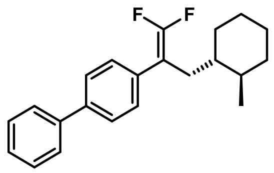

Purification by pTLC (Petroleum ether) afforded 17.9 mg (55%) of the title compound **19** (d.r. > 4:3)
Physical State: colorless oil.
***R**$_f$* = 0.75 (petroleum ether).
^{1}H NMR (400 MHz, CDCl$_3$): δ 7.65 – 7.56 (m, 4H), 7.44 (ddd, *J* = 7.8, 6.3, 1.3 Hz, 2H), 7.41 – 7.32 (m, 3H), 2.78 (ddt, *J* = 14.3, 4.9, 3.5 Hz, 1H), 2.09 (ddd, *J* = 14.3, 10.2, 2.1 Hz, 1H), 1.75 (dd, *J* = 11.8, 2.4 Hz, 1H), 1.63 (tdt, *J* = 12.5, 3.3, 1.5 Hz, 3H), 1.40 – 1.31 (m, 1H), 1.23 – 1.14 (m, 2H), 1.08 (ddt, *J* = 15.9, 12.5, 3.3 Hz, 1H), 0.94 (d, *J* = 6.5 Hz, 3H), 0.91 – 0.86 (m, 2H).
^{13}C NMR (126 MHz, CDCl$_3$): δ 154.14 (dd, *J* = 289.9, 286.3 Hz), 140.72, 139.93, 133.12 (dd,*J* = 4.4, 2.6 Hz), 128.94, 128.73 (t, *J* = 3.5 Hz), 127.49, 127.16 (d, *J* = 4.0 Hz), 91.16 (dd, *J* = 22.5, 12.1 Hz), 42.11, 37.60, 35.75, 31.94, 31.45, 26.49, 26.35, 20.39.
^{19}F NMR (376 MHz, CDCl$_3$): δ -90.87 (d, *J* = 43.6 Hz), -91.11 (d, *J* = 43.6 Hz).
HRMS (ESI-TOF): calc'd for $C_{22}H_{25}F_2$ [M+H]$^+$: 327.1919, found: 327.1917.

Compound 20

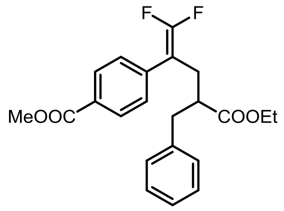

Purification by flash column chromatography (silica gel, petroleum ether : EtOAc) afforded 19.8 mg (51%) of the title compound **20**.
Physical State: white solid.
***R**$_f$* = 0.60 (10:1 petroleum ether : EtOAc).
^{1}H NMR (400 MHz, CDCl$_3$): δ 7.99 (d, *J* = 8.4 Hz, 2H), 7.30 – 7.15 (m, 5H), 7.10 – 7.02 (m, 2H), 3.95 (q, *J* = 7.2 Hz, 2H), 3.92 (s, 3H), 3.04 – 2.90 (m, 1H), 2.87 – 2.77 (m, 1H), 2.76 – 2.70 (m, 1H), 2.66 – 2.56 (m, 2H), 1.09 (t, *J* = 7.1 Hz, 3H) ppm.
^{13}C NMR (126 MHz, CDCl$_3$): δ 174.1, 166.6, 155.6 (dd, *J* = 293.0, 289.8 Hz), 138.4, 129.7, 129.2, 129.0, 128.8, 128.4, 128.2 (t, *J* = 3.3 Hz), 126.5, 60.5, 52.2, 45.7 (t, *J* = 2.6 Hz), 38.0, 29.7, 14.0 ppm.
^{19}F NMR (471 MHz, CDCl$_3$): δ -87.63 (d, *J* = 35.8 Hz), -88.21 (d, *J* = 34.6 Hz) ppm.
HRMS (EI-TOF): calc'd for $C_{22}H_{22}F_2O_4$ [M+Na]$^+$: 411.1378, found: 411.1383.

Compound 21

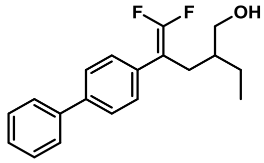

Purification by pTLC (10:1 petroleum ether : EtOAc) afforded 17.8 mg (59%) of the title compound **4**.

Physical State: colorless oil.

R_f = 0.30 (10:1 petroleum ether : EtOAc).

^{1}H NMR (400 MHz, CDCl$_3$): δ 7.64 – 7.56 (m, 4H), 7.49 – 7.42 (m, 2H), 7.44 – 7.33 (m, 3H), 3.55 (h, *J* = 5.5 Hz, 1H), 2.55 (ddt, *J* = 14.7, 7.5, 2.4 Hz, 1H), 2.44 (ddt, *J* = 14.5, 6.8, 2.4 Hz, 1H), 1.55 – 1.34 (m, 4H), 0.90 (t, *J* = 7.4 Hz, 3H).

^{13}C NMR (101 MHz, CDCl$_3$): δ 154.16 (dd, *J* = 290.4, 285.0 Hz), 140.64, 140.25, 132.69, 128.95, 128.73 (t, *J* = 3.2 Hz), 127.56, 127.32, 127.14, 90.98 (dd, *J* = 19.4, 15.6 Hz), 64.42, 40.37, 28.93, 23.31, 11.18.

^{19}F NMR (376 MHz, CDCl$_3$): δ -90.50.

HRMS (ESI-TOF): calc'd for $C_{19}H_{22}F_2O$ $[M+H]^+$: 303.1555, found: 303.1555.

Compound 22

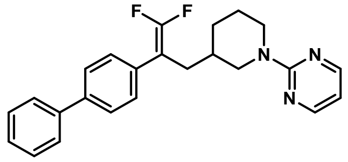

Purification by pTLC (10:1 petroleum ether : EtOAc) afforded 12.1 mg (32%) of the title compound **22**.

Physical State: colorless oil.

R_f = 0.35 (10:1 petroleum ether : EtOAc).

^{1}H NMR (400 MHz, CDCl$_3$): δ 8.26 (d, *J* = 4.8 Hz, 2H), 7.63 – 7.54 (m, 4H), 7.47 – 7.40 (m, 4H), 7.38 – 7.31 (m, 1H), 6.40 (t, *J* = 4.8 Hz, 1H), 4.59 (ddt, *J* = 13.0, 3.8, 1.7 Hz, 1H), 4.55 – 4.44 (m, 1H), 2.97 (ddd, *J* = 13.1, 11.3, 3.1 Hz, 1H), 2.76 (dd, *J* = 13.0, 10.1 Hz, 1H), 2.49 (ddt, *J* = 14.3, 6.7, 2.6 Hz, 1H), 2.36 (ddt, *J* = 14.3, 7.9, 2.1 Hz, 1H), 1.94 – 1.81 (m, 1H), 1.77 – 1.68 (m, 1H), 1.60 (dtd, *J* = 13.2, 6.5, 3.3 Hz, 1H), 1.50 – 1.38 (m, 1H), 1.32 – 1.27 (m, 1H).

^{13}C NMR (101 MHz, CDCl$_3$): δ 161.69, 157.80, 154.20 (dd, *J* = 292.5, 283.9 Hz), 140.70, 140.29, 132.65 (d, *J* = 2.3 Hz), 128.93, 128.91, 127.53, 127.30, 127.17, 109.40, 90.43 (dd, *J* = 22.4, 13.7 Hz), 49.51, 44.50, 34.08, 31.93, 30.75, 24.79.

^{19}F NMR (376 MHz, CDCl$_3$): δ -90.44 (d, *J* = 8.2 Hz).

HRMS (EI-TOF): calc'd for $C_{24}H_{24}F_2N_3Na$ $[M+Na]^+$: 414.1752, found: 414.1749.

Compound 23

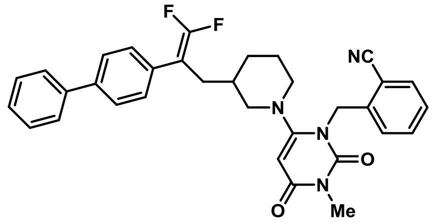

Purification by pTLC (5:1 petroleum ether : EtOAc) afforded 19.3 mg (35%) of the title compound **23**.

Physical State: colorless oil.

R$_f$ = 0.35 (5:1 petroleum ether : EtOAc).

^{1}H NMR (400 MHz, CDCl$_3$): δ 7.64 – 7.59 (m, 4H), 7.55 (dd, *J* = 7.8, 1.5 Hz, 1H), 7.49 – 7.41 (m, 3H), 7.39 – 7.33 (m, 1H), 7.31 – 7.27 (m, 3H), 7.04 (dd, *J* = 8.1, 4.5 Hz, 1H) , 5.34 (s, 1H), 5.16 (d, *J* = 3.8 Hz, 2H), 3.29 (s, 3H), 3.04 – 2.92 (m, 2H), 2.56 (t, *J* = 10.4 Hz, 1H), 2.43 – 2.19 (m, 3H), 1.82 (dd, *J* = 13.9, 3.2 Hz, 1H), 1.72 – 1.55 (m, 3H), 1.16 – 1.10 (m, 1H).

^{13}C NMR (101 MHz, CDCl$_3$): δ 163.27, 160.10, 154.16 (dd, *J* = 292.1, 284.3 Hz), 152.78, 140.89 (d, *J* = 6.4), 140.44 (d, *J* = 10.0 Hz), 133.18, 133.15, 132.04, 129.00, 128.52 (t, *J* = 3.4 Hz), 127.89, 127.69, 127.65, 127.46, 127.12, 126.52, 117.08, 110.83, 90.44, 89.69 (dd, *J* = 15.5, 15.9 Hz), 56.84, 52.33, 46.52, 34.16, 31.69, 30.09, 28.06, 24.45.

^{19}F NMR (376 MHz, CDCl$_3$): δ -89.34, -128.06.

HRMS (ESI-TOF): calc'd for $C_{33}H_{31}F_2N_4O_2$ $[M+H]^+$:553.2410, found: 553.2409.

Compound 24

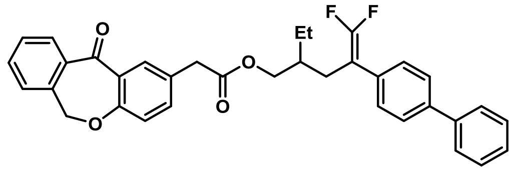

Purification by pTLC (20:1 petroleum ether : EtOAc) afforded 22.1 mg (40%) of the title compound **24**.

Physical State: colorless oil.

R$_f$ = 0.20 (20:1 petroleum ether : EtOAc).

^{1}H NMR (400 MHz, CDCl$_3$): δ 8.14 (d, *J* = 2.3 Hz, 1H), 7.88 (dd, *J* = 7.6, 1.5 Hz, 1H), 7.61 – 7.53 (m, 5H), 7.50 – 7.39 (m, 4H), 7.38 – 7.32 (m, 4H), 7.03 (d, *J* = 8.4 Hz, 1H), 5.17 (s, 2H), 4.08 – 3.97 (m, 2H), 3.64 (s, 2H), 2.56 – 2.33 (m, 2H), 1.71 – 1.61 (m, 1H), 1.44 – 1.30 (m, 2H), 0.86 (t, *J* = 7.5 Hz, 3H).

^{19}F NMR (376 MHz, CDCl$_3$): δ -90.12 (dd, *J* = 40.9, 42.0 Hz，).

HRMS (ESI-TOF): calc'd for $C_{35}H_{31}F_2O_4$ $[M+H]^+$: 553.2185, found: 553.2184.

Compound 25

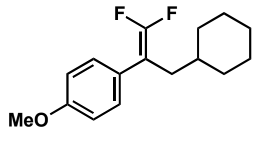

Purification by pTLC (30:1 petroleum ether : EtOAc) afforded 17.8 mg (67%) of the title compound **25**.

Physical State: colorless oil.

R$_f$ = 0.40 (30:1 petroleum ether : EtOAc).

^{1}H NMR (400 MHz, CDCl$_3$): δ 7.23 (d, *J* = 8.2 Hz, 2H), 6.89 (d, *J* = 8.8 Hz, 2H), 3.82 (s, 3H), 2.25-2.23 (m, 2H), 1.74-1.60 (m, 5H), 1.29-1.24 (m, 1H), 1.17-1.06 (m, 3H), 0.97-0.87 (m, 2H) ppm.

^{13}C NMR (101 MHz, CDCl$_3$): δ 158.7, 154.0 (dd, *J* = 288.8, 285.3 Hz), 129.5 (t, *J* = 3.2 Hz), 126.4 (dd, *J* = 3.1,3.0 Hz), 114.0, 90.6 (dd, *J* = 22.1, 13.0 Hz), 55.3, 35.8 (t, *J* = 2.4 Hz), 35.5, 33.0, 26.6, 26.2 ppm.

^{19}F NMR (376 MHz, CDCl$_3$): δ -92.36 (d, *J* = 46.5 Hz), -92.77 (d, *J* = 46.6 Hz) ppm.

All data matched that reported in the literature [S1]

Compound 26

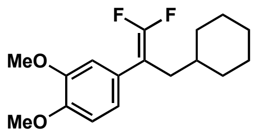

Purification by pTLC (30:1 petroleum ether : EtOAc) afforded 20.4 mg (69%) of the title compound **26**.

Physical State: colorless oil.

R_f = 0.30 (30:1 petroleum ether : EtOAc).

^{1}H NMR (400 MHz, CDCl$_3$): δ 6.85 (d, *J* = 1.2 Hz, 2H), 6.82 (s, 1H), 3.89 (s, 3H), 3.88 (s, 3H), 2.23 (dt, *J* = 7.2, 2.5 Hz, 2H), 1.74-1.59 (m, 5H), 1.30-1.21 (m, 1H), 1.16-1.09 (m, 3H), 0.98-0.86 (m, 2H) ppm.

^{13}C NMR (126 MHz, CDCl$_3$): δ 154.0 (dd, *J* = 291.2, 286.6 Hz), 148.8, 148.2, 126.8, 120.9 (t, *J* = 3.2 Hz), 111.8 (t, *J* = 3.5 Hz), 111.1, 90.9 (dd, *J* = 19.7, 15.2 Hz), 56.1, 56.0, 35.9 (t, *J* = 2.5 Hz), 35.6, 33.0, 26.6, 26.2 ppm.

^{19}F NMR (376 MHz, CDCl$_3$): δ -92.12 ppm.

HRMS (ESI-TOF): calc'd for $C_{17}H_{22}F_2O_2$ $[M+Na]^+$: 319.1480, found: 319.1481.

Compound 27

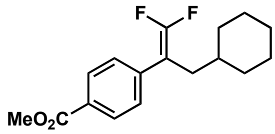

Purification by pTLC (30:1 petroleum ether : EtOAc) afforded 24.1 mg (82%) of the title compound **27**.

Physical State: colorless oil.

R_f = 0.40 (30:1 petroleum ether : EtOAc).

^{1}H NMR (400 MHz, CDCl$_3$): δ 8.01 (d, *J* = 8.4 Hz,2H), 7.38 (d, *J* = 7.0 Hz, 2H), 3.91 (s, 3H), 2.30 (dt, *J* = 7.3, 2.5 Hz, 2H), 1.72-1.52 (m, 5H), 1.27-1.19 (m, 1H), 1.15-1.04 (m, 3H), 0.97-0.83 (m, 2H) ppm.

^{13}C NMR (101 MHz, CDCl$_3$): δ 166.9, 154.3 (dd, *J* = 292.4, 287.5 Hz), 139.2 (dd, *J* = 4.9, 3.6 Hz), 129.8, 128.9, 128.3 (t, *J* = 3.5 Hz), 91.0 (dd, *J* = 22.8, 11.8 Hz), 52.2, 36.0 (t, *J* = 2.4 Hz), 35.0, 32.9, 26.4, 26.1 ppm.

^{19}F NMR (376 MHz, CDCl$_3$): δ -89.04 (d, *J* = 38.9 Hz), -89.71 (d, *J* = 39.1 Hz) ppm.

HRMS (ESI-TOF): calc'd for $C_{17}H_{20}F_2O_2$ $[M+H]^+$: 295.1504, found: 295.1505.

Compound 28

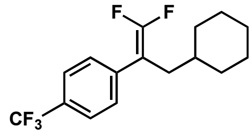

Purification by flash column chromatography (silica gel, petroleum ether) afforded 25.8 mg (85%)

of the title compound **28**.
Physical State: colorless oil.
R_f = 0.75 (petroleum ether).
^{1}H NMR (400 MHz, CDCl$_3$): δ 7.60 (d, *J* = 8.1 Hz, 2H), 7.42 (d, *J* = 8.1 Hz, 2H), 2.30 (dt, *J* = 7.3, 2.5 Hz, 2H), 1.73-1.57 (m, 5H), 1.25-1.17 (m, 1H), 1.15-1.06 (m, 3H), 0.96-0.86 (m, 2H) ppm.
^{13}C NMR (101 MHz, CDCl$_3$): δ 154.4 (dd, *J* = 292.0, 287.5 Hz), 138.1, 129.4 (q, *J* = 32.6 Hz), 128.7 (t, *J* = 3.3 Hz), 125.5 (q, *J* = 3.9 Hz), 124.2 (q, *J* = 271.9 Hz), 90.7 (dd, *J* = 23.2, 11.9 Hz), 35.9 (t, *J* = 2.4 Hz), 35.1, 33.0, 26.5, 26.1 ppm.
^{19}F NMR (376 MHz, CDCl$_3$): δ -62.63, -89.21 (d, *J* = 39.6 Hz), -90.07 (d, *J* = 39.7 Hz) ppm.
HRMS (EI-TOF): calc'd for $C_{16}H_{17}F_5$ M: 304.1250, found: 304.1246.

Compound 29

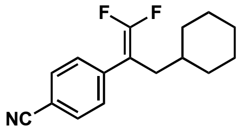

Purification by pTLC (30:1 petroleum ether : EtOAc) afforded 20.4 mg (78%) of the title compound **29**.
Physical State: colorless oil.
R_f = 0.60 (30:1 petroleum ether : EtOAc).
^{1}H NMR (400 MHz, CDCl$_3$): δ 7.64 (d, *J* = 8.4 Hz, 2H), 7.42 (d, *J* = 7.0 Hz, 2H), 2.29 (dt, *J* = 7.3, 2.5 Hz, 2H), 1.68-1.59 (m, 5H), 1.24-1.16 (m, 1H), 1.14-1.04 (m, 3H), 0.97-0.86 (m, 2H) ppm.
^{13}C NMR (101 MHz, CDCl$_3$): δ 154.4 (dd, *J* = 290.9, 289.9 Hz), 139.3 (dd, *J* = 4.8, 3.8 Hz), 132.3, 129.0 (t, *J* = 3.6 Hz), 118.8, 110.9, 90.7 (dd, *J* = 23.3, 11.3 Hz), 36.0 (t, *J* = 2.4 Hz), 34.8, 32.9, 26.4, 26.1 ppm.
^{19}F NMR (376 MHz, CDCl$_3$): δ -87.61 (d, *J* = 36.2 Hz), -88.66 (d, *J* = 36.4 Hz) ppm.
All data matched that reported in the literature [2]

Compound 30

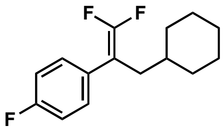

Purification by flash column chromatography (silica gel, petroleum ether) afforded 18.0 mg (71%) of the title compound **30**.
Physical State: colorless oil.
R_f = 0.80 (petroleum ether).
^{1}H NMR (400 MHz, CDCl$_3$): δ 7.34-7.19 (m, 2H), 7.12-6.94 (m, 2H), 2.24 (dt, *J* = 7.3, 2.5 Hz, 2H), 1.71-1.57 (m, 5H), 1.24-1.17 (m, 1H), 1.15-1.08 (m, 3H), 0.97-0.84 (m, 2H) ppm.
^{13}C NMR (101 MHz, CDCl$_3$): δ 163.1, 160.7, 157.3-151.1 (m), 130.1 (dt, *J* = 8.0, 3.4 Hz), 115.5 (d, *J* = 21.6 Hz), 90.4 (dd, *J* = 22.6, 12.8 Hz), 35.8 (t, *J* = 2.5 Hz), 35.5, 33.0, 26.5, 26.2 pm.
^{19}F NMR (376 MHz, CDCl$_3$): δ -91.48 (d, *J* = 44.4 Hz), -91.98 (d, *J* = 44.4 Hz), -114.96 ppm.
All data matched that reported in the literature [S1]

Compound 31

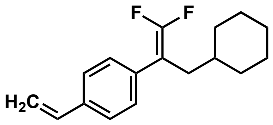

Purification by flash column chromatography (silica gel, petroleum ether) afforded 20.2 mg (77%) of the title compound **31**.

Physical State: colorless oil.

R

R_f = 0.85 (petroleum ether).

^{1}H NMR (400 MHz, $CDCl_3$): δ 7.39 (d, *J* = 8.3 Hz, 2H), 7.27 (d, *J* = 9.4 Hz, 2H), 6.71 (dd, *J* = 17.6, 10.9 Hz, 1H), 5.76 (d, *J* = 17.6 Hz, 1H), 5.25 (d, *J* = 10.9 Hz, 1H), 2.27 (dt, *J* = 7.2, 2.5 Hz, 2H), 1.74-1.61 (m, 5H), 1.31-1.18 (m, 1H), 1.15-1.05 (m, 3H), 0.98-0.84 (m, 2H) ppm.

^{13}C NMR (101 MHz, $CDCl_3$): δ 154.2 (dd, *J* = 290.7, 286.1 Hz), 136.5, 133.7 (dd, *J* = 4.6, 3.8 Hz), 128.9, 128.5 (t, *J* = 3.4 Hz), 126.3, 114.1, 91.1 (dd, *J* = 22.6, 12.7 Hz), 35.9 (d, *J* = 2.2 Hz), 35.2, 33.0, 26.5, 26.2 ppm.

^{19}F NMR (376 MHz, $CDCl_3$): δ -90.83 (d, *J* = 43.4 Hz), -91.25 (d, *J* = 42.6 Hz) ppm.

All data matched that reported in the literature [S1]

Compound 32

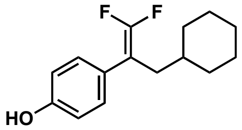

Purification by pTLC (5:1 petroleum ether : EtOAc) afforded 7.8 mg (31%) of the title compound **32**.

Physical State: colorless oil.

R_f = 0.40 (5:1 petroleum ether : EtOAc).

^{1}H NMR (400 MHz, $CDCl_3$): δ 7.18 (d, *J* = 8.0 Hz, 2H), 6.82 (d, *J* = 8.6 Hz, 2H), 5.05 (brs, 1H), 2.22 (dt, *J* = 7.3, 2.5 Hz, 2H), 1.78-1.53 (m, 5H), 1.27-1.21 (m, 1H), 1.16-1.05 (m, 3H), 0.95-0.85 (m, 2H) ppm.

^{13}C NMR (101 MHz, $CDCl_3$): δ 154.7, 154.0 (dd, *J* = 290.4, 286.3 Hz), 129.7 (t, *J* = 3.2 Hz), 126.6 (dd, *J* = 4.8, 2.5 Hz), 115.4, 90.6(dd, *J* = 20.6, 13.6 Hz), 35.8 (t, *J* = 2.4 Hz), 35.5, 33.0, 26.6, 26.2 ppm.

^{19}F NMR (376 MHz, $CDCl_3$): δ -92.33 (d, *J* = 46.4 Hz), -92.71 (d, *J* = 46.5 Hz) ppm.

HRMS (ESI-TOF): calc'd for $C_{15}H_{18}F_2O$ $[M+H]^+$: 253.1398, found: 253.1391.

Compound 33

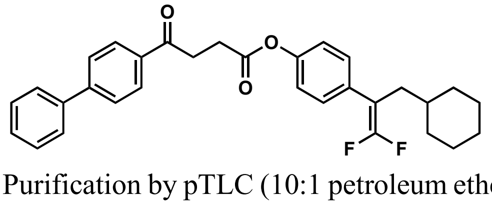

Purification by pTLC (10:1 petroleum ether : EtOAc) afforded 37.6 mg (77%) of the title compound **33**.

Physical State: white solid.

R_f = 0.50 (10:1 petroleum ether : EtOAc).

^{1}H NMR (400 MHz, $CDCl_3$): δ 8.09 (d, J = 8.3 Hz, 2H), 7.71 (d, J = 8.3 Hz, 2H), 7.64 (d, J = 7.3 Hz, 2H), 7.48 (t, J = 7.5 Hz, 2H), 7.42 (d, J = 7.3 Hz, 1H), 7.32 (d, J = 8.2 Hz, 2H), 7.13 (d, J = 8.6 Hz, 2H), 3.47 (t, J = 6.5 Hz, 2H), 3.05 (t, J = 6.5 Hz, 2H), 2.25 (dt, J = 7.3, 1.7 Hz, 2H), 1.75-1.61 (m, 5H), 1.31-1.21 (m, 1H), 1.20-1.04 (m, 3H), 1.00-0.84 (m, 2H) ppm.

^{13}C NMR (126MHz, $CDCl_3$): δ 197.6, 171.7, 154.1 (dd, J = 290.4, 286.0 Hz), 149.8, 146.2, 139.9, 135.3, 131.8 (dd, J=3.8, 2.3Hz), 129.4 (t, J = 3.3 Hz), 129.1, 128.8, 128.4, 127.4, 127.4, 121.6, 90.6 (dd, J = 22.6, 12.7 Hz), 35.7 (t, J = 2.7 Hz), 35.4, 33.6, 33.0, 28.7, 26.5, 26.1 ppm.

^{19}F NMR (376 MHz, $CDCl_3$): δ -90.99 (d, J = 43.6 Hz), -91.43 (d, J = 43.5 Hz) ppm.

HRMS (ESI-TOF): calc'd for $C_{31}H_{30}F_2O_3$ $[M+H]^+$: 489.2236, found: 489.2235.

Compound 34

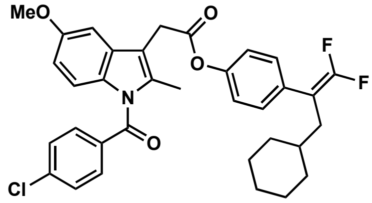

Purification by pTLC (10:1 petroleum ether : EtOAc) afforded 47.9 mg (81%) of the title compound **34**.

Physical State: colorless oil.

R_f = 0.50 (10:1 petroleum ether : EtOAc)).

^{1}H NMR (500 MHz, $CDCl_3$): δ 7.68 (d, J = 8.5 Hz, 2H), 7.48 (d, J = 8.5 Hz, 2H), 7.29 (d, J =7.6 Hz, 2H), 7.10-7.03 (m, 3H), 6.90 (d, J = 9.0 Hz, 1H), 6.70 (dd, J = 9.0, 2.5 Hz, 1H), 3.91 (s, 2H), 3.85 (s, 3H), 2.46 (s, 3H), 2.24 (dt, J = 7.4, 2.5 Hz, 2H), 1.71-1.61 (m, 5H), 1.28-1.19 (m, 1H), 1.15-1.07 (m, 3H), 0.95-0.86 (m, 2H) ppm.

^{13}C NMR (126 MHz, $CDCl_3$): δ 169.4, 168.4, 156.3, 154.1 (dd, J = 290.4, 286.3 Hz), 149.7, 139.5, 136.4, 133.9, 131.9 (dd, J = 3.7, 3.3 Hz), 131.3, 131.0, 130.6, 129.4 (t, J = 3.4 Hz), 129.3, 121.4, 115.2, 112.1, 111.9, 101.3, 90.5 (dd, J = 22.8, 12.5 Hz), 55.9, 35.7 (t, J = 2.4 Hz), 35.3, 32.9, 30.7, 26.5, 26.1, 13.5 ppm.

^{19}F NMR (376 MHz, $CDCl_3$): δ -90.86 (d, J = 43.0 Hz), -91.36 (d, J = 43.5 Hz) ppm.

All data matched that reported in the literature [S2]

Compound 35

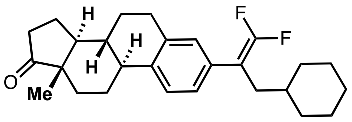

Purification by flash column chromatography (silica gel, petroleum ether) afforded 21.0mg (51%) of the title compound **35**.

Physical State: colorless oil.

R_f = 0.41 (petroleum ether).

^{1}H NMR (400 MHz, $CDCl_3$): δ 7.27 (d, J = 8.1 Hz, 1H), 7.09 (d, J = 8.2 Hz, 1H), 7.03 (s, 1H), 2.92 (dd, J = 9.0, 4.2 Hz, 2H), 2.63 – 2.39 (m, 2H), 2.31 (td, J = 11.0, 4.1 Hz, 1H), 2.24 (dt, J = 7.2, 2.4

Hz, 2H), 2.20 – 1.93 (m, 4H), 1.75 – 1.42 (m, 11H), 1.34-1.23 (m, 1H), 1.16-1.10 (m, 3H), 0.98-0.85 (m, 5H) ppm.
^{13}C NMR (101MHz, CDCl$_3$): δ 221.0, 154.1 (dd, *J* = 289.8, 285.9 Hz), 138.8, 136.6, 131.6 (dd, *J* =3.8, 2.6 Hz), 128.9 (t, *J* = 3.2 Hz), 125.8 (t, *J* = 3.2 Hz), 125.5, 90.8 (dd, *J* = 21.7, 12.6 Hz), 50.7, 48.1, 44.5, 38.2, 36.0, 35.7 (t, *J* = 2.8 Hz), 35.3, 33.0, 31.7, 29.6, 26.6, 26.6, 26.2, 25.7, 21.7, 14.0 ppm.
^{19}F NMR (376 MHz, CDCl$_3$): δ -91.47 (d, *J* = 45.0 Hz), -91.86 (d, *J* = 45.0 Hz) ppm.
HRMS (ESI-TOF): calc'd for $C_{27}H_{34}F_2O$ [M+H]$^+$: 413.2650, found: 413.2651.

Compound 36

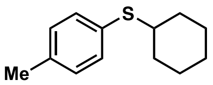

Purification by flash column chromatography (silica gel, petroleum ether) afforded 11.7 mg (57%) of the title compound **36**.
Physical State: colorless oil.
R$_f$ = 0.80 (petroleum ether).
^{1}H NMR (400 MHz, CDCl$_3$): δ 7.31 (d, *J* = 8.2 Hz, 2H), 7.10 (d, *J* = 7.9 Hz, 2H), 3.06-2.95 (m, 1H), 2.33 (s, 3H), 1.99-1.92 (m, 2H), 1.81-1.71 (m, 2H), 1.63-1.58 (m, 1H), 1.36- 1.25 (m, 5H) ppm.
^{13}C NMR (126 MHz, CDCl$_3$): δ 137.0, 132.9, 131.3, 129.7, 47.2, 33.5, 26.2, 25.9, 21.2 ppm.
All data matched that reported in the literature [3]

Compound 37

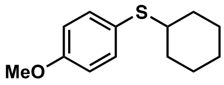

Purification by pTLC (100:1 petroleum ether : EtOAc) afforded 12.4 mg (56%) of the title compound **37**.
Physical State: colorless oil.
R$_f$ = 0.51 (100:1 petroleum ether : EtOAc).
^{1}H NMR (400 MHz, CDCl$_3$): δ 7.38 (d, *J* = 8.7 Hz, 2H), 6.84 (d, *J* = 8.8 Hz, 2H), 3.80 (s, 3H), 2.93-2.86 (m, 1H), 1.96-1.89 (m, 2H), 1.79-1.71 (m, 2H), 1.63-1.58 (m, 1H), 1.31-1.24 (m, 5H) ppm.
^{13}C NMR (101 MHz, CDCl$_3$): δ 159.4, 135.7, 125.1, 114.4, 55.4, 48.1, 33.5, 26.3, 25.9 ppm.
All data matched that reported in the literature [3]

Compound 38

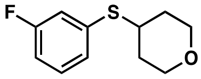

Purification by flash column chromatography (silica gel, petroleum ether : EtOAc)) afforded 11.2 mg (52%) of the title compound **38**.
Physical State: colorless oil.
R$_f$ = 0.30 (petroleum ether : EtOAc)).
^{1}H NMR (500 MHz, CDCl$_3$): δ 7.32-7.24 (m, 1H), 7.19 (dt, *J* = 7.8, 1.3 Hz, 1H), 7.13 (dt, *J* = 9.2, 2.2 Hz, 1H), 6.98-6.94 (m, 1H), 4.00 (dt, *J* = 11.8, 3.8 Hz, 2H), 3.47 (td, *J* = 11.2, 2.4 Hz, 2H), 3.37-

3.31 (m, 1H), 1.97-1.92 (m, 2H), 1.74-1.66 (m, 2H) ppm.
^{13}C NMR (126 MHz, CDCl$_3$): δ 162.8 (d, *J* = 248.6 Hz), 136.5 (d, *J* = 7.9 Hz), 130.3 (d, *J* = 8.6 Hz), 127.6 (d, *J* = 2.8 Hz), 118.6 (d, *J* = 22.0 Hz), 114.2 (d, *J* = 21.1 Hz), 67.4, 43.3, 33.1 ppm.
^{19}F NMR (471 MHz, CDCl$_3$): δ -112.28 ppm.
HRMS (ESI-TOF): calc'd for $C_{11}H_{13}FOS$ $[M+H]^+$: 213.0744, found: 213.0737.

Compound 39

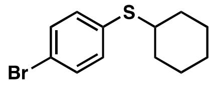

Purification by flash column chromatography (silica gel, petroleum ether) afforded 11.6 mg (43%) of the title compound **39**.
Physical State: colorless oil.
R$_f$ = 0.38 (petroleum ether).
^{1}H NMR (400 MHz, CDCl$_3$): δ 7.40 (d, *J* = 8.5 Hz, 2H), 7.25 (d, *J* = 8.6 Hz, 2H), 3.10-3.04 (m, 1H), 2.00-1.92 (m, 2H), 1.79-1.75 (m, 2H), 1.65-1.59 (m, 1H), 1.35-1.26 (m, 5H) ppm.
^{13}C NMR (101 MHz, CDCl$_3$): δ 134.5, 133.5, 132.0, 120.7, 46.9, 33.4, 26.1, 25.8 ppm.
All data matched that reported in the literature [4]

Compound 40

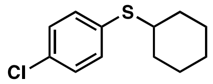

Purification by flash column chromatography (silica gel, petroleum ether) afforded 10.6 mg (47%) of the title compound **40**.
Physical State: colorless oil.
R$_f$ = 0.41 (petroleum ether).
^{1}H NMR (400 MHz, CDCl$_3$): δ 7.32 (d, *J* = 8.6 Hz, 2H), 7.25 (d, *J* = 8.6 Hz, 2H), 3.10-3.02 (m, 1H), 2.01-1.90 (m, 2H), 1.78-1.75 (m, 2H), 1.67-1.59 (m, 1H), 1.37-1.25 (m, 5H) ppm.
^{13}C NMR (101 MHz, CDCl$_3$): δ 133.8, 133.4, 132.8, 129.0, 47.0, 33.4, 26.2, 25.9 ppm.
All data matched that reported in the literature[4]

Compound 41

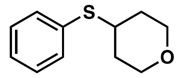

Purification by pTLC (30:1 petroleum ether : EtOAc) afforded 9.0 mg (46%) of the title compound **41**.
Physical State: colorless oil.
R$_f$ = 0.25 (30:1 petroleum ether : EtOAc).
^{1}H NMR (400 MHz, CDCl$_3$): δ 7.48-7.38 (m, 2H), 7.36-7.19 (m, 3H), 3.97 (dt, *J* = 11.7, 3.8 Hz, 2H), 3.43 (ddd, *J* = 11.6, 10.7, 2.4 Hz, 2H), 3.31-3.23 (m, 1H), 1.94-1.87 (m, 2H), 1.73-1.61 (m, 2H) ppm.
^{13}C NMR (101 MHz, CDCl$_3$): δ 135.4, 129.1, 128.2, 127.9, 68.2, 38.9, 34.1 ppm.
All data matched that reported in the literature [5]

Compound 42

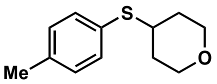

Purification by pTLC (30:1 petroleum ether : EtOAc) afforded 10.6 mg (51%) of the title compound **42**.

Physical State: colorless oil.

R_f = 0.25 (30:1 petroleum ether : EtOAc).

^{1}H NMR (400 MHz, $CDCl_3$): δ 7.34 (d, *J* = 8.1 Hz, 2H), 7.12 (d, *J* = 7.9 Hz, 2H), 3.96 (dt, *J* = 11.7, 3.7 Hz, 2H), 3.40 (td, *J* = 11.3, 2.4 Hz, 2H), 3.22-3.14 (m, 1H), 2.33 (s, 3H), 1.91-1.84 (m, 2H), 1.69 -1.61 (m, 2H) ppm.

^{13}C NMR (101 MHz, $CDCl_3$): δ 137.8, 133.6, 129.9, 129.8, 67.5, 44.0, 33.3, 21.2 ppm.

All data matched that reported in the literature [S5]

Compound 43

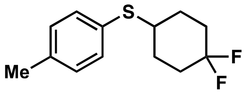

Purification by flash column chromatography (silica gel, petroleum ether) afforded 11.6 mg (48%) of the title compound **43**.

Physical State: colorless oil.

R_f = 0.42 (petroleum ether).

^{1}H NMR (400 MHz, $CDCl_3$): δ 7.33 (d, *J* = 8.1 Hz, 2H), 7.12 (d, *J* = 7.8 Hz, 2H), 3.19-3.12 (m, 1H), 2.34 (s, 3H), 2.20-2.10 (m, 2H), 2.02-1.96 (m, 2H), 1.85-1.68 (m, 4H) ppm.

^{13}C NMR (126 MHz, $CDCl_3$): δ 137.9, 133.4, 130.4, 129.9, 44.5, 32.4 (t, *J* = 24.4 Hz), 29.8, 28.8 (t, *J* = 5.0 Hz), 21.3 ppm.

^{19}F NMR (471 MHz, $CDCl_3$): δ -95.8 (d, *J* = 237.2 Hz), -98.1 (d, *J* = 233.4 Hz) ppm.

HRMS (ESI-TOF): calc'd for $C_{13}H_{16}F_2S$ $[M+H]^+$: 243.1014, found: 243.1025.

Compound 44

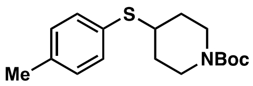

Purification by pTLC (20:1 petroleum ether : EtOAc) afforded 16.0 mg (52%) of the title compound **44**.

Physical State: colorless oil.

R_f = 0.25 (20:1 petroleum ether : EtOAc).

^{1}H NMR (400 MHz, $CDCl_3$): δ 7.33 (d, *J* = 8.1 Hz, 2H), 7.11 (d, *J* = 7.8 Hz, 2H), 3.95 (s, 2H), 3.15-3.08 (m, 1H), 2.94-2.83 (m, 2H), 2.33 (s, 3H), 1.91-1.86 (m, 2H), 1.52-1,47 (m, 2H), 1.44 (s, 9H) ppm.

^{13}C NMR (101 MHz, $CDCl_3$): δ 154.8, 137.8, 133.6, 130.1, 129.8, 79.7, 53.5, 45.1, 43.4, 32.3, 28.5, 21.2 ppm.

All data matched that reported in the literature [6]

Compound 45

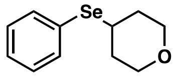

Purification by pTLC (30:1 petroleum ether : EtOAc) afforded 14.0 mg (58%) of the title compound **45**.

Physical State: colorless oil.

R_f = 0.25 (30:1 petroleum ether : EtOAc).

^{1}H NMR (400 MHz, CDCl$_3$): δ 7.60-7.53 (m, 2H), 7.33-7.23 (m, 3H), 3.93 (dt, J = 11.7, 3.8 Hz, 2H), 3.54-3.27 (m, 3H), 2.03-1.87 (m, 2H), 1.87-1.72 (m, 2H) ppm.

^{13}C NMR (101 MHz, CDCl$_3$): δ 135.4, 129.1, 128.2, 127.9, 68.2, 38.9, 34.1 ppm.

HRMS (ESI-TOF): calc'd for $C_{11}H_{14}OSe$ $[M+H]^+$:243.0283, found: 243.0275.

Compound 46

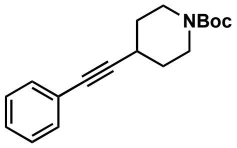

Purification by pTLC (10:1 petroleum ether : EtOAc) afforded 15.4 mg (54%) of the title compound **46**.

Physical State: colorless oil.

R_f = 0.55 (10:1 petroleum ether : EtOAc).

^{1}H NMR (400 MHz, CDCl$_3$): δ 7.43-7.36 (m, 2H), 7.31-7.25 (m, 3H), 3.77-3.70 (m, 2H), 3.31-3.20 (m, 2H), 2.80 (tt, J = 8.0, 4.0 Hz, 1H), 1.90-1.80 (m, 2H), 1.70- 1.64 (m, 2H), 1.46 (s, 9H) ppm.

^{13}C NMR (101 MHz, CDCl$_3$): δ 155.0, 131.7, 128.4, 127.9, 123.7, 91.9, 82.1, 79.6, 42.6(br), 31.6, 28.6, 27.7 ppm.

All data matched that reported in the literature [7]

Compound 47

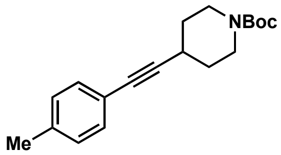

Purification by pTLC (10:1 petroleum ether : EtOAc) afforded 18.0 mg (60%) of the title compound **47**.

Physical State: colorless oil.

R_f = 0.55 (10:1 petroleum ether : EtOAc).

^{1}H NMR (400 MHz, CDCl$_3$): δ 7.28 (d, J = 7.9 Hz, 2H), 7.09 (d, J = 7.8 Hz, 2H), 3.78-3.67 (m, 2H), 3.24 (ddd, J = 13.5, 8.4, 3.4 Hz, 2H), 2.78 (tt, J = 8.1, 3.9 Hz, 1H), 2.33 (s, 3H), 1.88-1.80 (m, 2H), 1.67-1.62 (m, 2H), 1.46 (s, 9H) ppm.

^{13}C NMR (101 MHz, CDCl$_3$): δ 155.0, 137.9, 131.6, 129.1, 120.6, 91.1, 82.1, 79.6, 42.2(br),31.6, 28.6, 27.7, 21.5 ppm.

All data matched that reported in the literature [8]

Compound 48

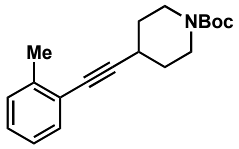

Purification by pTLC (10:1 petroleum ether : EtOAc) afforded 15.2 mg (51%) of the title compound **48**.

Physical State: colorless oil.

R_f = 0.55 (10:1 petroleum ether : EtOAc).

^{1}H NMR (400 MHz, CDCl$_3$): δ 7.36 (d, J = 7.3 Hz, 1H), 7.21-7.15 (m, 2H), 7.14-7.08 (m, 1H), 3.75-3.65 (m, 2H), 3.31 (ddd, J = 13.4, 8.0, 3.5 Hz, 2H), 2.86 (tt, J = 7.9, 4.0 Hz, 1H), 2.41 (s, 3H), 1.96-1.81 (m, 2H), 1.77-1.60 (m, 2H), 1.47 (s, 9H) ppm.

^{13}C NMR (101 MHz, CDCl$_3$): δ 155.0, 140.1, 131.9, 129.5, 127.9, 125.6, 123.4, 96.0, 81.0, 79.6, 42.2(br), 31.7, 28.6, 27.8, 20.9 ppm.

HRMS (ESI-TOF): calc'd for $C_{19}H_{25}NO_2$ $[M+Na]^+$: 322.1778, found: 322.1777.

Compound 49

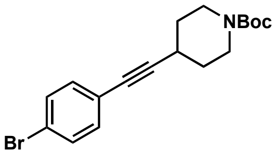

Purification by pTLC (10:1 petroleum ether : EtOAc) afforded 20.5 mg (57%) of the title compound **49**.

Physical State: colorless oil.

R_f = 0.50 (10:1 petroleum ether : EtOAc).

^{1}H NMR (400 MHz, CDCl$_3$): δ 7.41 (d, J = 8.5 Hz, 2H), 7.24 (d, J = 8.5 Hz, 2H), 3.79 – 3.68 (m, 2H), 3.22 (ddd, J = 13.5, 8.5, 3.4 Hz, 2H), 2.77 (tt, J = 8.1, 4.0 Hz, 1H), 1.89 – 1.80 (m, 2H), 1.70-1.60 (m, 2H), 1.46 (s, 9H) ppm.

^{13}C NMR (101 MHz, CDCl$_3$): δ 154.9, 133.2, 131.6, 122.7, 122.0, 93.2, 81.1, 79.7, 42.4(br), 31.4, 28.6, 27.8 ppm.

HRMS (ESI-TOF): calc'd for $C_{18}H_{22}BrNO_2$ $[M+H]^+$: 364.0907, found: 364.0908.

Compound 50

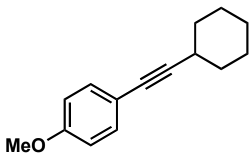

Purification by pTLC (30:1 petroleum ether : EtOAc) afforded 9.6 mg (45%) of the title compound **50**.

Physical State: colorless oil.

R_f = 0.20 (30:1 petroleum ether : EtOAc).

^{1}H NMR (400 MHz, CDCl$_3$): δ 7.33 (d, J = 8.9 Hz, 2H), 6.80 (d, J = 8.8 Hz, 2H), 3.79 (s, 3H), 2.61-2.50 (m, 1H), 1.92-1.82 (m, 2H), 1.80-1.70 (m, 2H), 1.58-1.45 (m, 3H), 1.41-1.29 (m, 3H) ppm.

^{13}C NMR (101 MHz, CDCl$_3$): δ 159.1, 133.0, 116.4, 113.9, 93.0, 80.3, 55.4, 33.0, 29.8, 26.1, 25.1

ppm.
All data matched that reported in the literature [9]

Compound 52

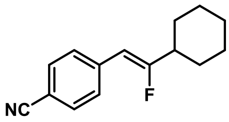

Purification by pTLC (30:1 petroleum ether : EtOAc) afforded 5.7 mg (25%) of the title compound **52**.
Physical State: colorless oil.
***R**$_f$* = 0.50 (30:1 petroleum ether : EtOAc).
^{1}H NMR (400 MHz, CDCl$_3$): δ 7.56 (q, *J* = 8.5 Hz, 4H), 5.47 (d, *J* = 39.4 Hz, 1H), 2.32 – 2.20 (m, 1H), 2.00 – 1.92 (m, 2H), 1.85 – 1.79 (m, 2H), 1.76 – 1.69 (m, 1H), 1.41 – 1.20 (m, 6H) ppm.
^{13}C NMR (101 MHz, CDCl$_3$): δ 167.9 (d, *J* = 273.5 Hz), 138.8 (d, *J* = 2.3 Hz), 132.1, 128.7 (d, *J* = 8.2 Hz), 119.1, 109.7, 102.7 (d, *J* = 8.4 Hz), 41.7 (d, *J* = 24.0 Hz), 29.9 (d, *J* = 2.3 Hz), 25.8, 25.8 ppm .
^{19}F NMR (471 MHz, CDCl$_3$): δ -99.19 (d, *J* = 15.9 Hz), -99.30 (d, *J* = 15.8 Hz) ppm.

Compound 54

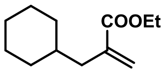

Purification by pTLC (30:1 petroleum ether : EtOAc) afforded 6.7 mg (37%) of the title compound **54**.
Physical State: colorless oil.
***R**$_f$* = 0.60 (30:1 petroleum ether : EtOAc).
^{1}H NMR (400 MHz, CDCl$_3$): δ 6.13 (d, *J* = 1.8 Hz, 1H), 5.64 – 5.35 (m, 1H), 4.19 (q, *J* = 7.1 Hz, 2H), 2.17 (dd, *J* = 7.0, 1.1 Hz, 2H), 1.75 – 1.53 (m, 5H), 1.50 – 1.38 (m, 1H), 1.29 (t, *J* = 7.1 Hz, 3H), 1.22 – 1.08 (m, 3H), 0.91 – 0.82 (m, 2H) ppm.
^{13}C NMR (101 MHz, CDCl$_3$): δ 167.5, 139.5, 125.4, 60.5, 39.9, 36.7, 33.1, 26.5, 26.2, 14.2 ppm.

Compound 55

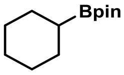

Purification by column chromatography (Petroleum ether) afforded 25 mg (60%) of the title compound **55**
Physical State: colorless oil.
***R**$_f$* = 0.50 (petroleum ether).
^{1}H NMR (400 MHz, CDCl$_3$) δ 1.69 – 1.62 (m, 2H), 1.60 – 1.53 (m, 2H), 1.41 – 1.23 (m, 6H), 1.22 (s, 12H), 1.03 – 0.91 (m, 1H).
^{13}C NMR (101 MHz, CDCl$_3$) δ 82.87, 28.10, 27.28, 26.90, 24.89. The signal of the α-B-carbon was not observed.
All data matched that reported in the literature[11]

Compound 56

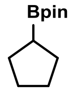

Purification by column chromatography (Petroleum ether) afforded 15.6 mg (Two parallel experiments) (20%) of the title compound **56**

Physical State: colorless oil.

***R**$_f$* = 0.50 (petroleum ether).

^{1}H NMR (500 MHz, CDCl$_3$) δ 1.78 – 1.69 (m, 2H), 1.61 – 1.38 (m, 6H), 1.23 (s, 12H), 1.20 – 1.13 (m, 1H).

^{13}C NMR (126 MHz, CDCl$_3$) δ 82.90, 28.64, 26.96, 24.86. The signal of the α-B-carbon was not observed.

All data matched that reported in the literature[12]

Compound 57

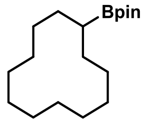

Purification by column chromatography (Petroleum ether) afforded 31.1 mg (53%) of the title compound **57**

Physical State: colorless oil.

***R**$_f$* = 0.50 (petroleum ether).

^{1}H NMR (500 MHz, CDCl$_3$) δ 1.45 – 1.35 (m, 10H), 1.34 – 1.26 (m, 12H), 1.23 (s, 12H), 0.92 – 0.85 (m, 1H).

^{13}C NMR (126 MHz, CDCl$_3$) δ 29.86, 25.05, 24.91, 24.41, 24.25, 23.60, 23.57, 23.53.

All data matched that reported in the literature[13]

Compound 58

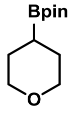

Purification by column chromatography (EA/PE=1:20) afforded 24.2 mg (32%) of the title compound **58**

Physical State: colorless oil.

***R**$_f$* = 0.40 (EA/PE=1:20).

^{1}H NMR (500 MHz, CDCl$_3$) δ 3.82 (dt, *J* = 11.2, 3.9 Hz, 2H), 3.52 – 3.41 (m, 2H), 1.68 – 1.53 (m, 5H), 1.24 (s, 12H).

^{13}C NMR (126 MHz, CDCl$_3$) δ 83.27, 68.98, 27.75, 24.90. The signal of the α-B-carbon was not observed.

All data matched that reported in the literature[13]

Compound 59

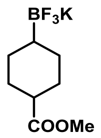

24.2mg (46%).

Physical State: white solid.

^{1}H NMR (500 MHz, DMSO-d_6) δ 3.54 (s, 3H), 2.09 (tt, J = 12.3, 3.5 Hz, 1H), 1.76 (d, J = 12.3 Hz, 2H), 1.62 – 1.50 (m, 2H), 1.12 (qd, J = 12.8, 3.4 Hz, 2H), 0.91 (qd, J = 13.3, 3.4 Hz, 2H), -0.03 – -0.17 (m, 1H).

^{13}C NMR (126 MHz, DMSO-d_6) δ 176.43, 51.00, 43.79, 30.73, 27.81, 27.81.

All data matched that reported in the literature[14]

NMR Spectra

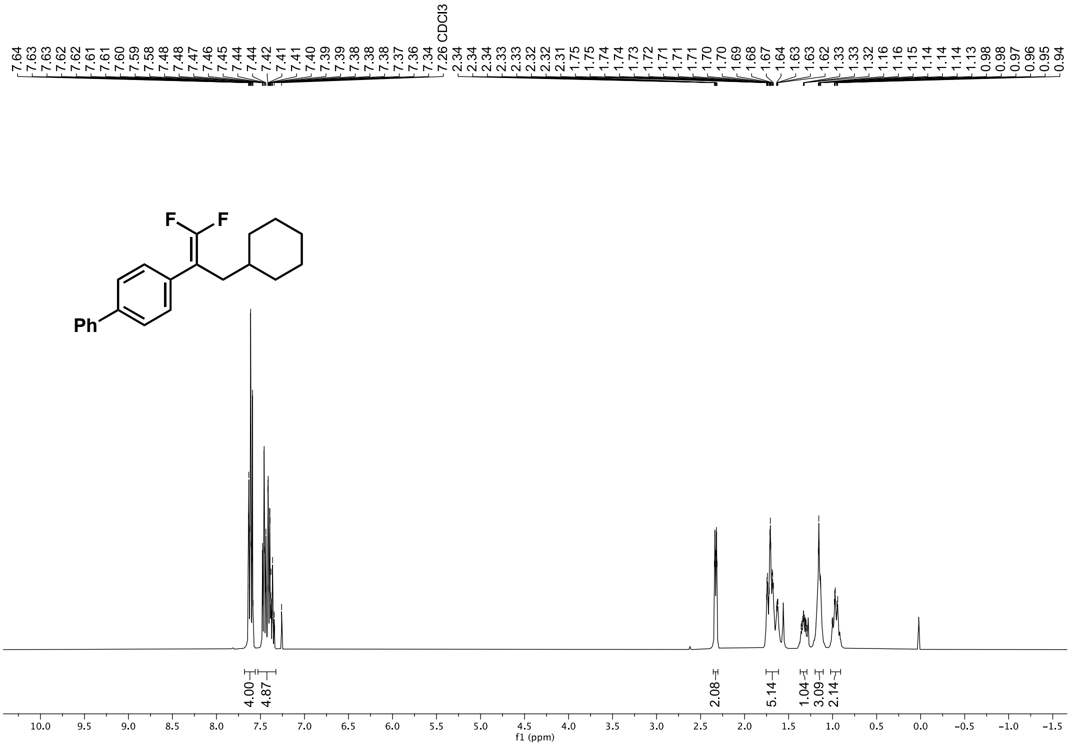

Supplementary Figure 4. ^{1}H NMR Spectra of product **3**

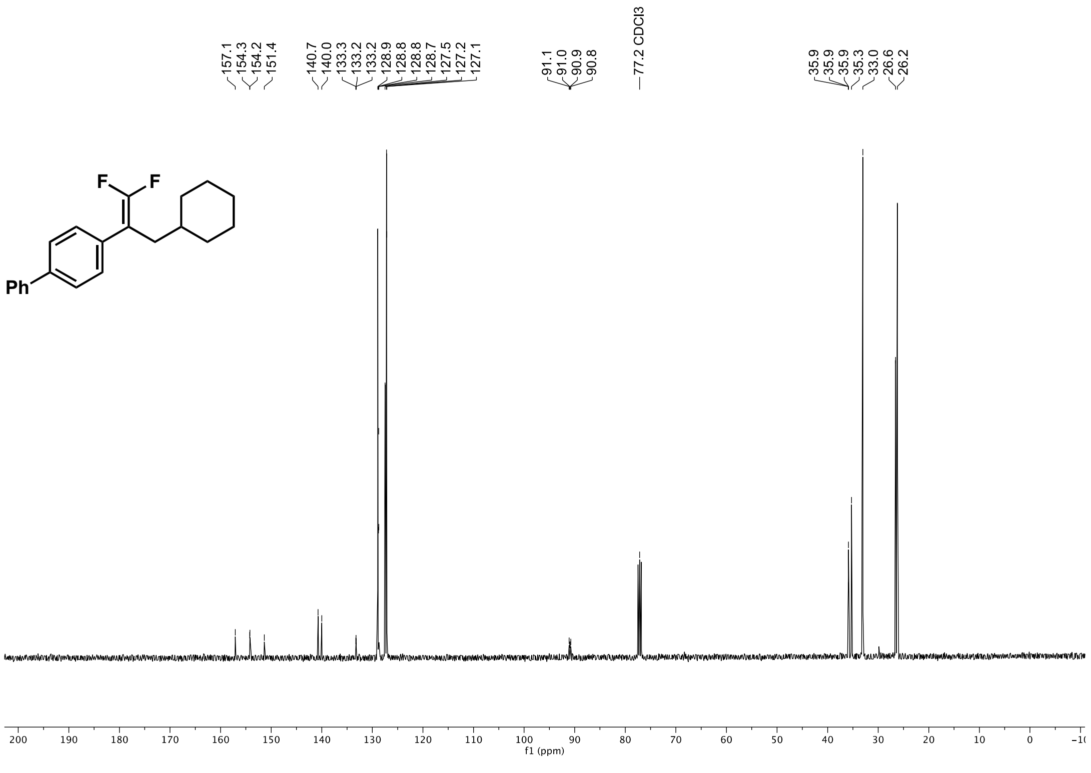

Supplementary Figure 5. ^{13}C NMR Spectra of product **3**

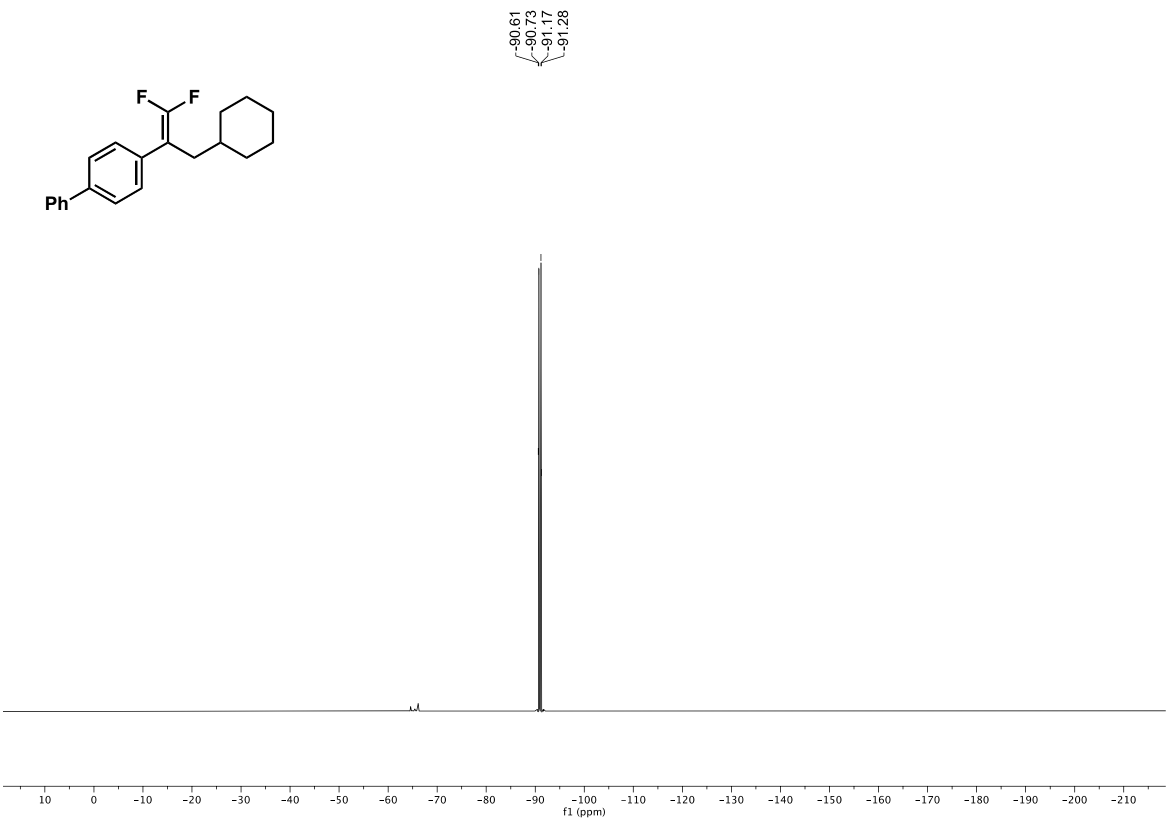

Supplementary Figure 6. ^{19}F NMR Spectra of product **3**

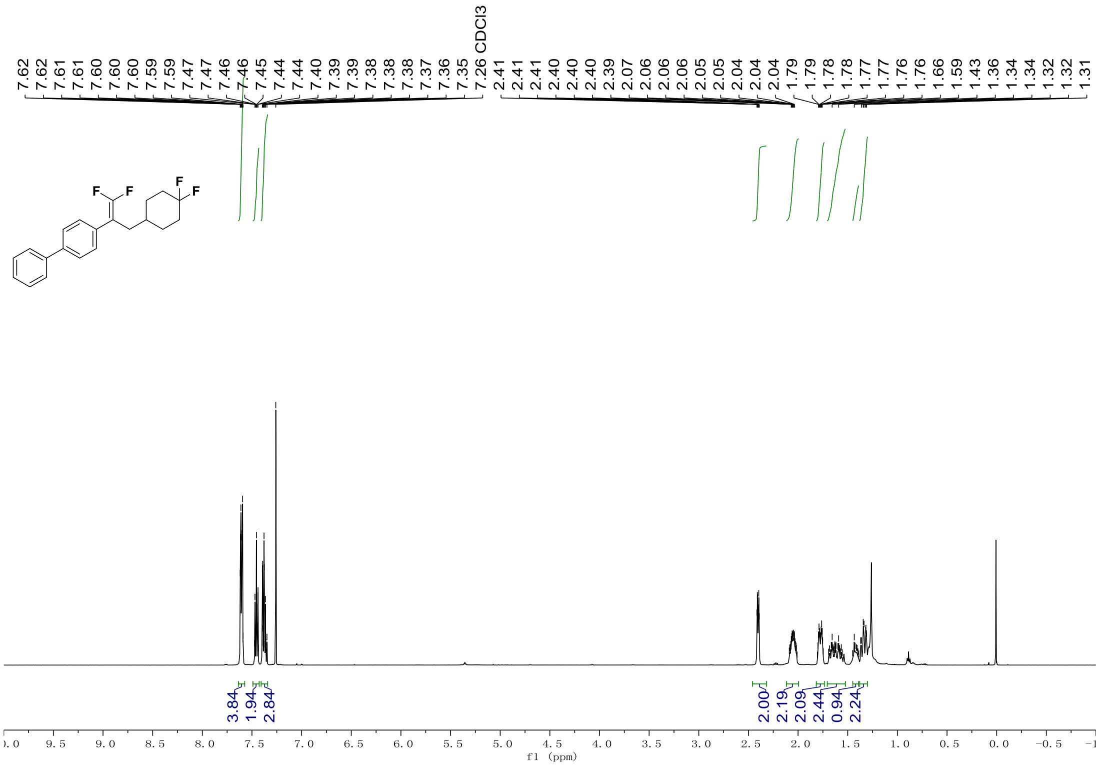

Supplementary Figure 7. ^{1}H NMR Spectra of product **4**

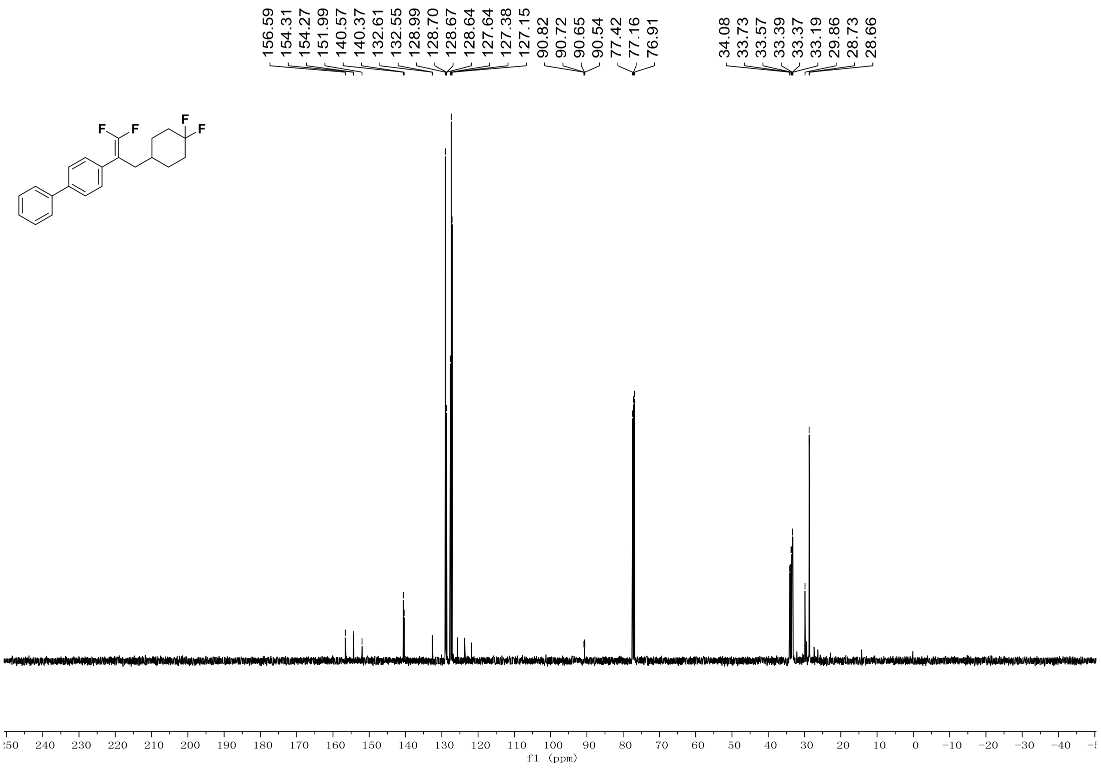

Supplementary Figure 8. ^{13}C NMR Spectra of product **4**

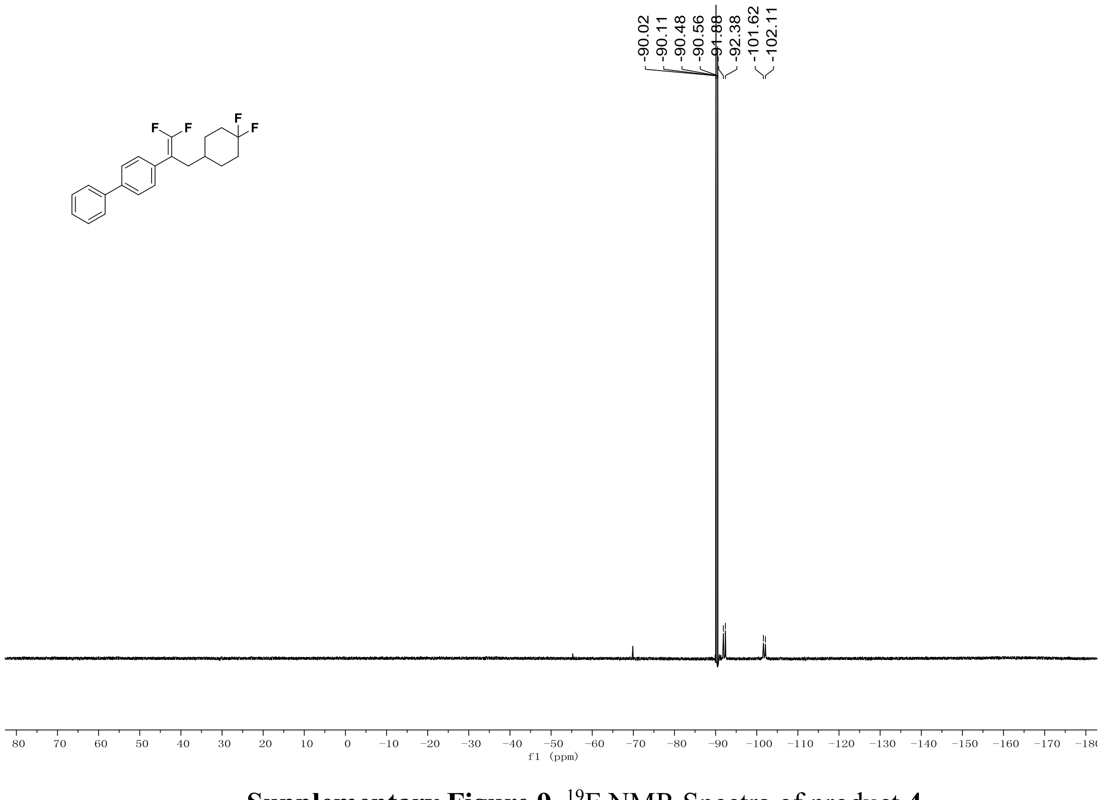

Supplementary Figure 9. ^{19}F NMR Spectra of product **4**

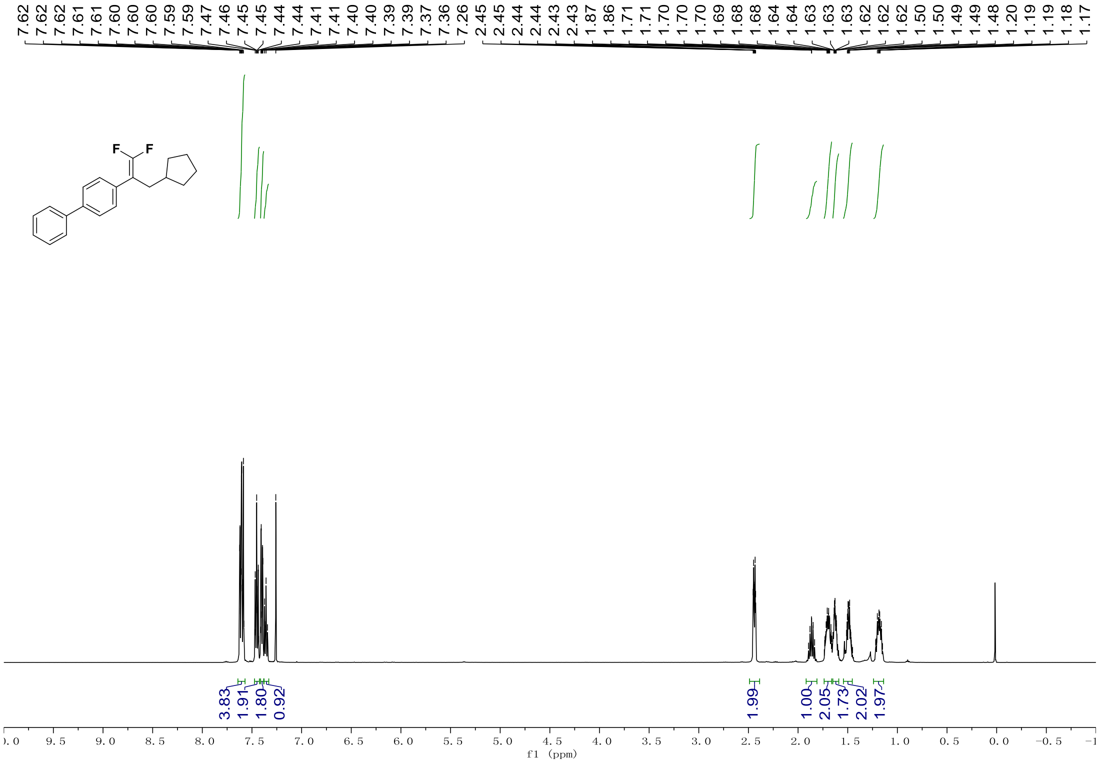

Supplementary Figure 10. ^{1}H NMR Spectra of product **5**

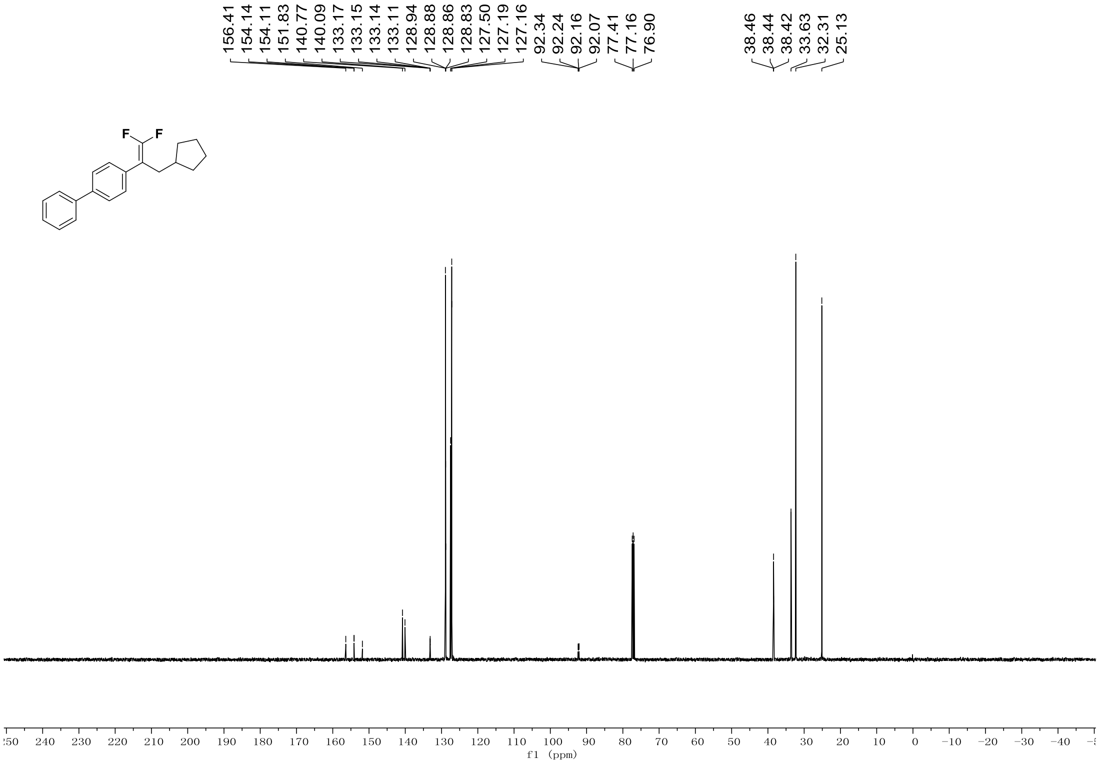

Supplementary Figure 11. ^{13}C NMR Spectra of product **5**

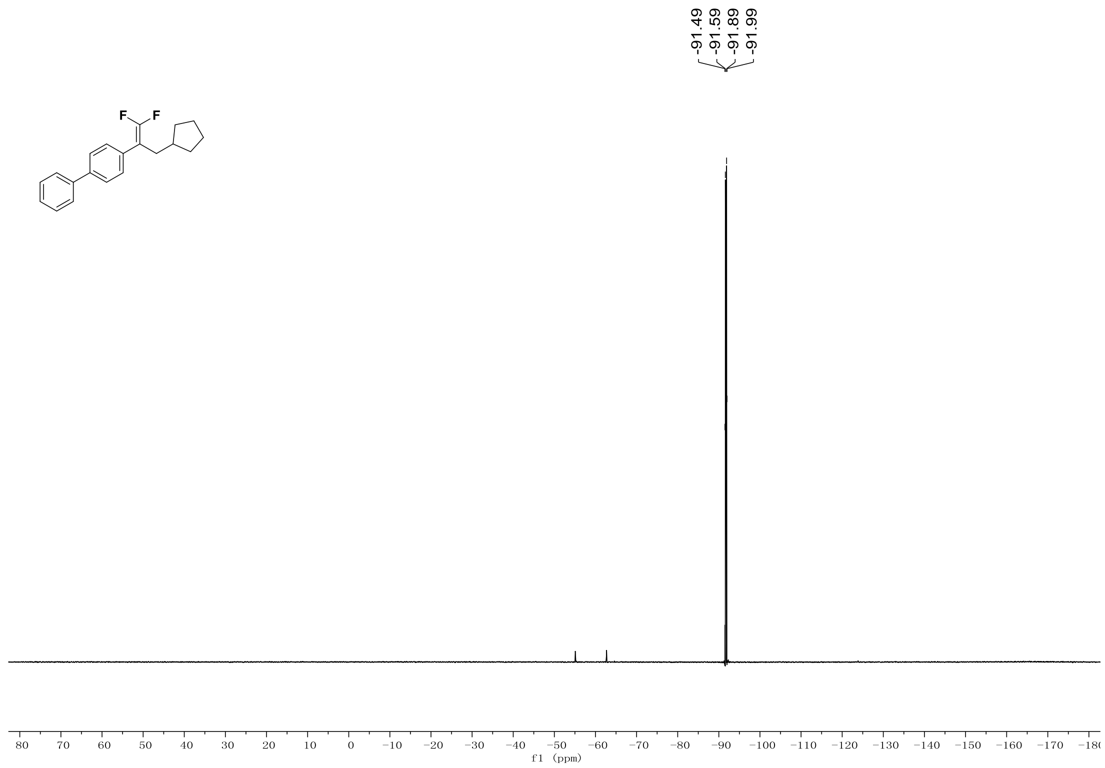

Supplementary Figure 12. ^{19}F NMR Spectra of product **5**

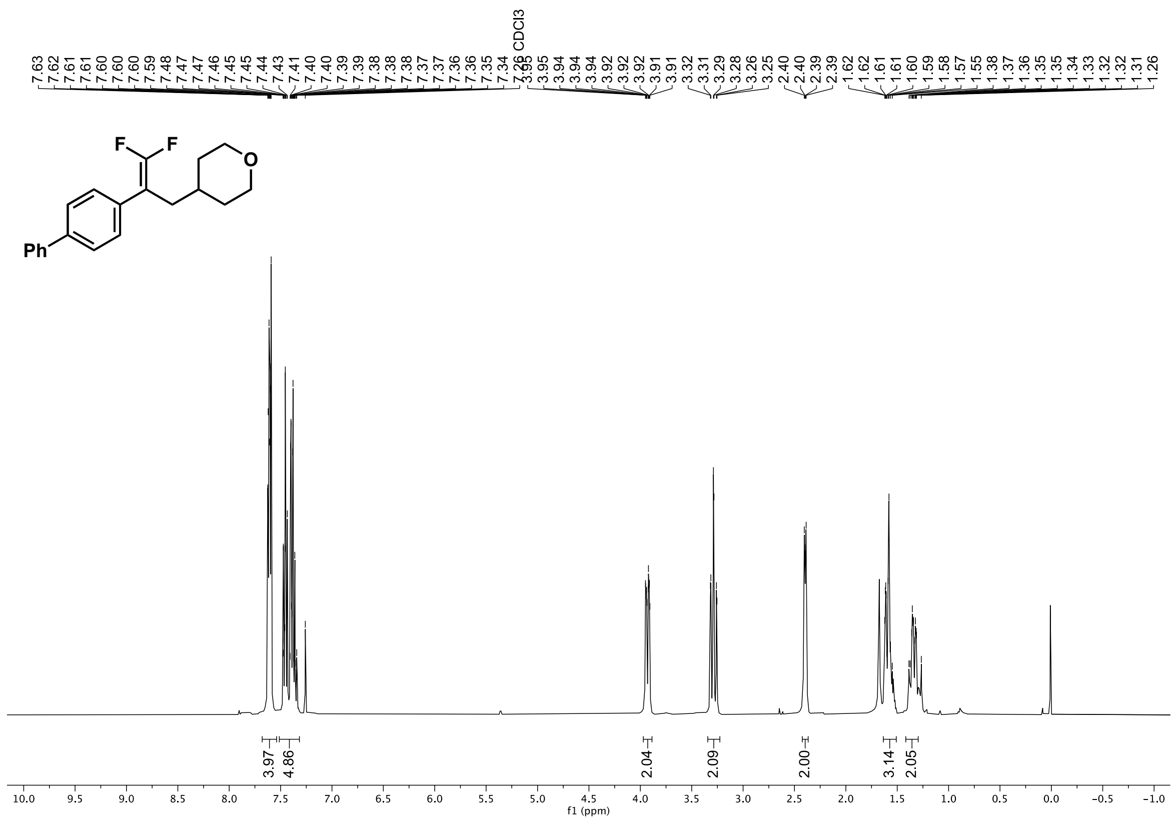

Supplementary Figure 13. ^{1}H NMR Spectra of product **6**

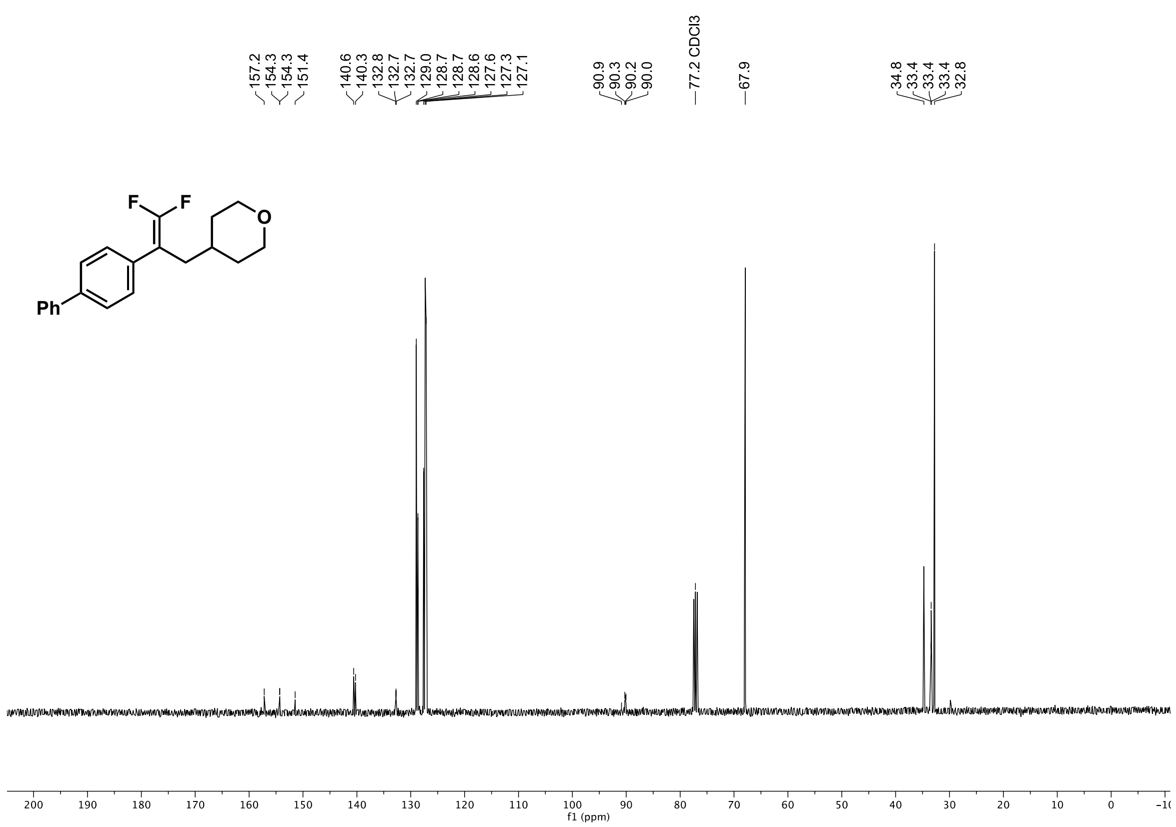

Supplementary Figure 14. ^{13}C NMR Spectra of product **6**

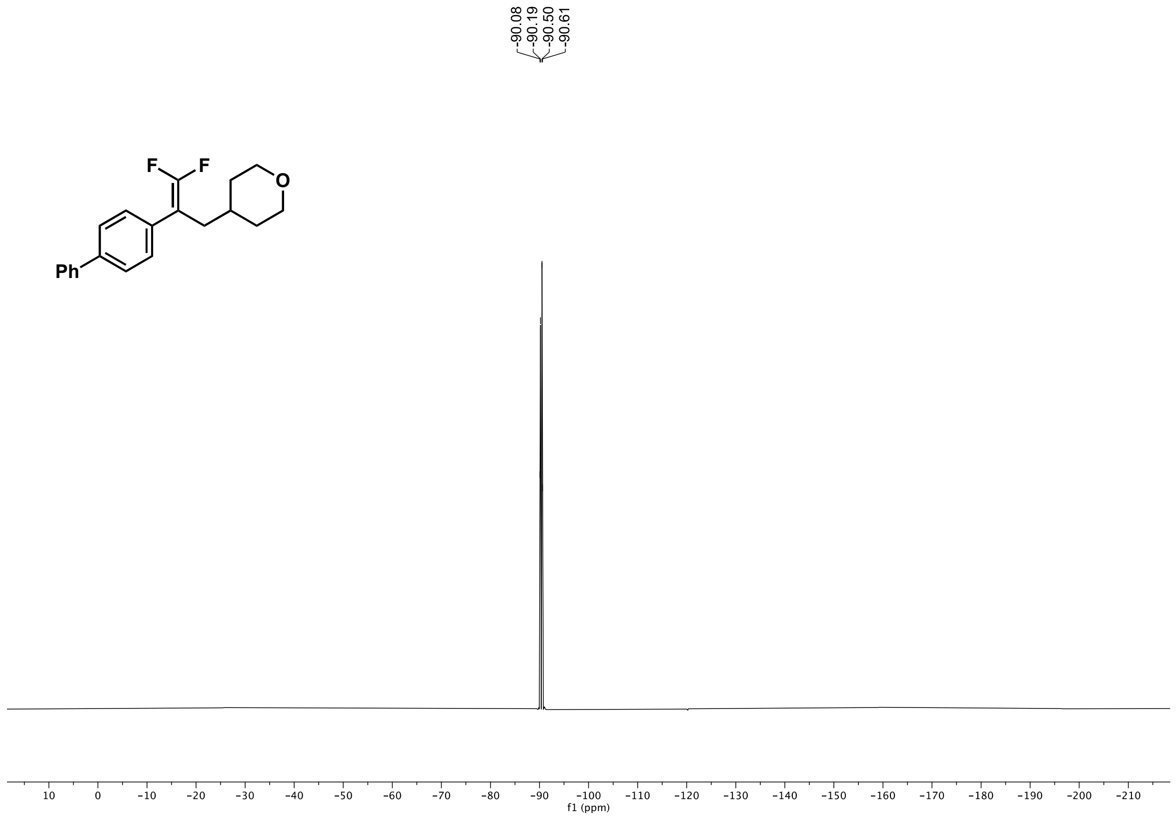

Supplementary Figure 15. ^{19}F NMR Spectra of product **6**

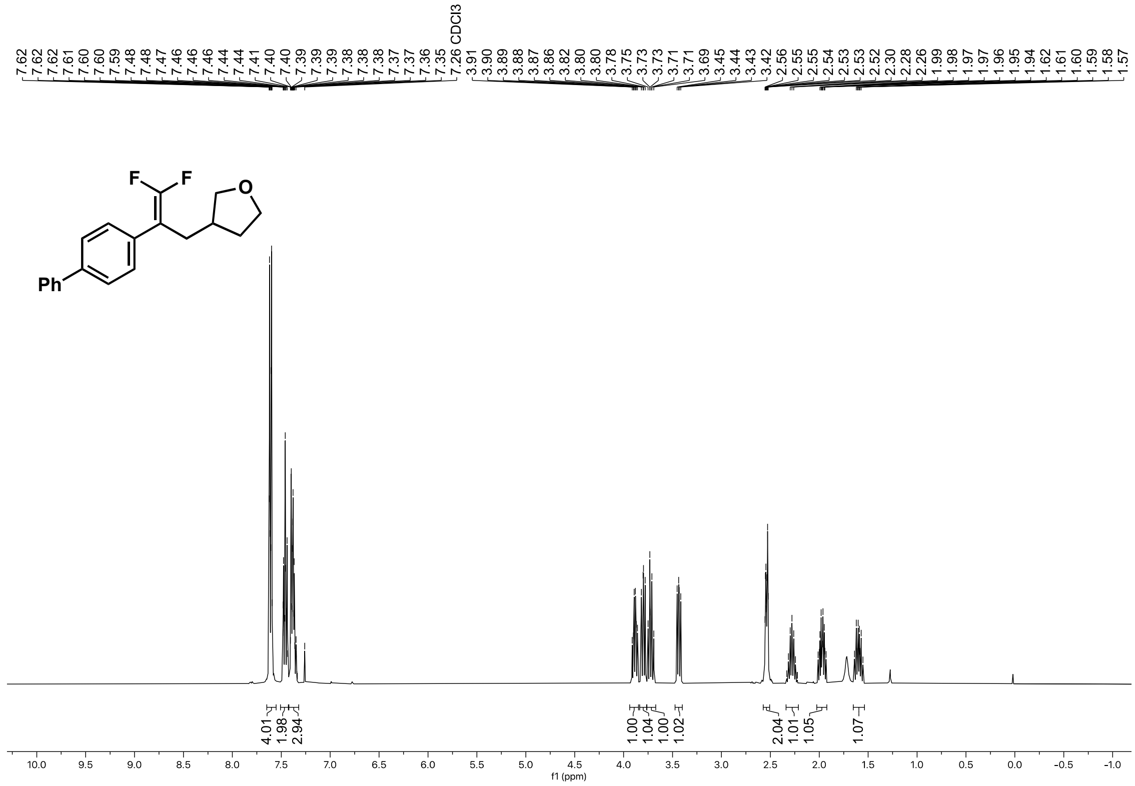

Supplementary Figure 16. ^{1}H NMR Spectra of product **7**

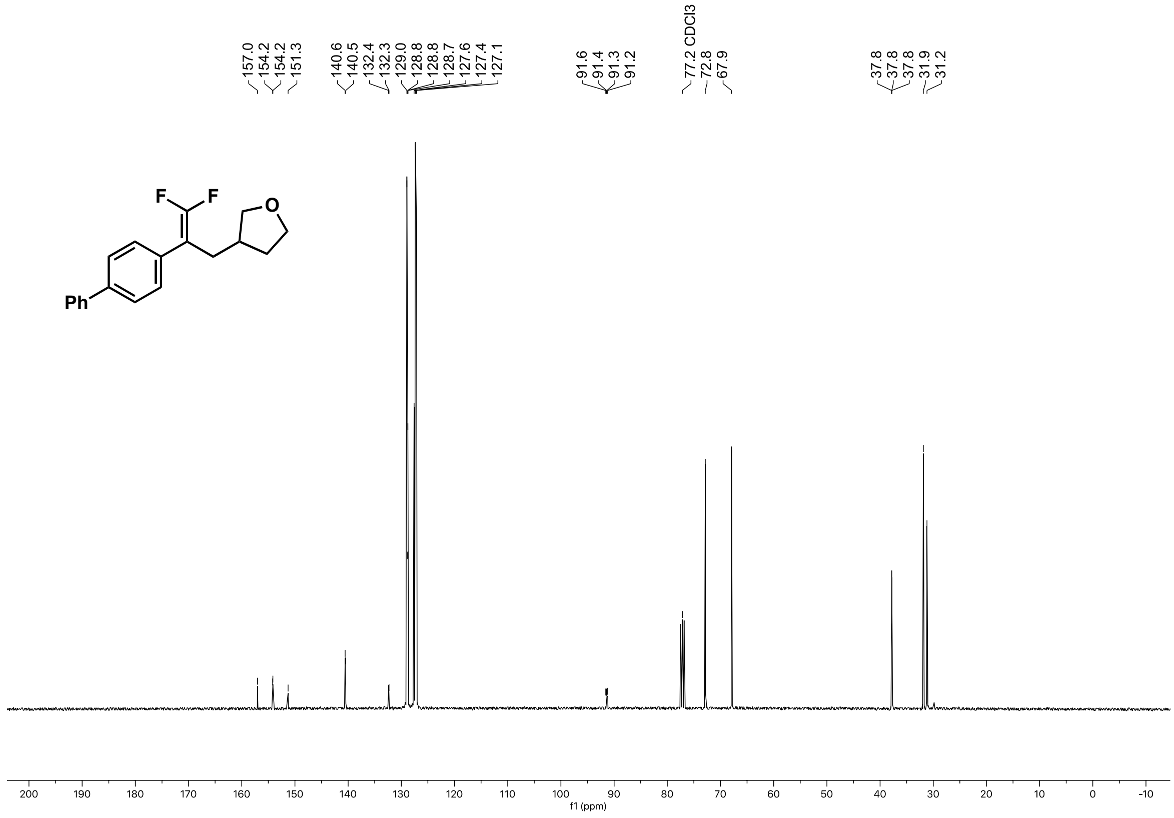

Supplementary Figure 17. ^{13}C NMR Spectra of product **7**

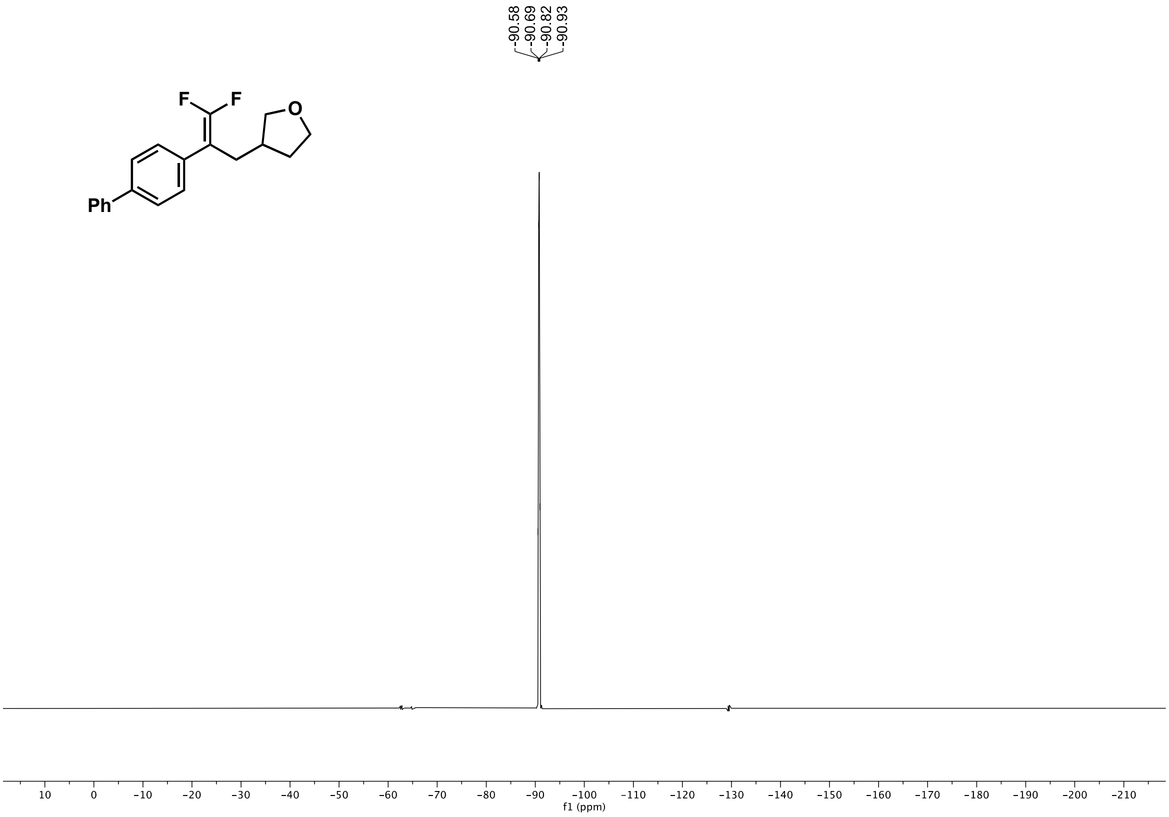

Supplementary Figure 18. ^{19}F NMR Spectra of product **7**

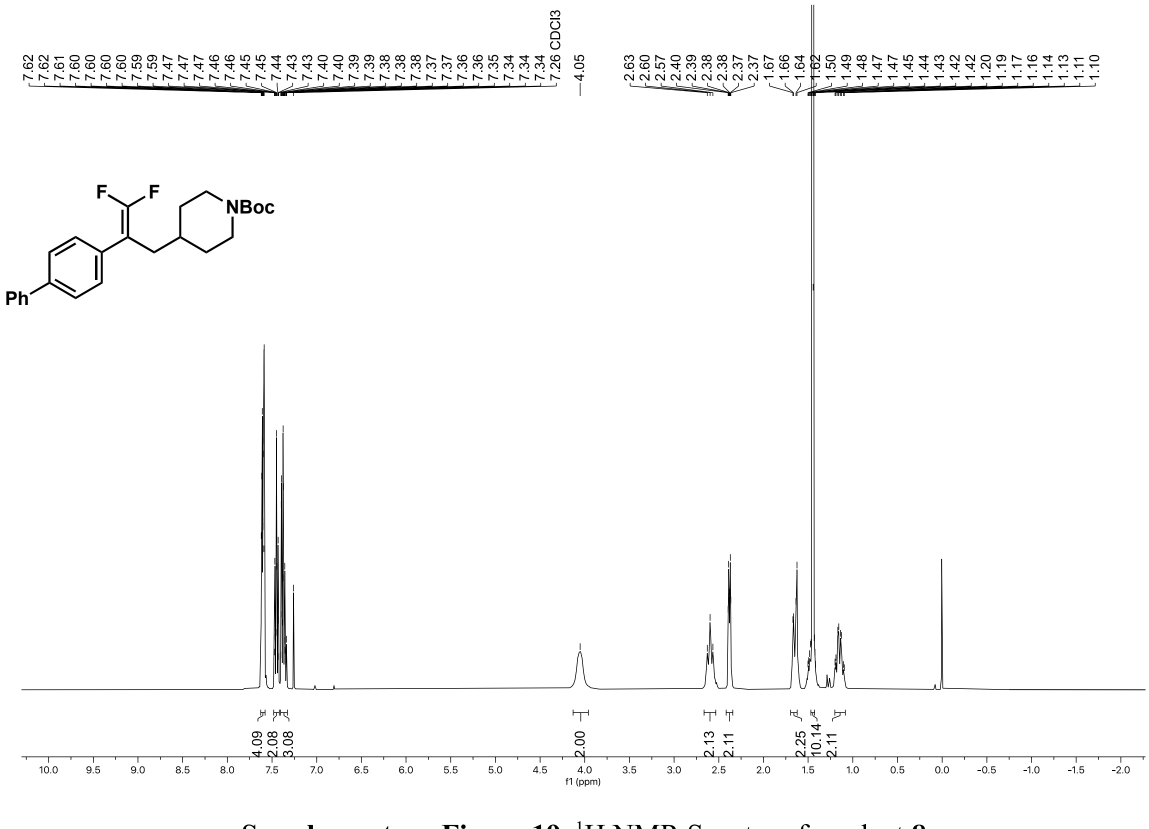

Supplementary Figure 19. ^{1}H NMR Spectra of product **8**

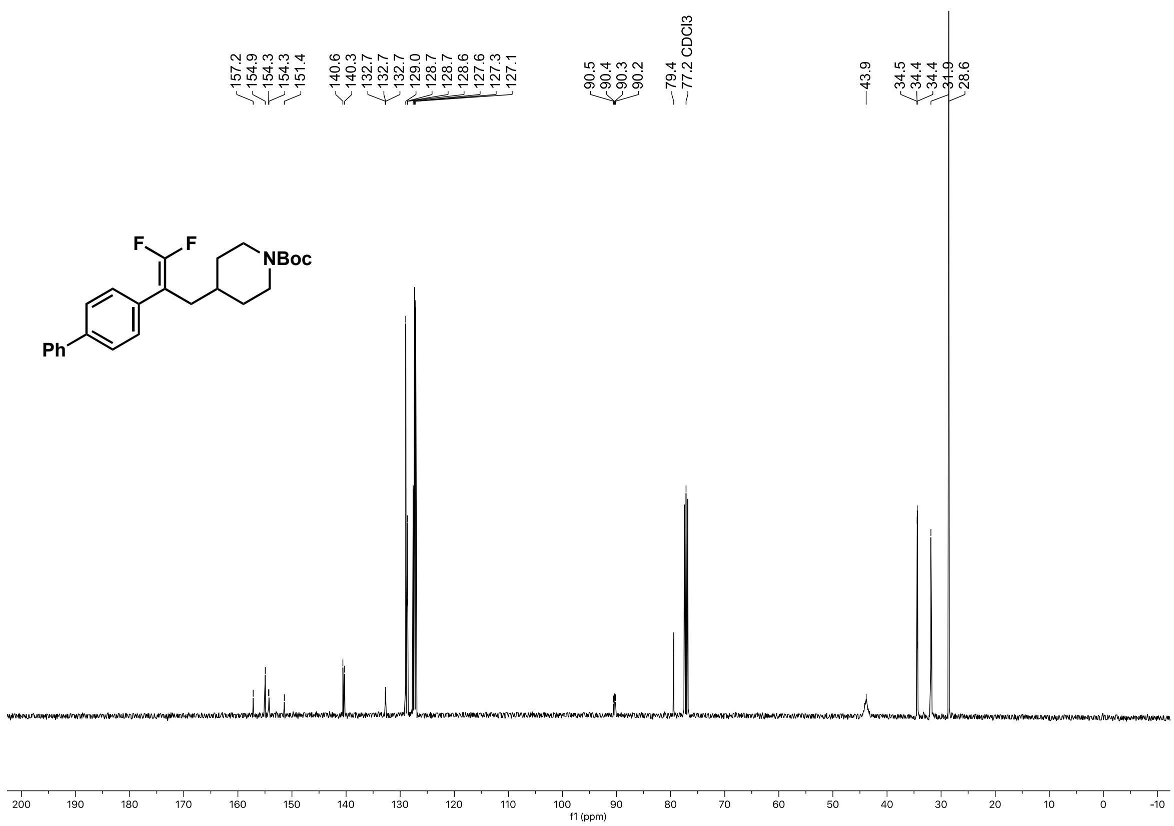

Supplementary Figure 20. ^{13}C NMR Spectra of product **8**

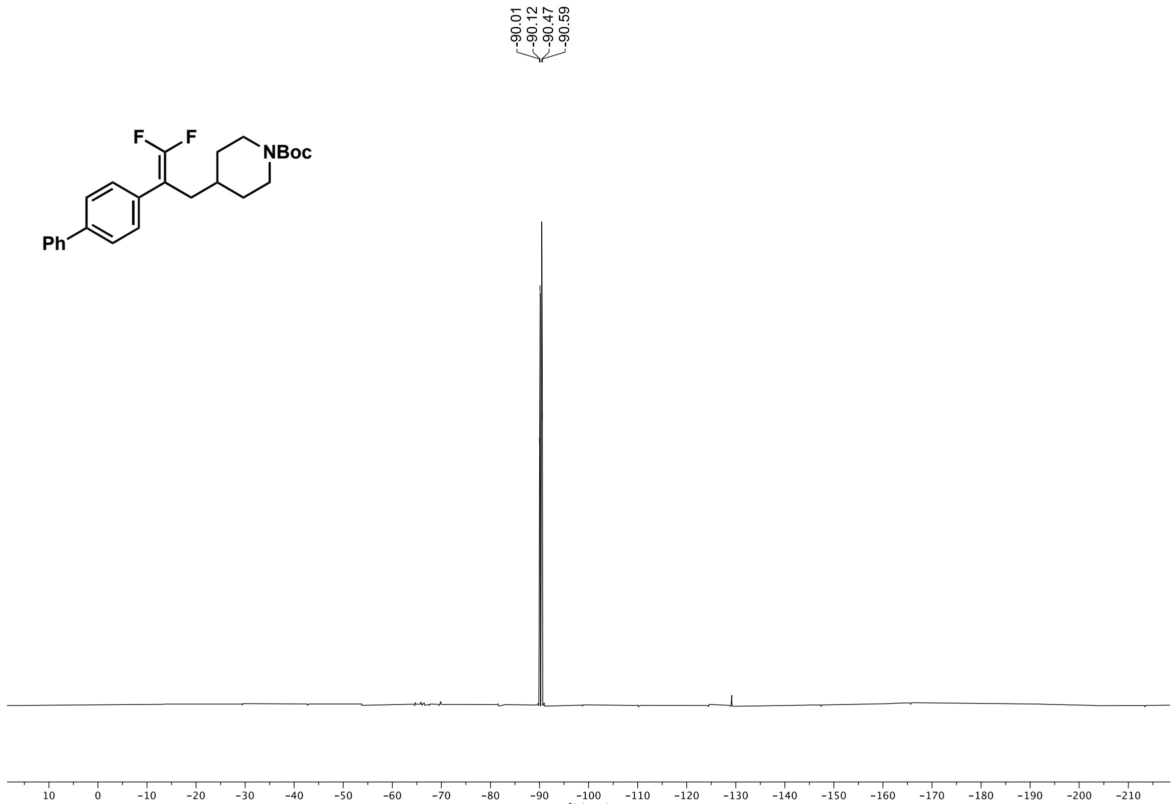

Supplementary Figure 21. ^{19}F NMR Spectra of product **8**

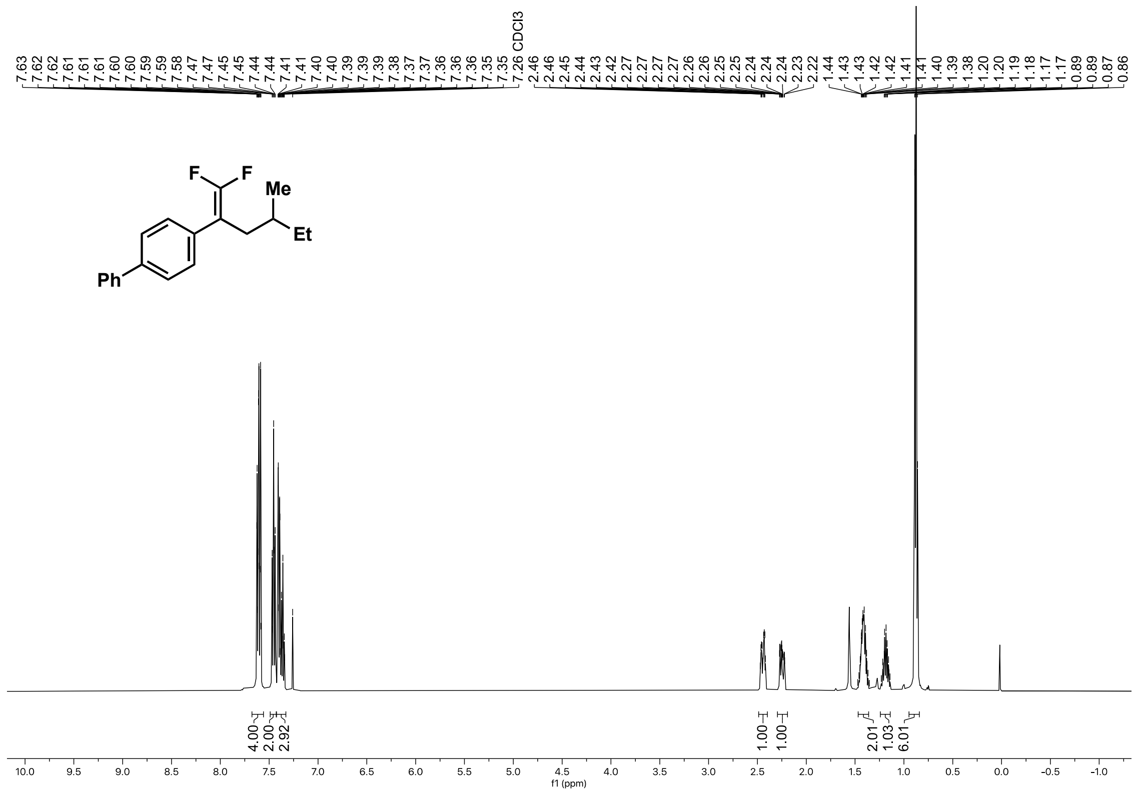

Supplementary Figure 22. ^{1}H NMR Spectra of product **9**

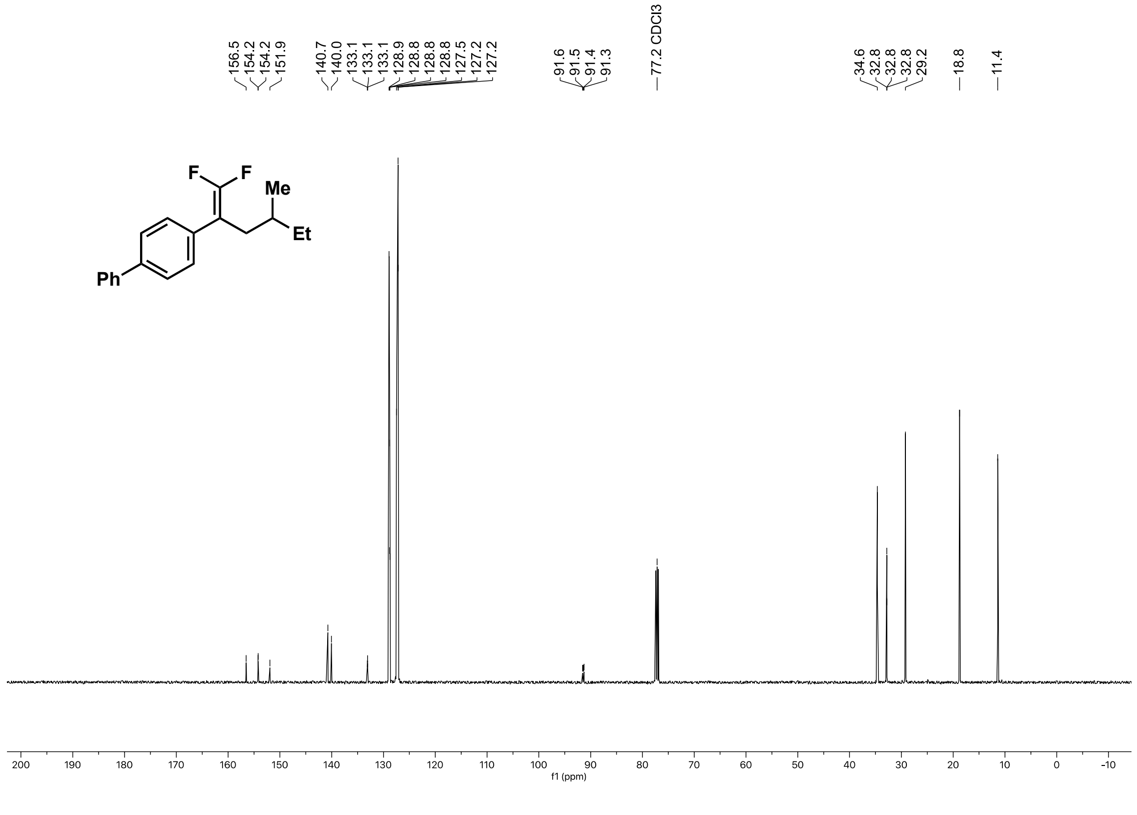

Supplementary Figure 23. ^{13}C NMR Spectra of product **9**

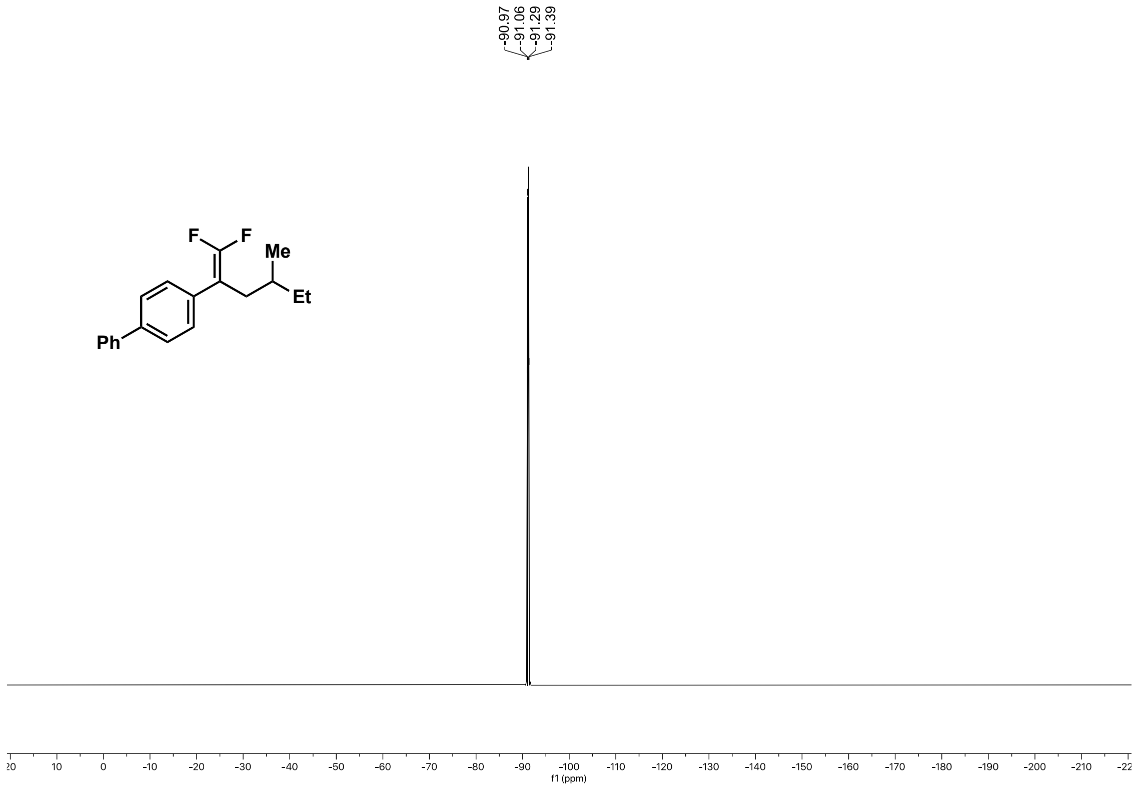

Supplementary Figure 24. ^{19}F NMR Spectra of product **9**

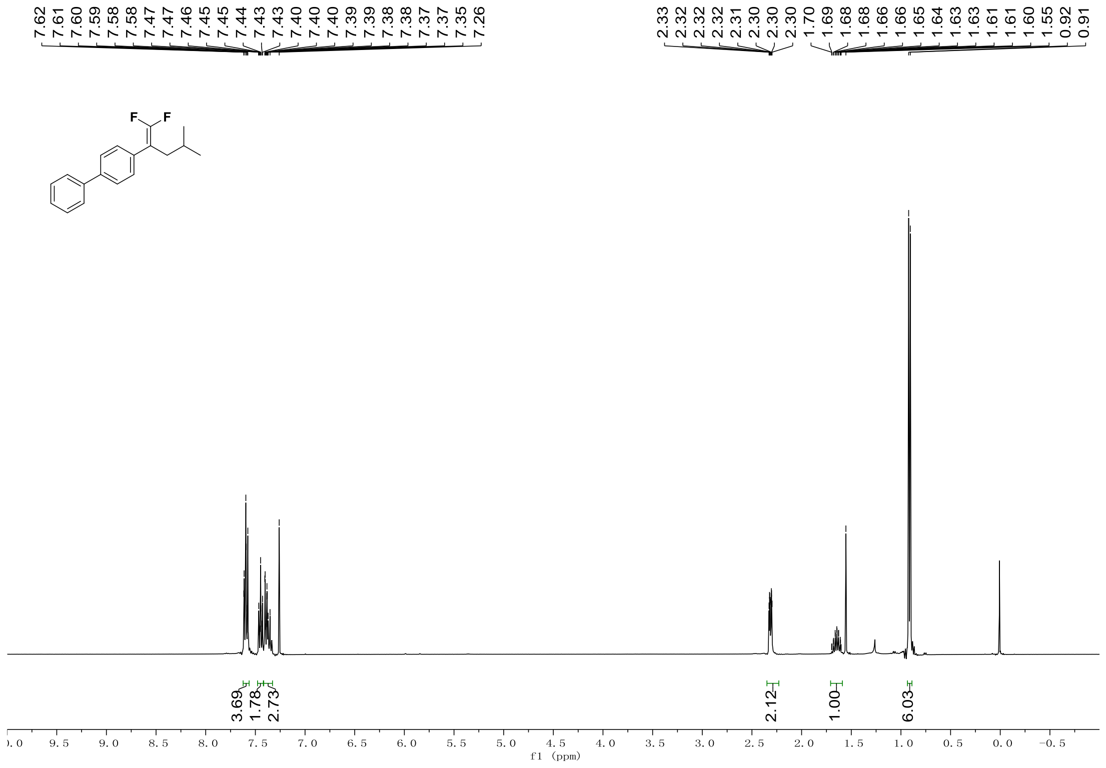

Supplementary Figure 25. ^{1}H NMR Spectra of product **10**

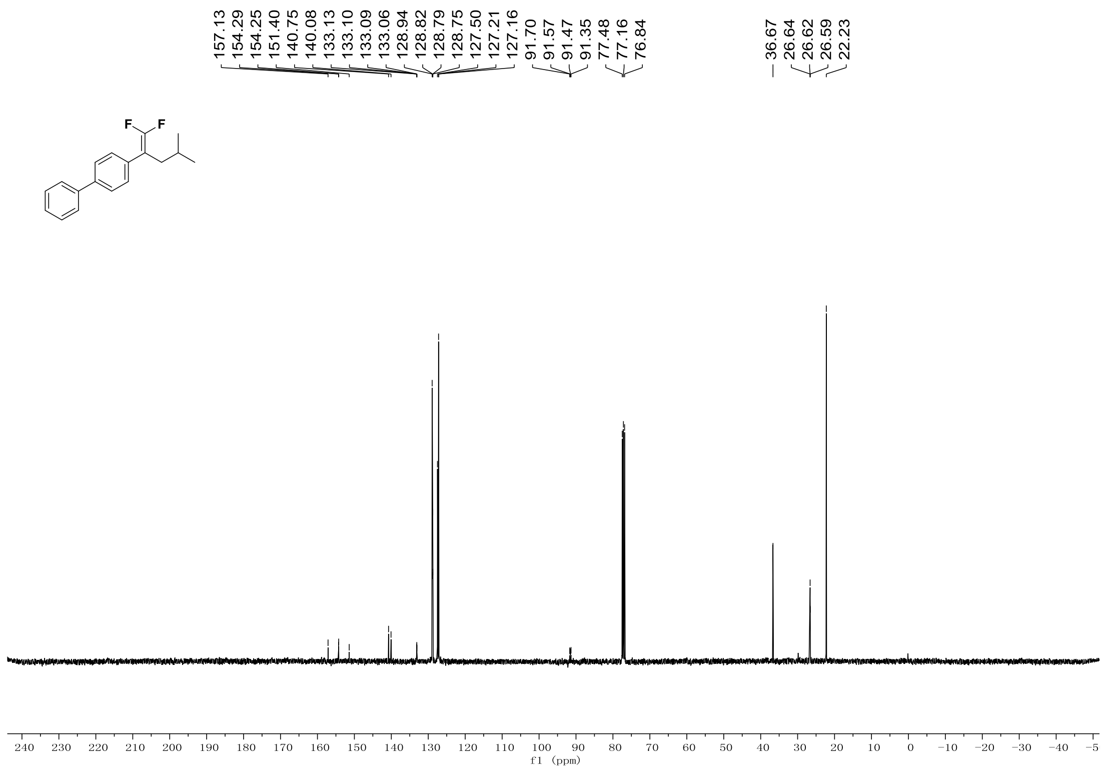

Supplementary Figure 26. ^{13}C NMR Spectra of product **10**

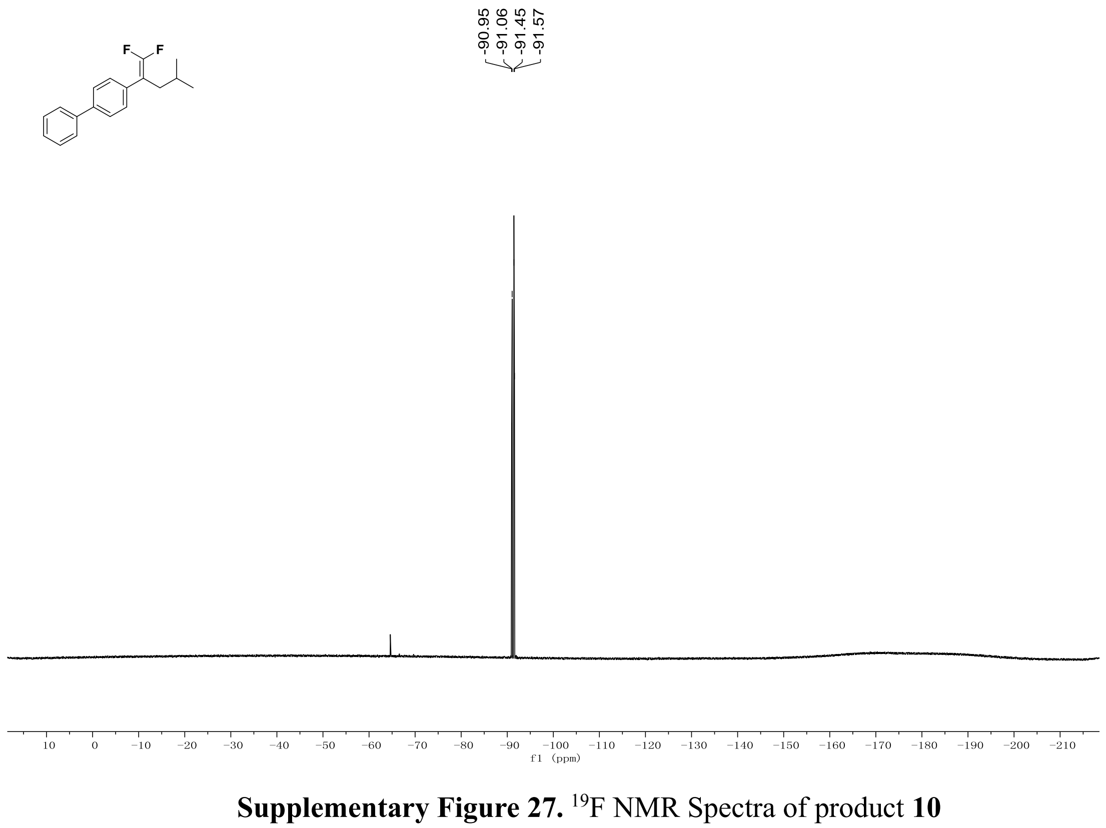

Supplementary Figure 27. ^{19}F NMR Spectra of product **10**

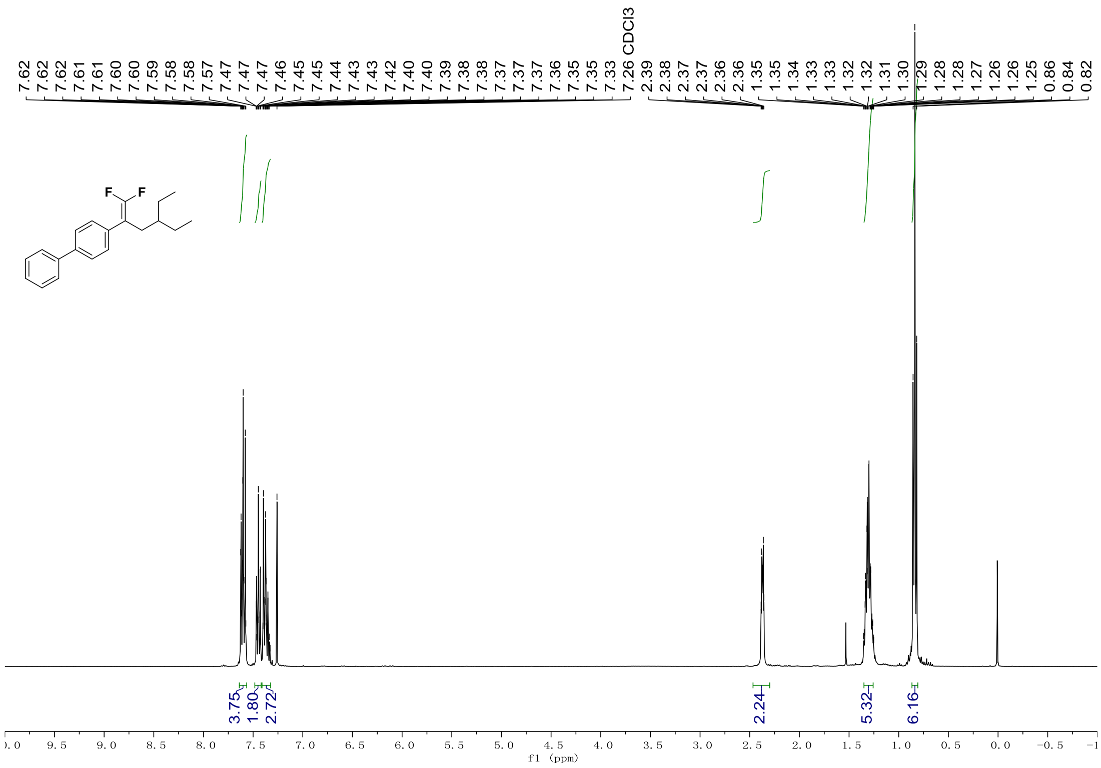

Supplementary Figure 28. ^{1}H NMR Spectra of product **11**

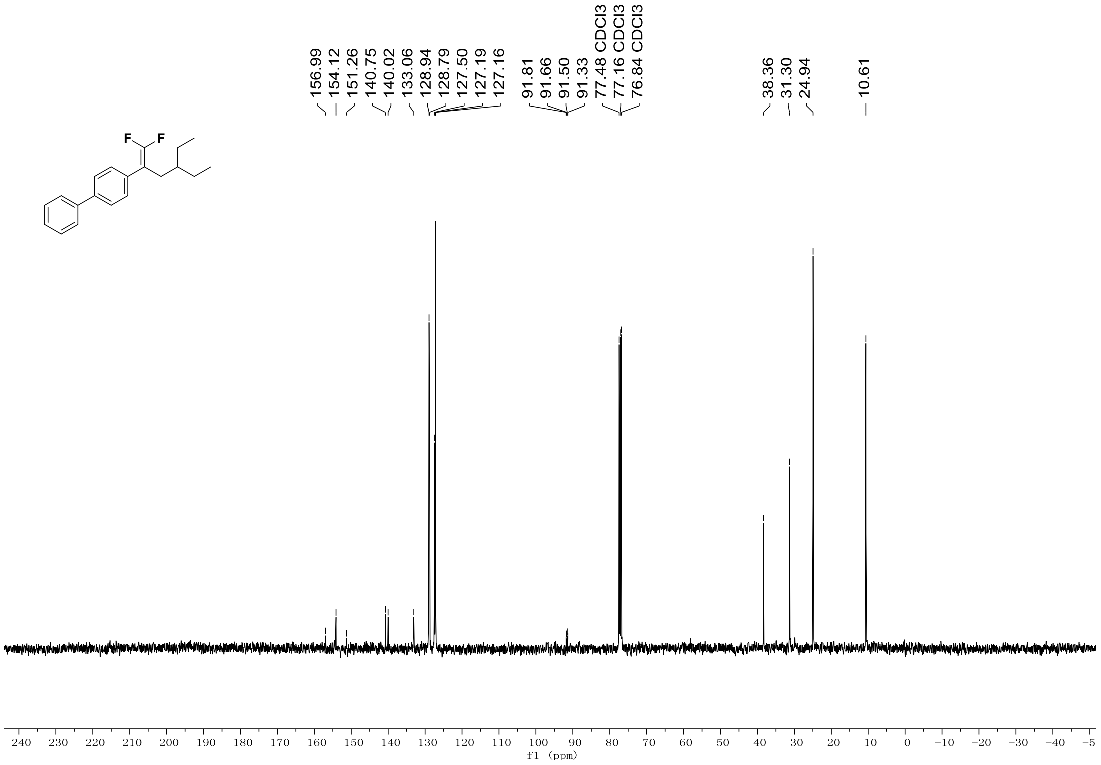

Supplementary Figure 29. ^{13}C NMR Spectra of product **11**

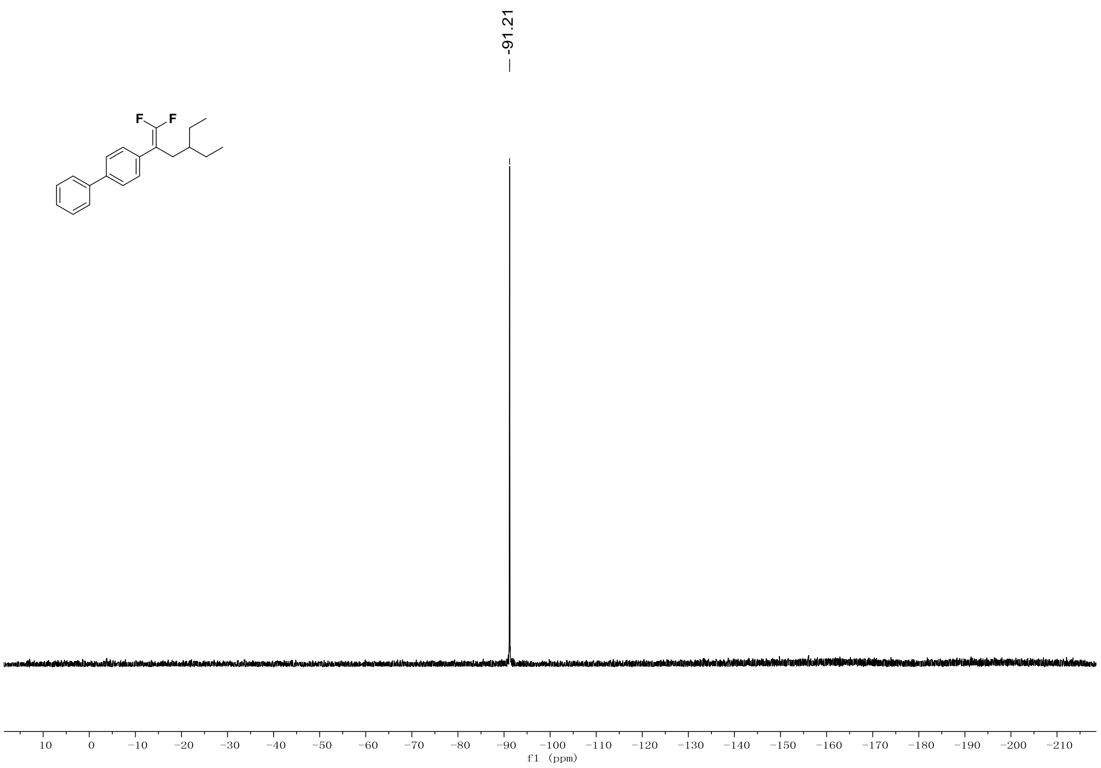

Supplementary Figure 30. ^{19}F NMR Spectra of product **11**

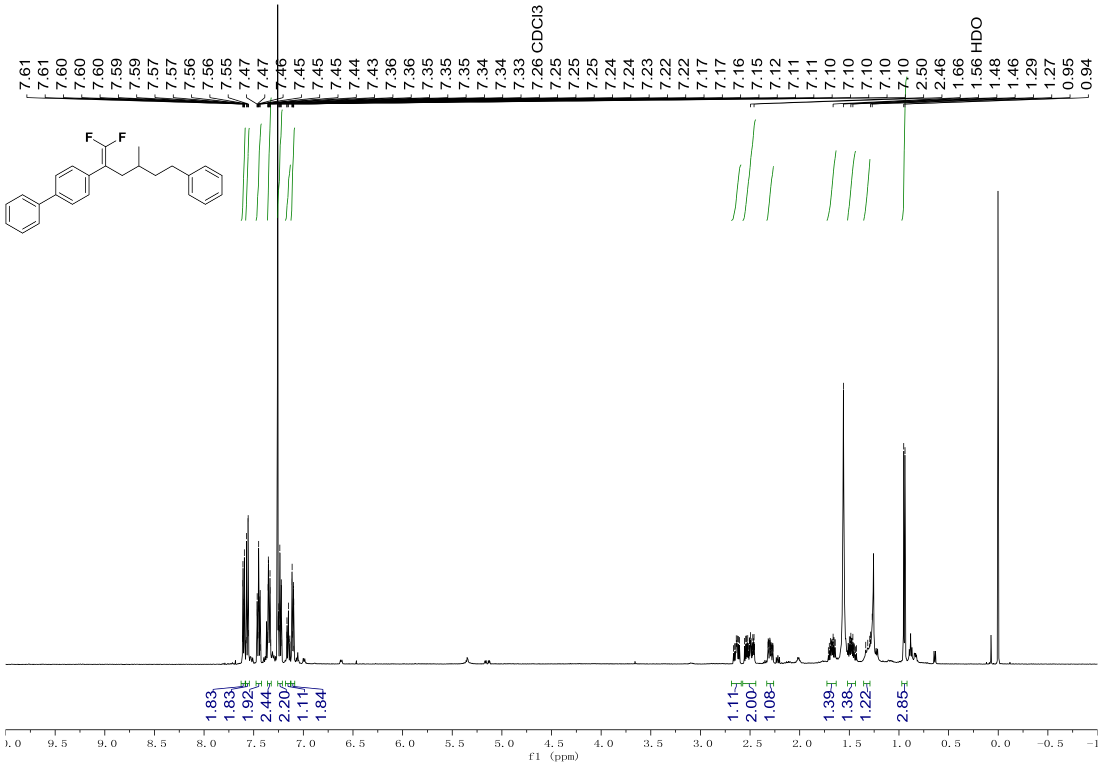

Supplementary Figure 31. ^{1}H NMR Spectra of product **12**

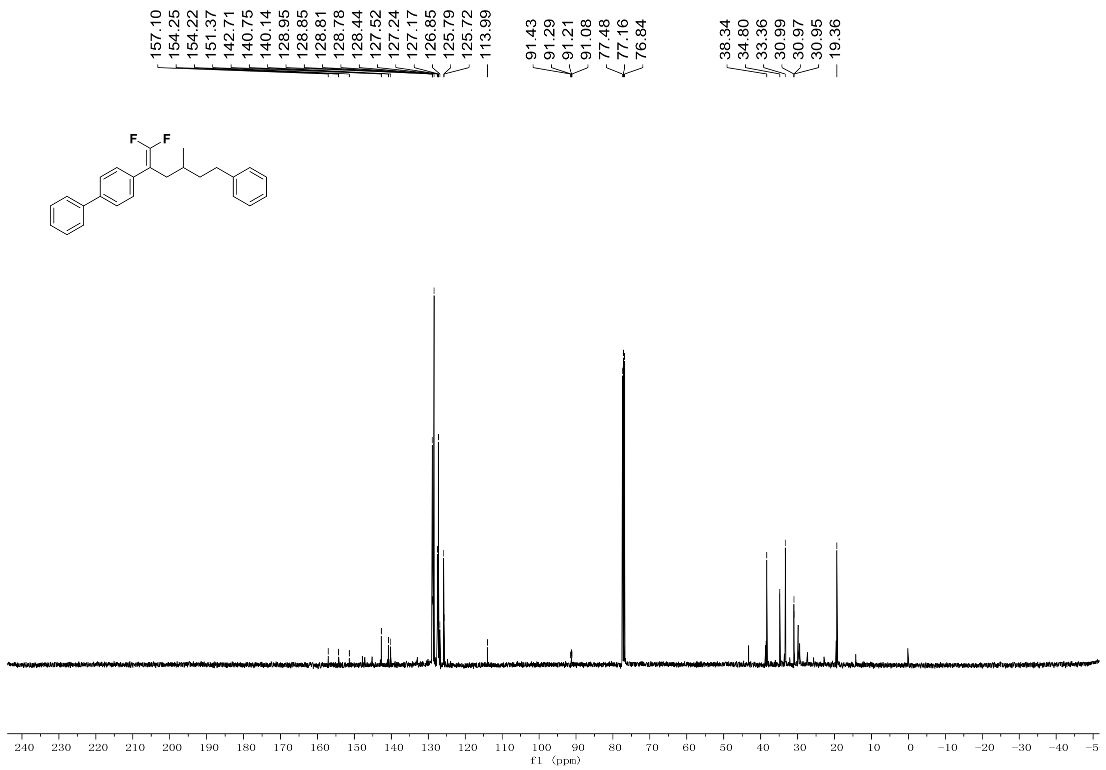

Supplementary Figure 32. ^{13}C NMR Spectra of product **12**

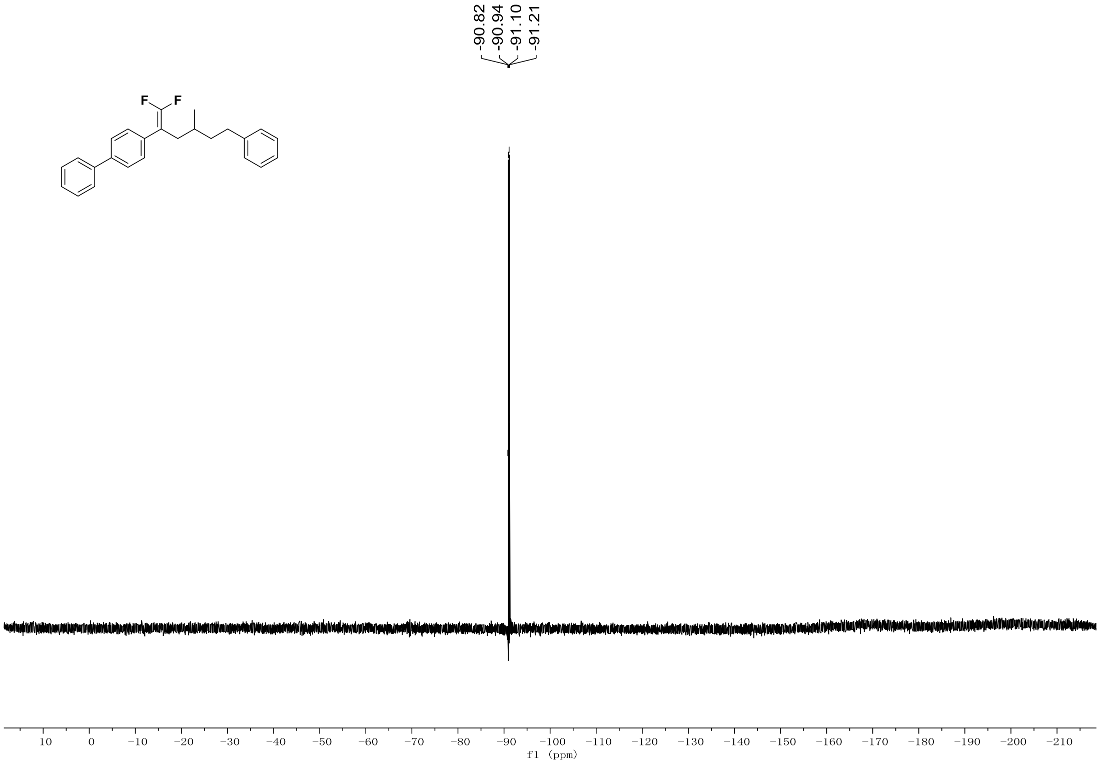

Supplementary Figure 33. ^{19}F NMR Spectra of product **12**

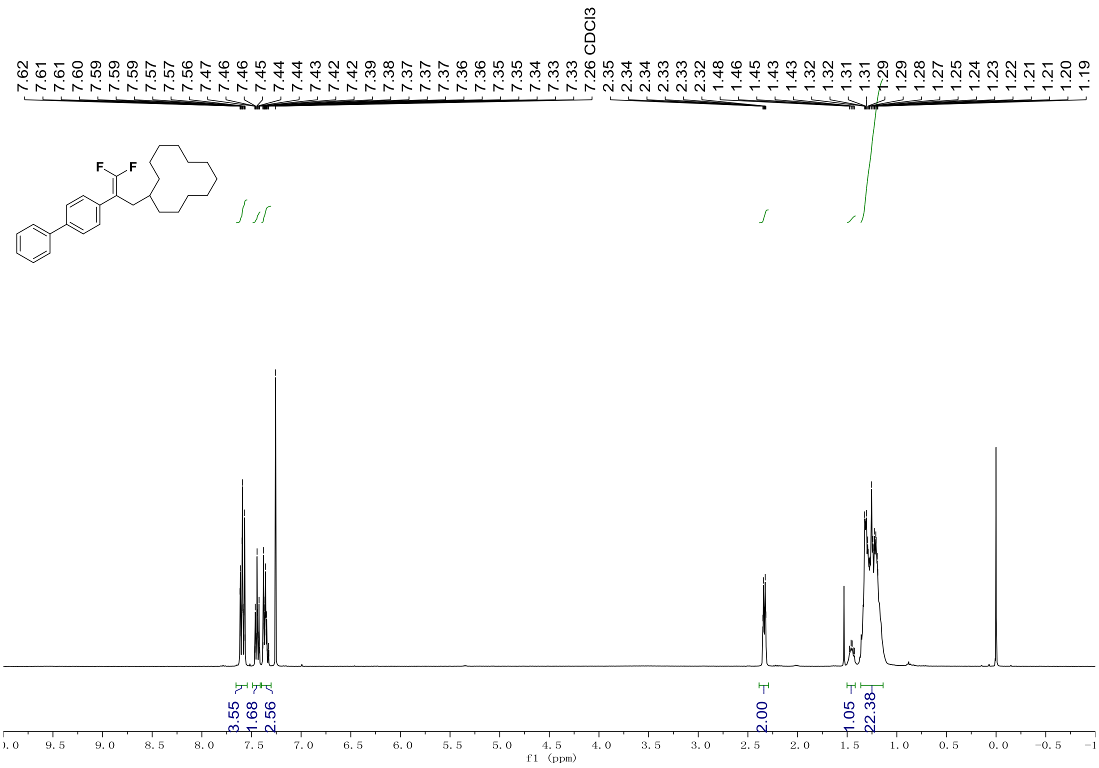

Supplementary Figure 34. ^{1}H NMR Spectra of product **13**

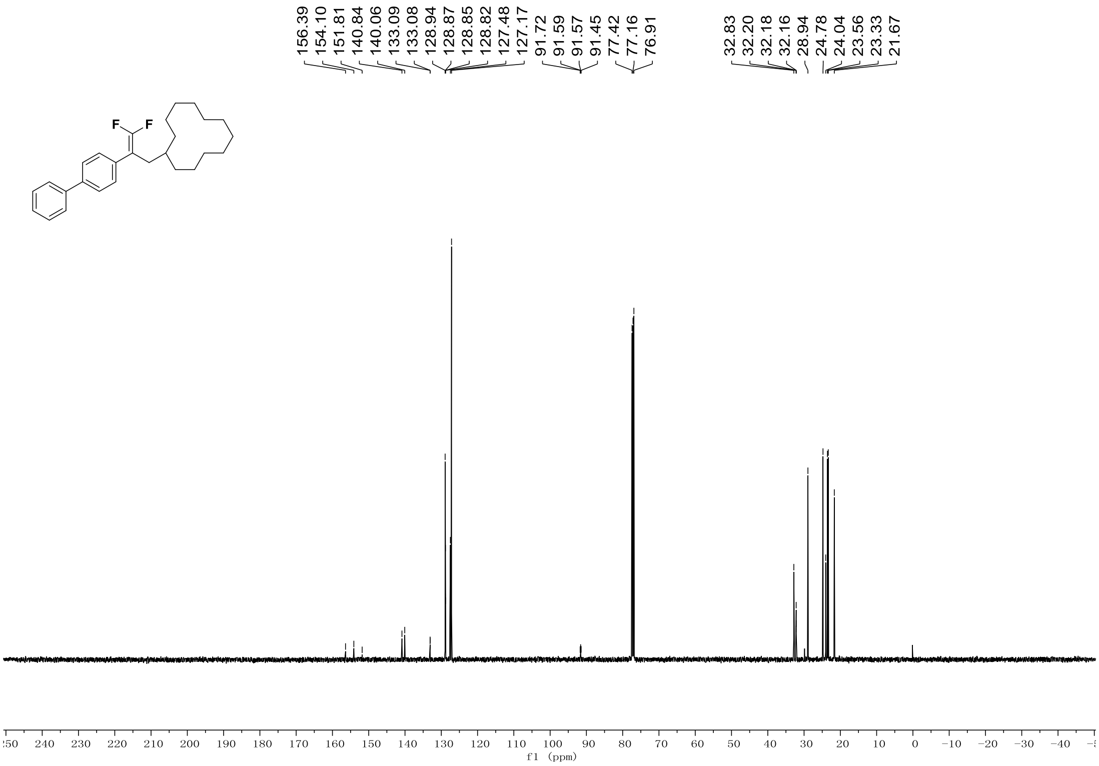

Supplementary Figure 35. ^{13}C NMR Spectra of product **13**

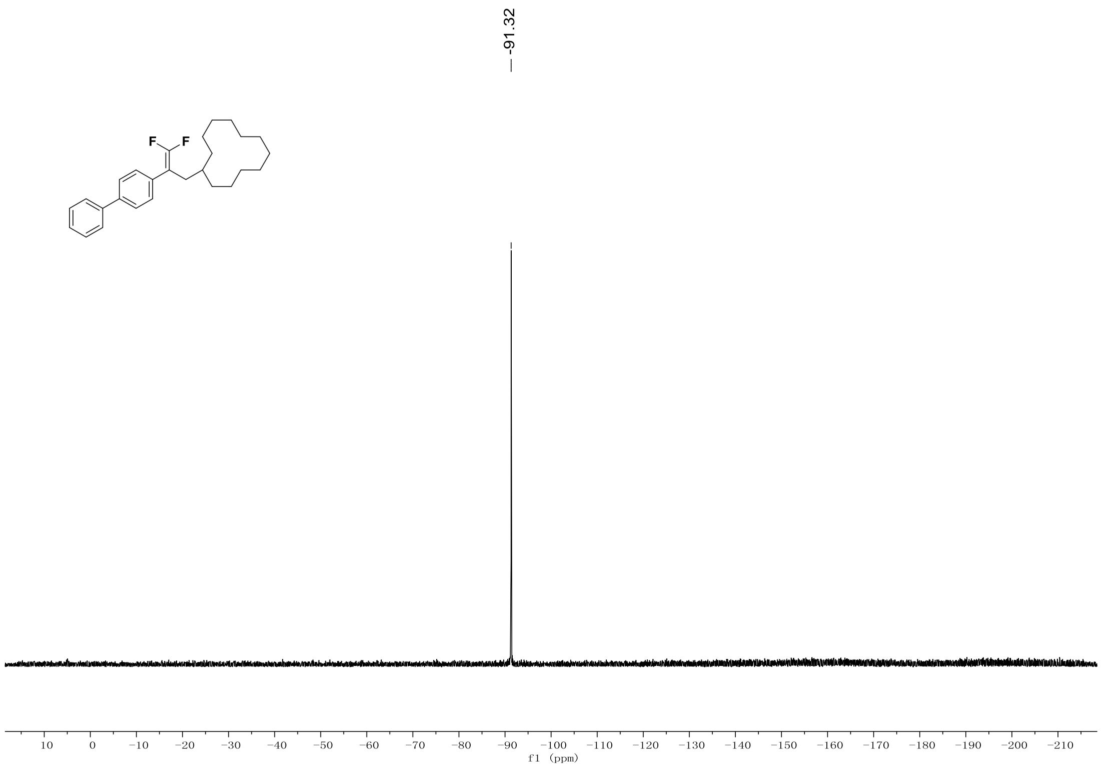

Supplementary Figure 36. ^{19}F NMR Spectra of product **13**

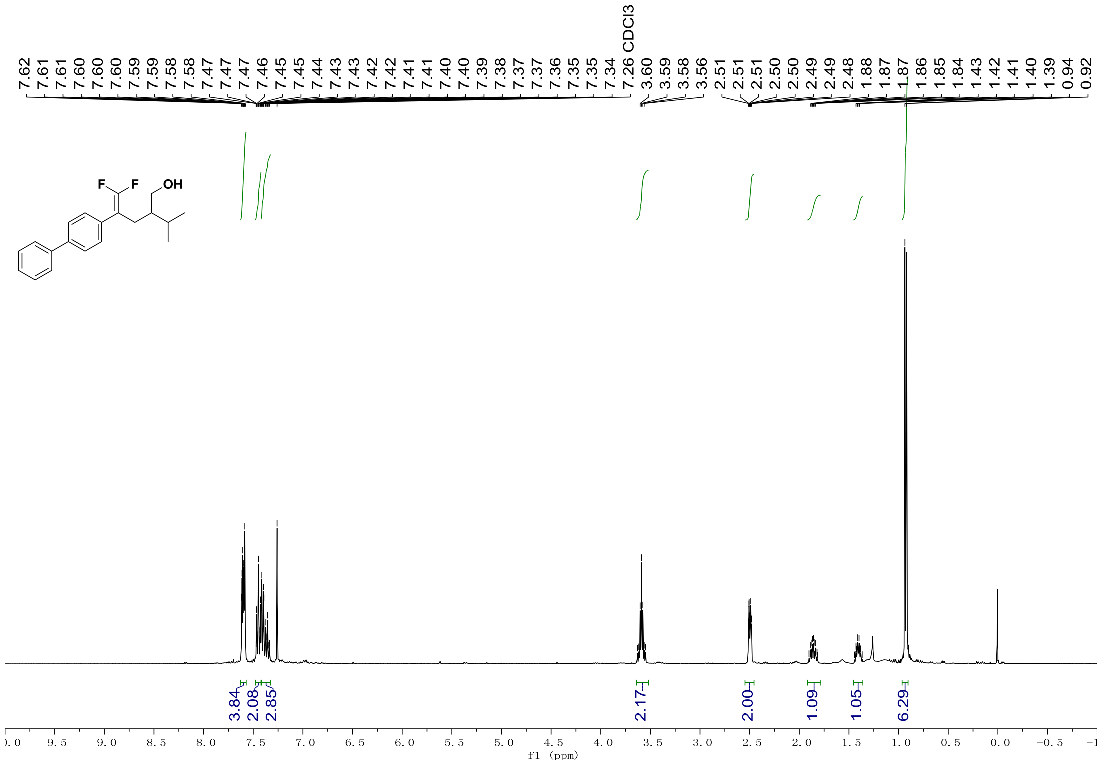

Supplementary Figure 37. ^{1}H NMR Spectra of product **14**

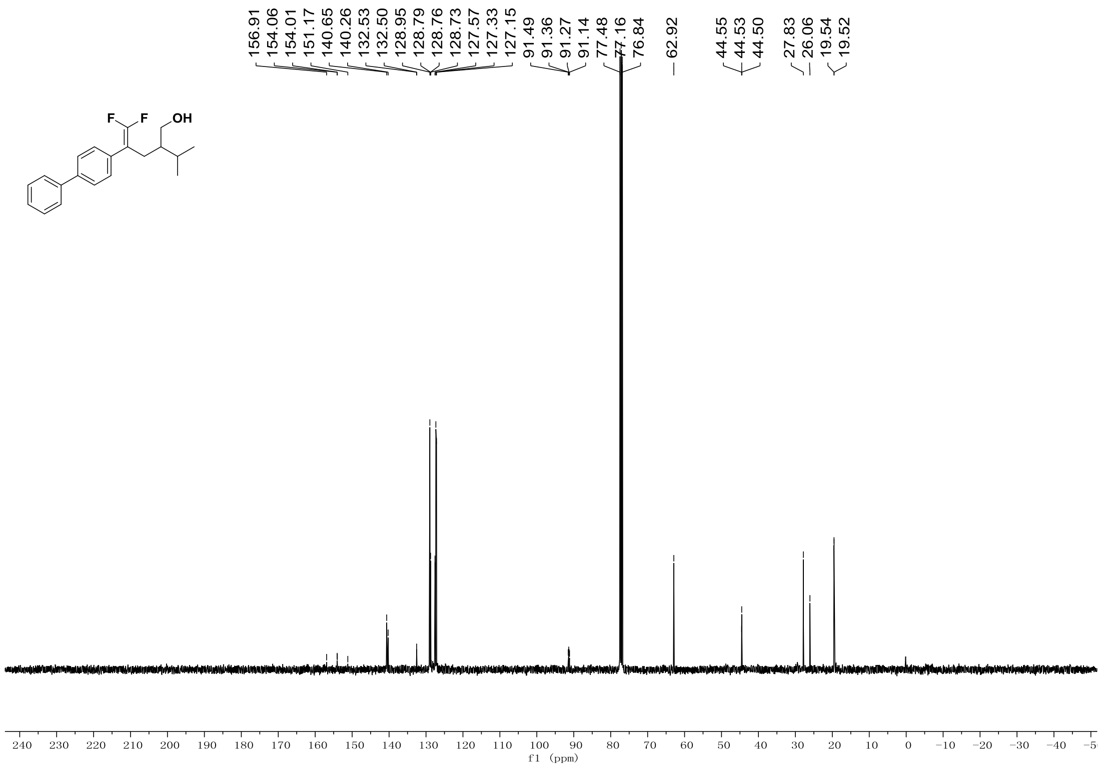

Supplementary Figure 38. ^{13}C NMR Spectra of product **14**

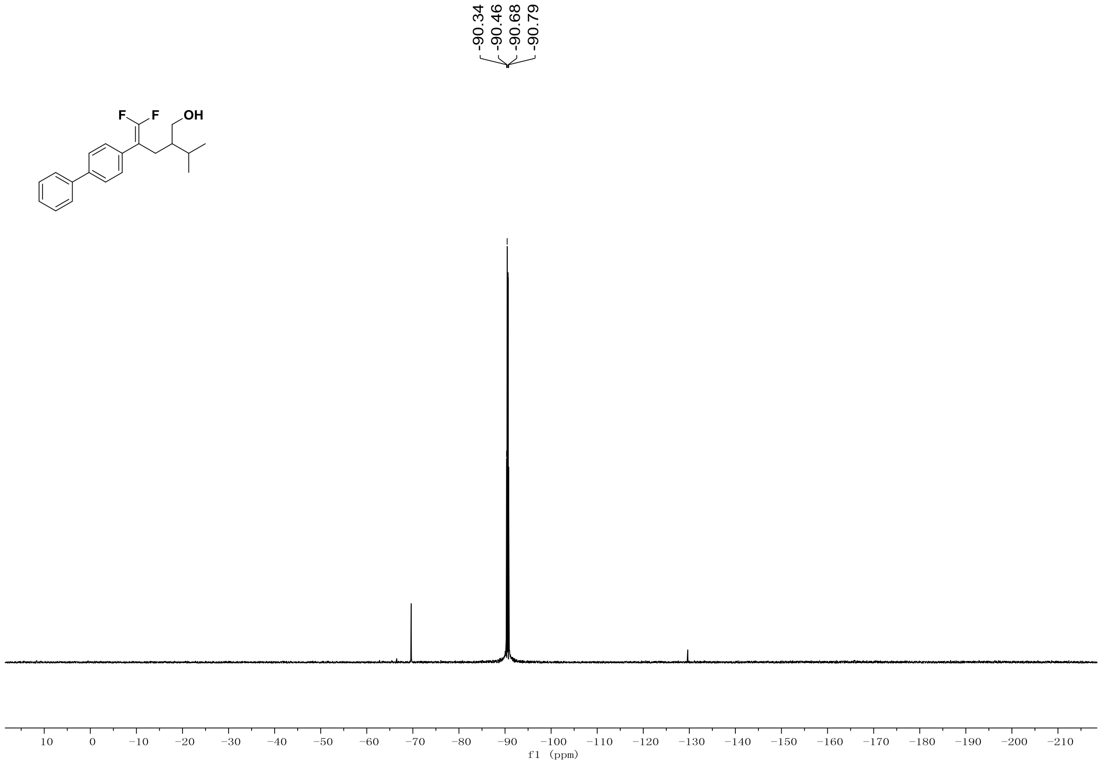

Supplementary Figure 39. ^{19}F NMR Spectra of product **14**

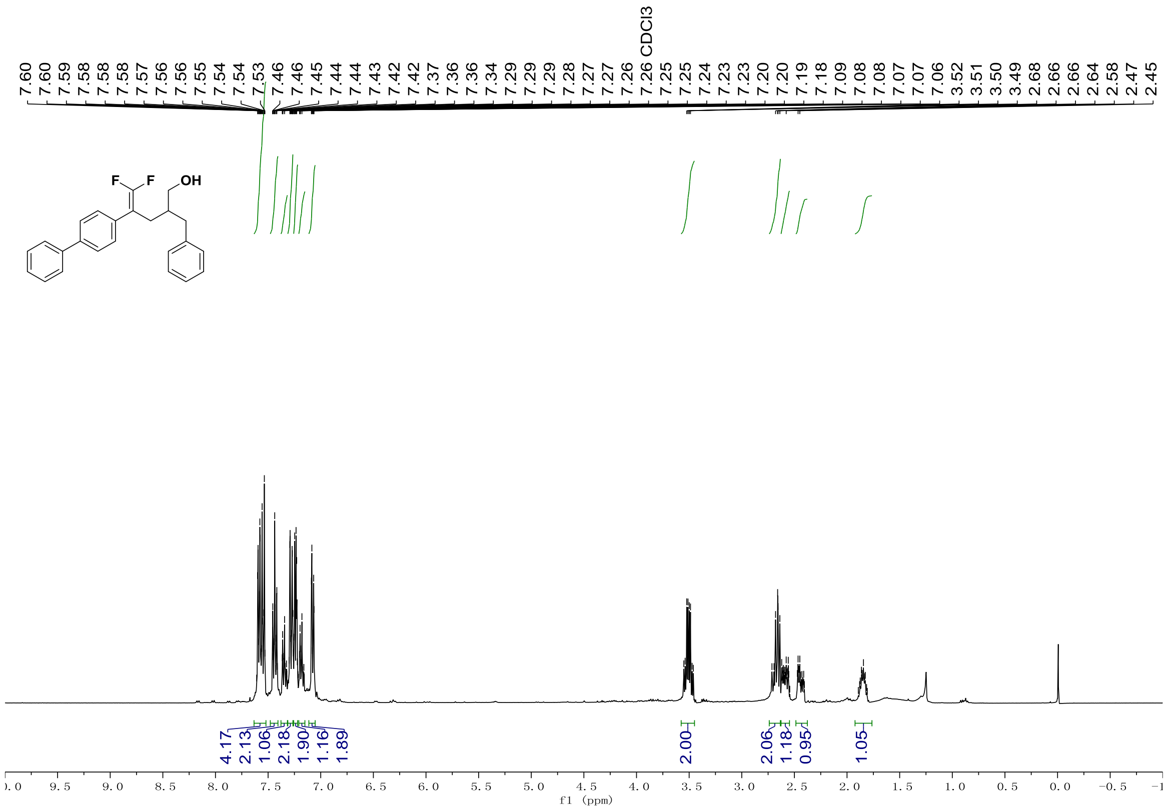

Supplementary Figure 40. ^{1}H NMR Spectra of product **15**

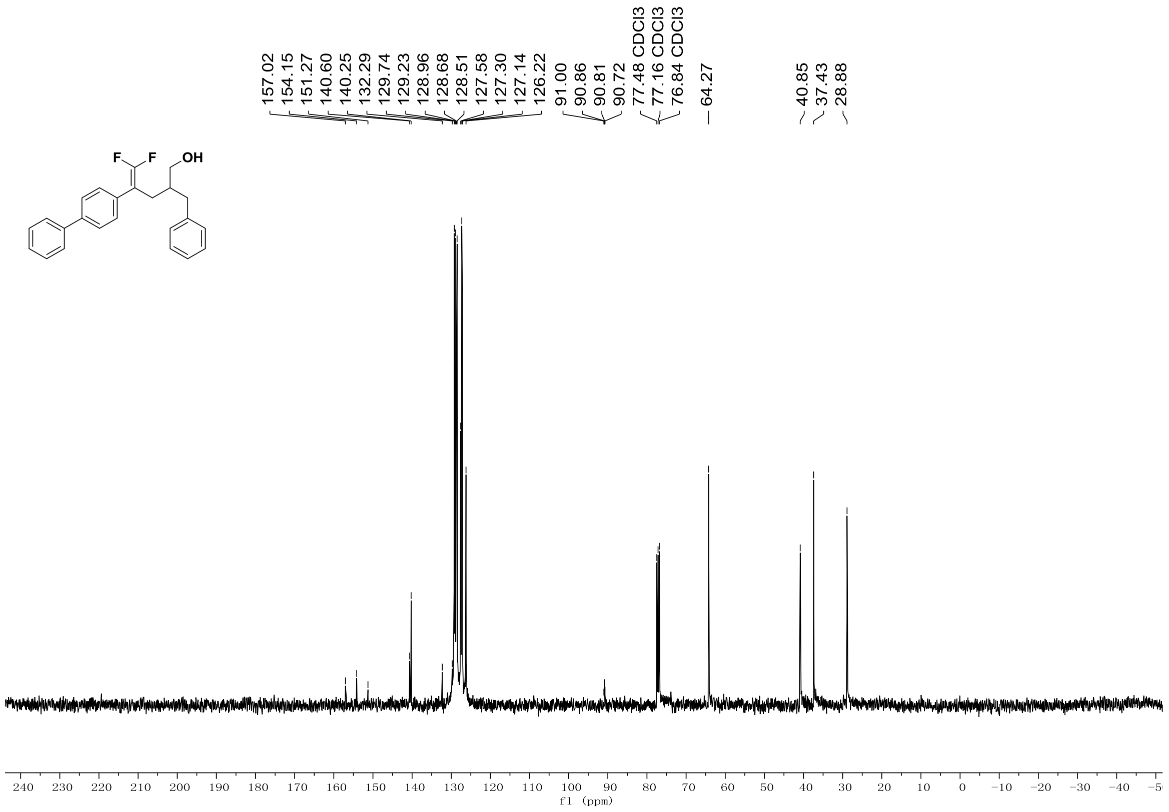

Supplementary Figure 41. ^{13}C NMR Spectra of product **15**

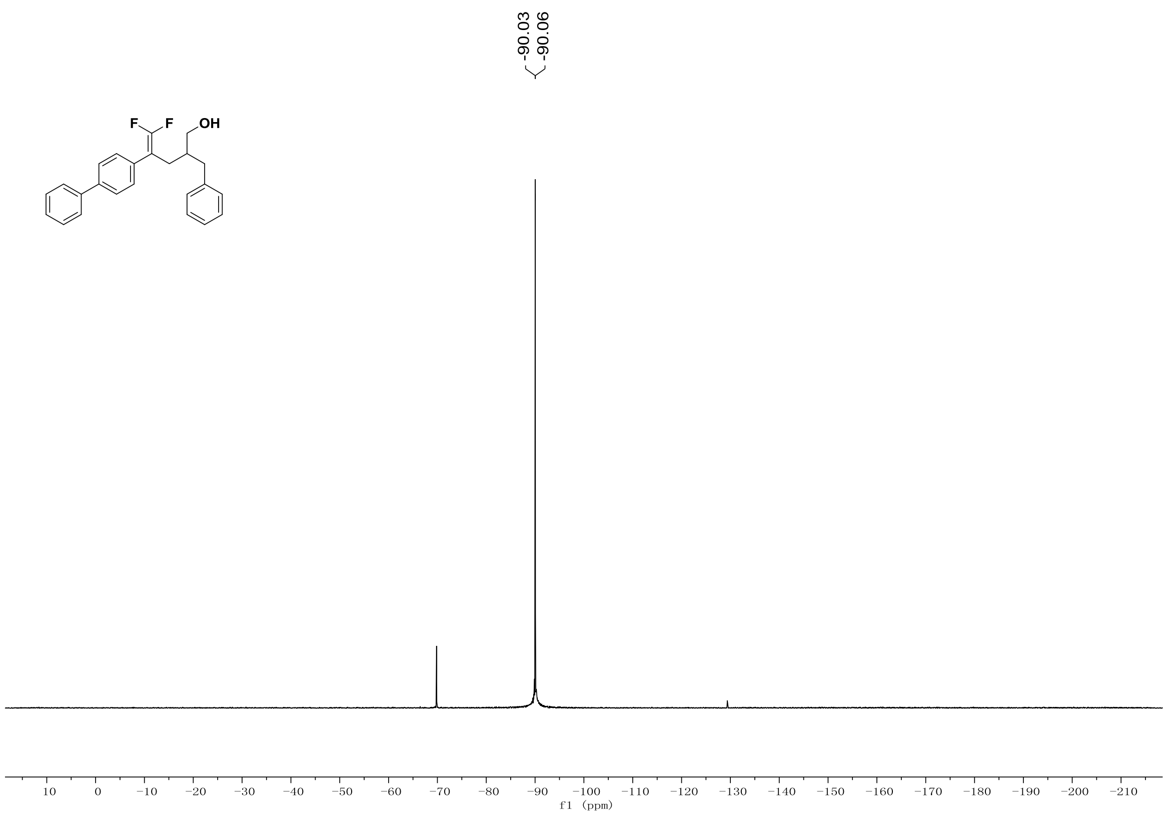

Supplementary Figure 42. ^{19}F NMR Spectra of product **15**

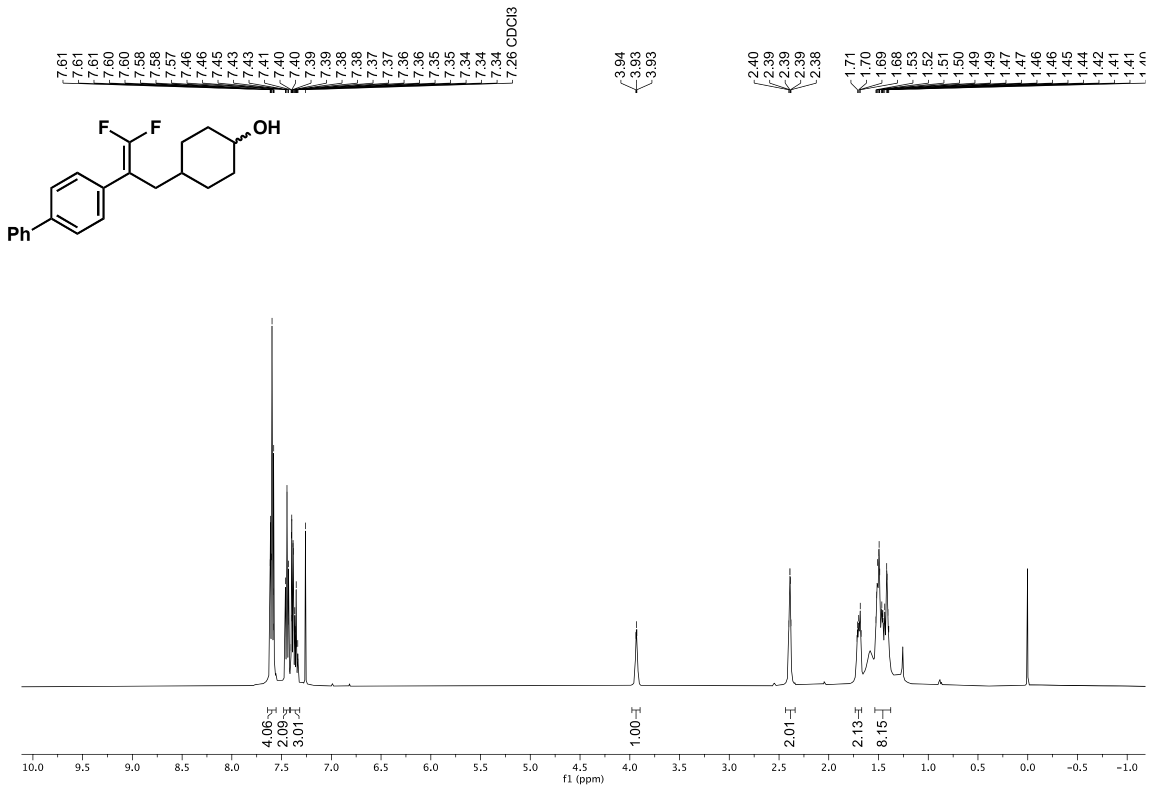

Supplementary Figure 43. ^{1}H NMR Spectra of product **16-1**

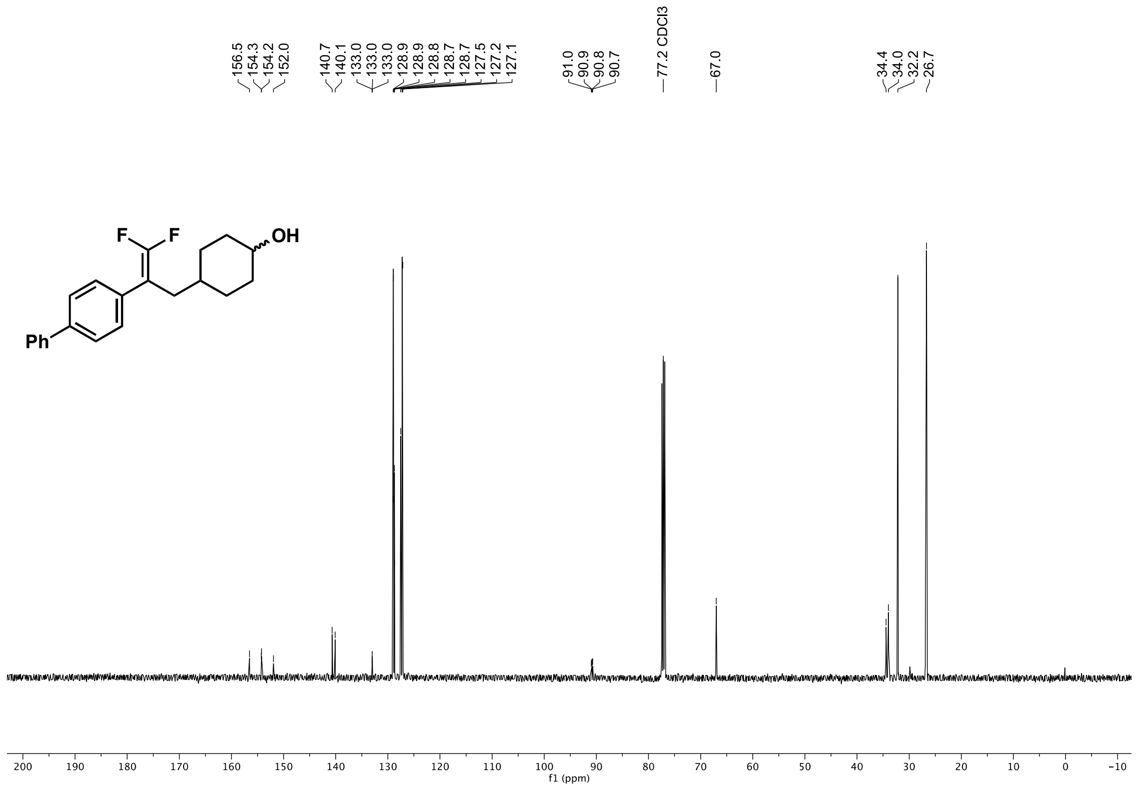

Supplementary Figure 44. ^{13}C NMR Spectra of product **16-1**

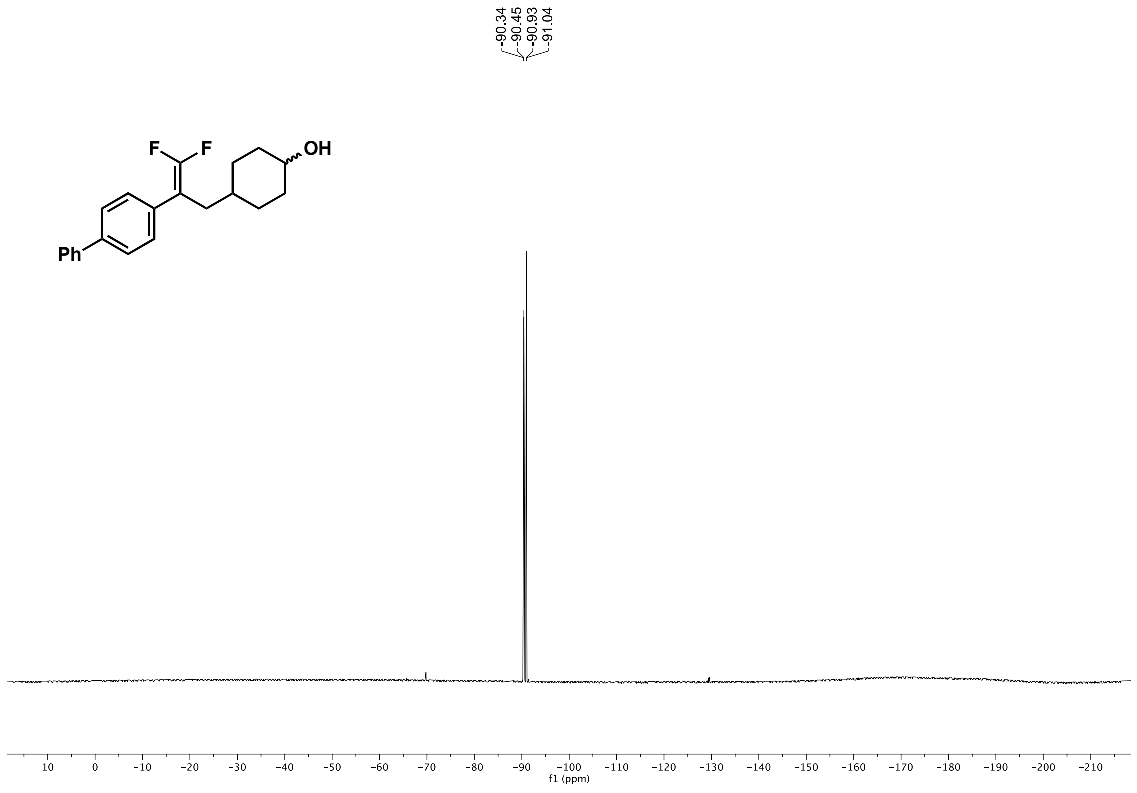

Supplementary Figure 45. ^{19}F NMR Spectra of product **16-1**

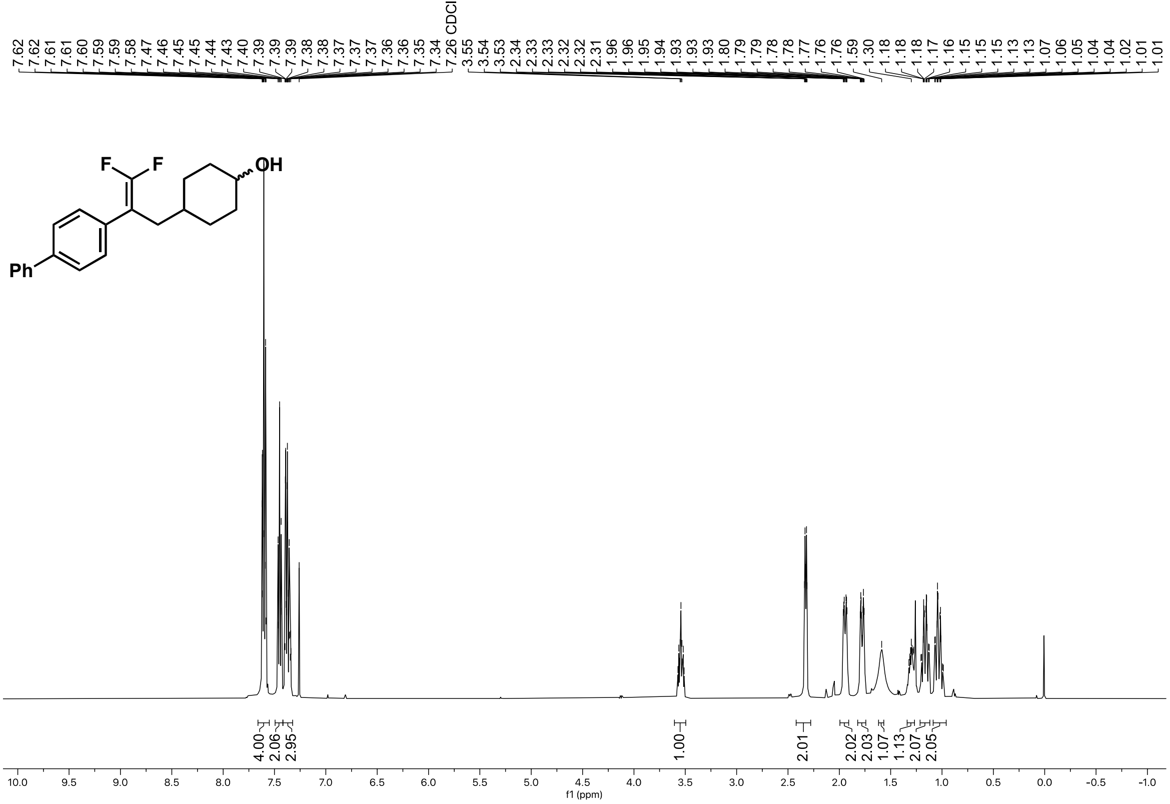

Supplementary Figure 46. ^{1}H NMR Spectra of product **16-2**

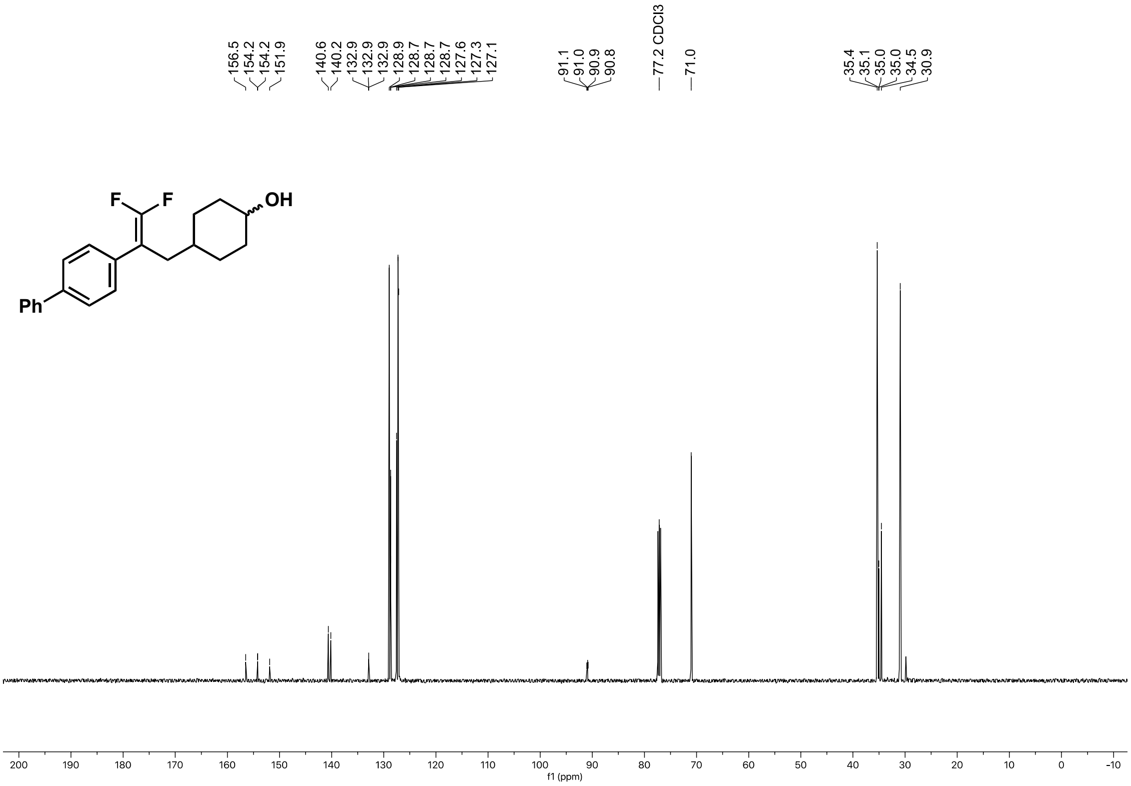

Supplementary Figure 47. ^{13}C NMR Spectra of product **16-2**

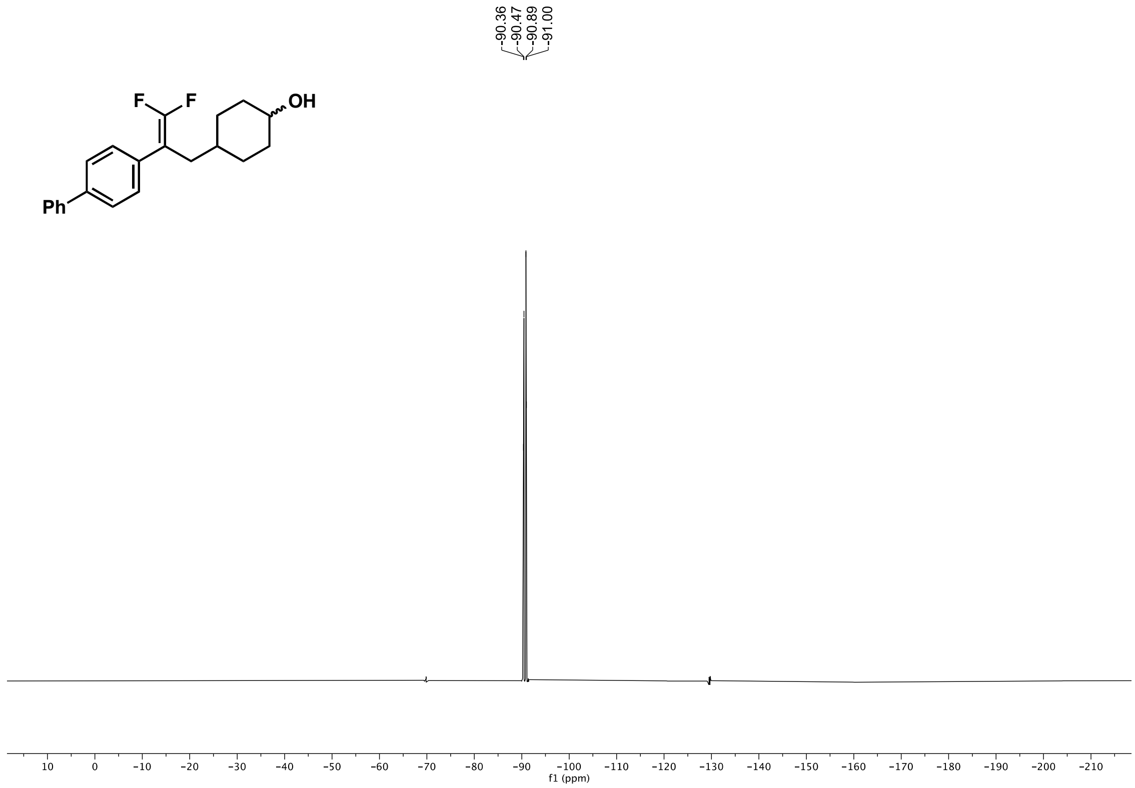

Supplementary Figure 48. ^{19}F NMR Spectra of product **16-2**

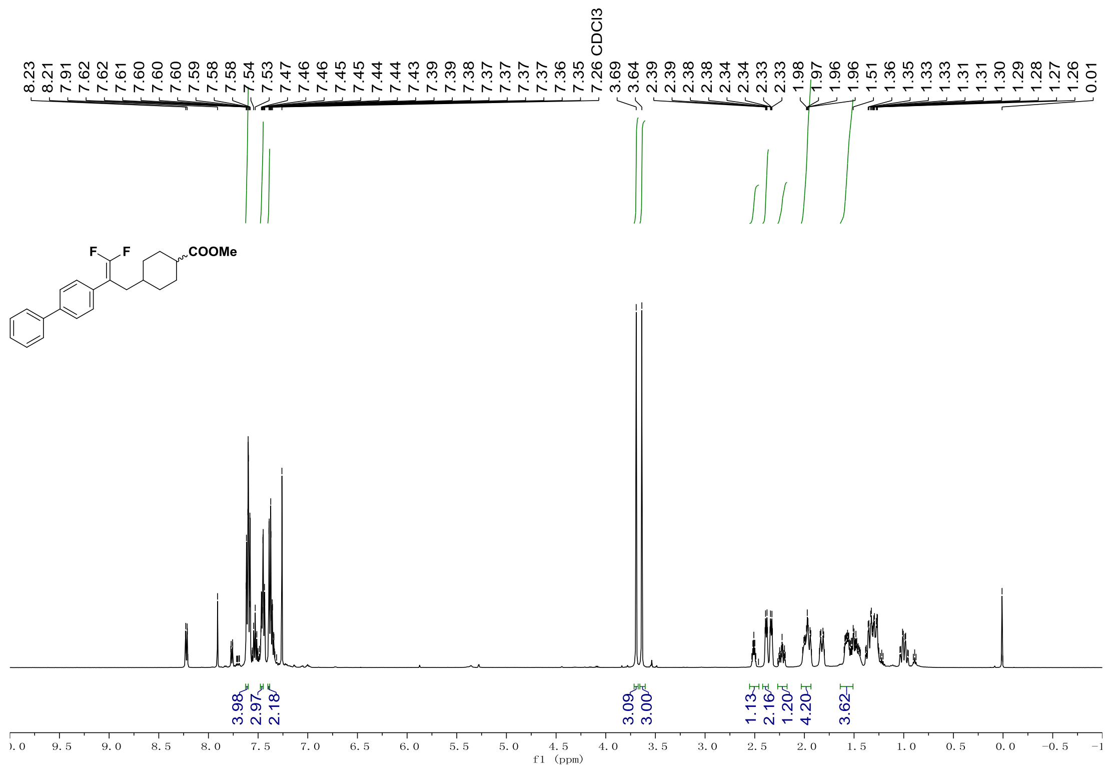

Supplementary Figure 49. ^{1}H NMR Spectra of product **17**

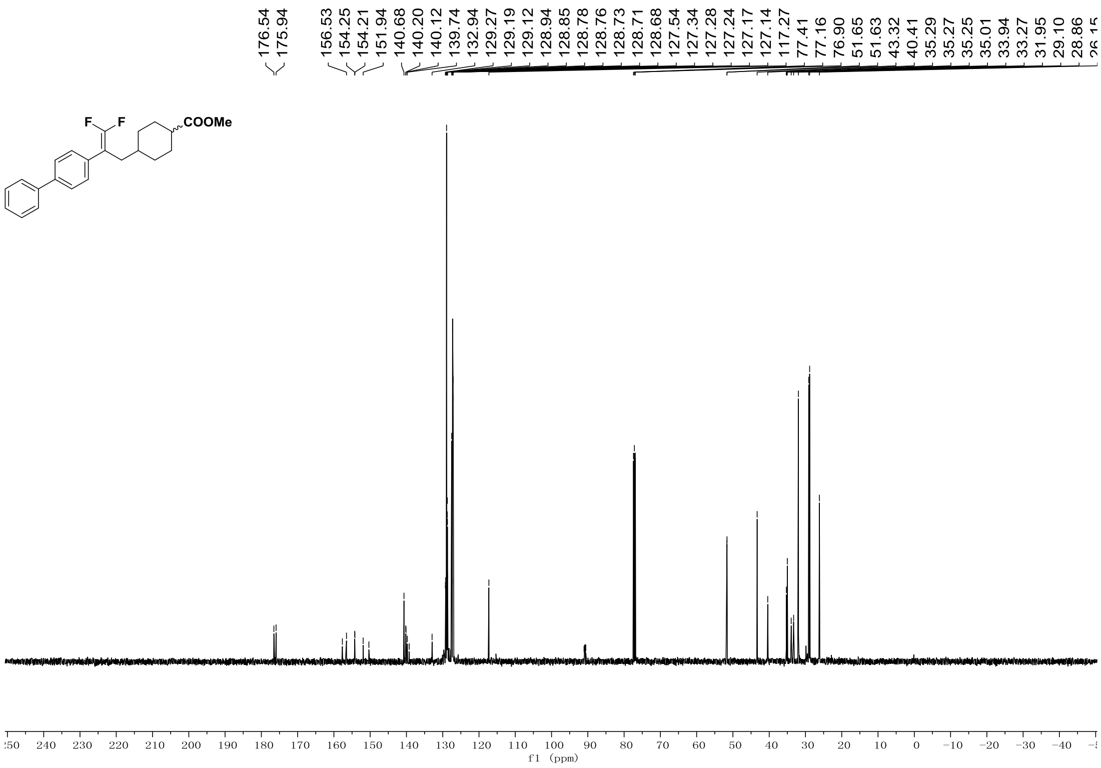

Supplementary Figure 50. ^{13}C NMR Spectra of product **17**

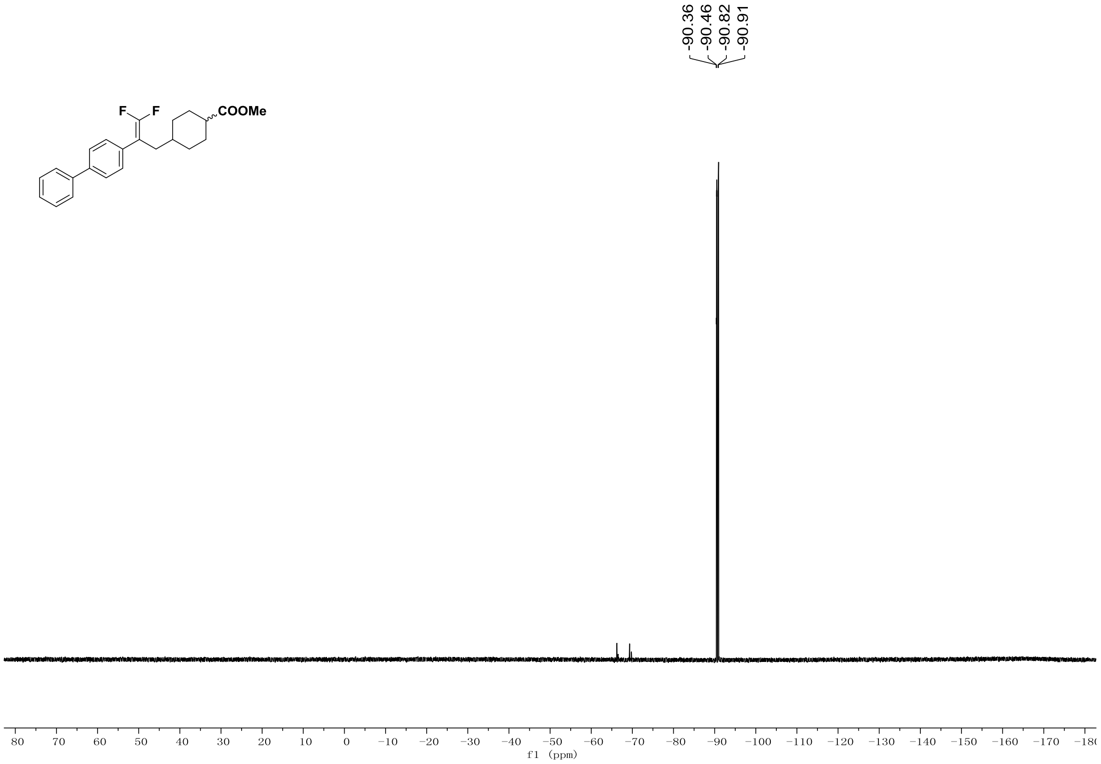

Supplementary Figure 51. ^{19}F NMR Spectra of product **17**

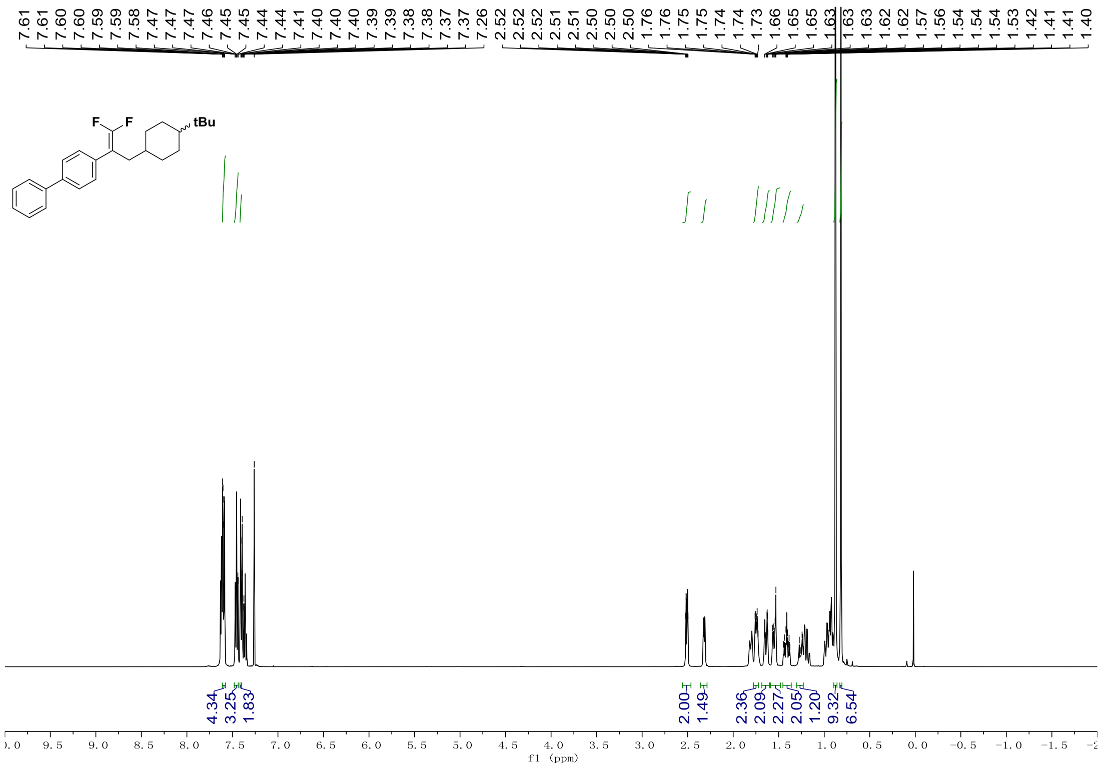

Supplementary Figure 52. ^{1}H NMR Spectra of product **18**

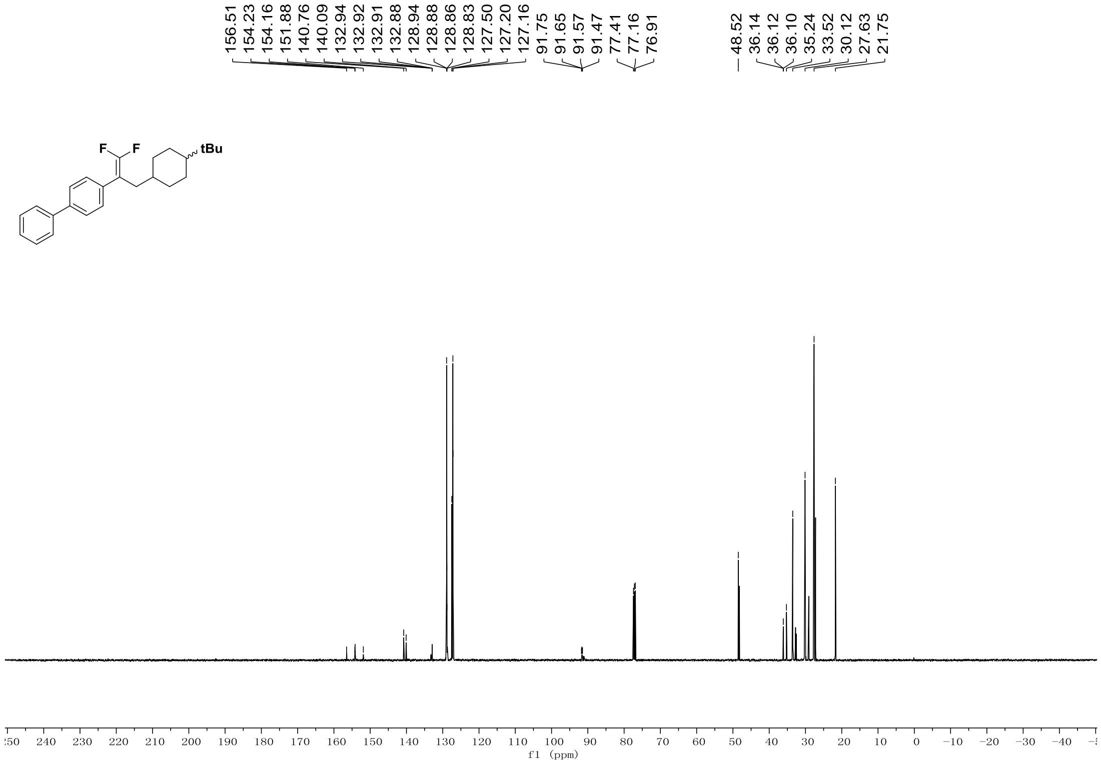

Supplementary Figure 53. ^{13}C NMR Spectra of product **18**

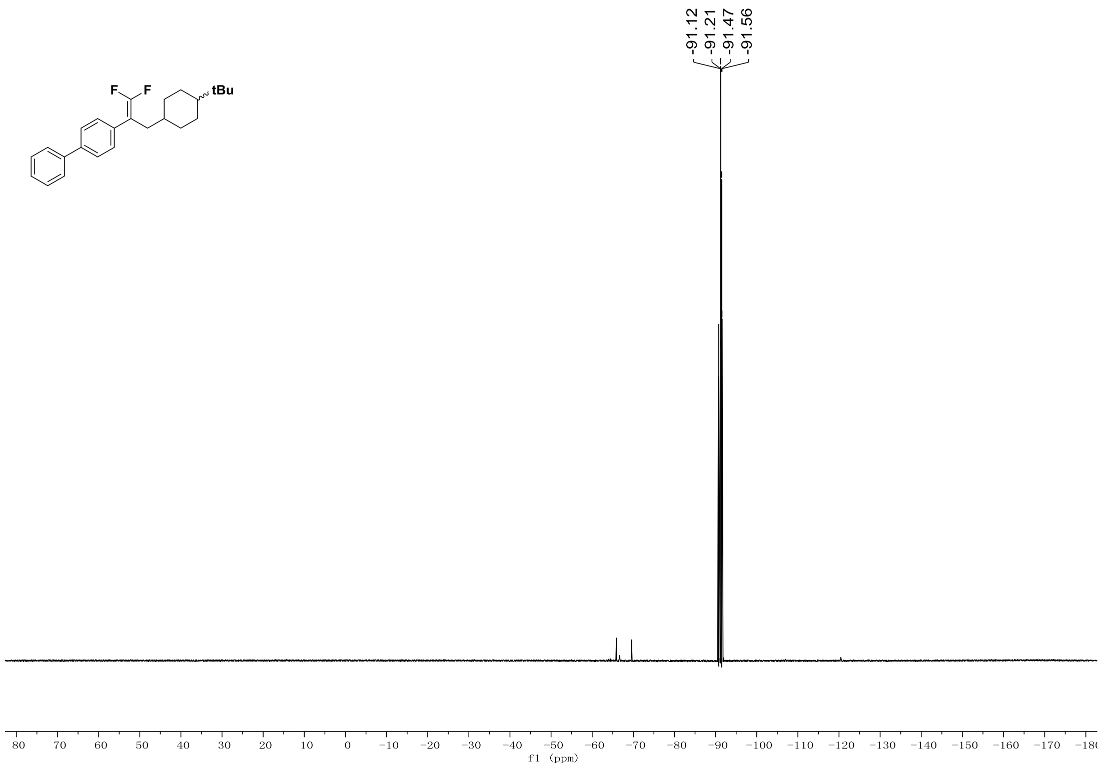

Supplementary Figure 54. ^{19}F NMR Spectra of product **18**

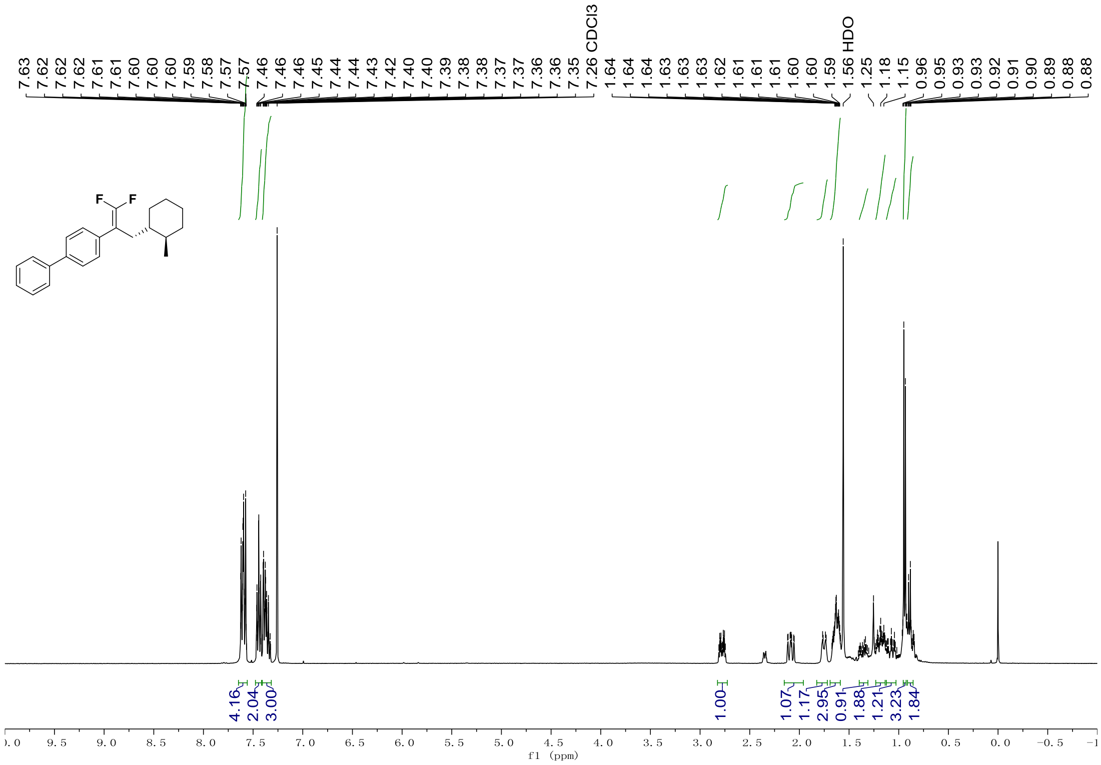

Supplementary Figure 55. ^{1}H NMR Spectra of product **19**

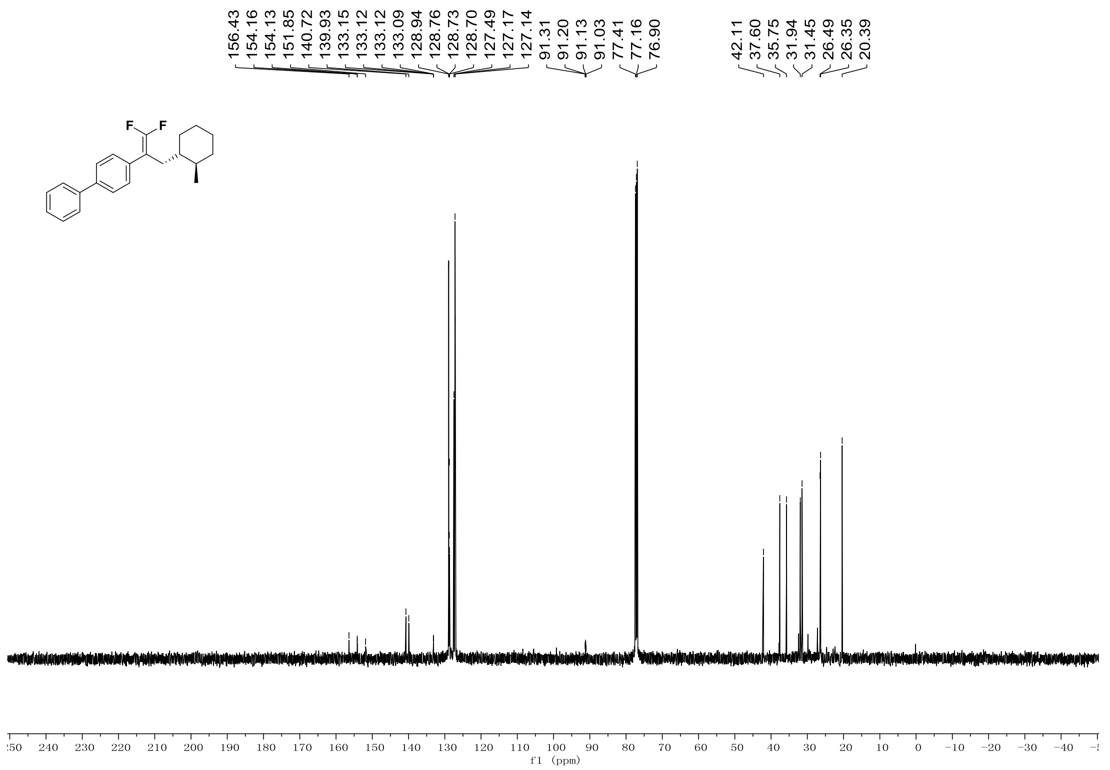

Supplementary Figure 56. ^{13}C NMR Spectra of product **19**

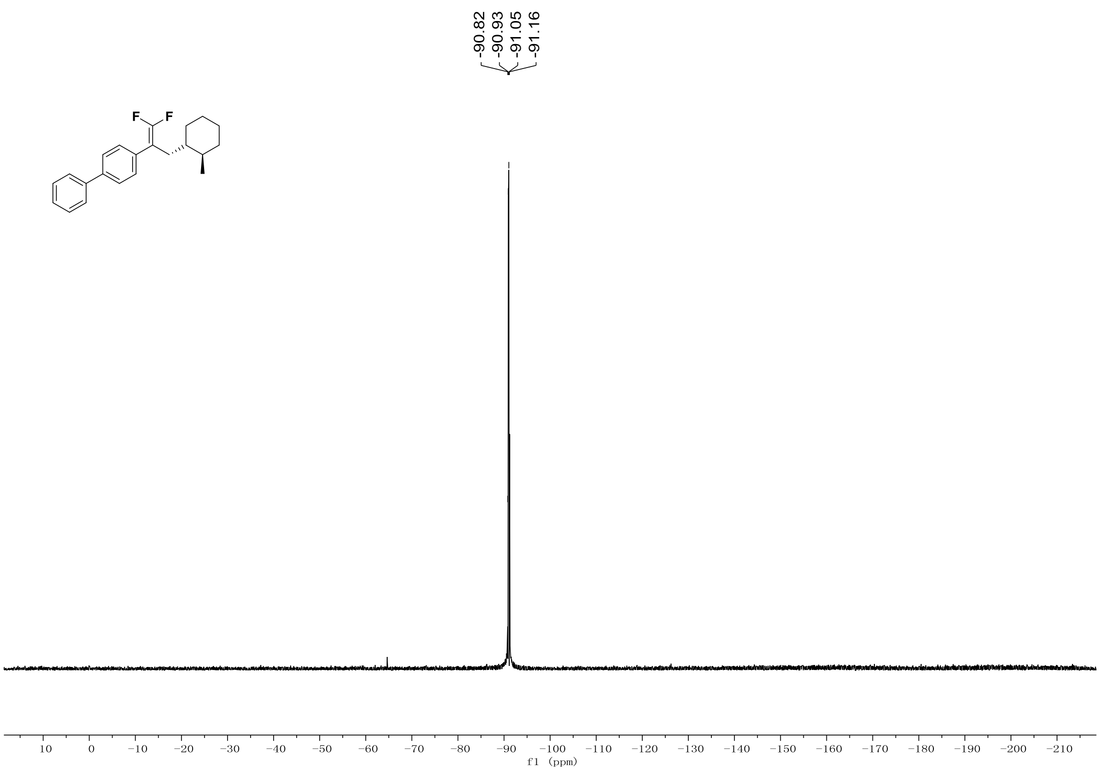

Supplementary Figure 57. ^{19}F NMR Spectra of product **19**

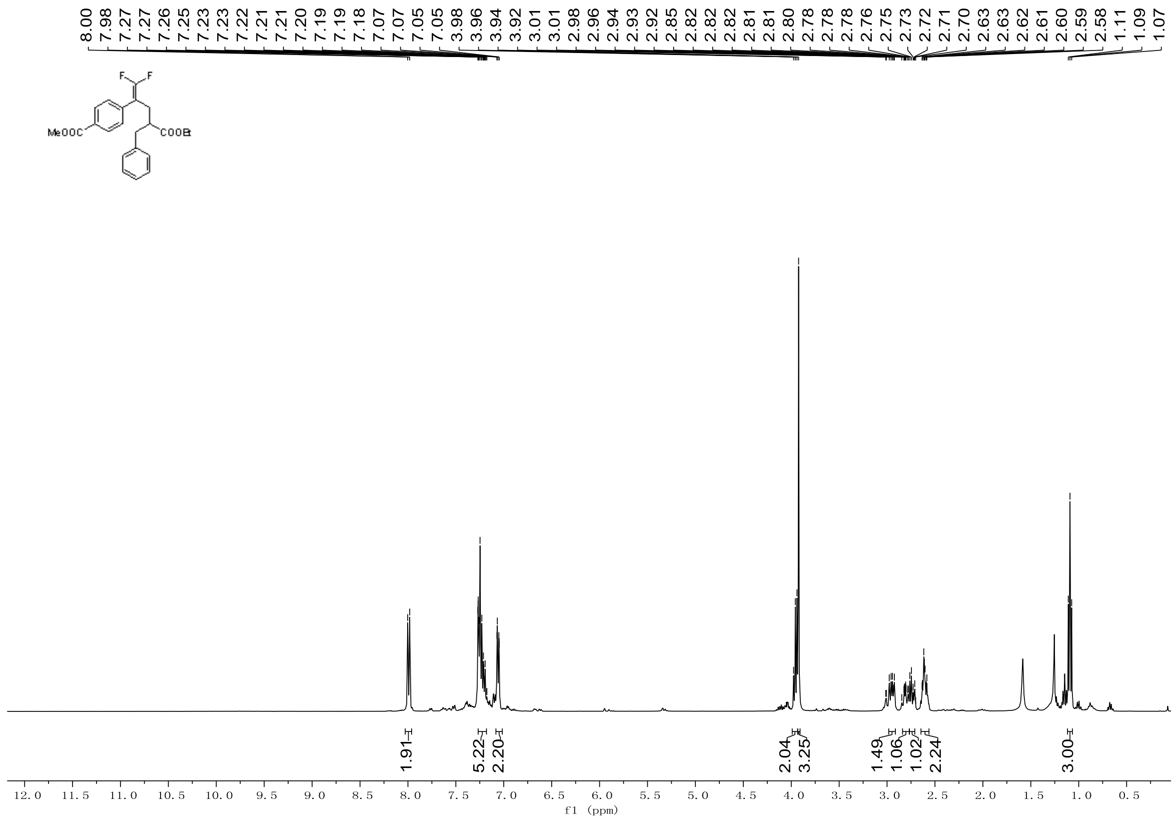

Supplementary Figure 58. ^{1}H NMR Spectra of product **20**

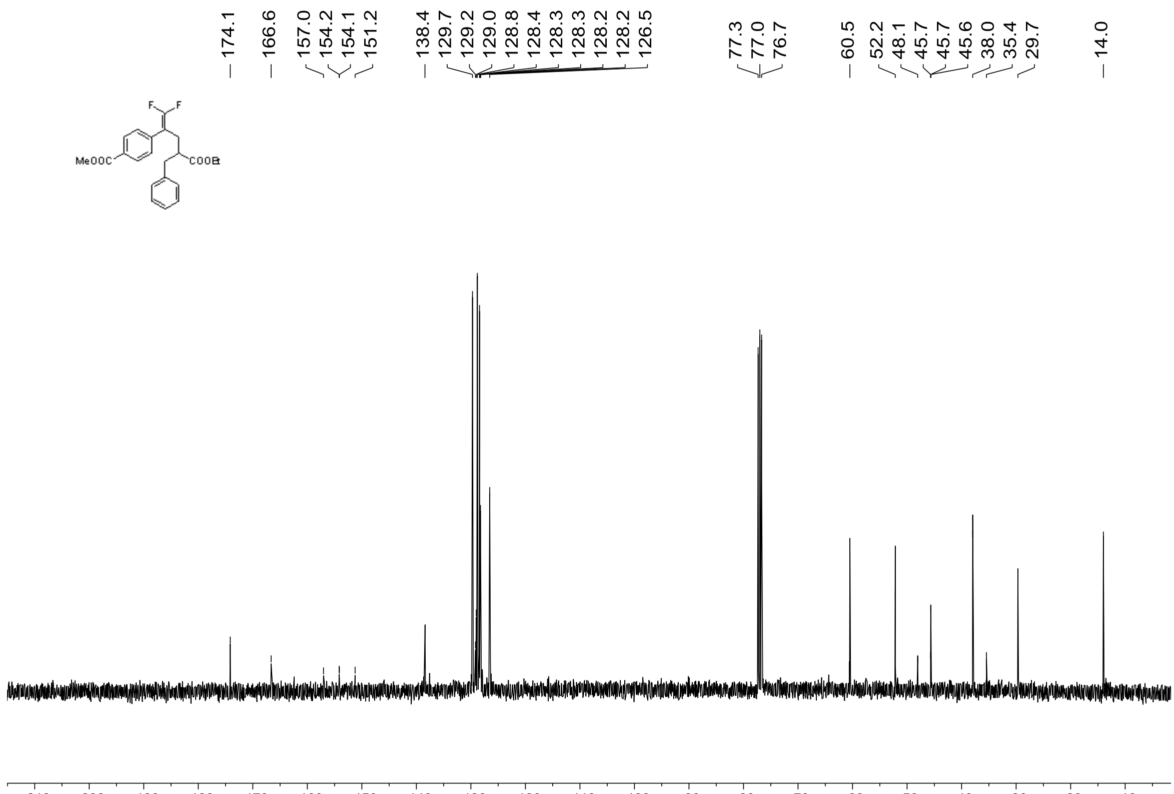

Supplementary Figure 59. ^{13}C NMR Spectra of product **20**

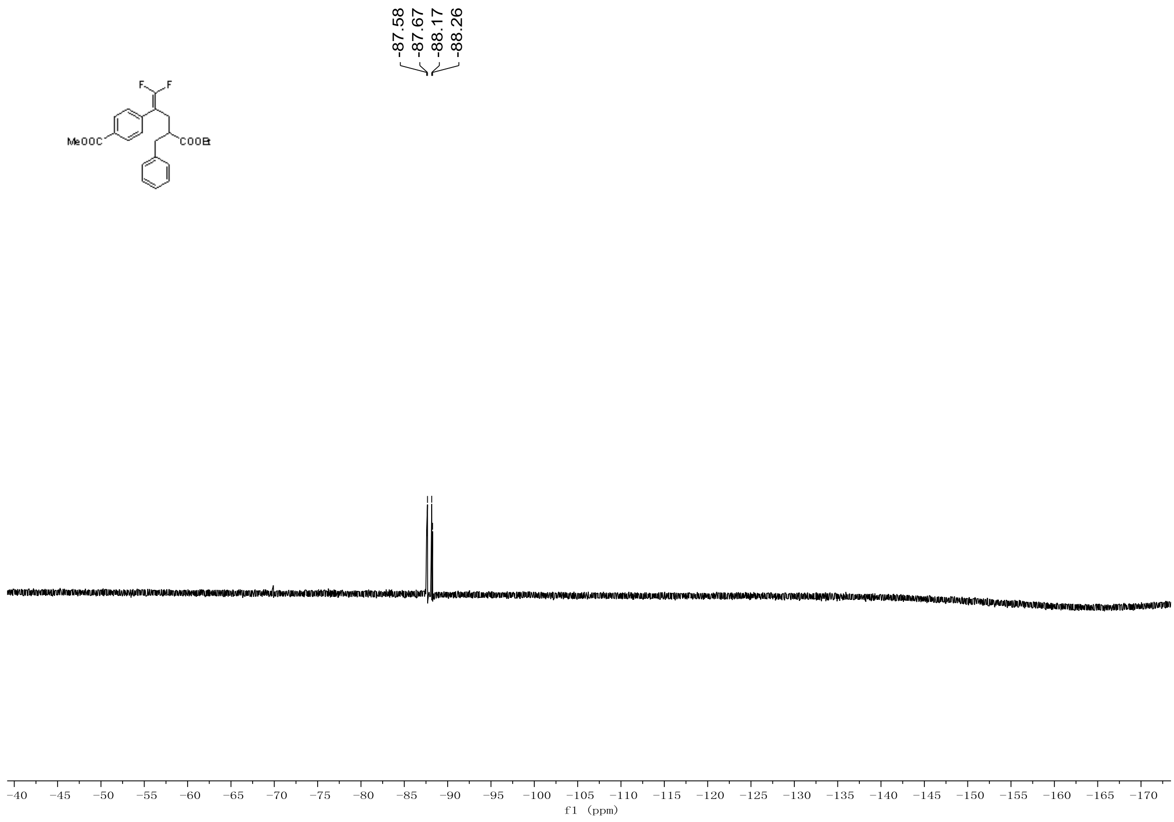

Supplementary Figure 60. ^{19}F NMR Spectra of product **20**

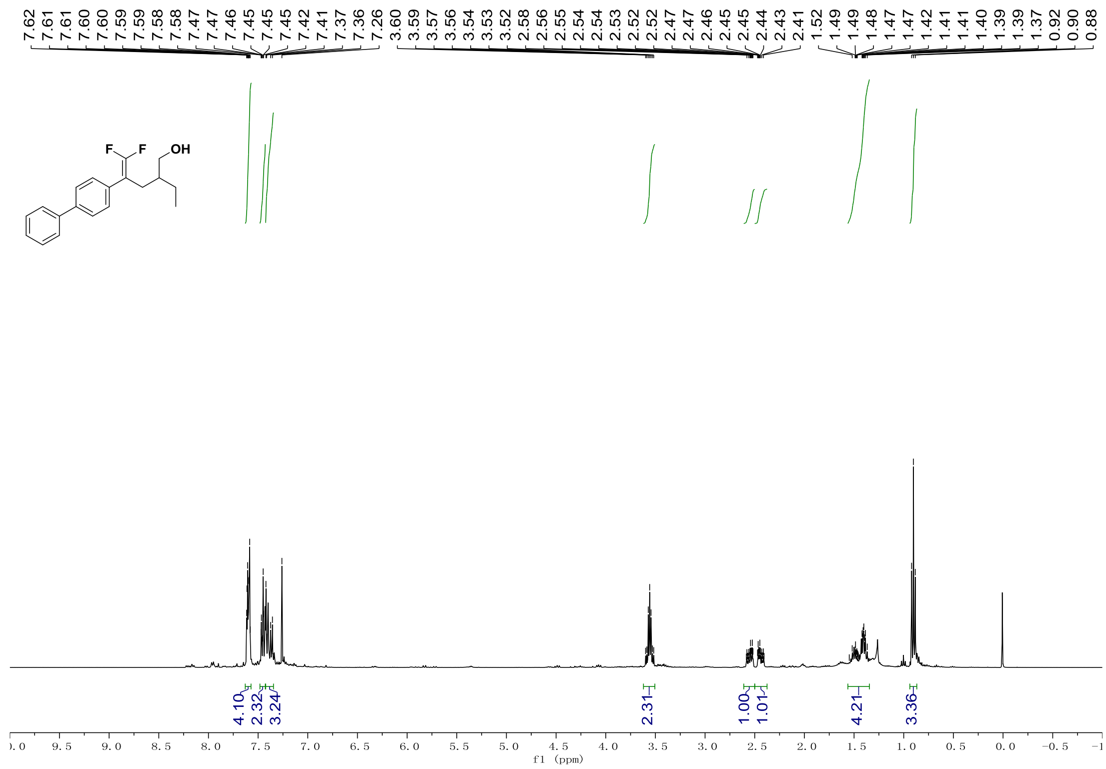

Supplementary Figure 61. ^{1}H NMR Spectra of product **21**

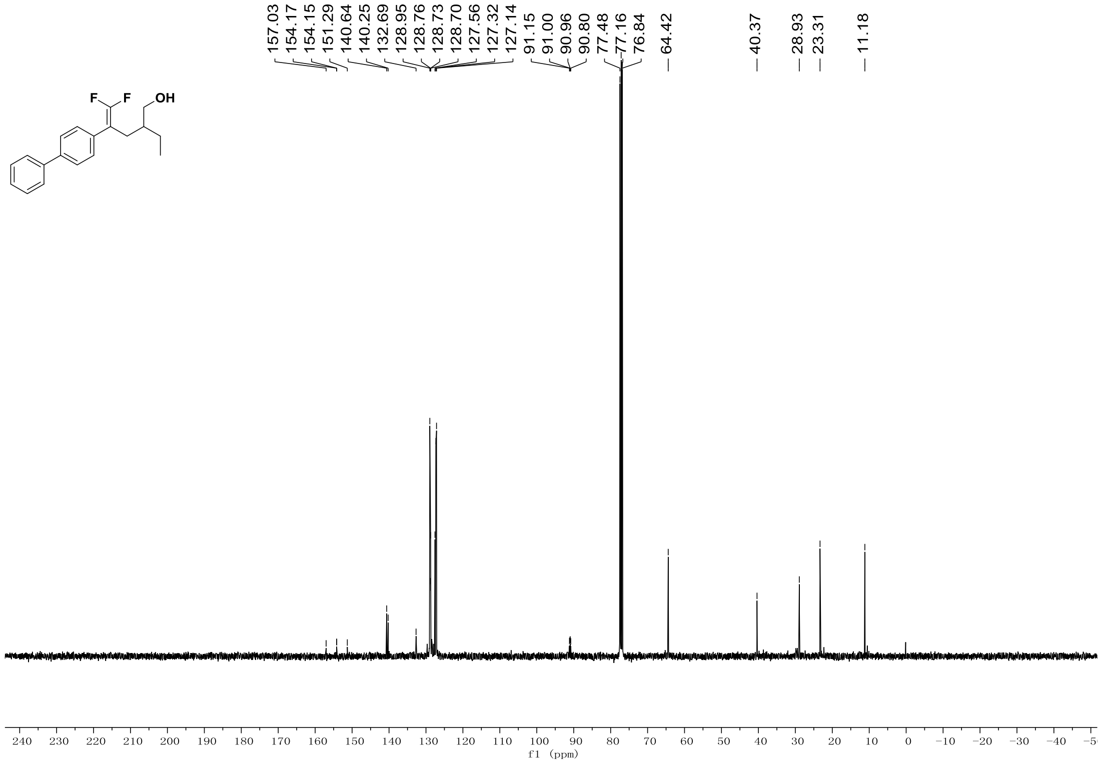

Supplementary Figure 62. ^{13}C NMR Spectra of product **21**

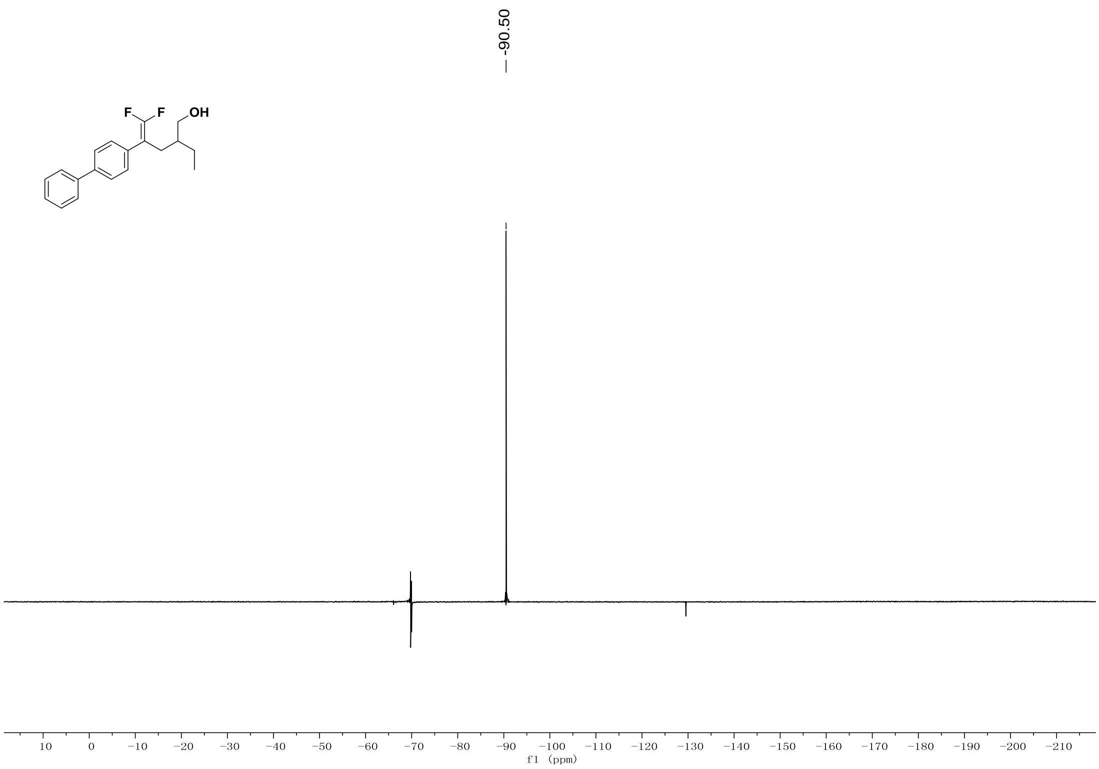

Supplementary Figure 63. ^{19}F NMR Spectra of product **21**

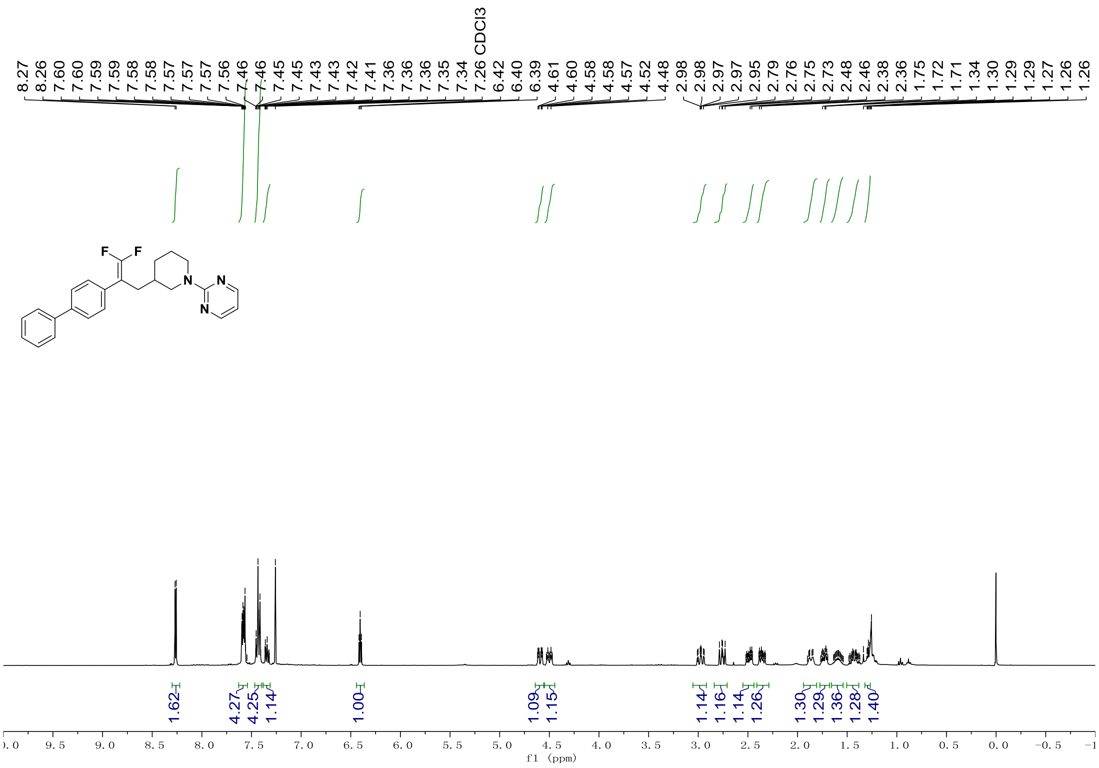

Supplementary Figure 64. ^{1}H NMR Spectra of product **22**

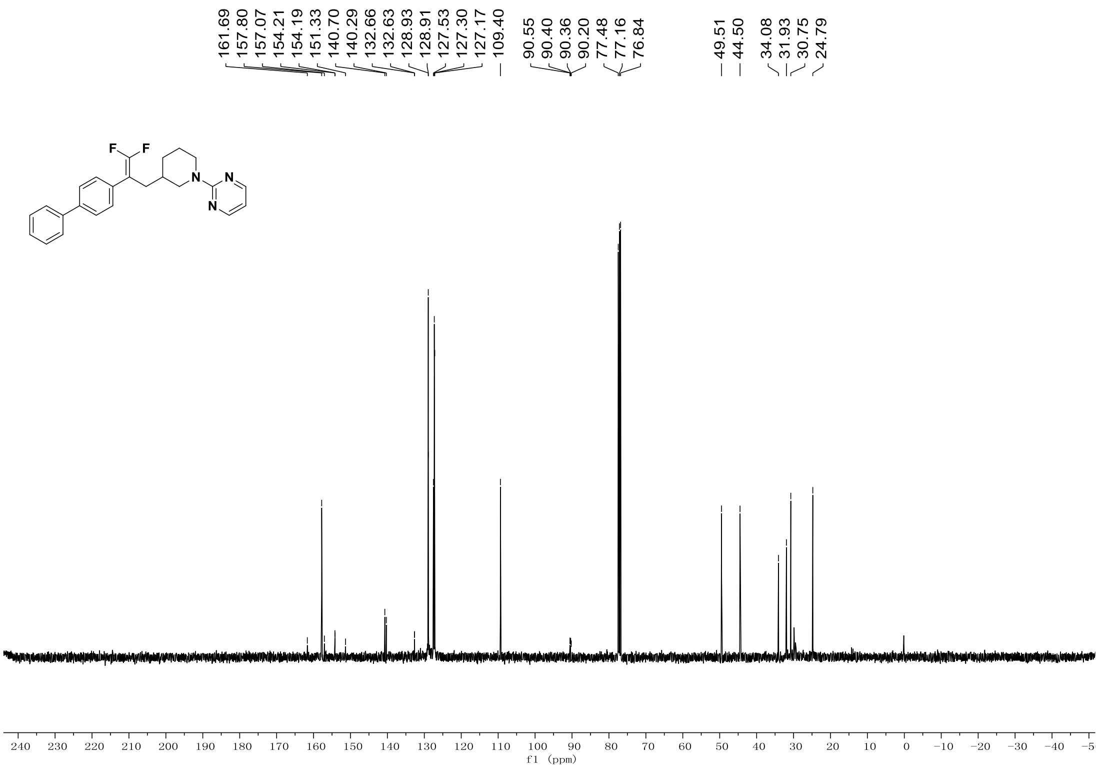

Supplementary Figure 65. ^{13}C NMR Spectra of product **22**

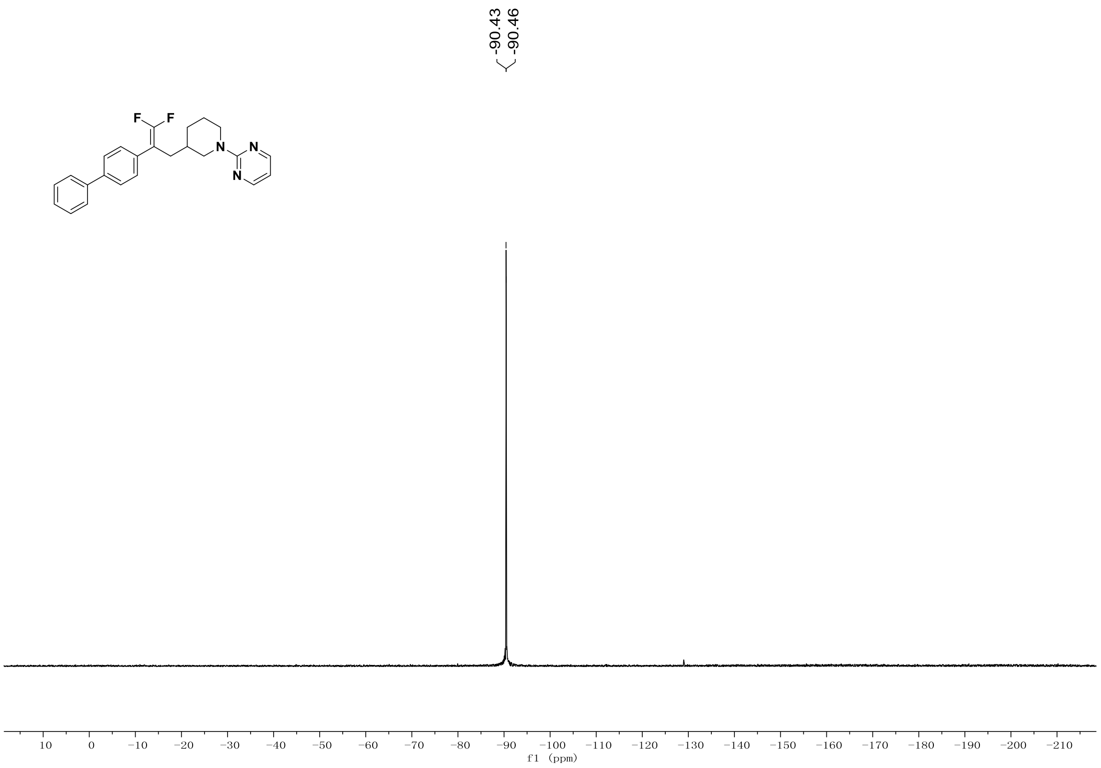

Supplementary Figure 66. ^{19}F NMR Spectra of product **22**

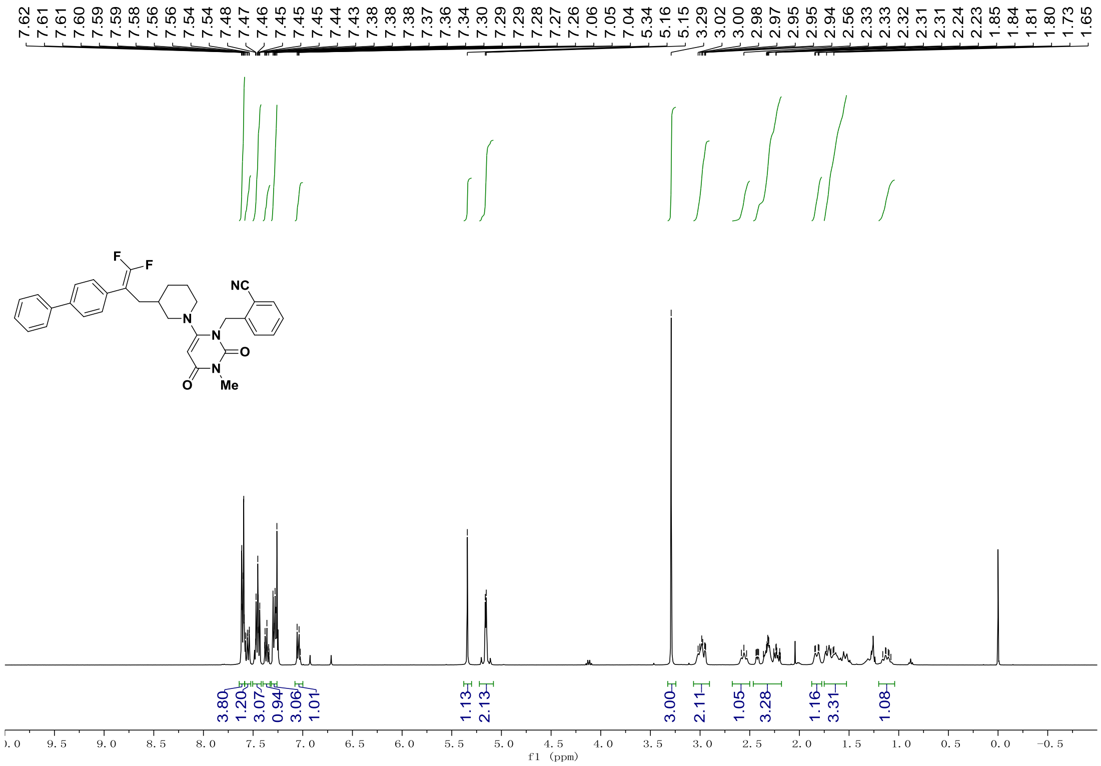

Supplementary Figure 67. ^{1}H NMR Spectra of product **23**

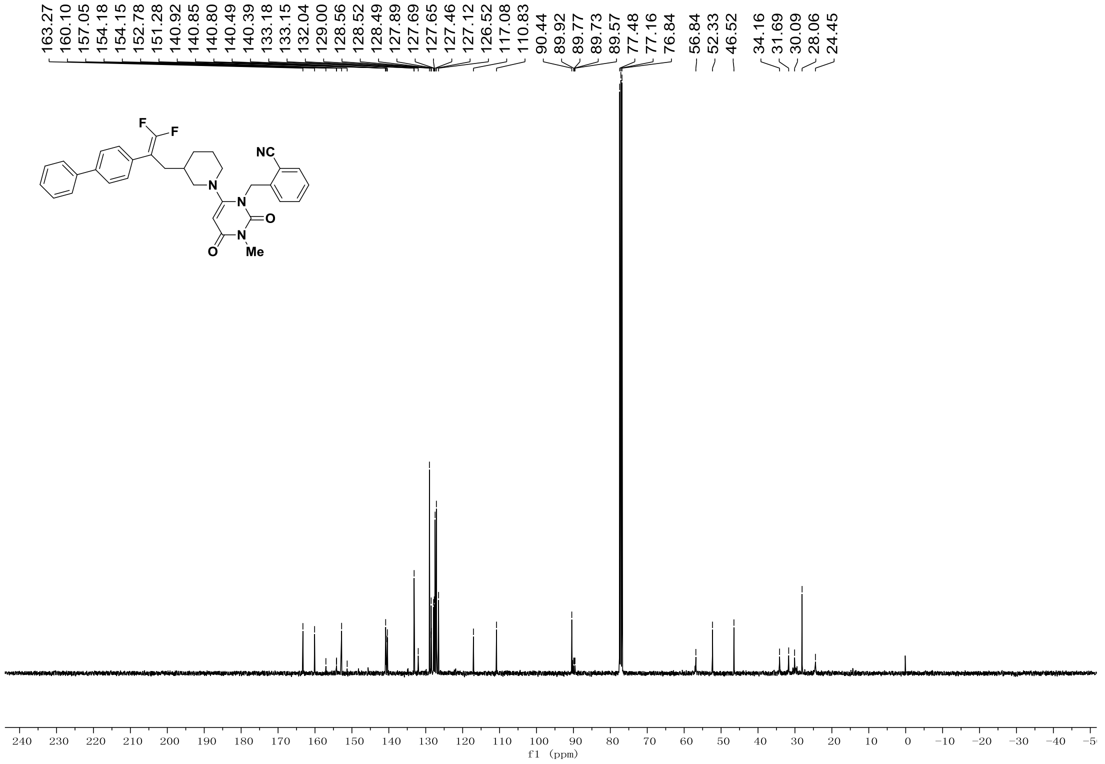

Supplementary Figure 68. ^{13}C NMR Spectra of product **23**

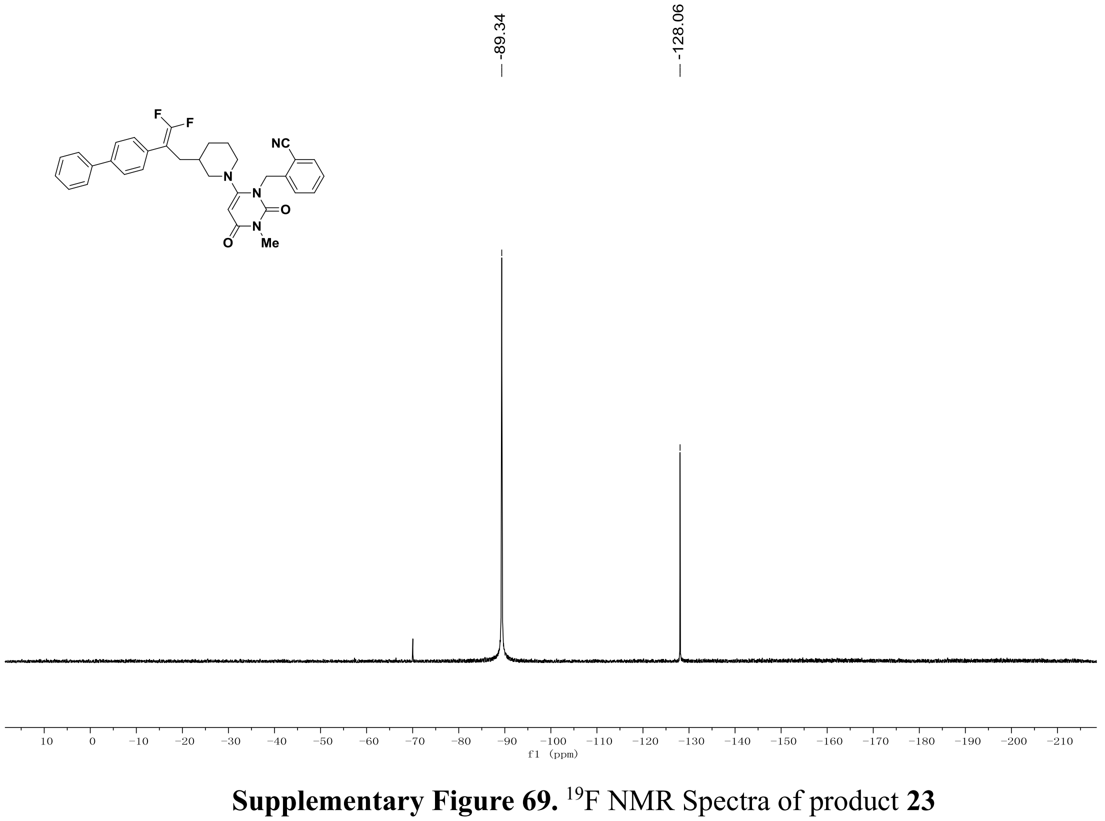

Supplementary Figure 69. ^{19}F NMR Spectra of product **23**

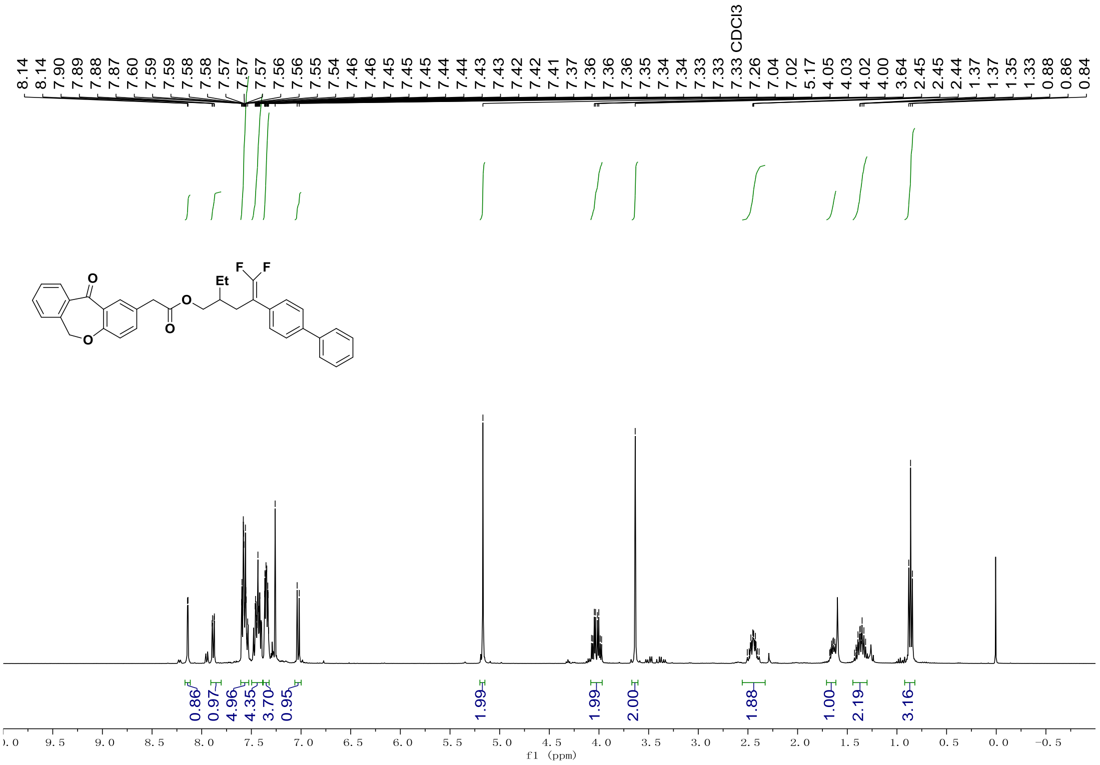

Supplementary Figure 70. ^{19}F NMR Spectra of product **24**

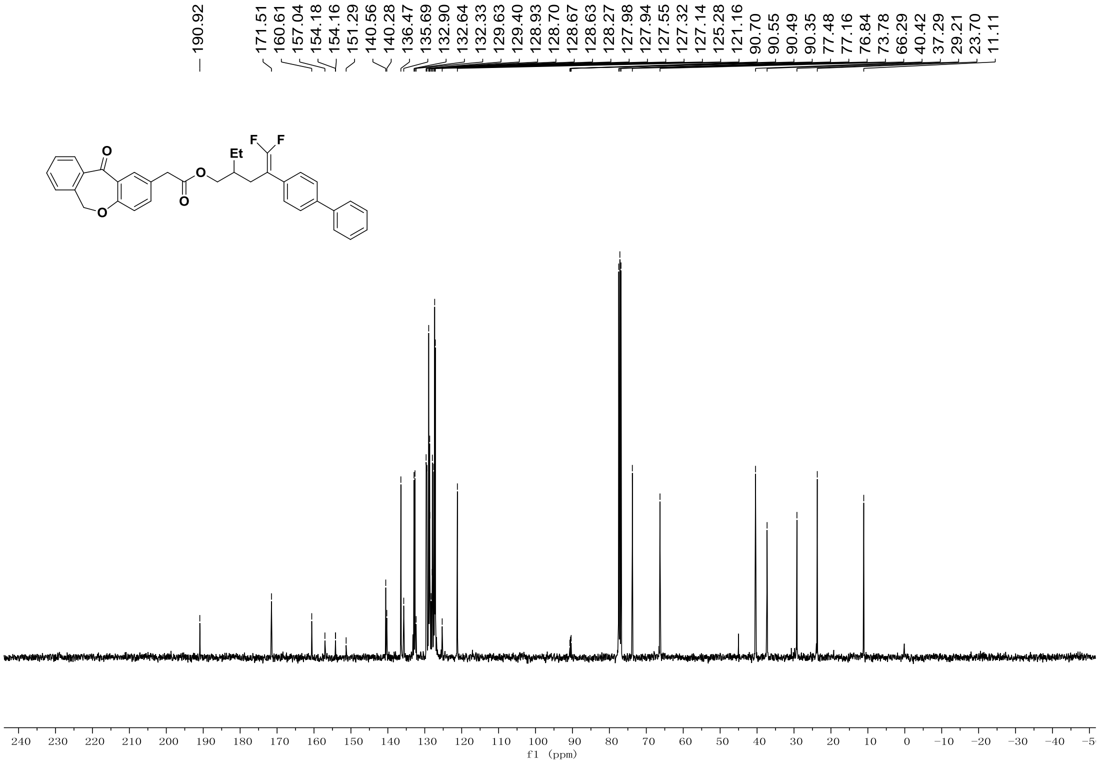

Supplementary Figure 71. ^{19}F NMR Spectra of product **24**

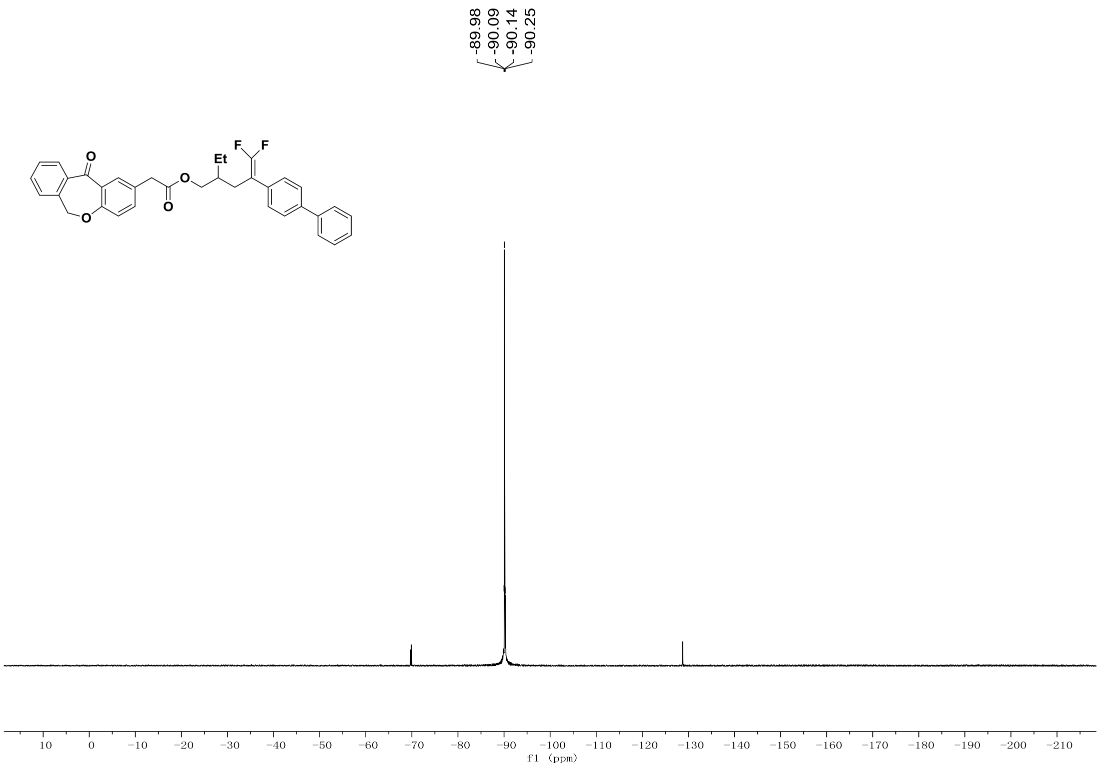

Supplementary Figure 72. ^{19}F NMR Spectra of product **24**

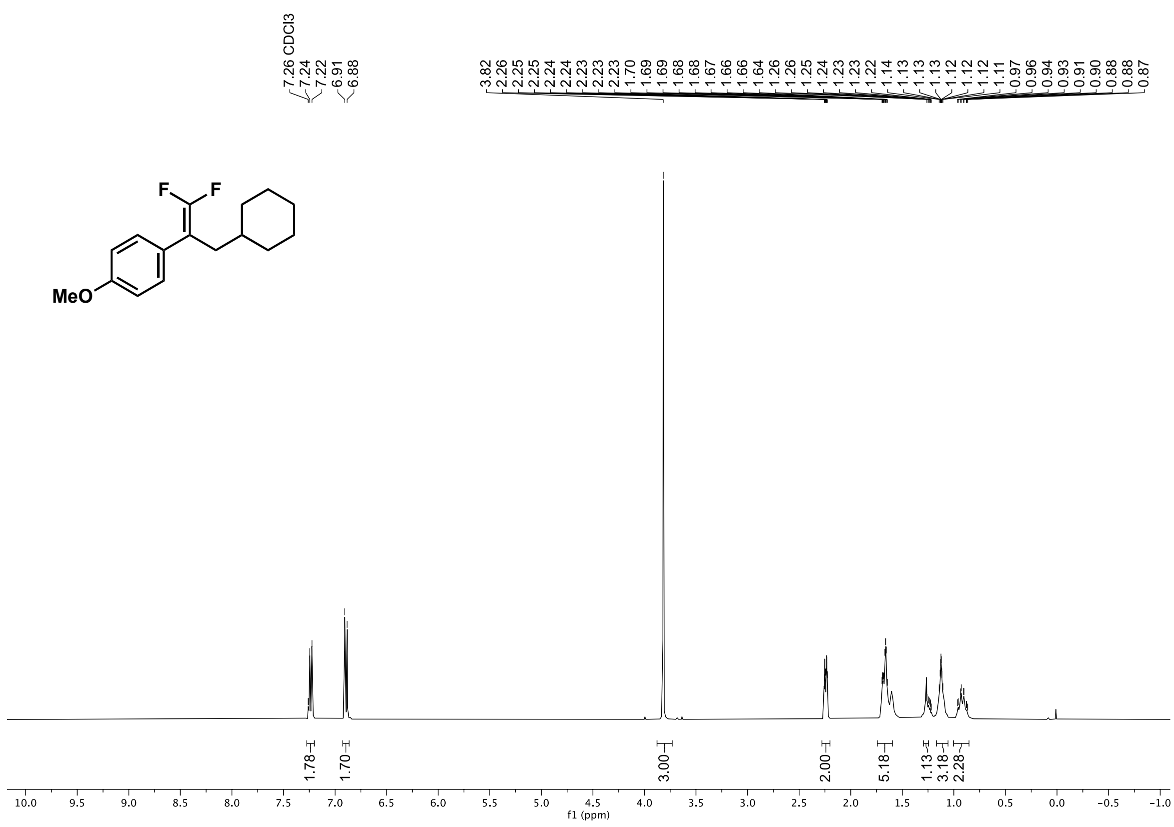

Supplementary Figure 73. ^{1}H NMR Spectra of product **25**

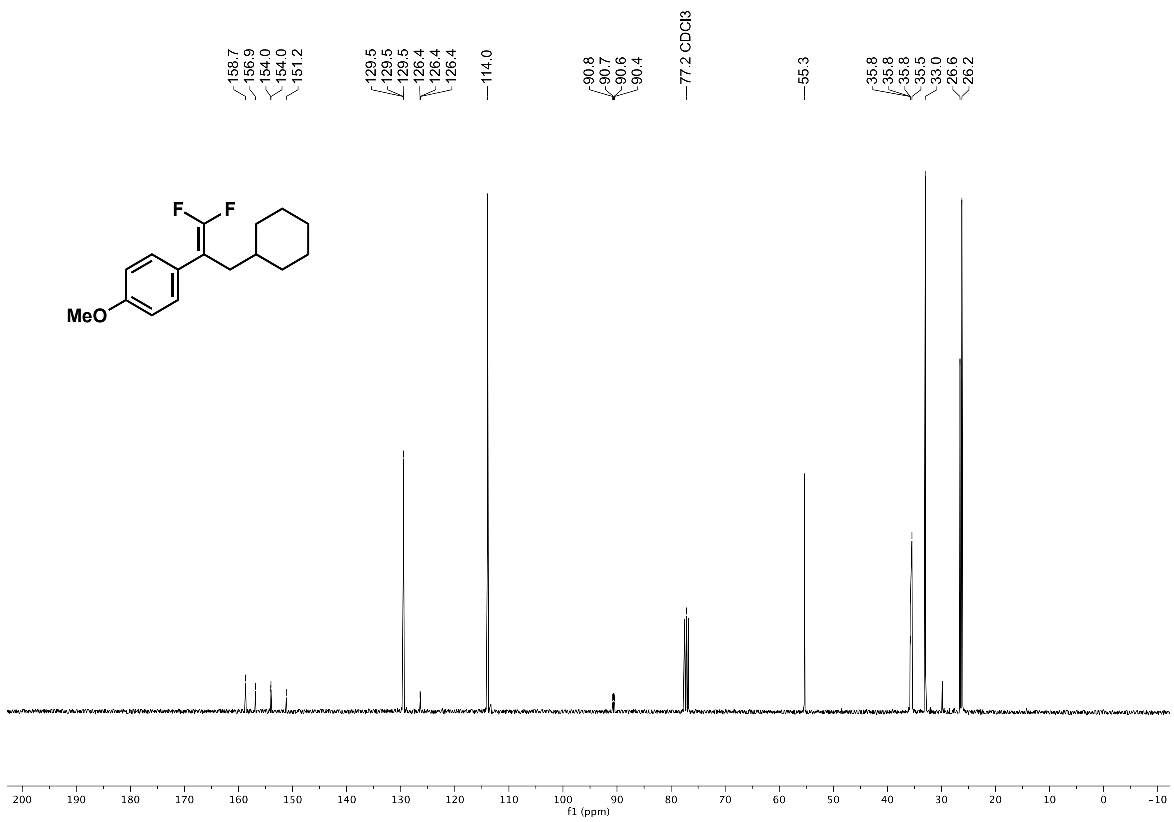

Supplementary Figure 74. ^{13}C NMR Spectra of product **25**

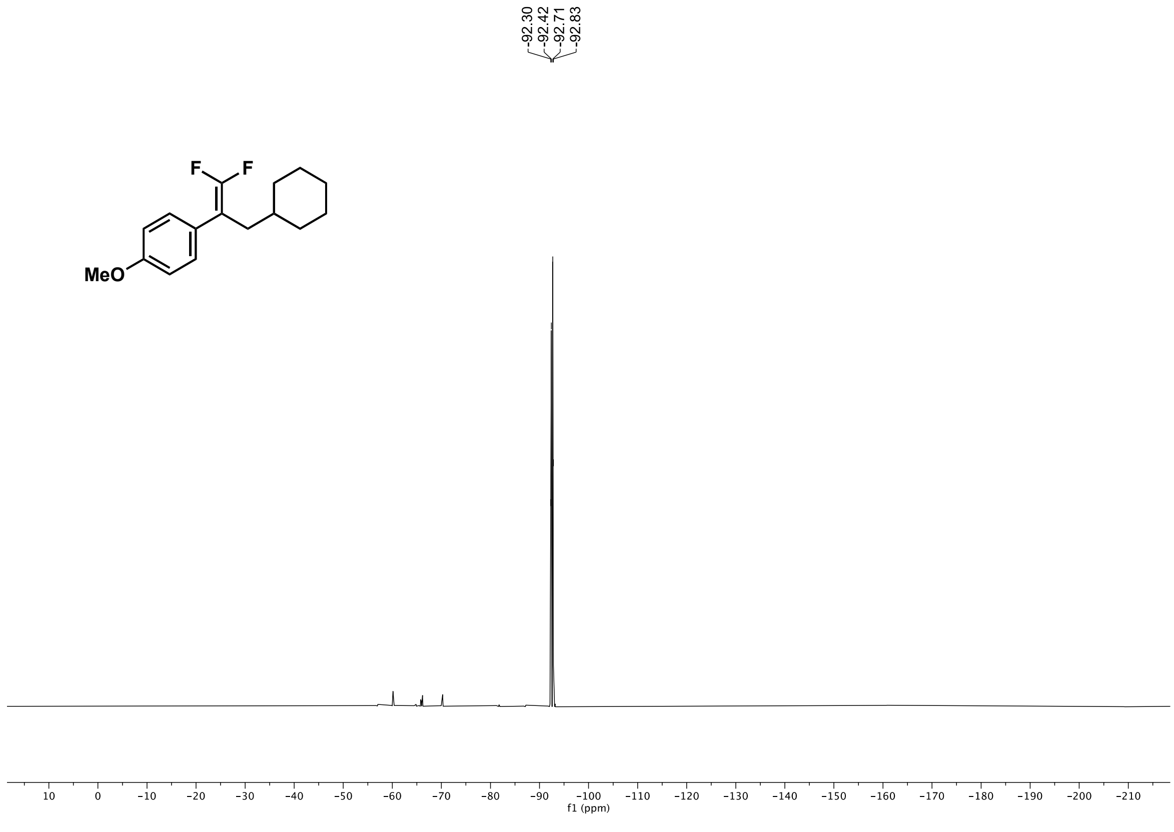

Supplementary Figure 75. ^{19}F NMR Spectra of product **25**

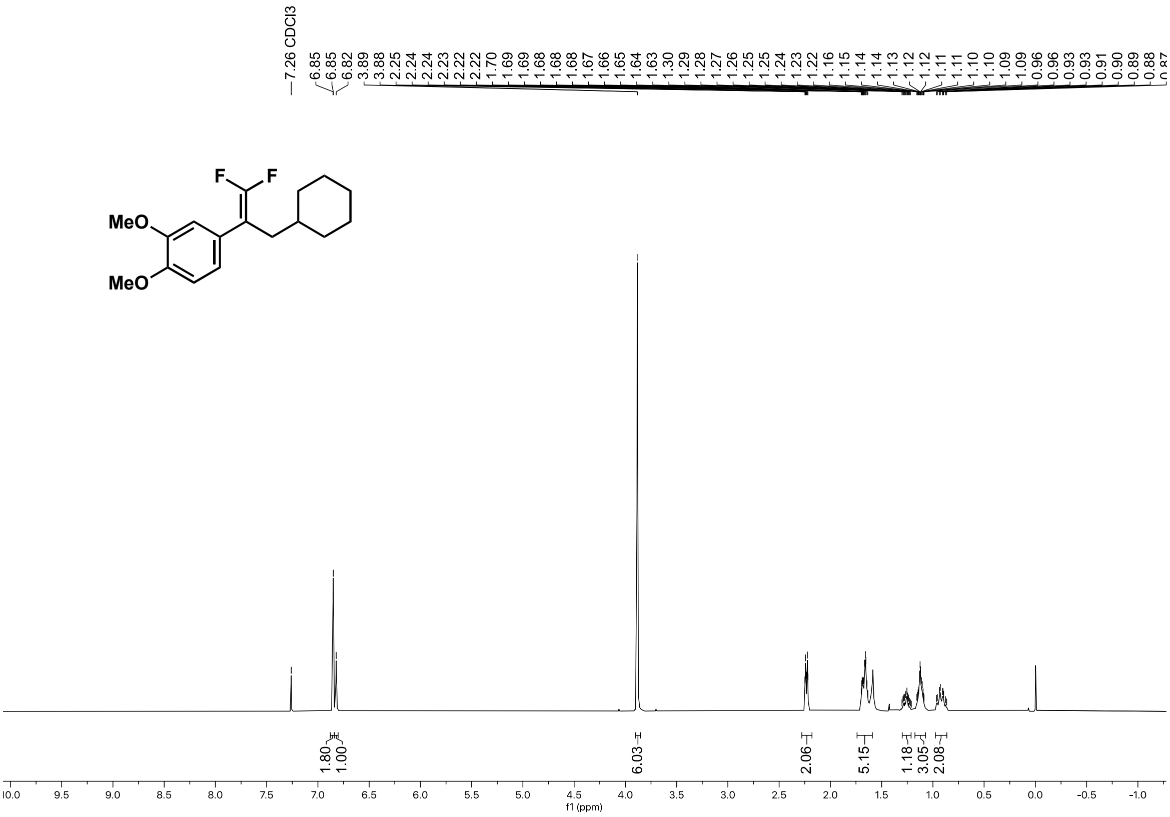

Supplementary Figure 76. ^{1}H NMR Spectra of product **26**

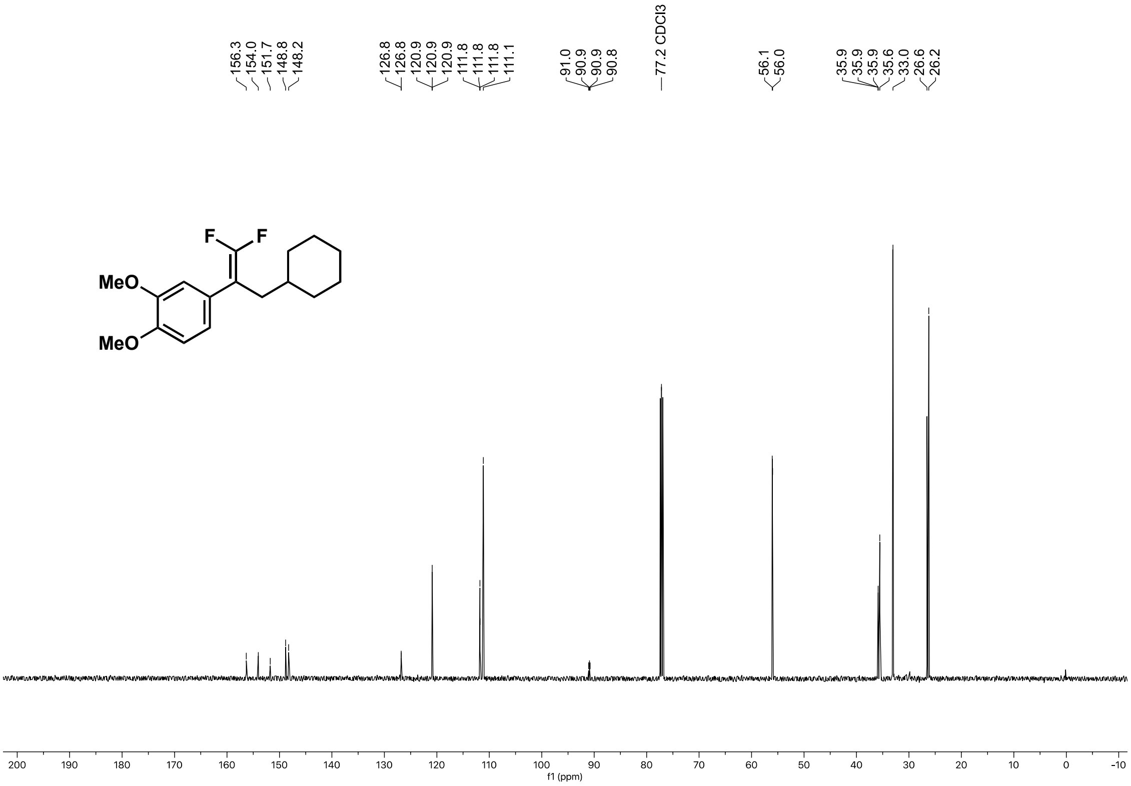

Supplementary Figure 77. ^{13}C NMR Spectra of product **26**

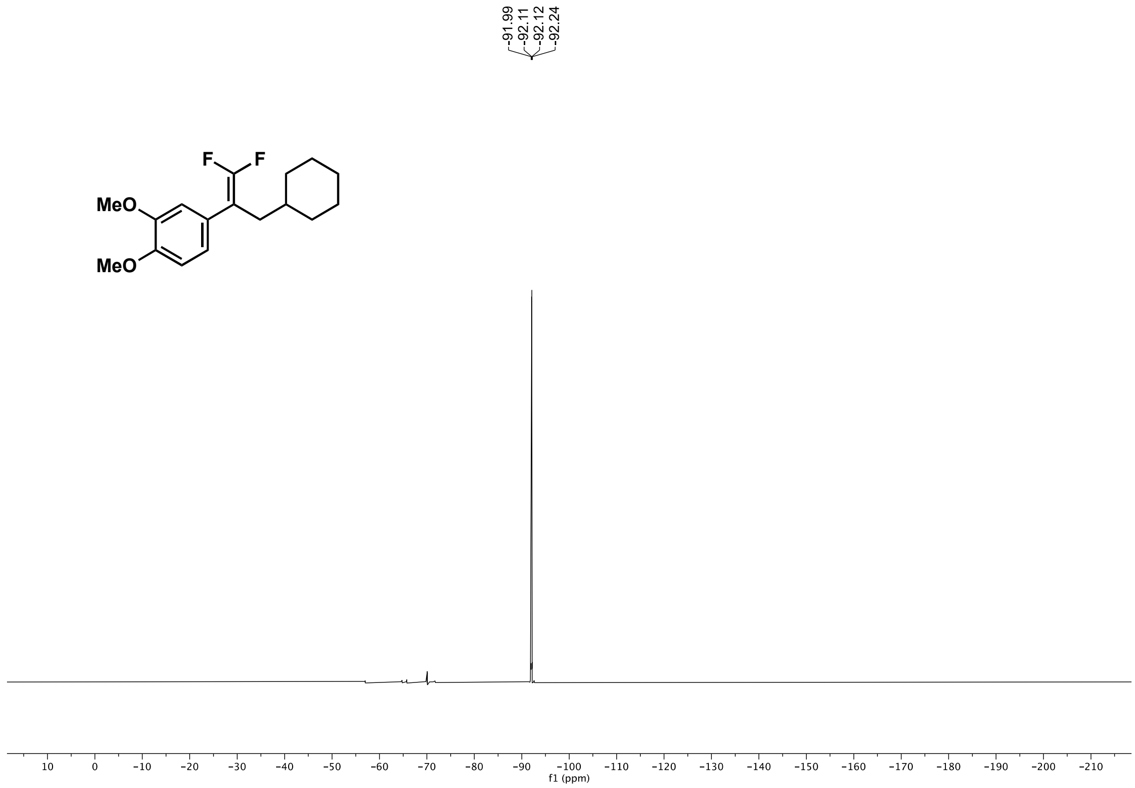

Supplementary Figure 48. ^{19}F NMR Spectra of product **26**

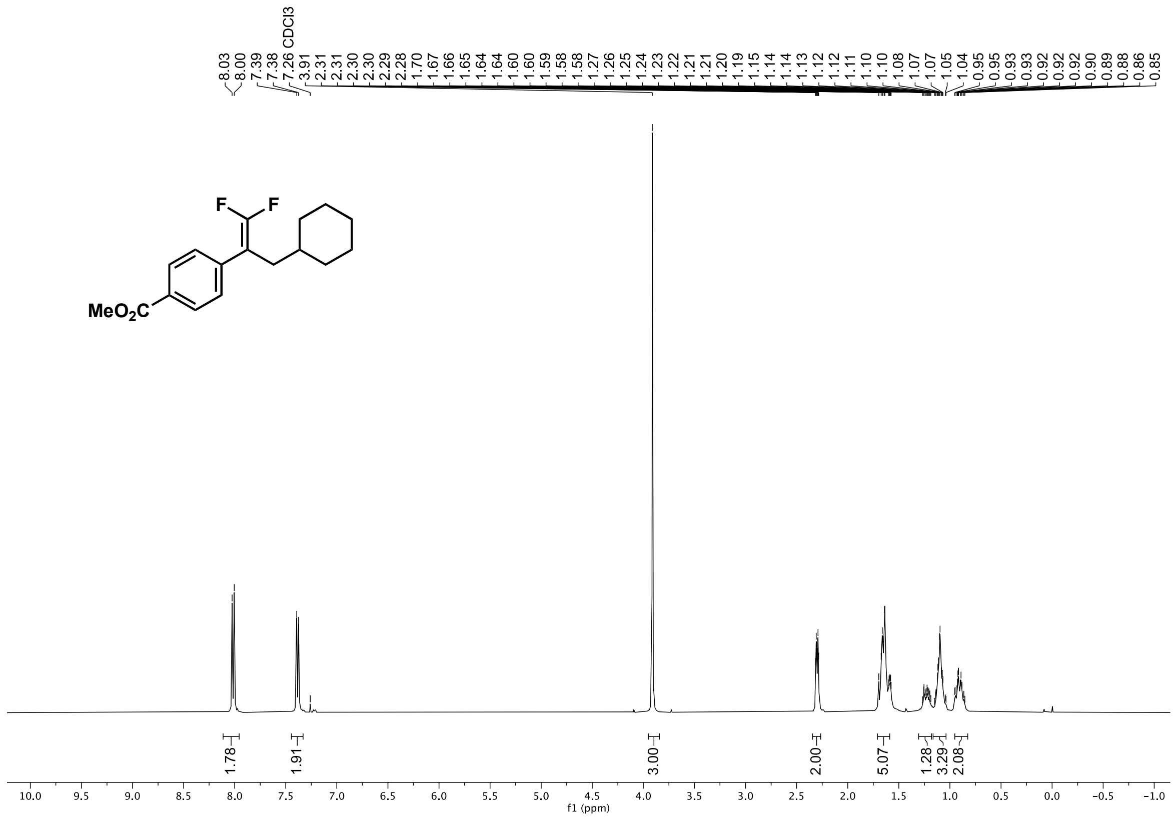

Supplementary Figure 79. ^{1}H NMR Spectra of product **27**

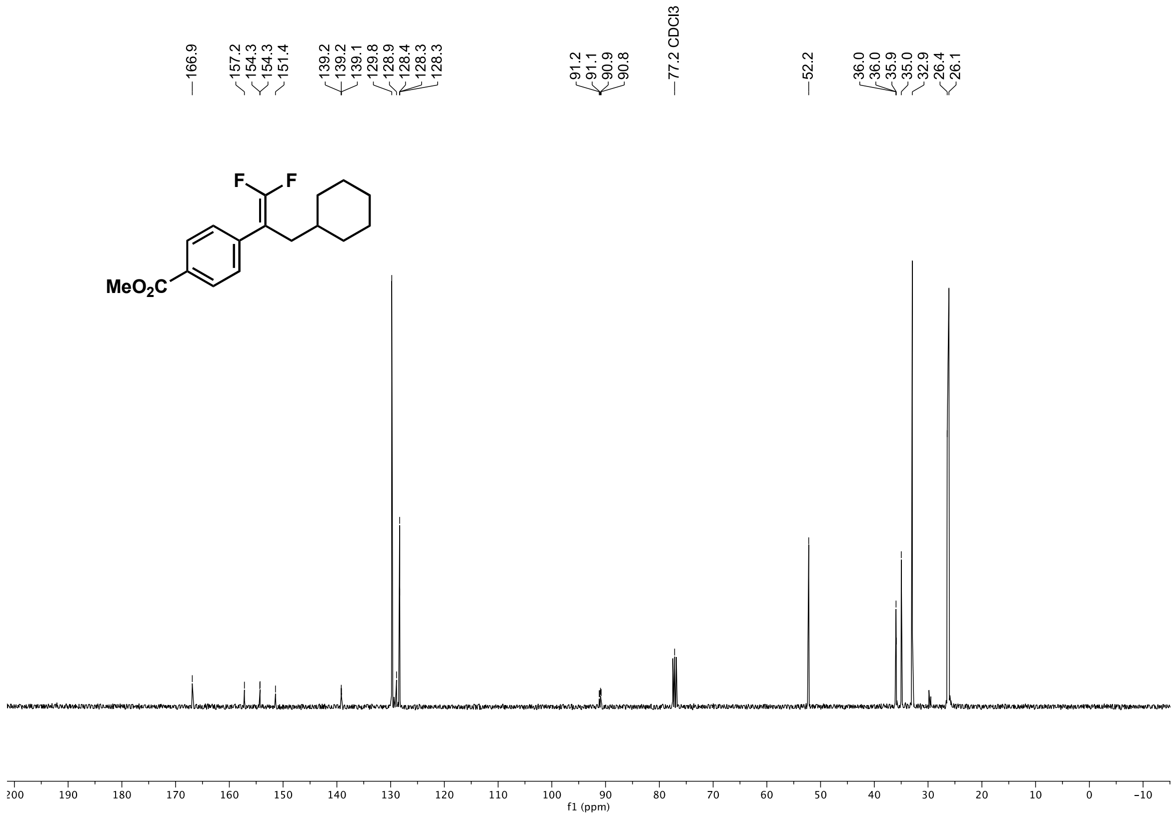

Supplementary Figure 80. ^{13}C NMR Spectra of product **27**

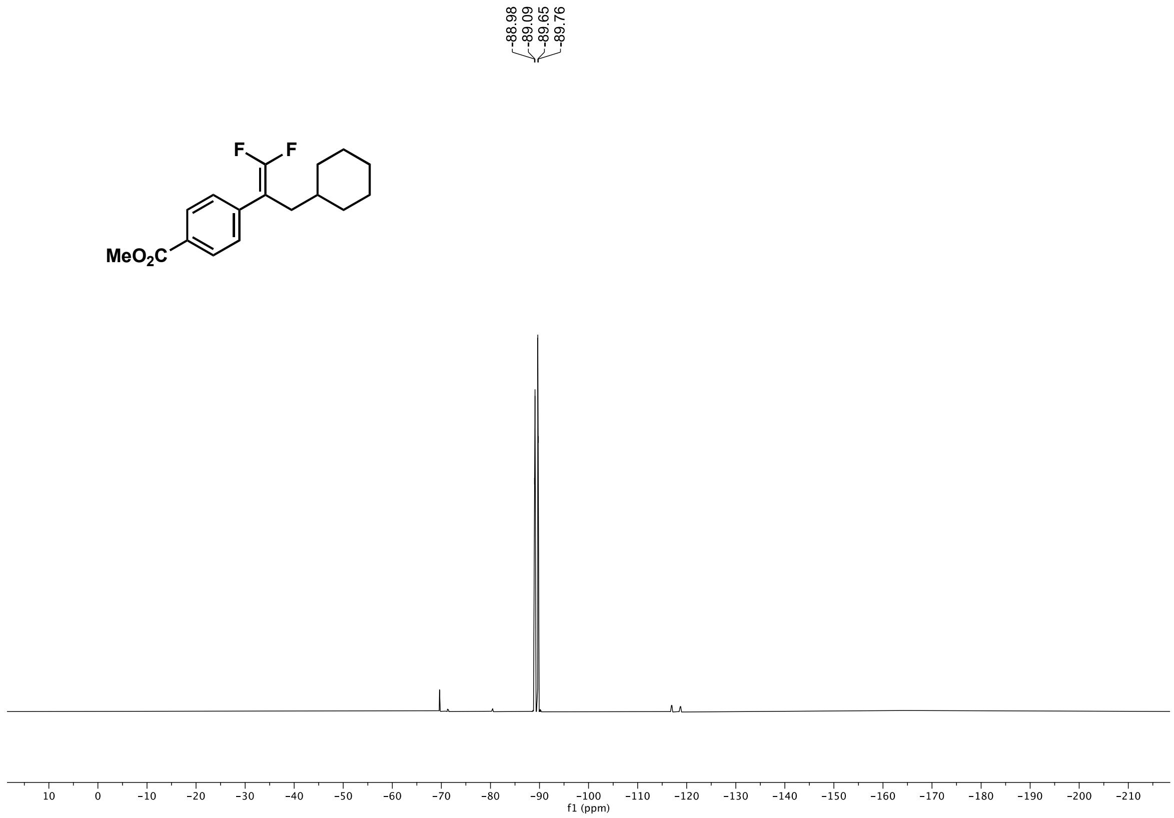

Supplementary Figure 81. ^{19}F NMR Spectra of product **27**

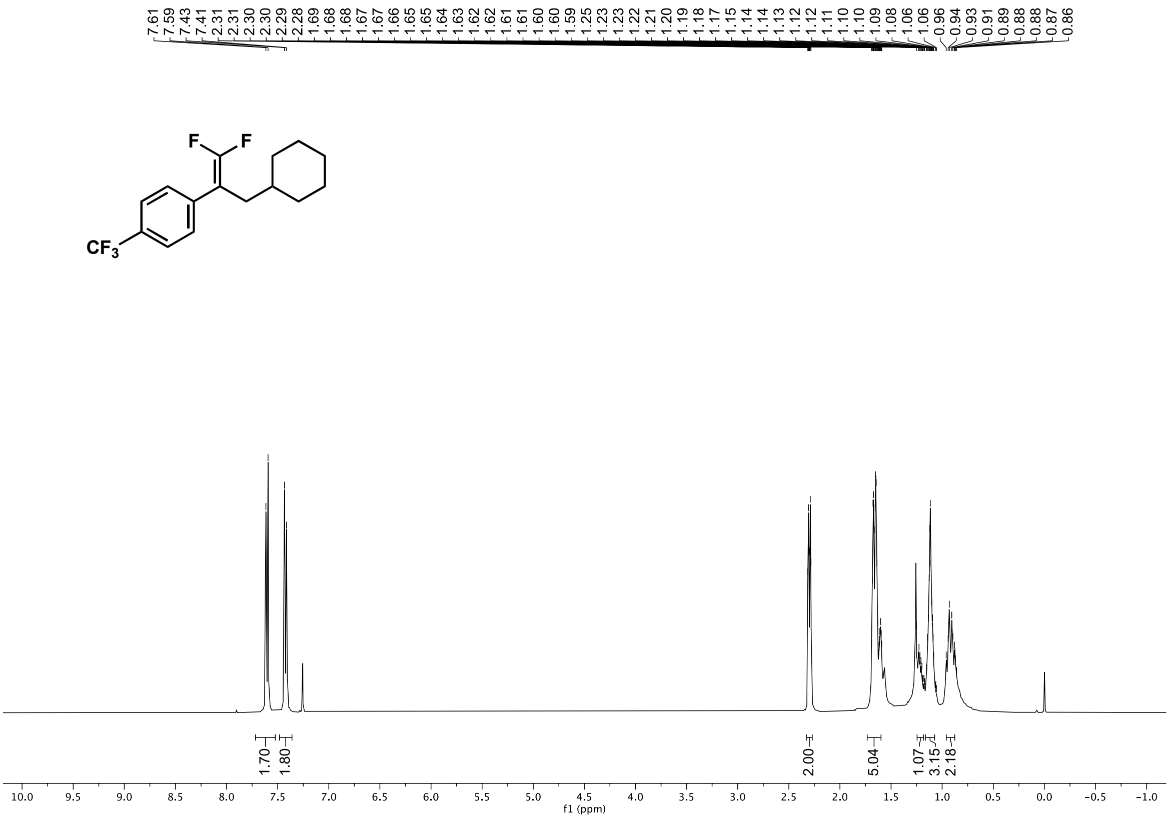

Supplementary Figure 82. ^{1}H NMR Spectra of product **28**

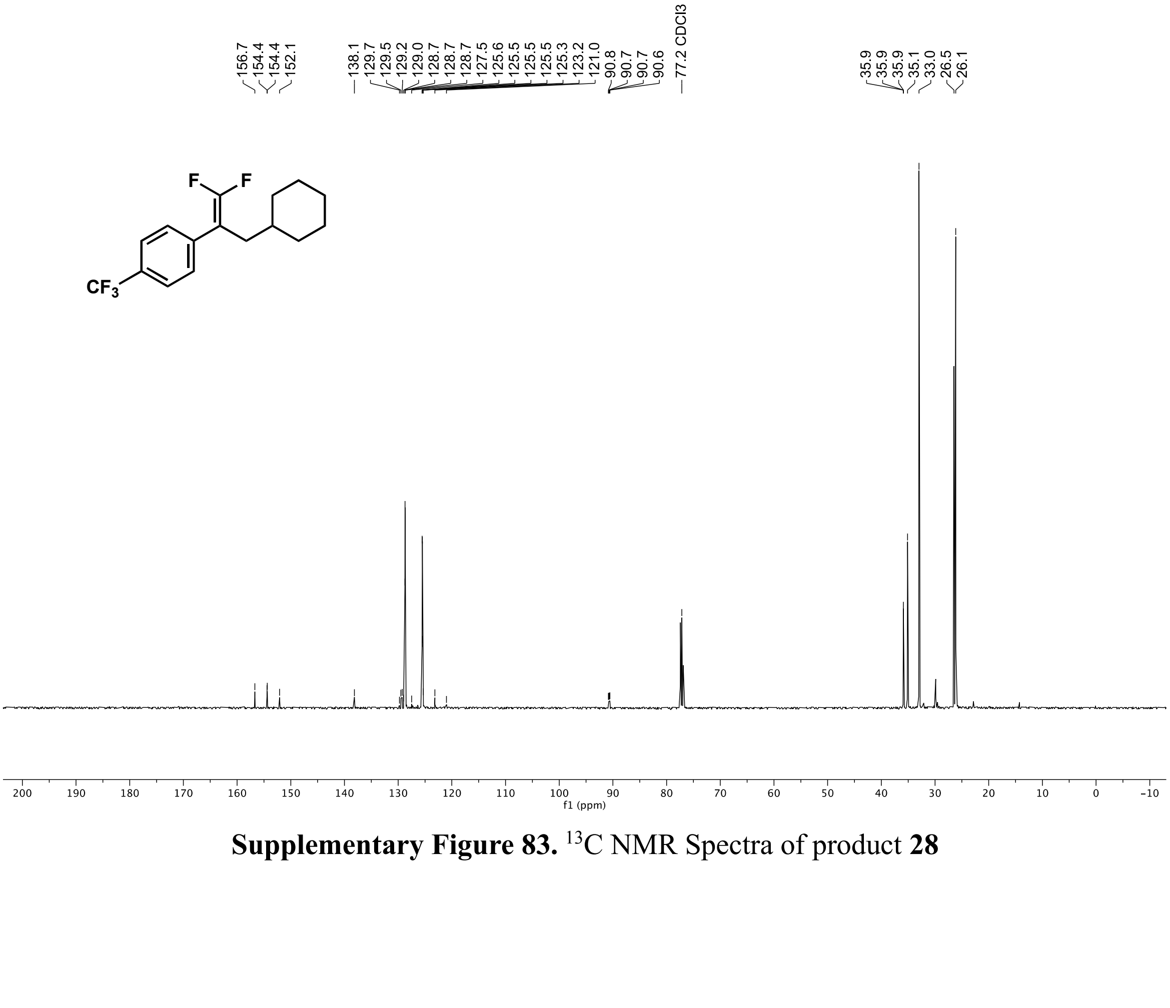

Supplementary Figure 83. ^{13}C NMR Spectra of product **28**

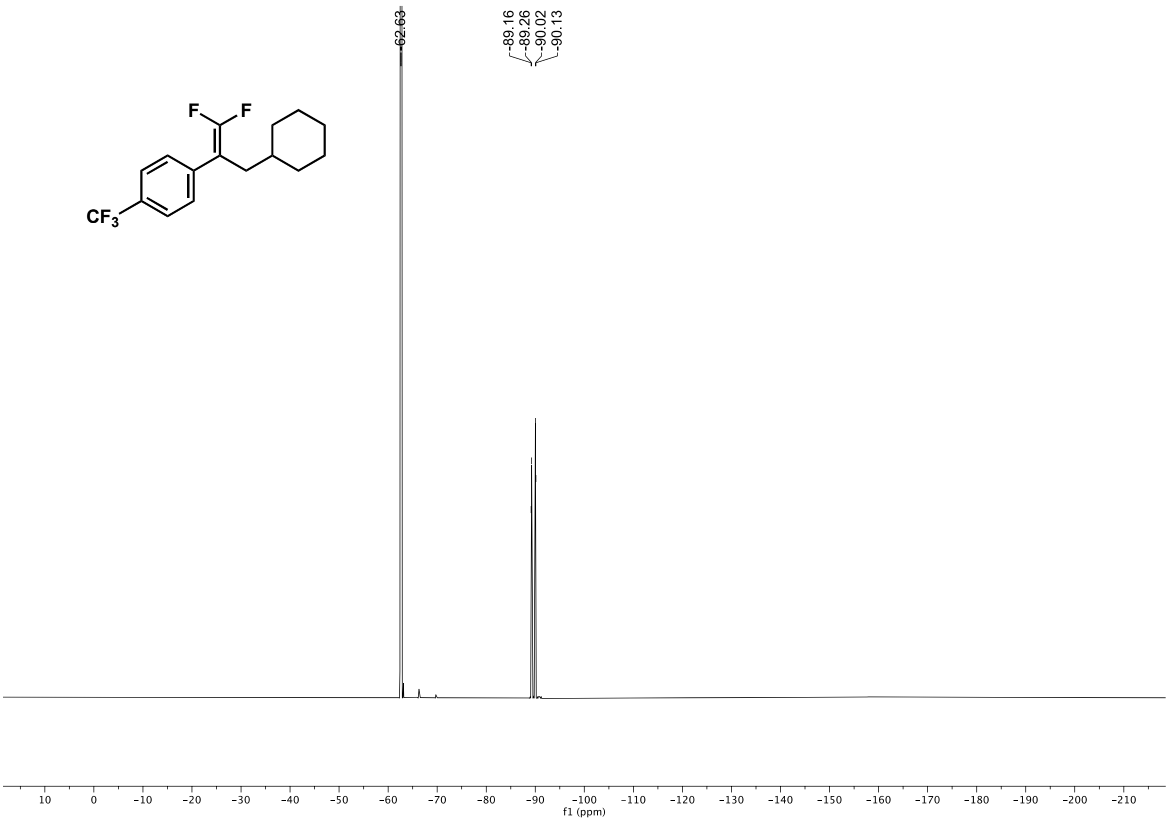

Supplementary Figure 84. ^{19}F NMR Spectra of product **28**

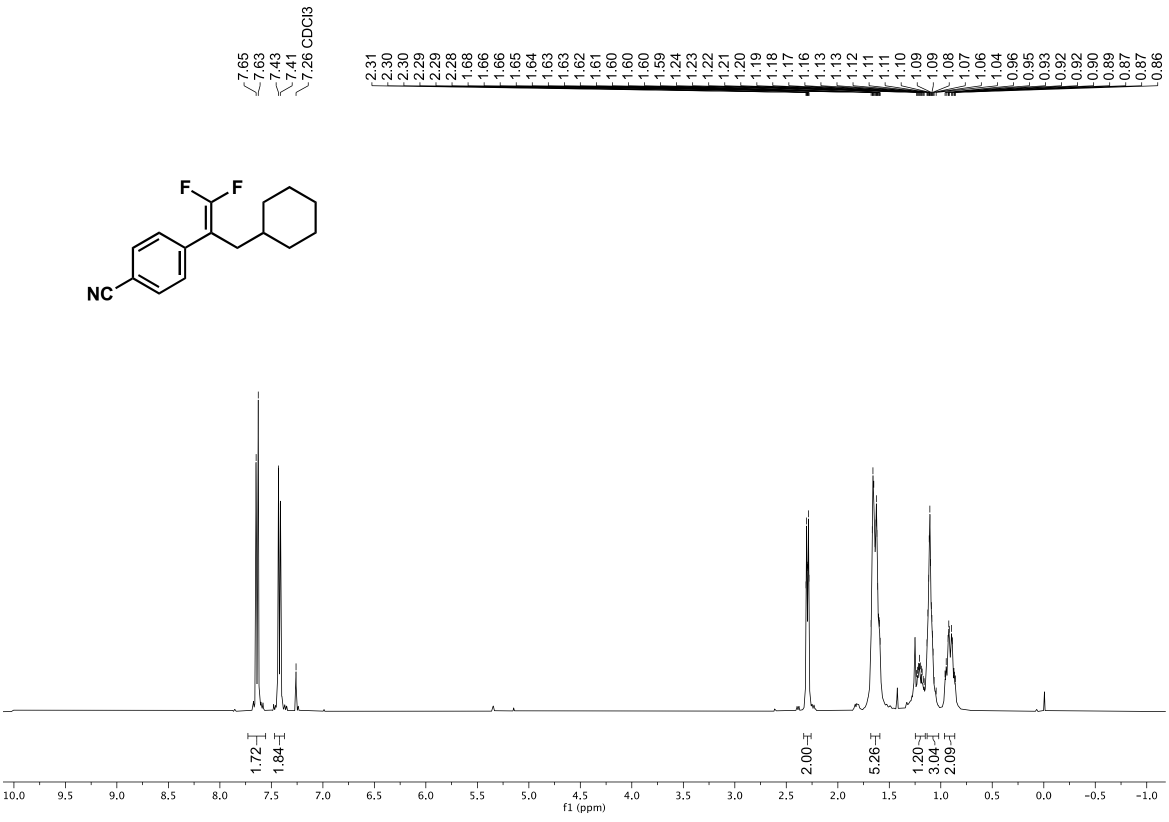

Supplementary Figure 85. ^{1}H NMR Spectra of product **29**

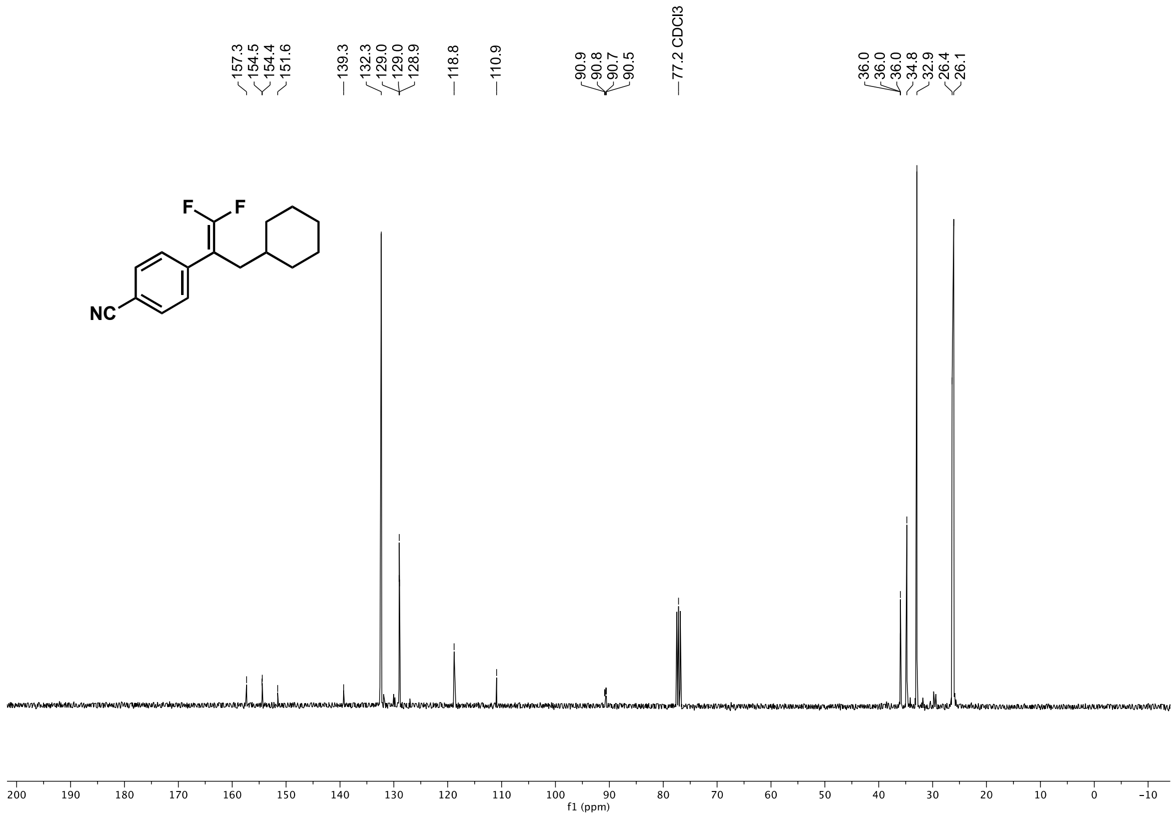

Supplementary Figure 86. ^{13}C NMR Spectra of product **29**

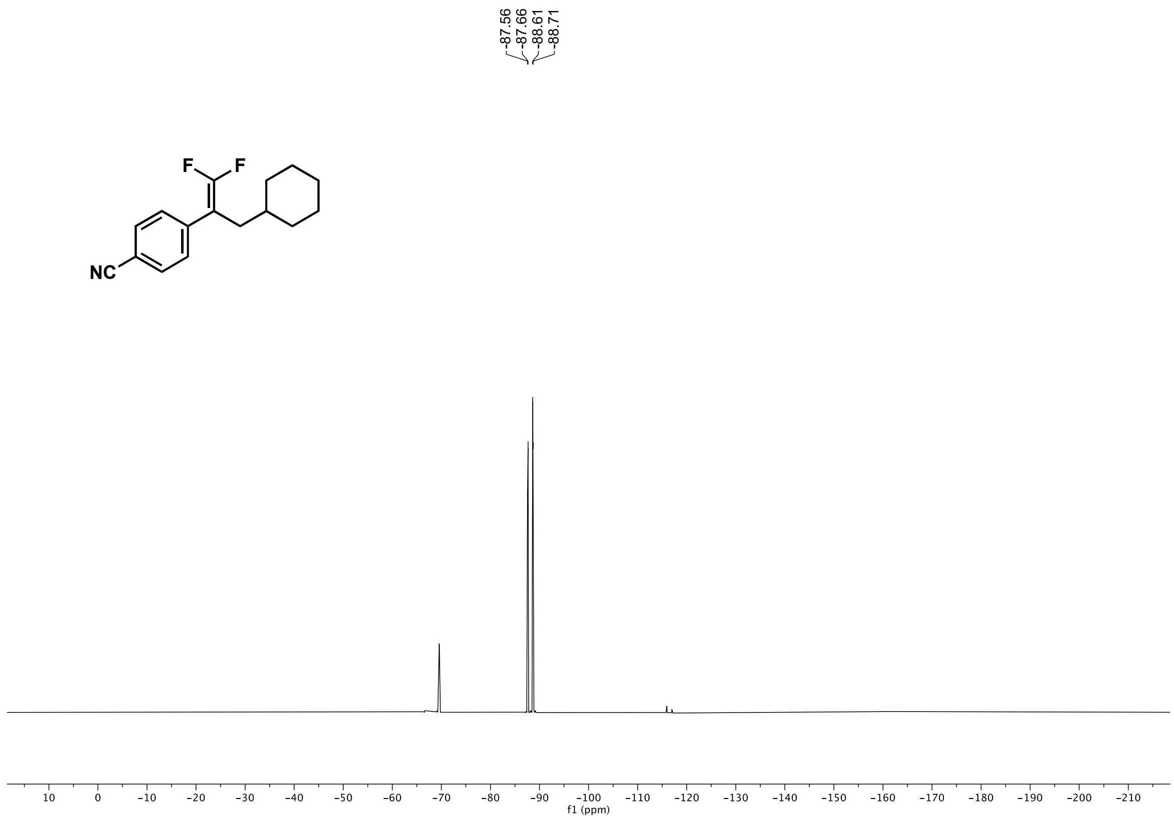

Supplementary Figure 87. ^{19}F NMR Spectra of product **29**

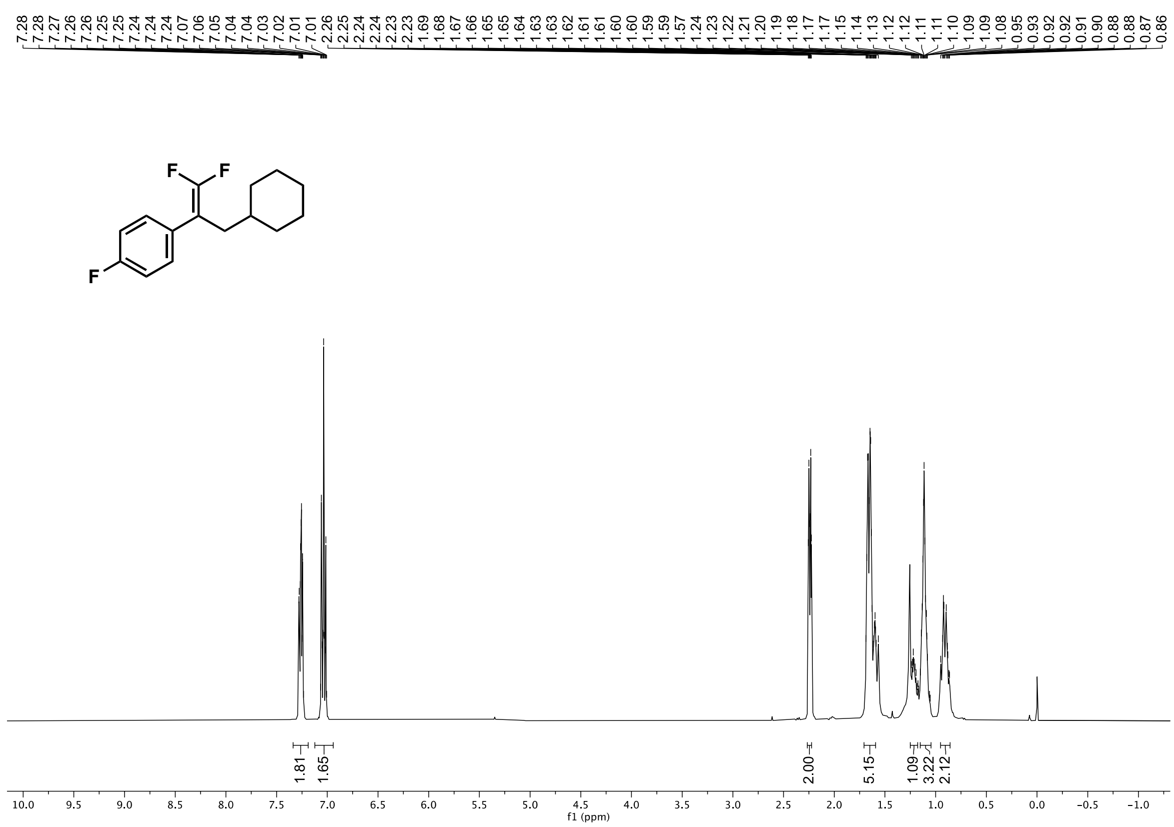

Supplementary Figure 88. ^{1}H NMR Spectra of product **30**

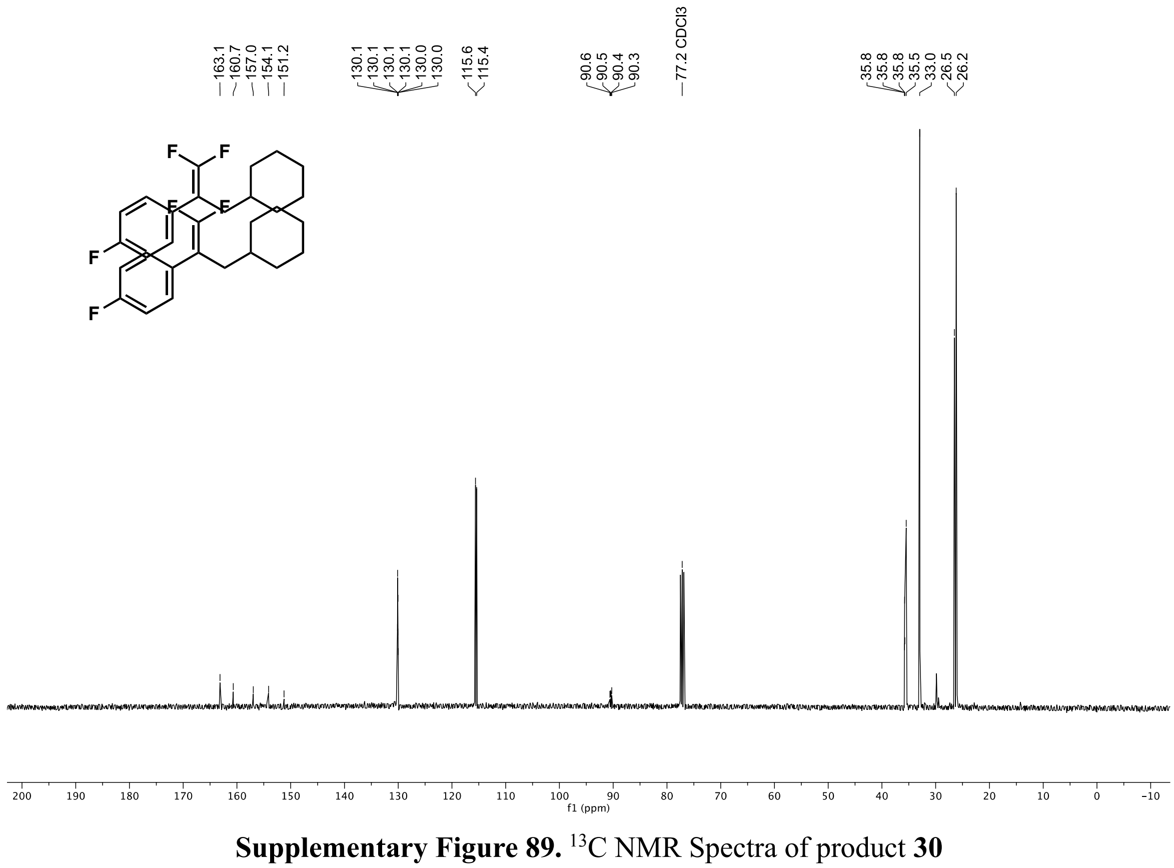

Supplementary Figure 89. ^{13}C NMR Spectra of product **30**

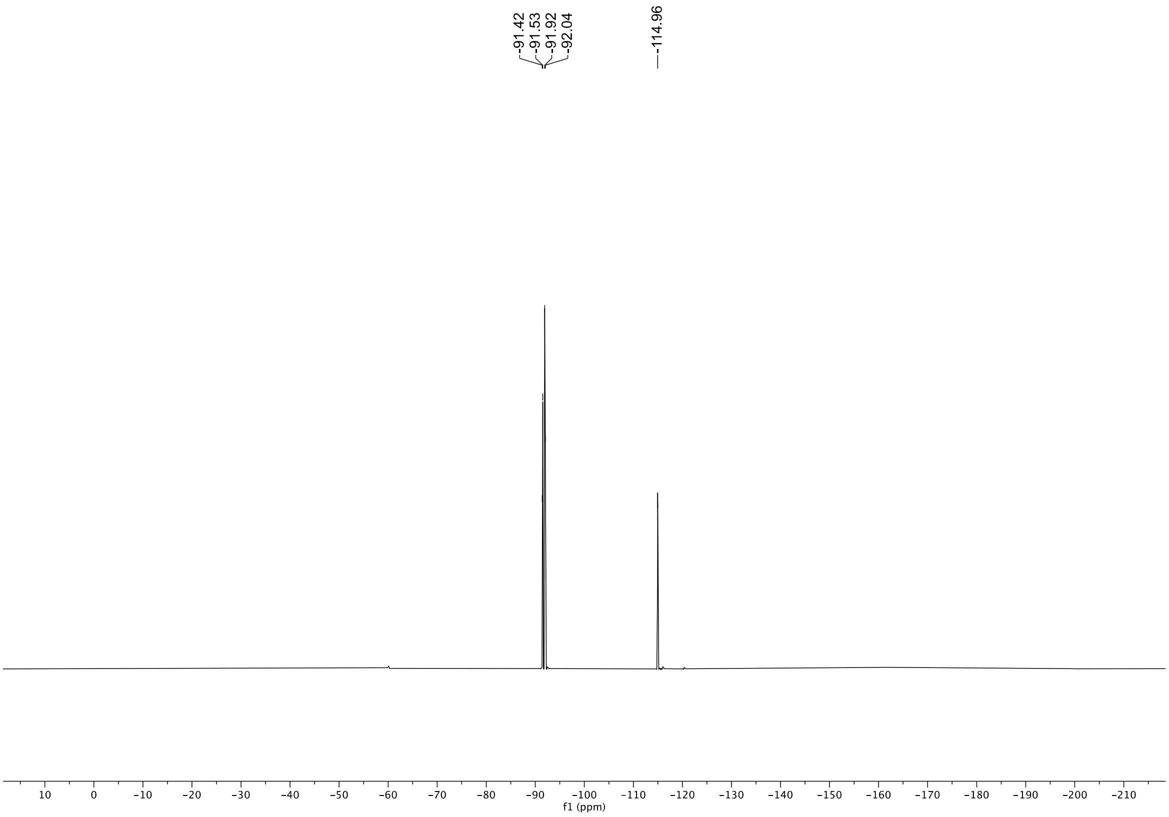

Supplementary Figure 90. ^{19}F NMR Spectra of product **30**

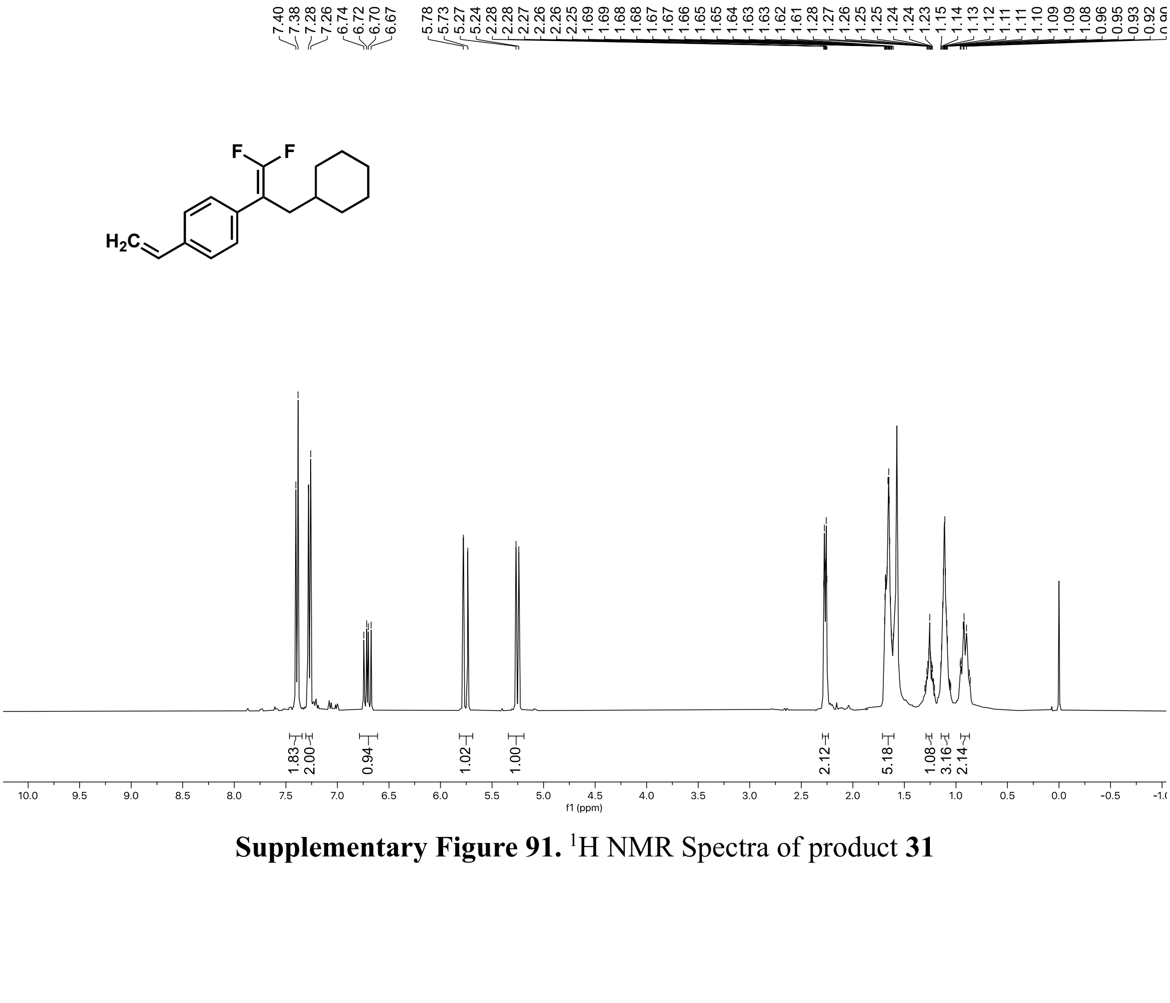

Supplementary Figure 91. ^{1}H NMR Spectra of product **31**

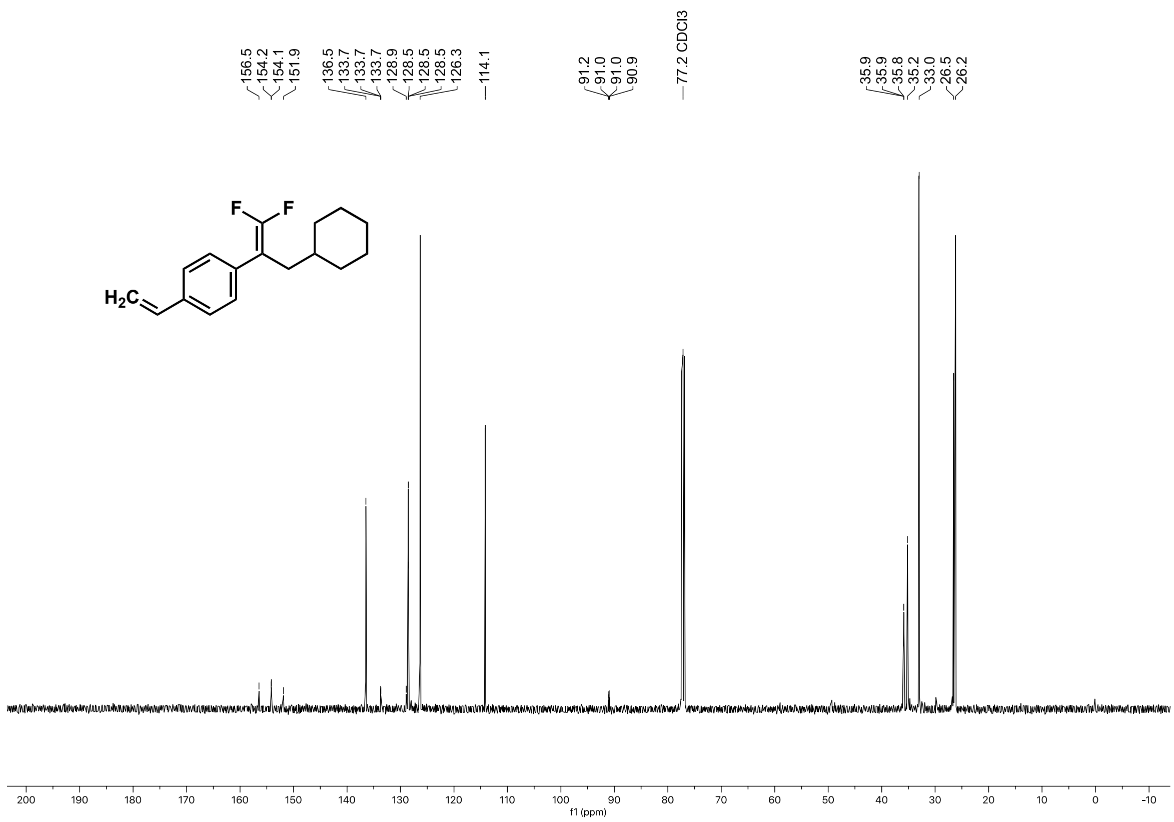

Supplementary Figure 92. ^{13}C NMR Spectra of product **31**

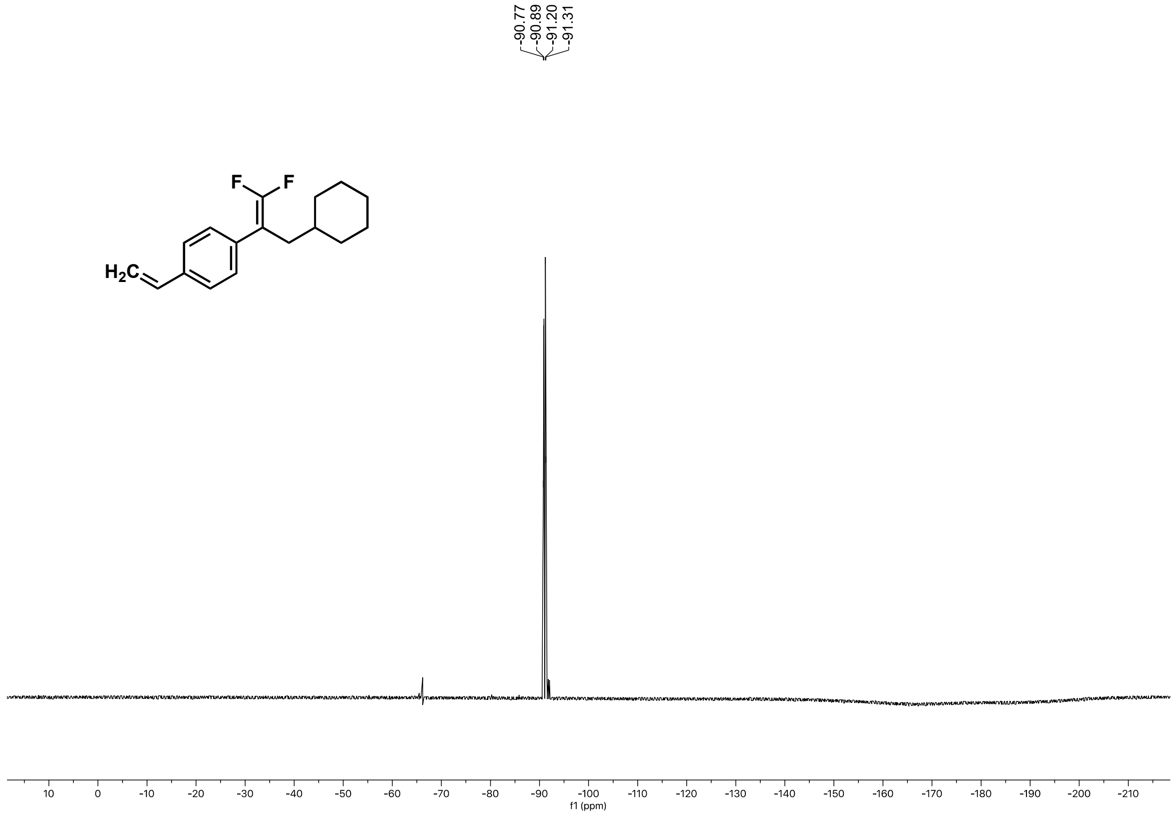

Supplementary Figure 93. ^{19}F NMR Spectra of product **31**

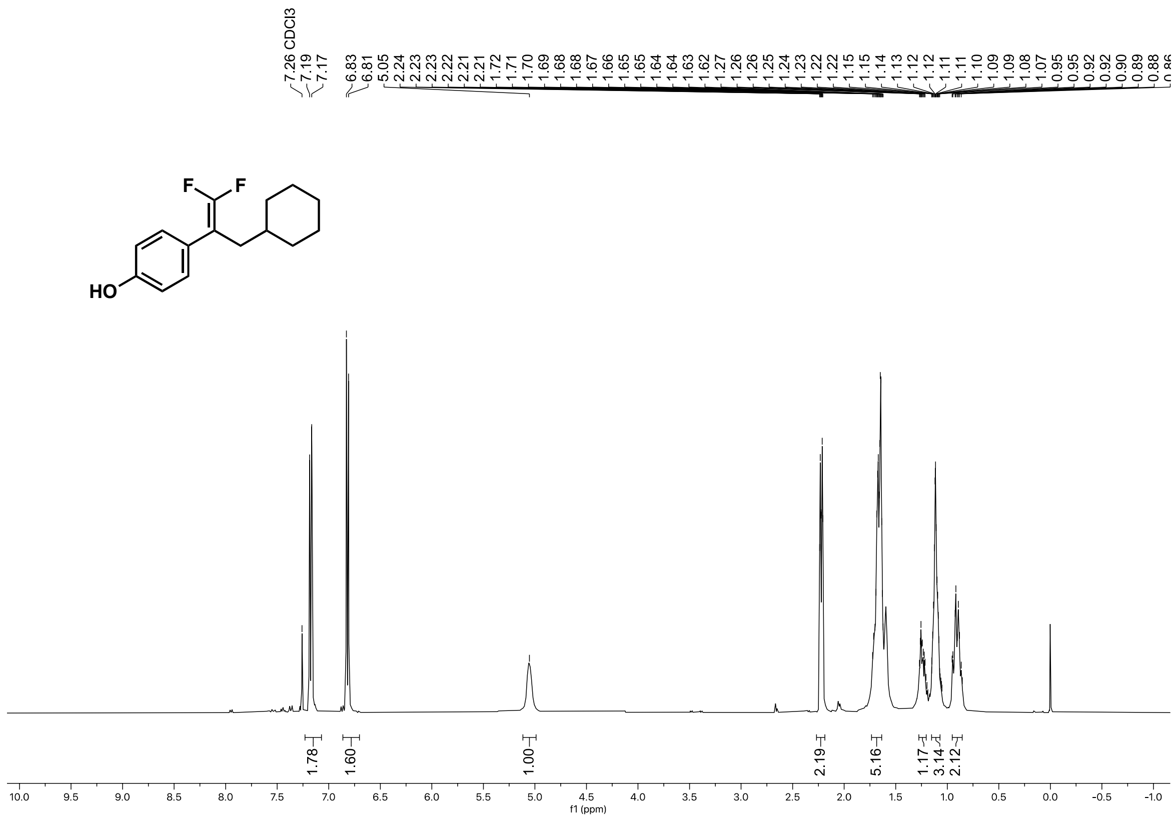

Supplementary Figure 94. ^{1}H NMR Spectra of product **32**

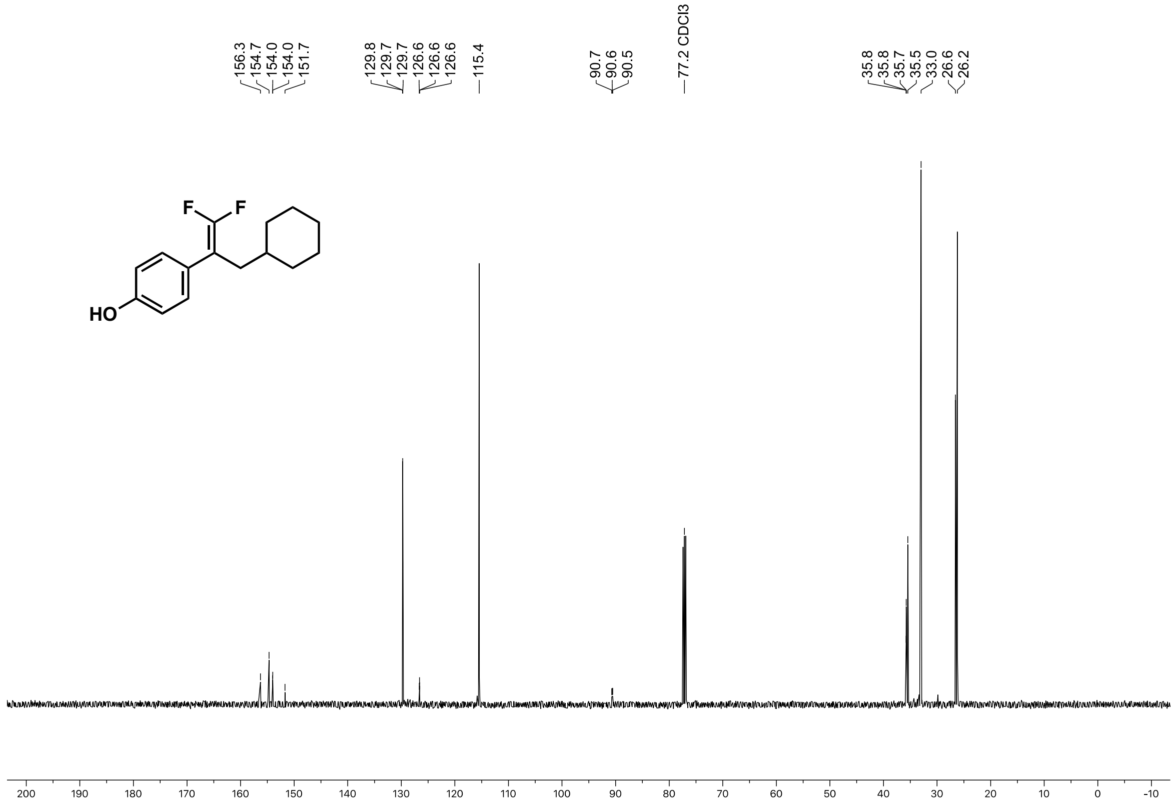

Supplementary Figure 95 ^{13}C NMR Spectra of product **32**

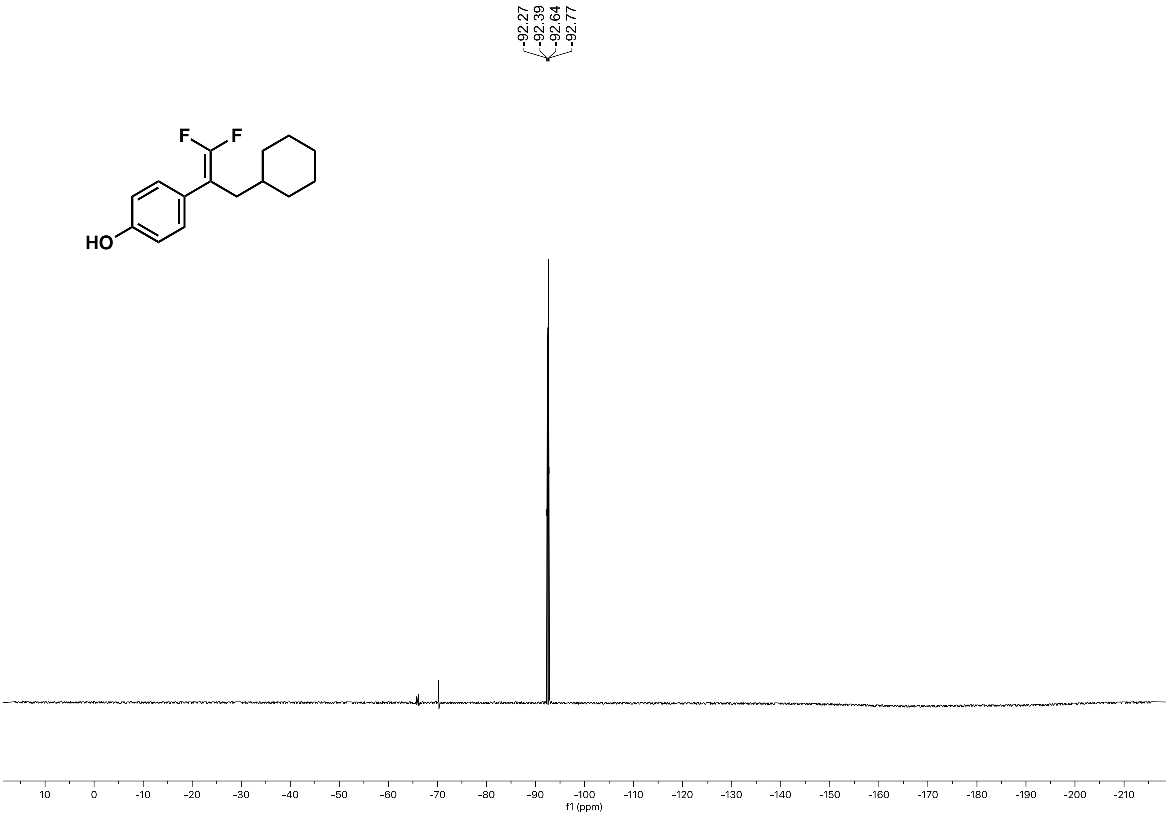

Supplementary Figure 96. ^{19}F NMR Spectra of product **32**

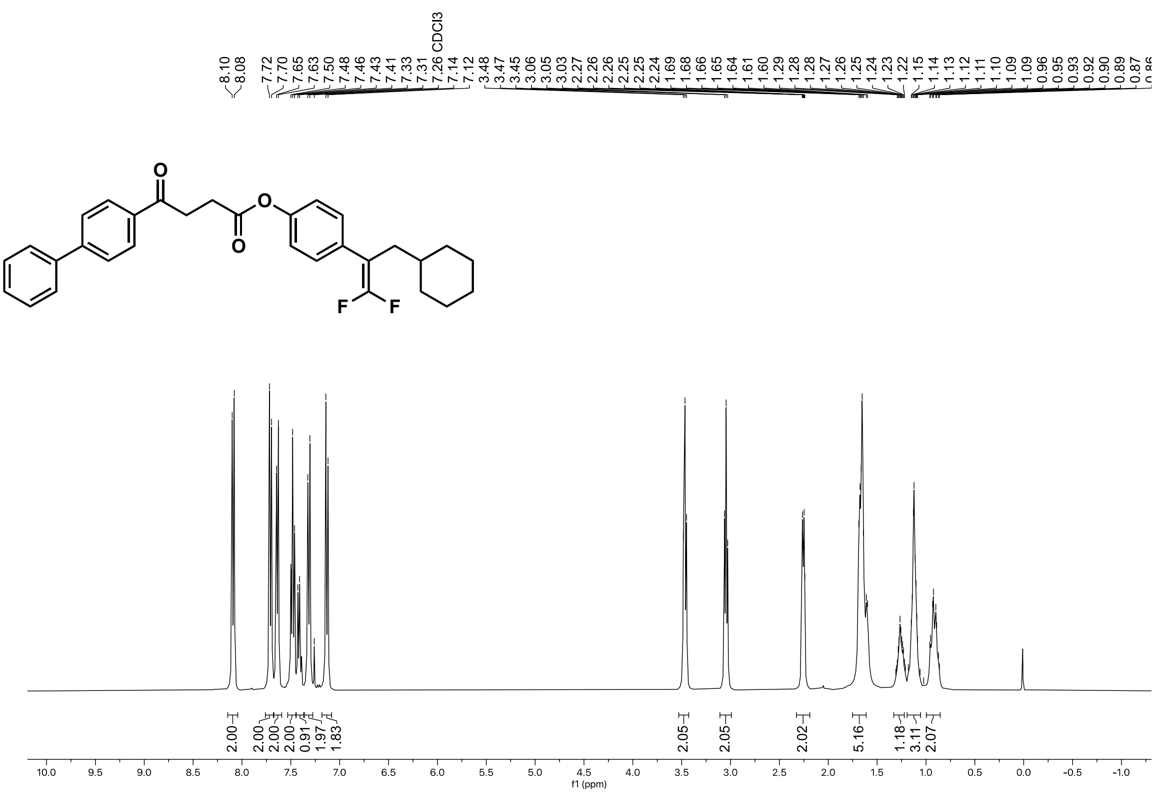

Supplementary Figure 97. ^{1}H NMR Spectra of product **33**

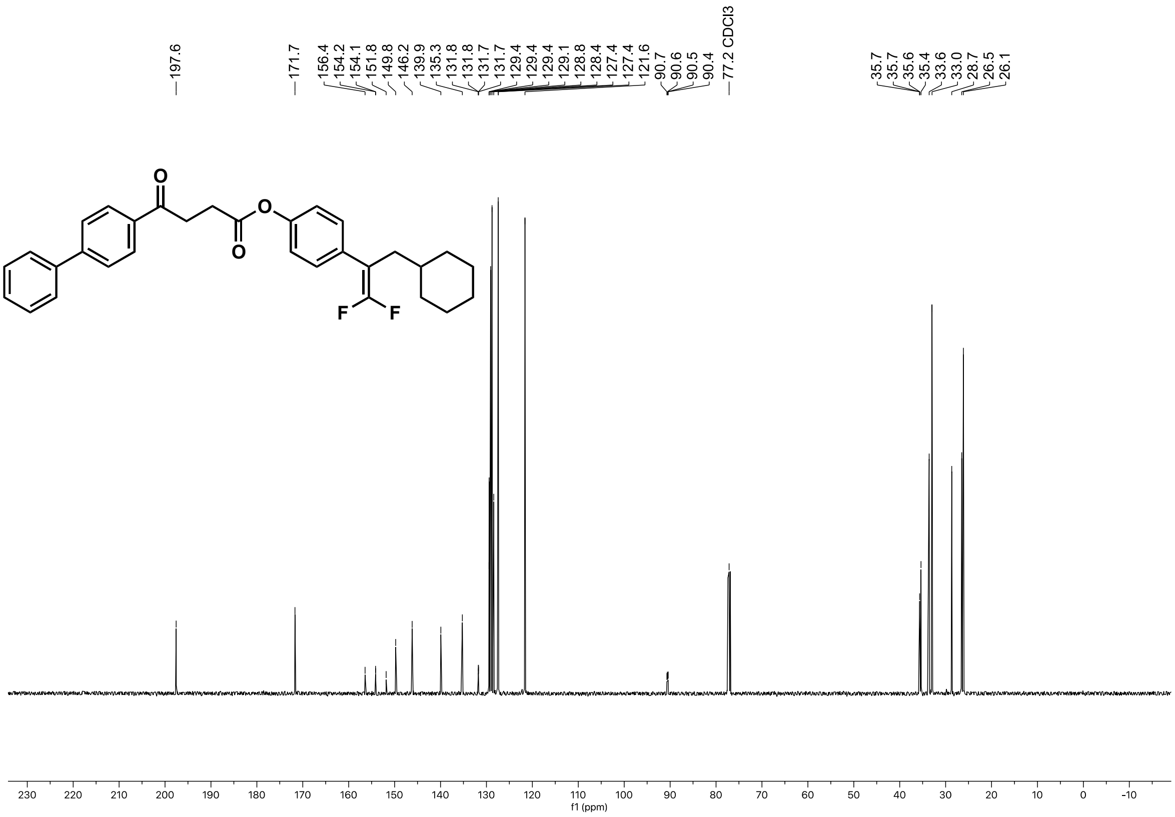

Supplementary Figure 98. ^{13}C NMR Spectra of product **33**

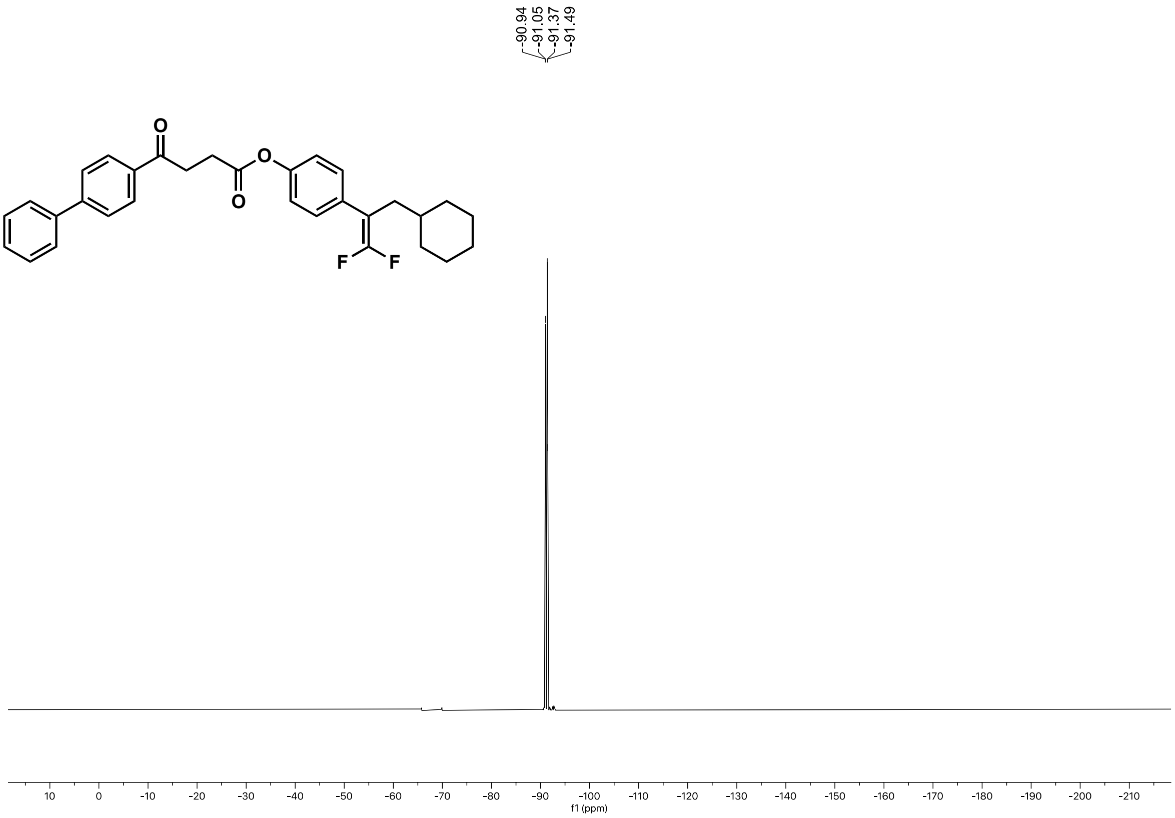

Supplementary Figure 99. ^{19}F NMR Spectra of product **33**

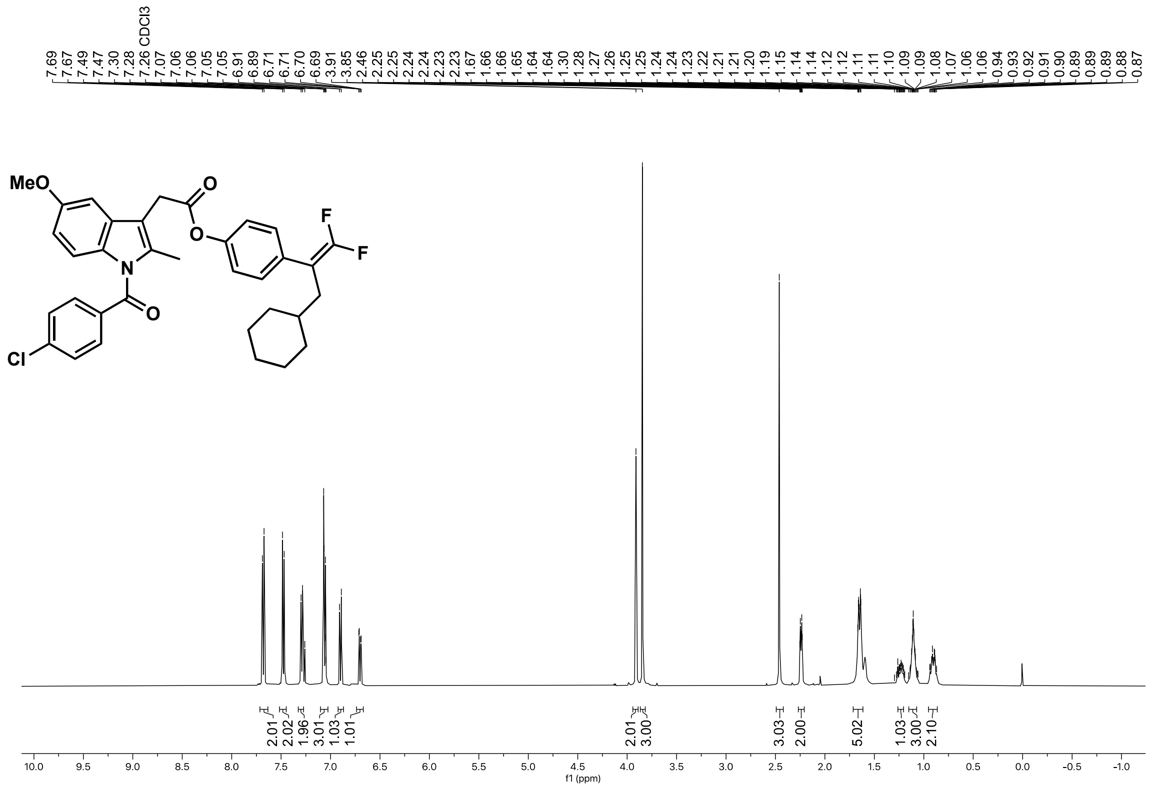

Supplementary Figure 100. ^{1}H NMR Spectra of product **34**

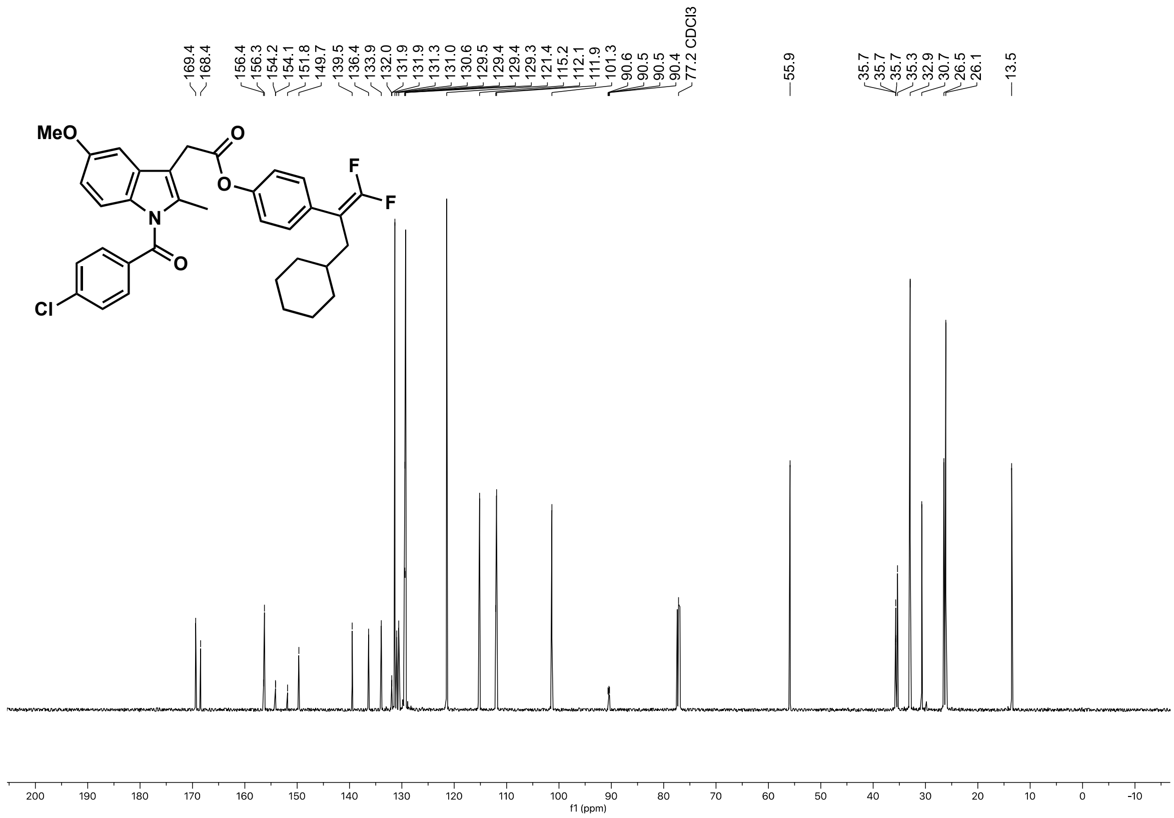

Supplementary Figure 101. ^{13}C NMR Spectra of product **34**

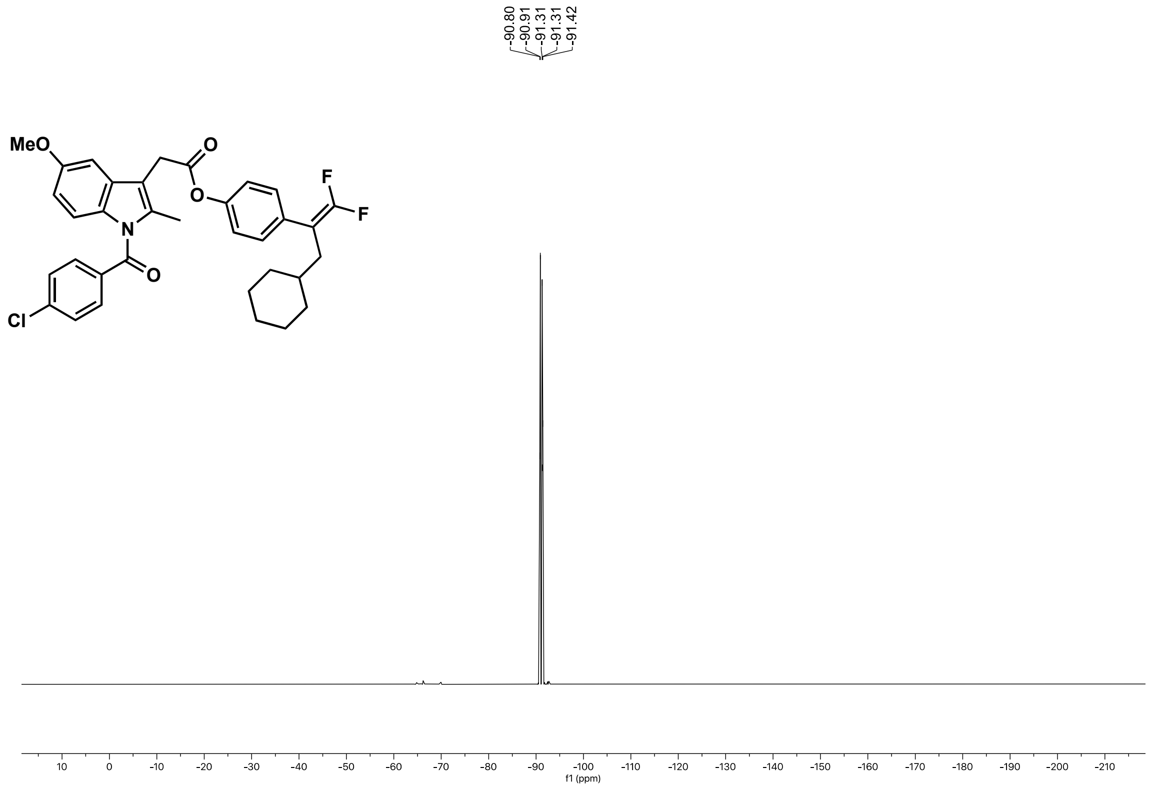

Supplementary Figure 102. ^{19}F NMR Spectra of product **34**

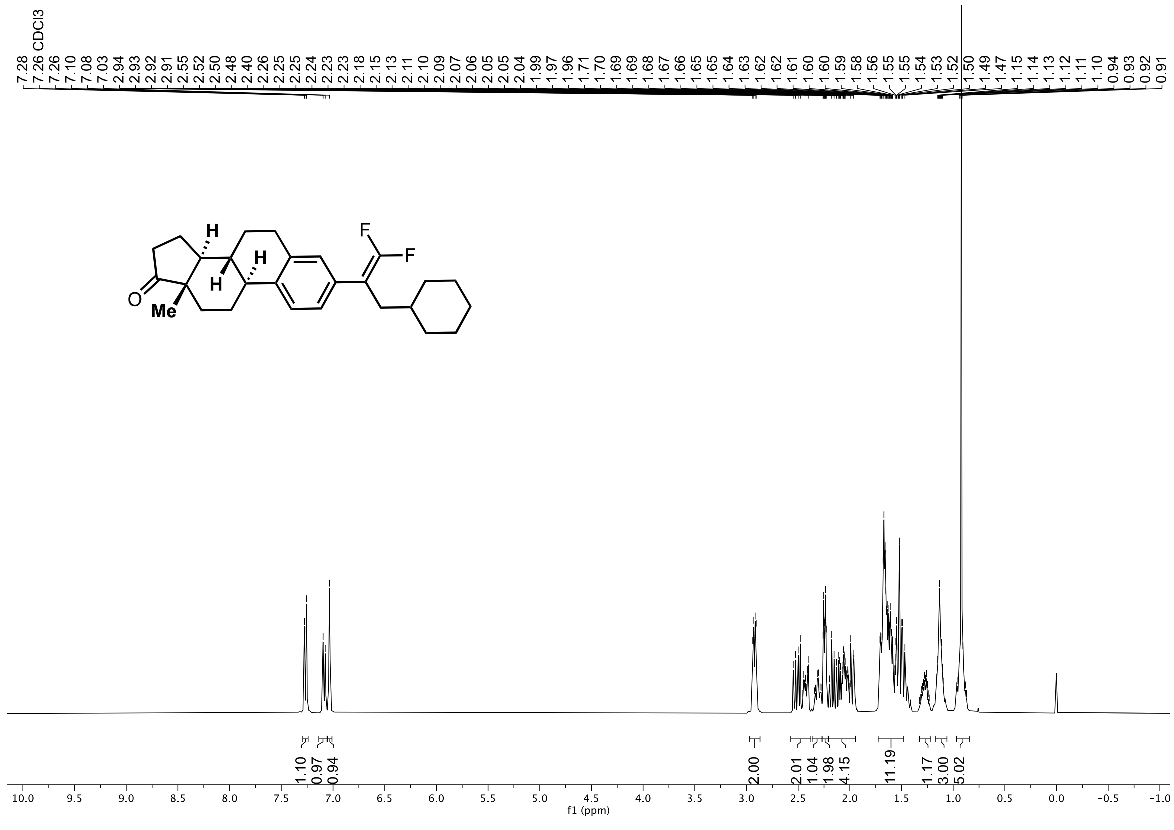

Supplementary Figure 103. ^{1}H NMR Spectra of product **35**

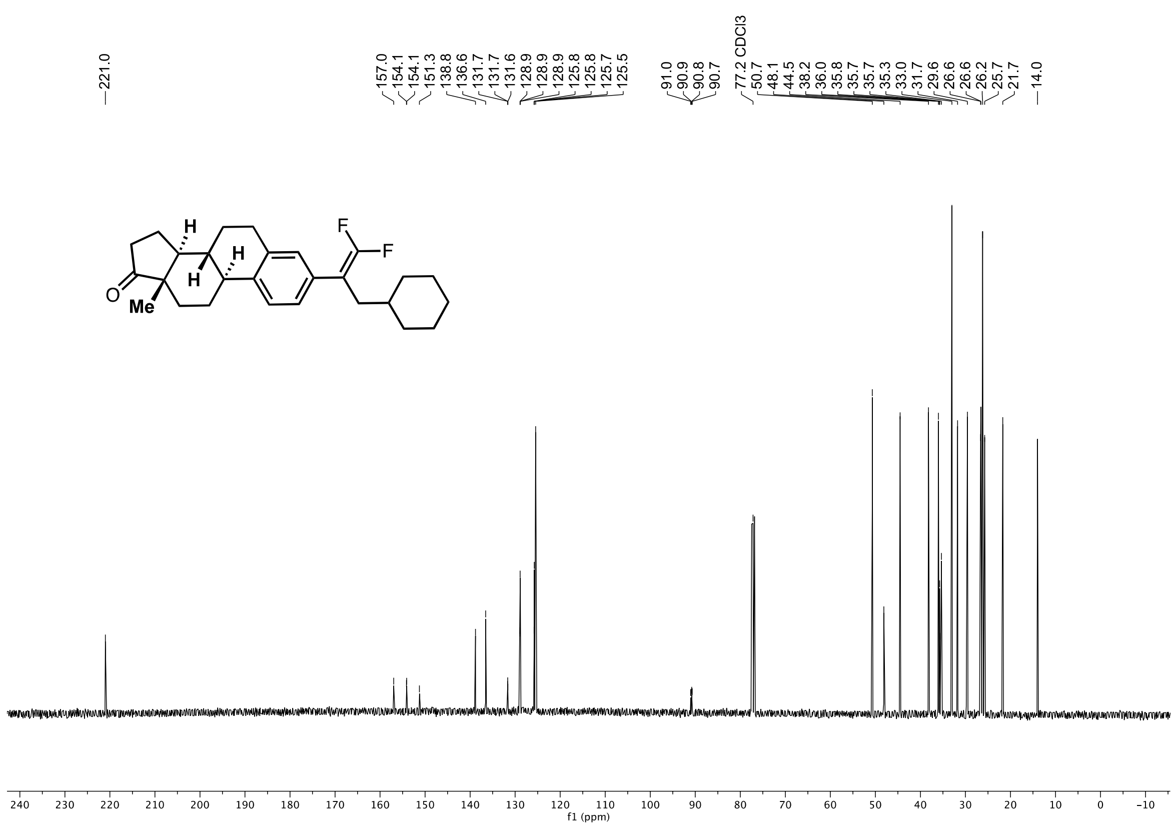

Supplementary Figure 104. ^{13}C NMR Spectra of product **35**

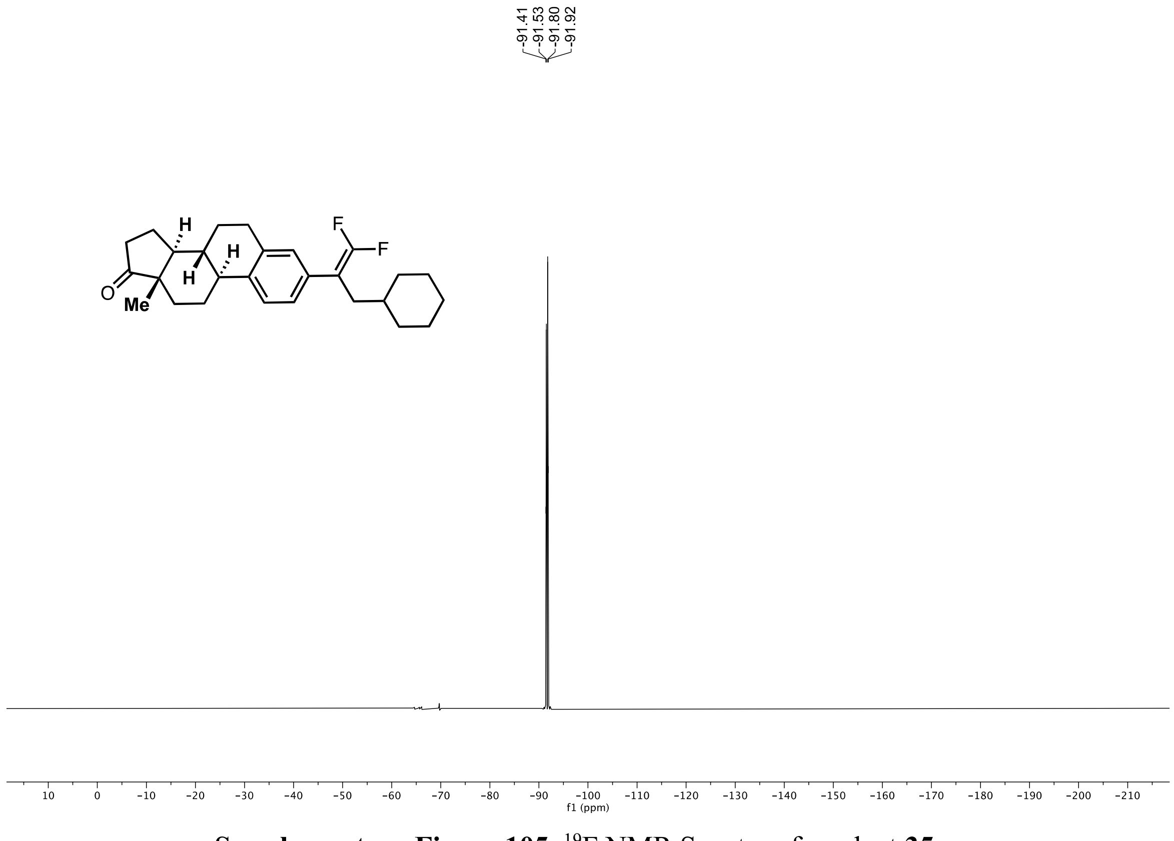

Supplementary Figure 105. ^{19}F NMR Spectra of product **35**

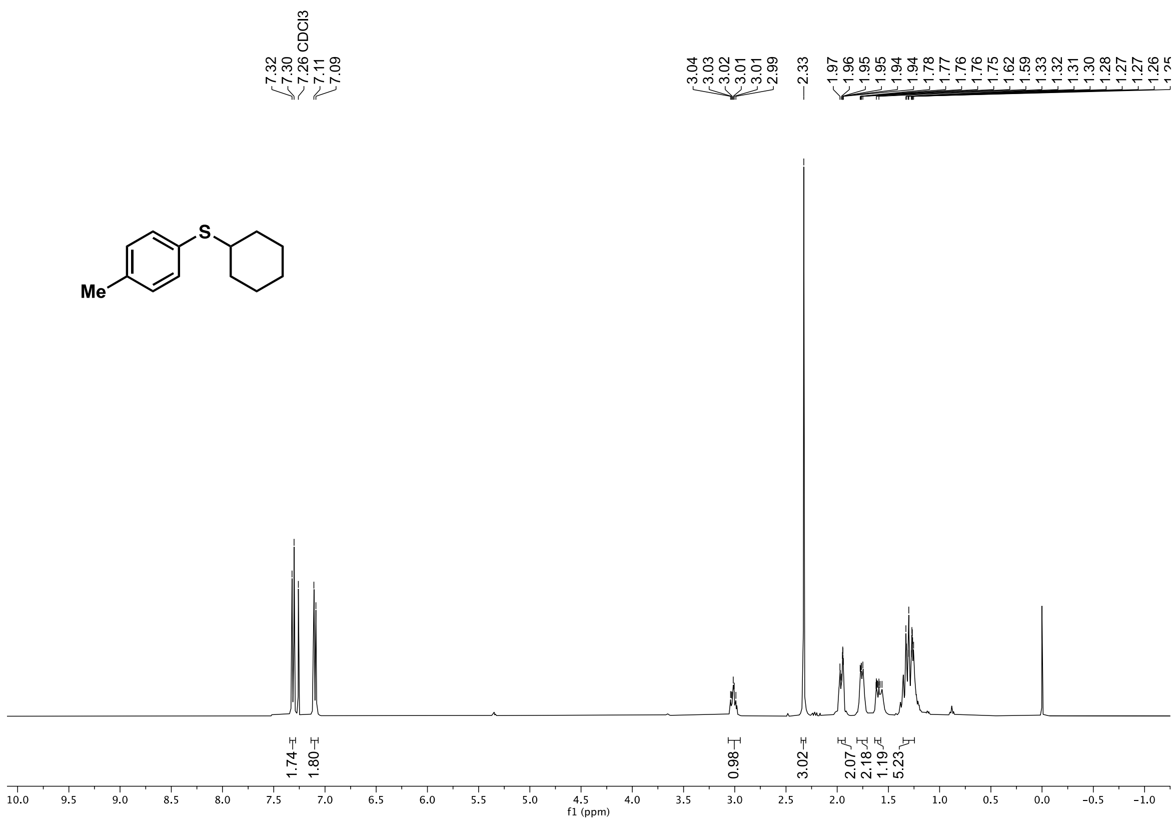

Supplementary Figure 106. ^{1}H NMR Spectra of product **36**

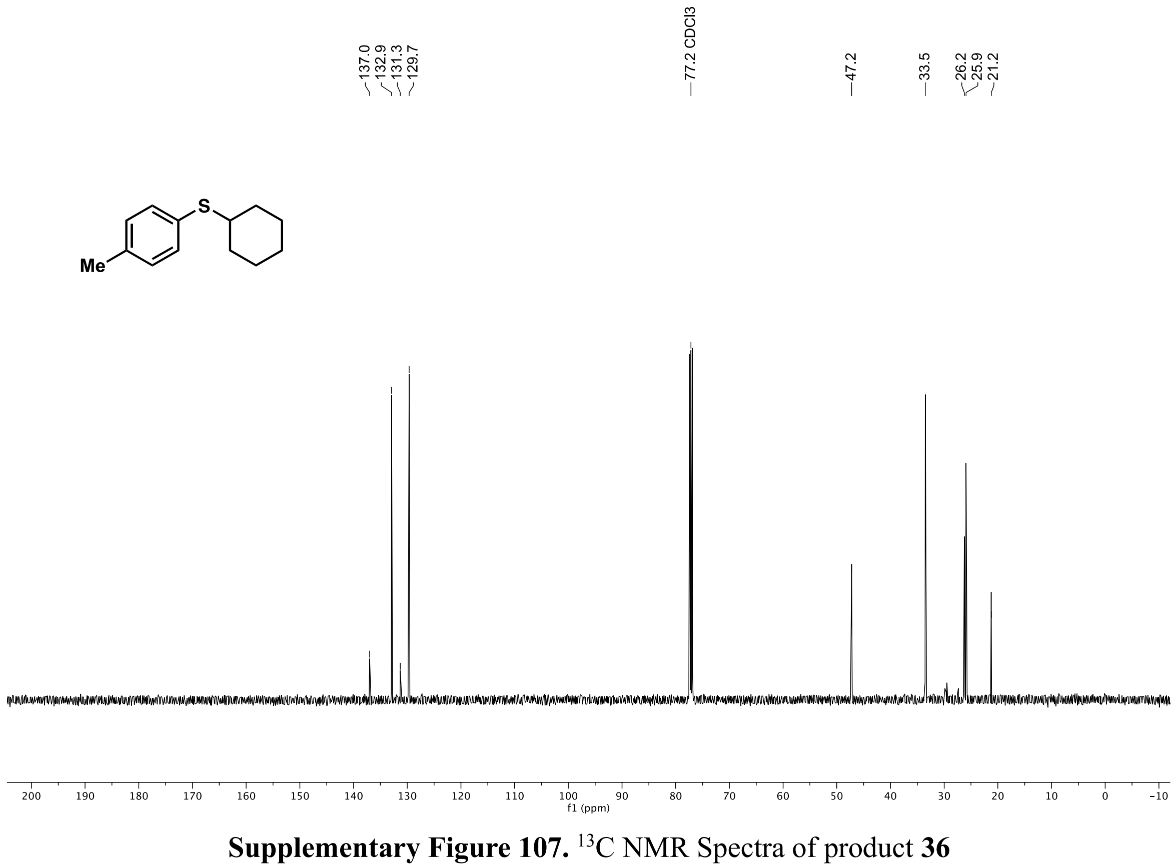

Supplementary Figure 107. ^{13}C NMR Spectra of product **36**

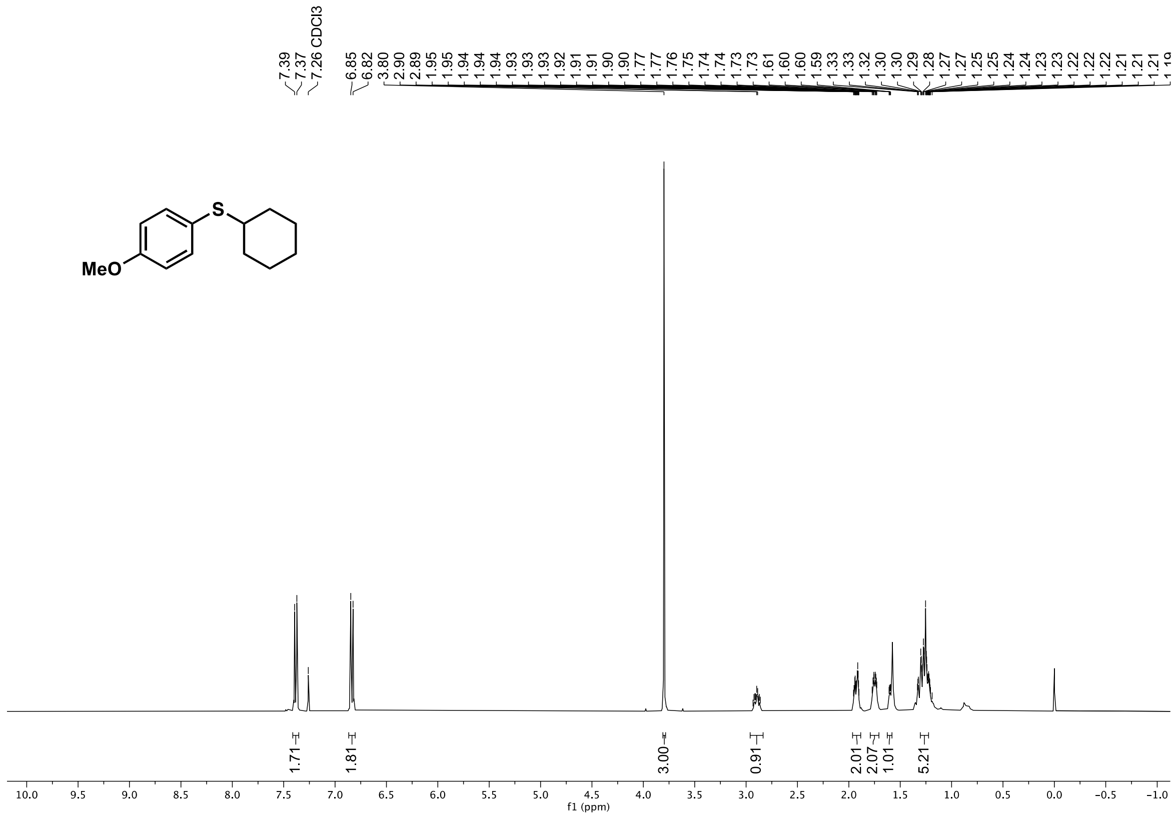

Supplementary Figure 108. ^{1}H NMR Spectra of product **36**

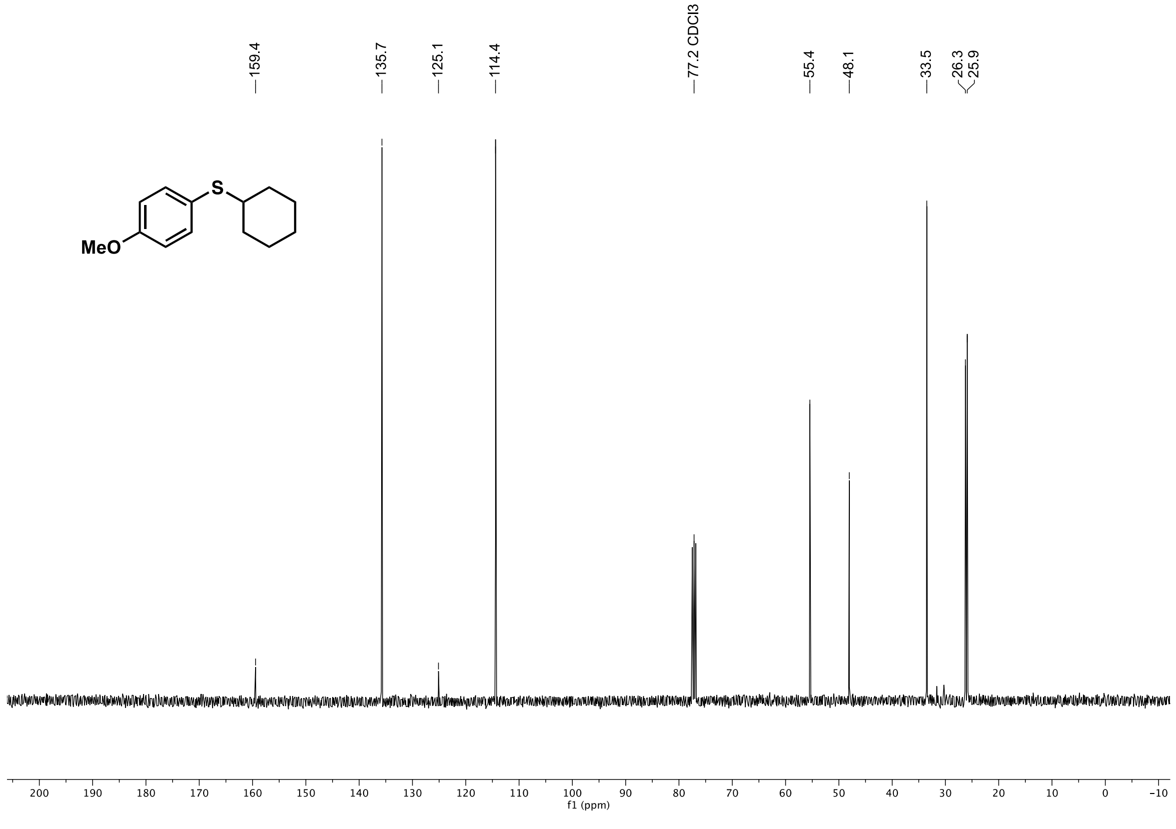

Supplementary Figure 109. ^{13}C NMR Spectra of product **37**

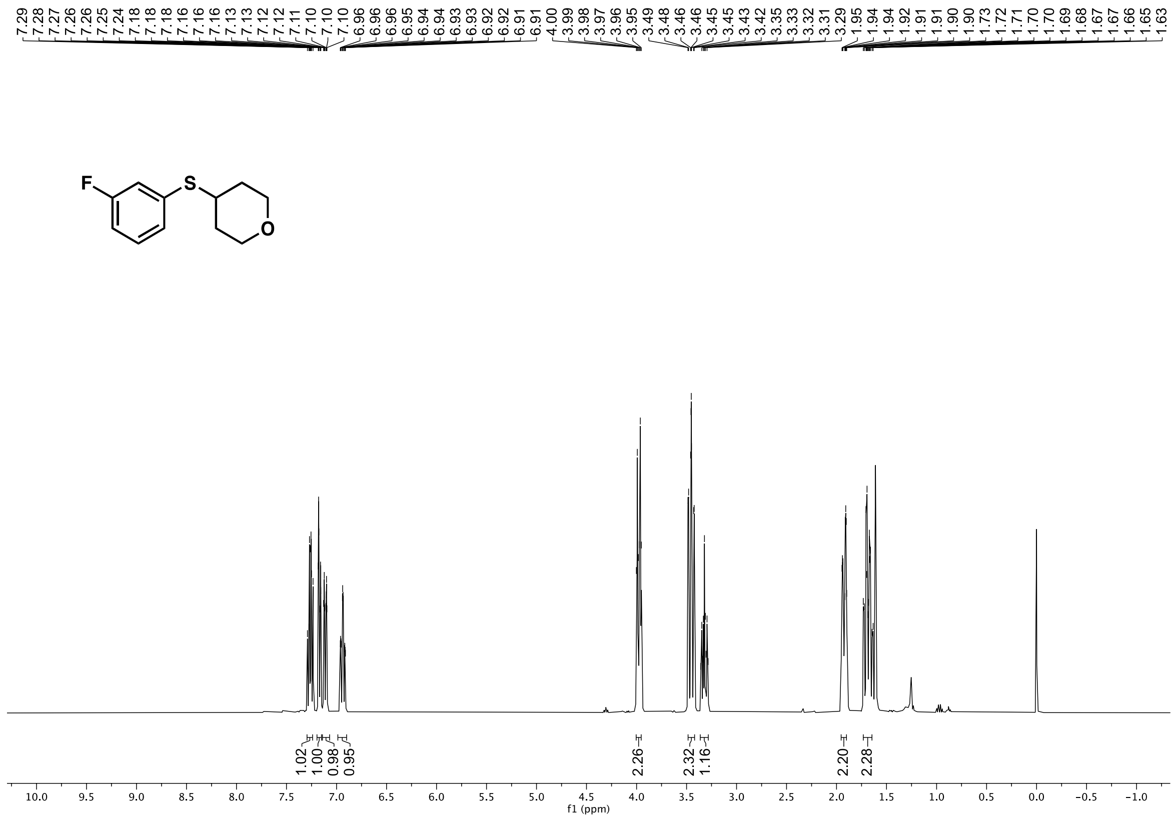

Supplementary Figure 110. ^{1}H NMR Spectra of product **38**

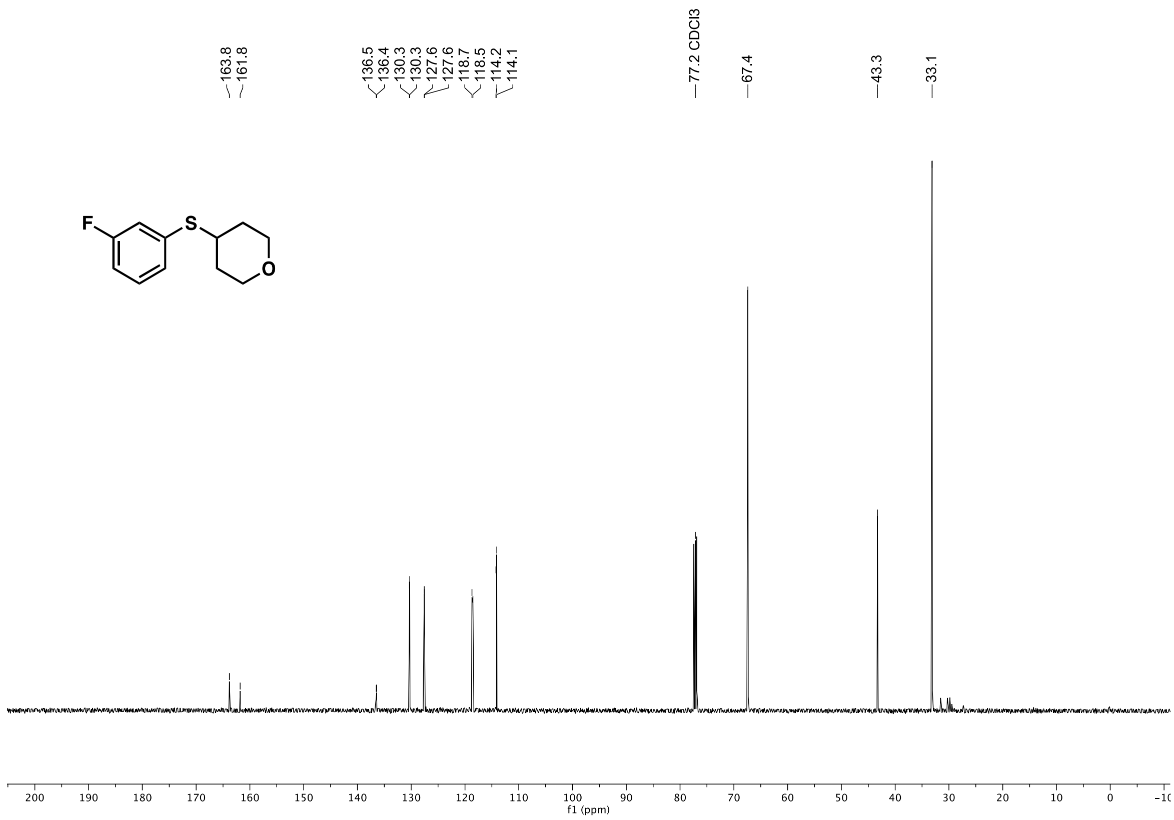

Supplementary Figure 111. ^{13}C NMR Spectra of product **38**

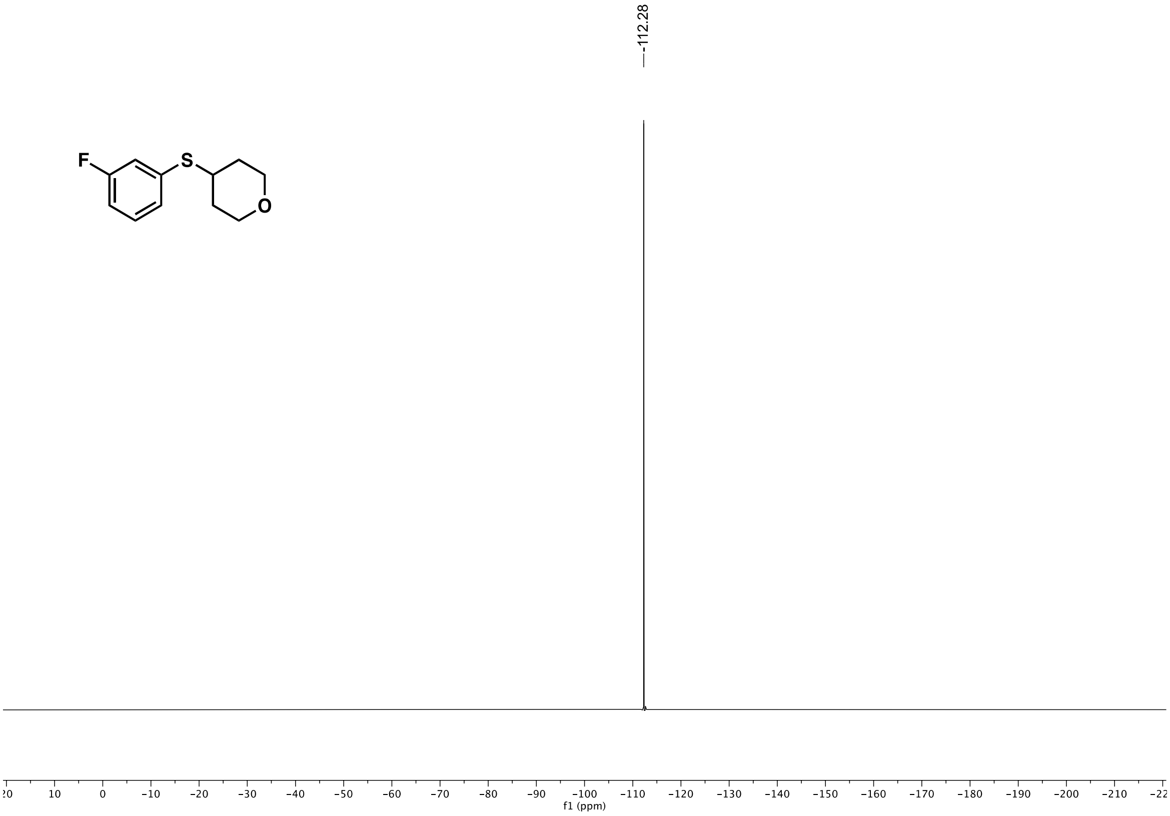

Supplementary Figure 112. ^{19}F NMR Spectra of product **38**

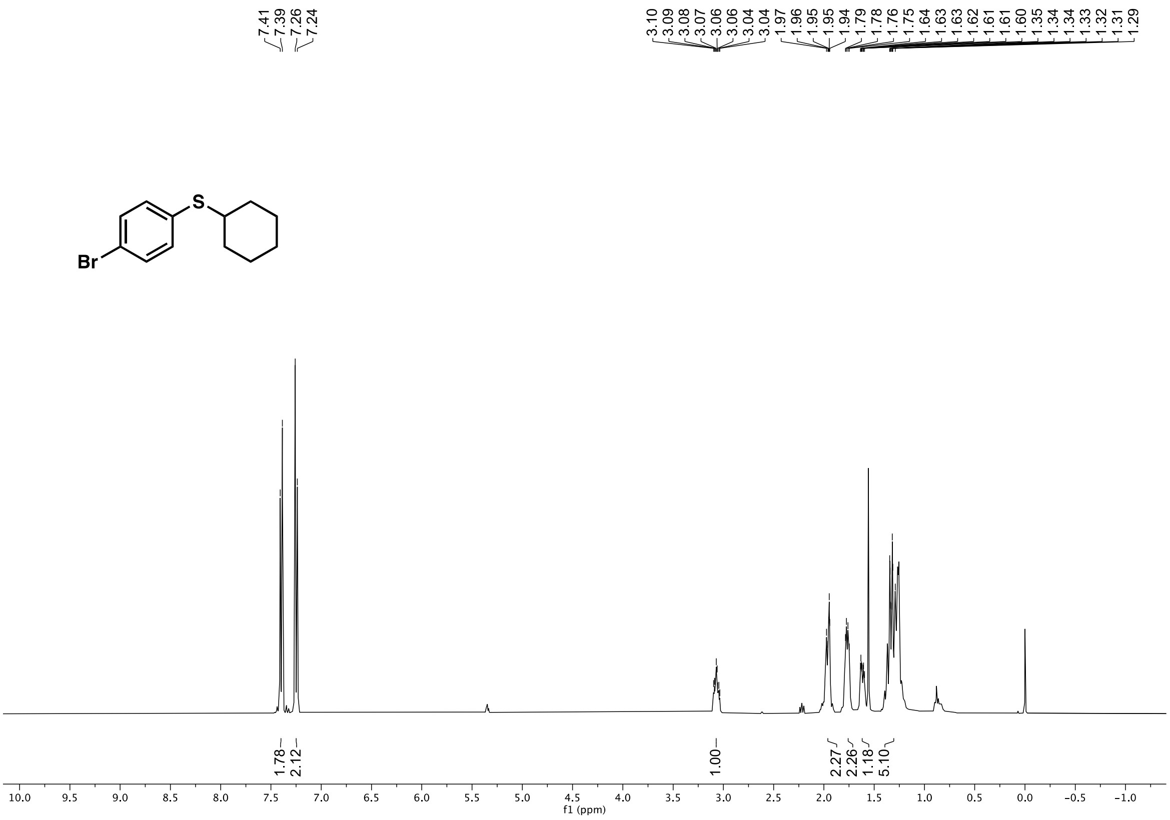

Supplementary Figure 113. ^{1}H NMR Spectra of product **39**

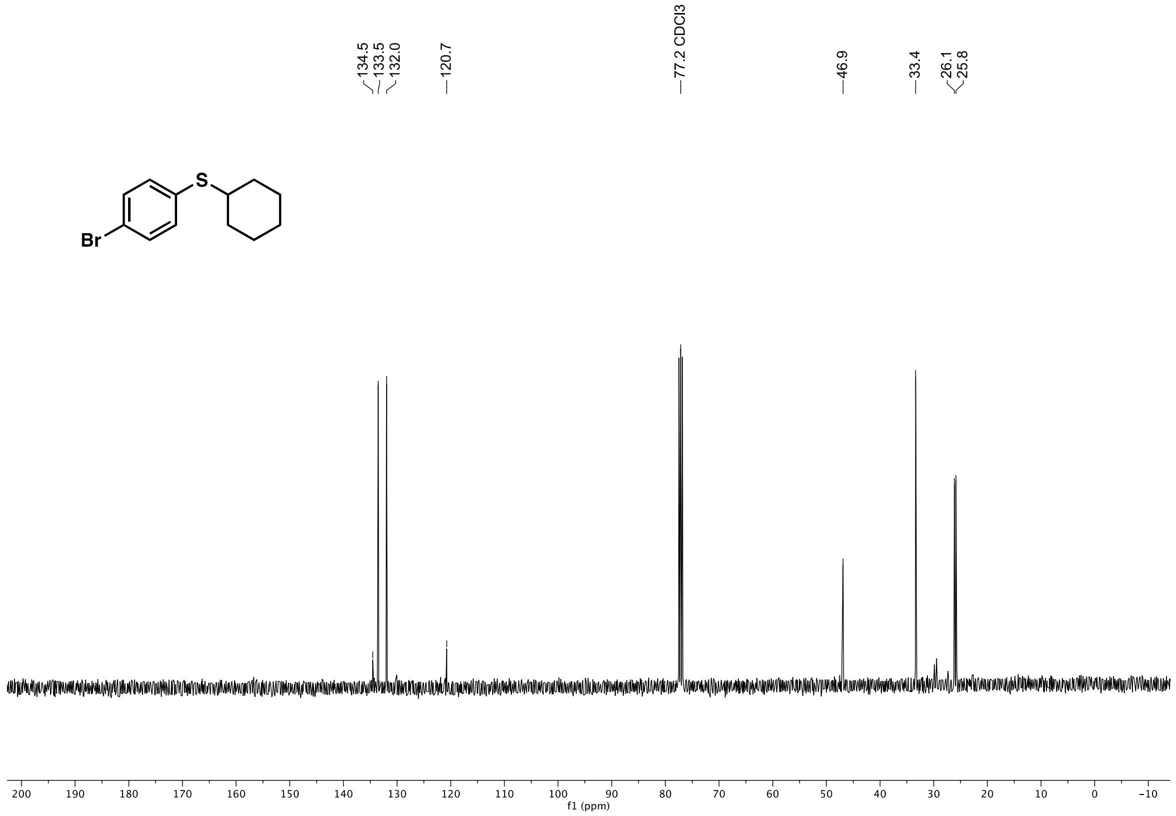

Supplementary Figure 114. ^{13}C NMR Spectra of product **39**

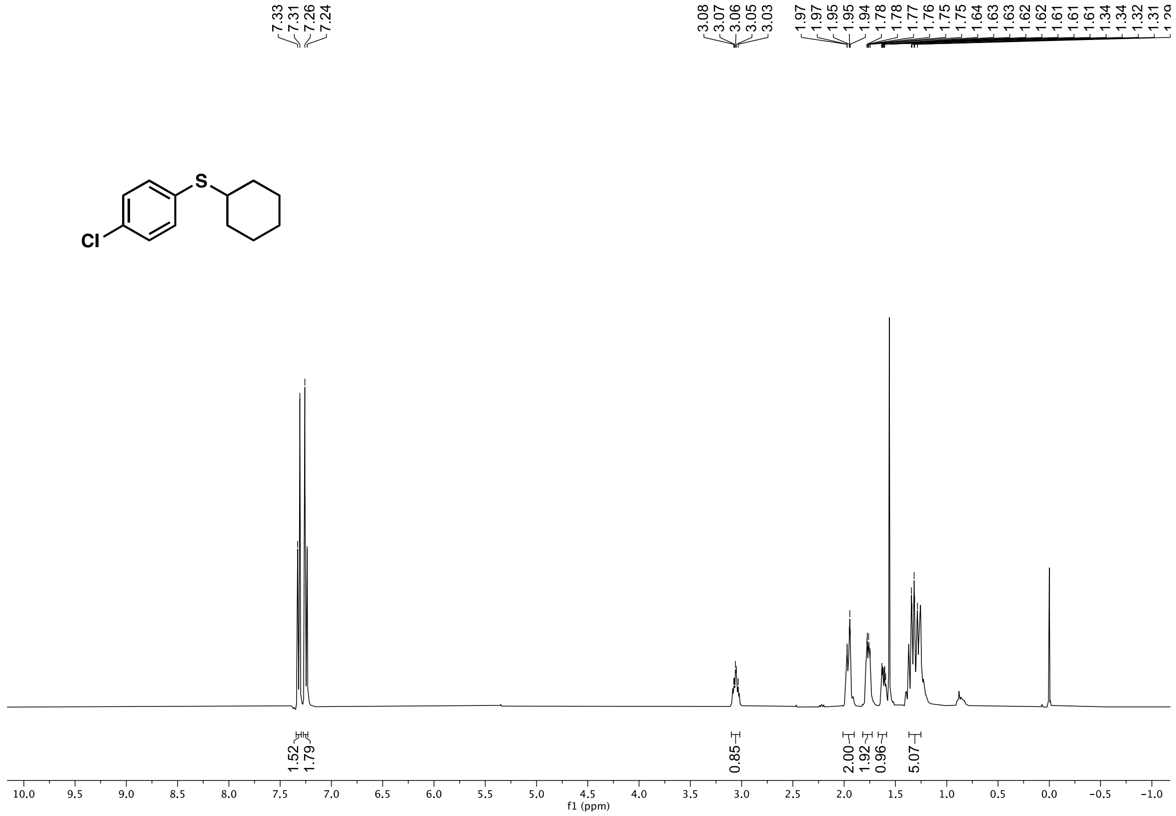

Supplementary Figure 115. ^{1}H NMR Spectra of product **40**

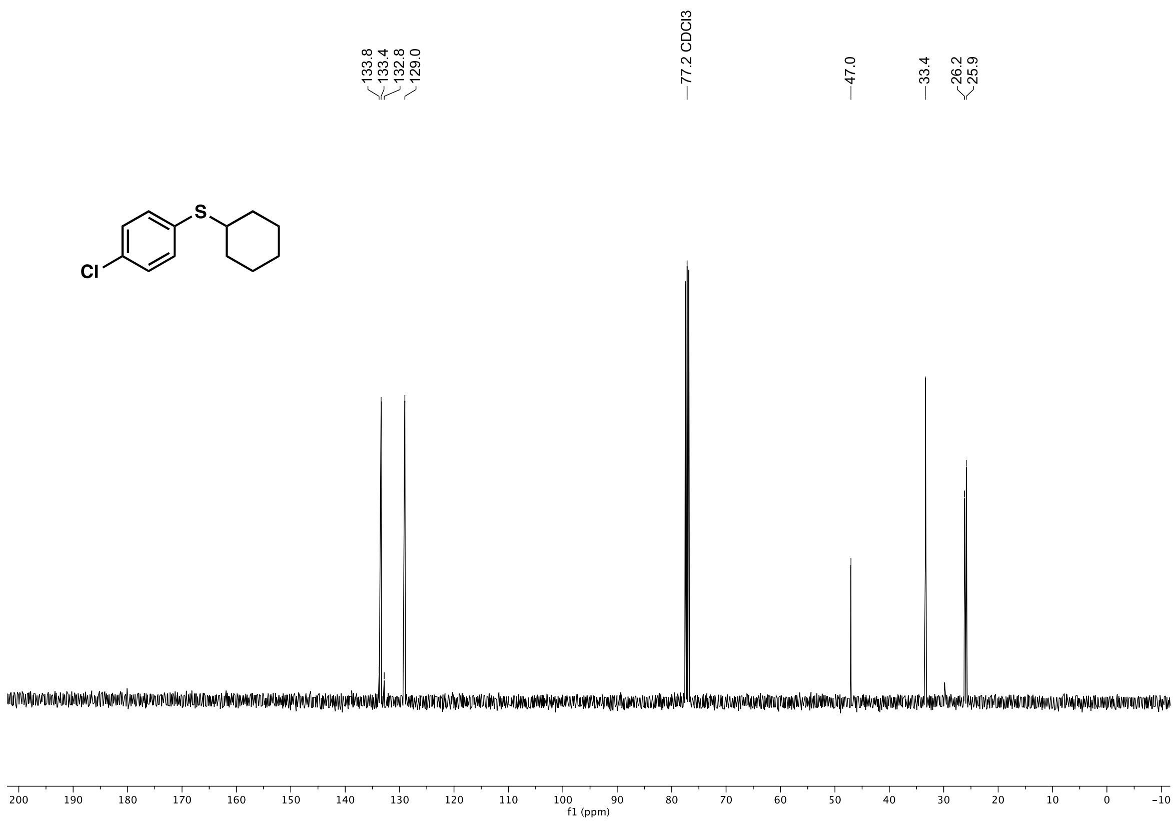

Supplementary Figure 116. ^{13}C NMR Spectra of product **40**

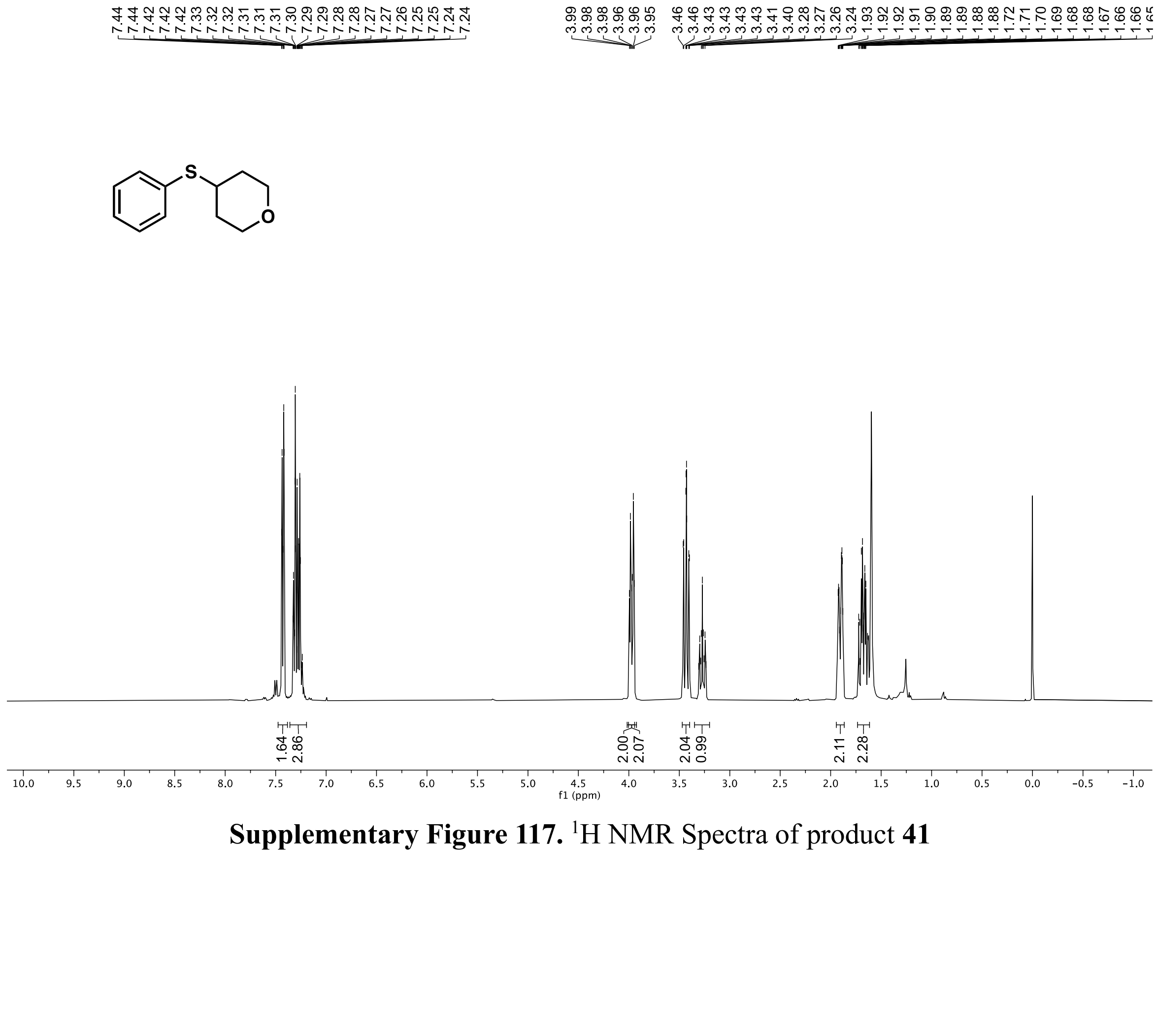

Supplementary Figure 117. ^{1}H NMR Spectra of product **41**

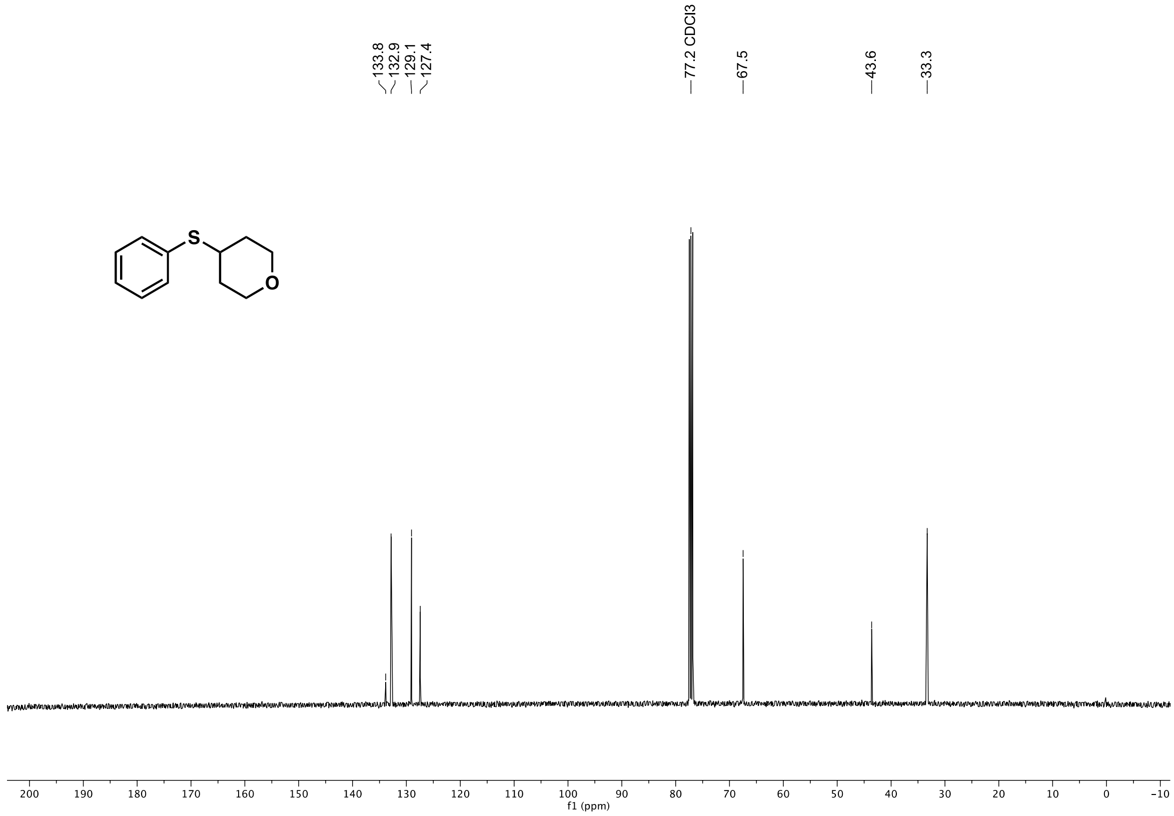

Supplementary Figure 118. ^{13}C NMR Spectra of product **41**

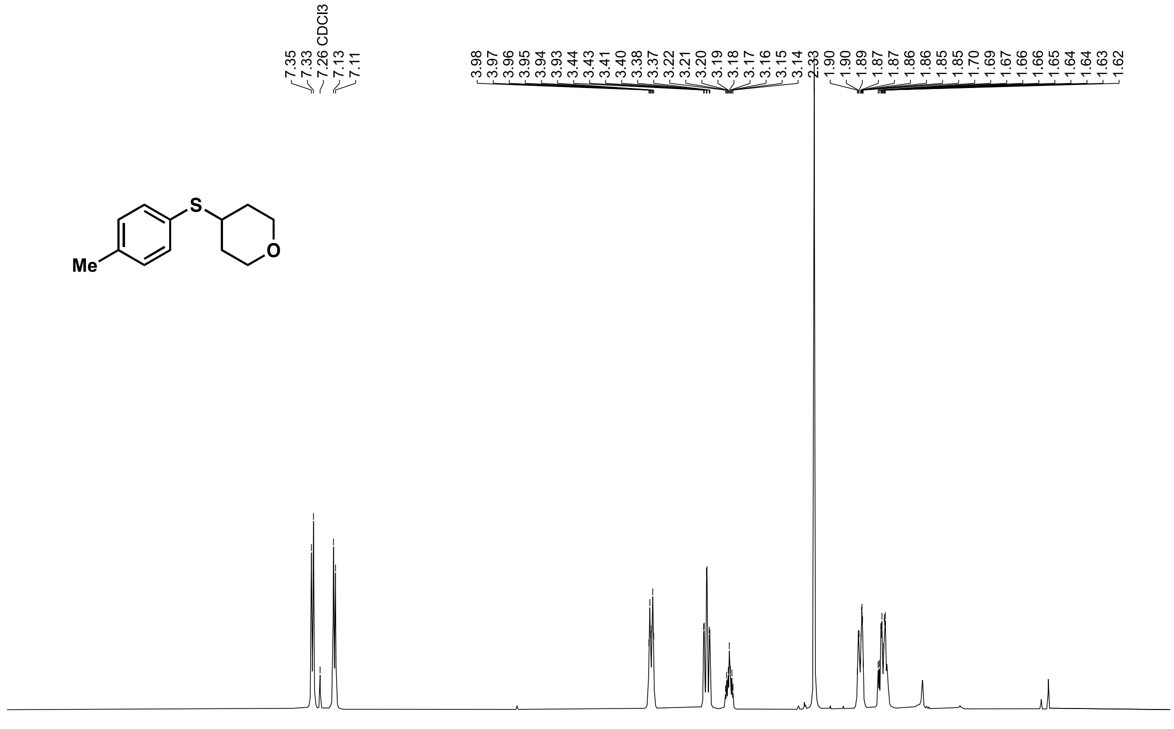

Supplementary Figure 119. ^{1}H NMR Spectra of product **42**

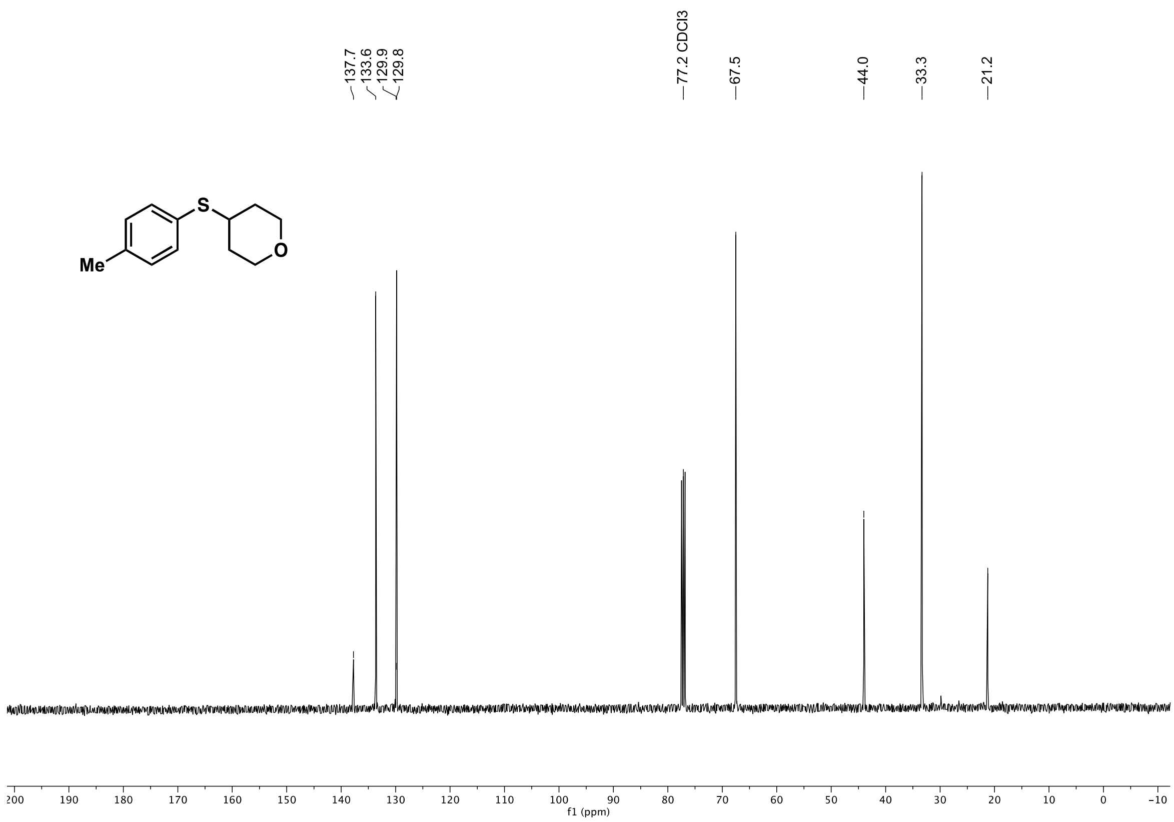

Supplementary Figure 120. ^{13}C NMR Spectra of product **42**

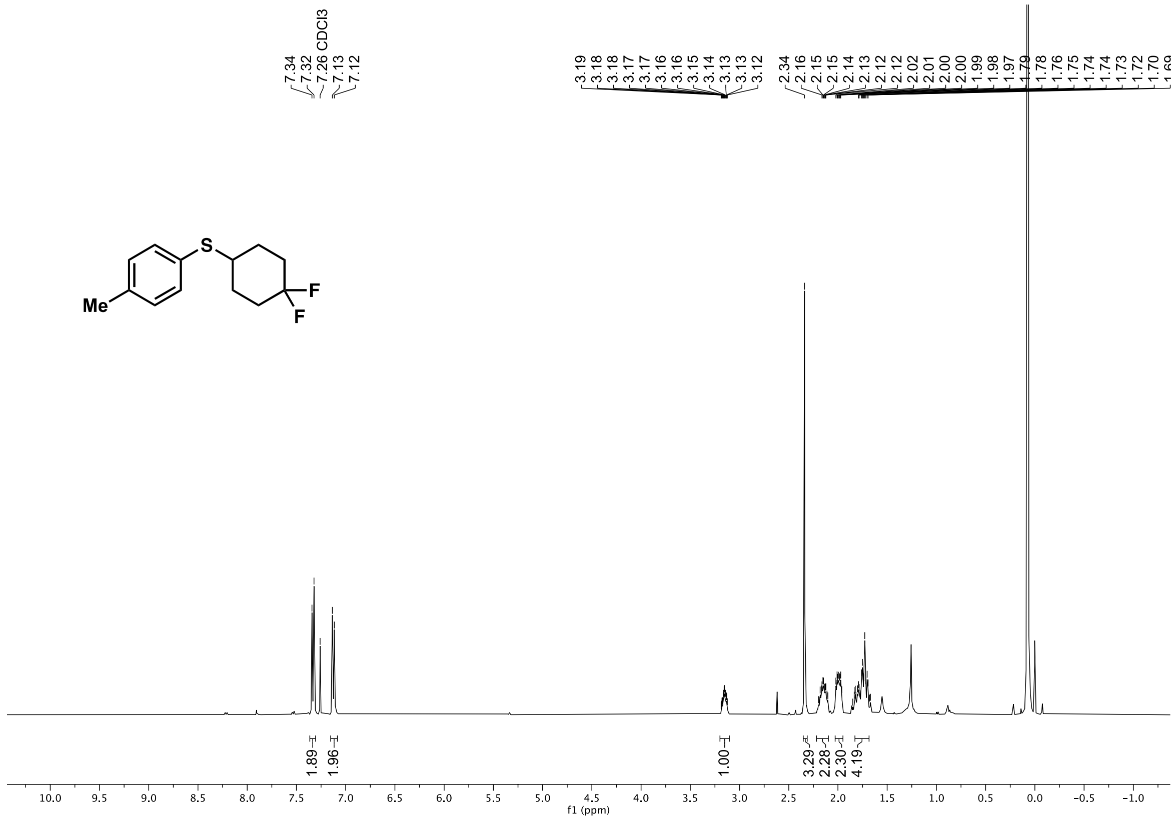

Supplementary Figure 121. ^{1}H NMR Spectra of product **43**

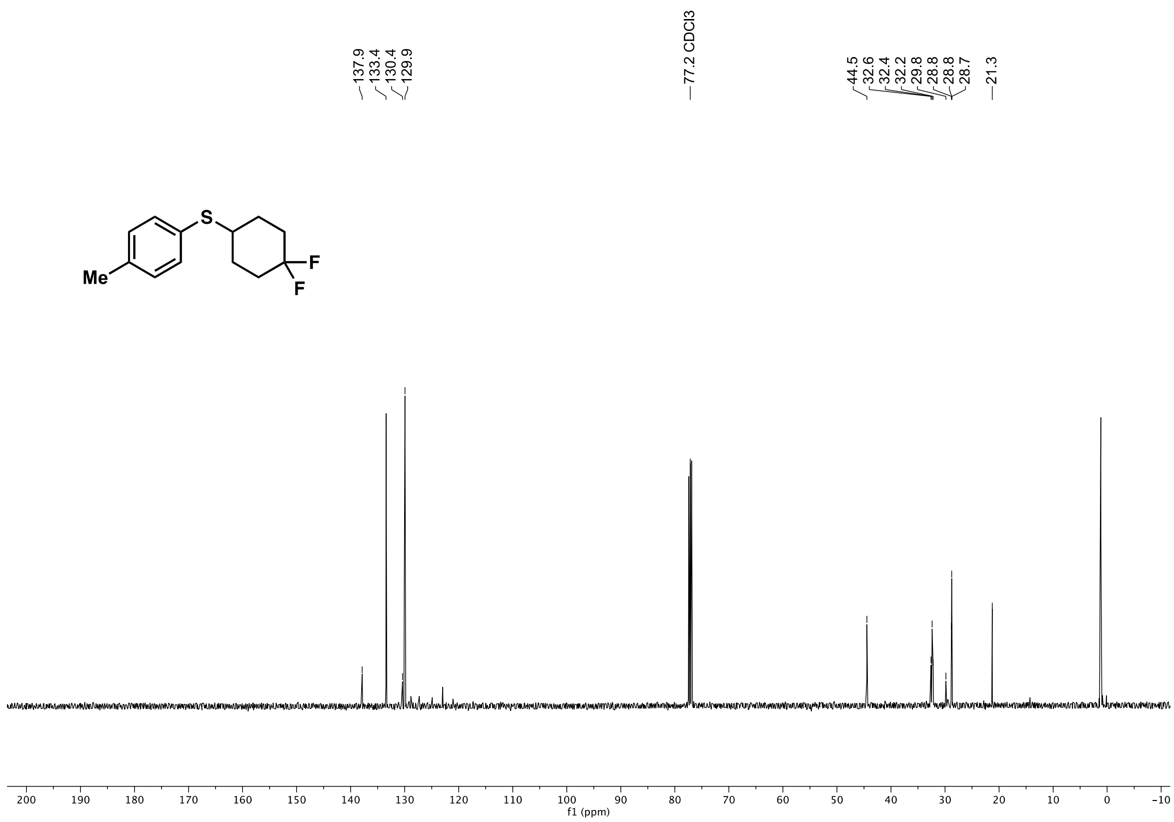

Supplementary Figure 122. ^{13}C NMR Spectra of product **43**

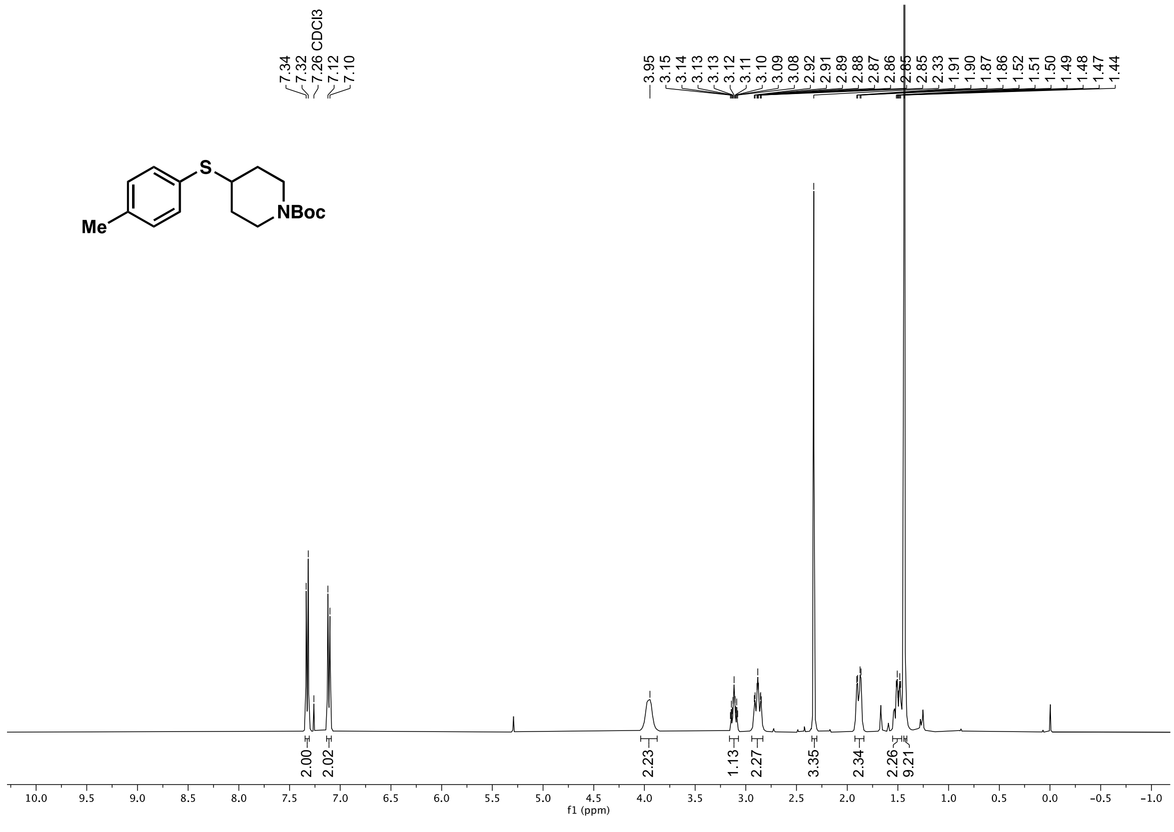

Supplementary Figure 123. ^{1}H NMR Spectra of product **44**

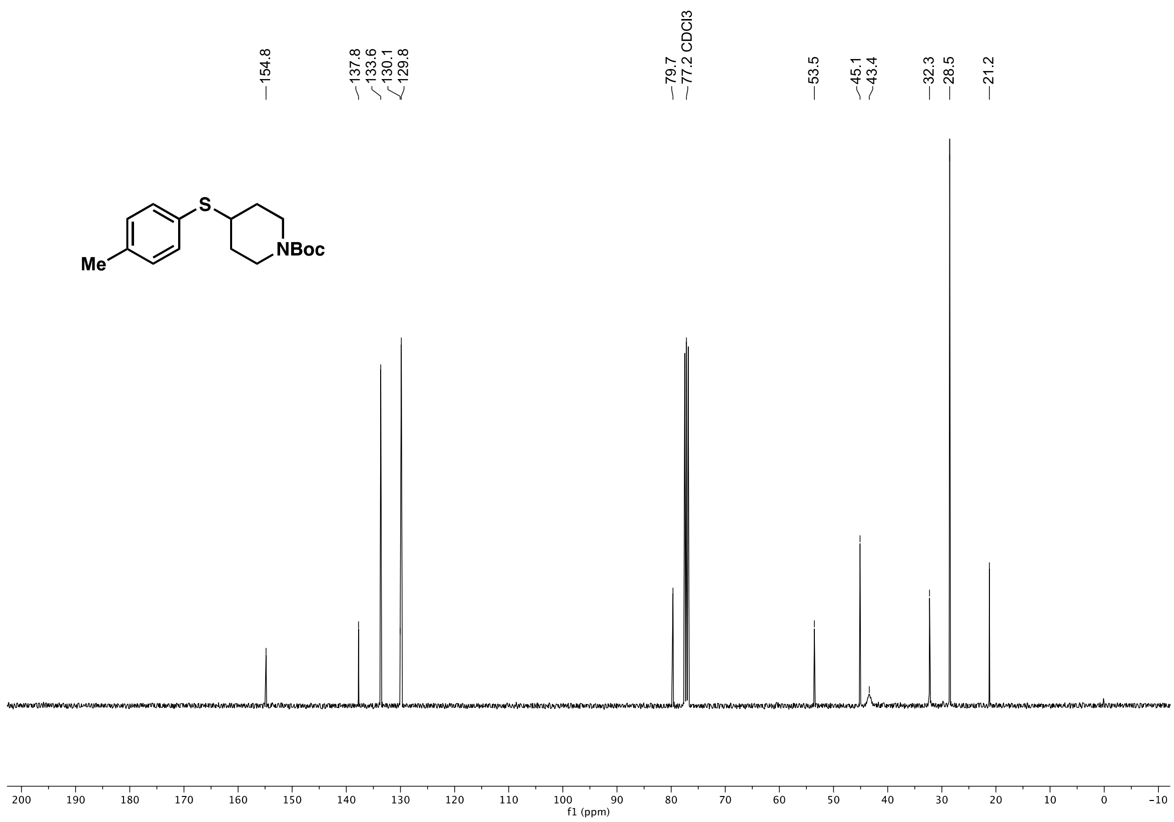

Supplementary Figure 124. ^{13}C NMR Spectra of product **44**

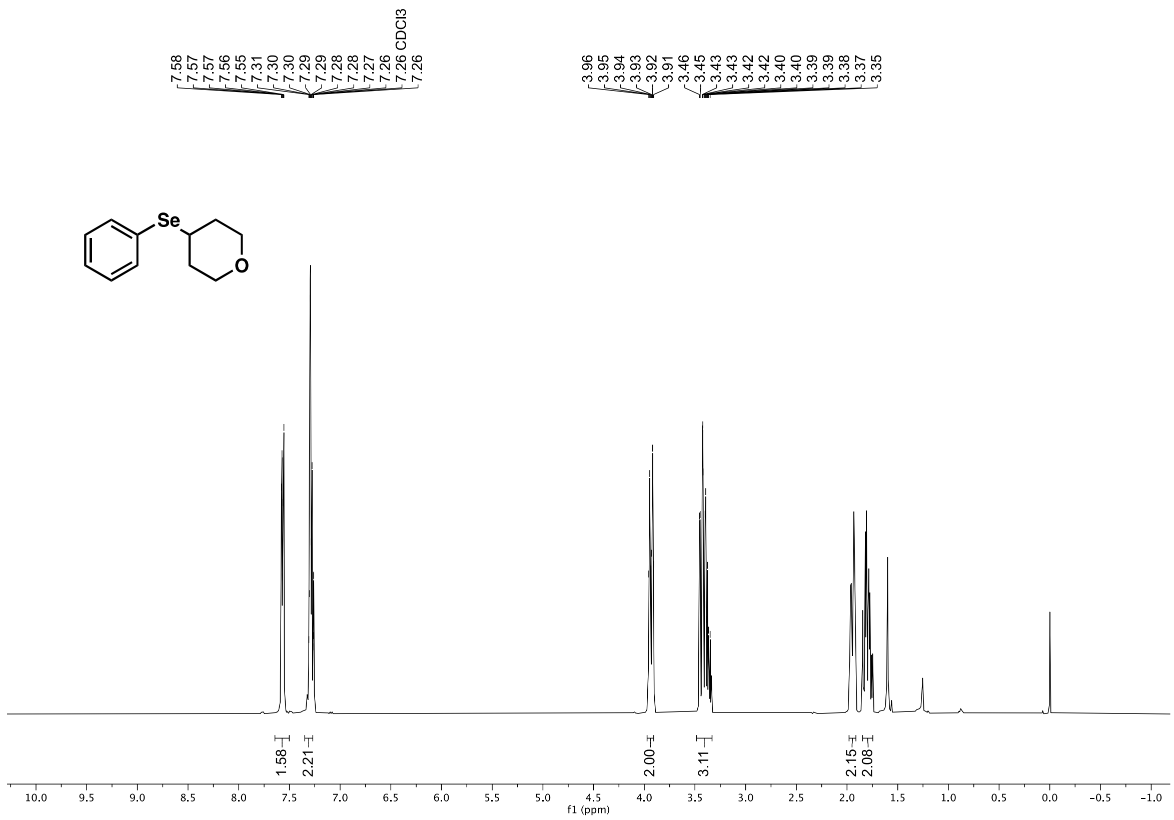

Supplementary Figure 125. ^{1}H NMR Spectra of product **45**

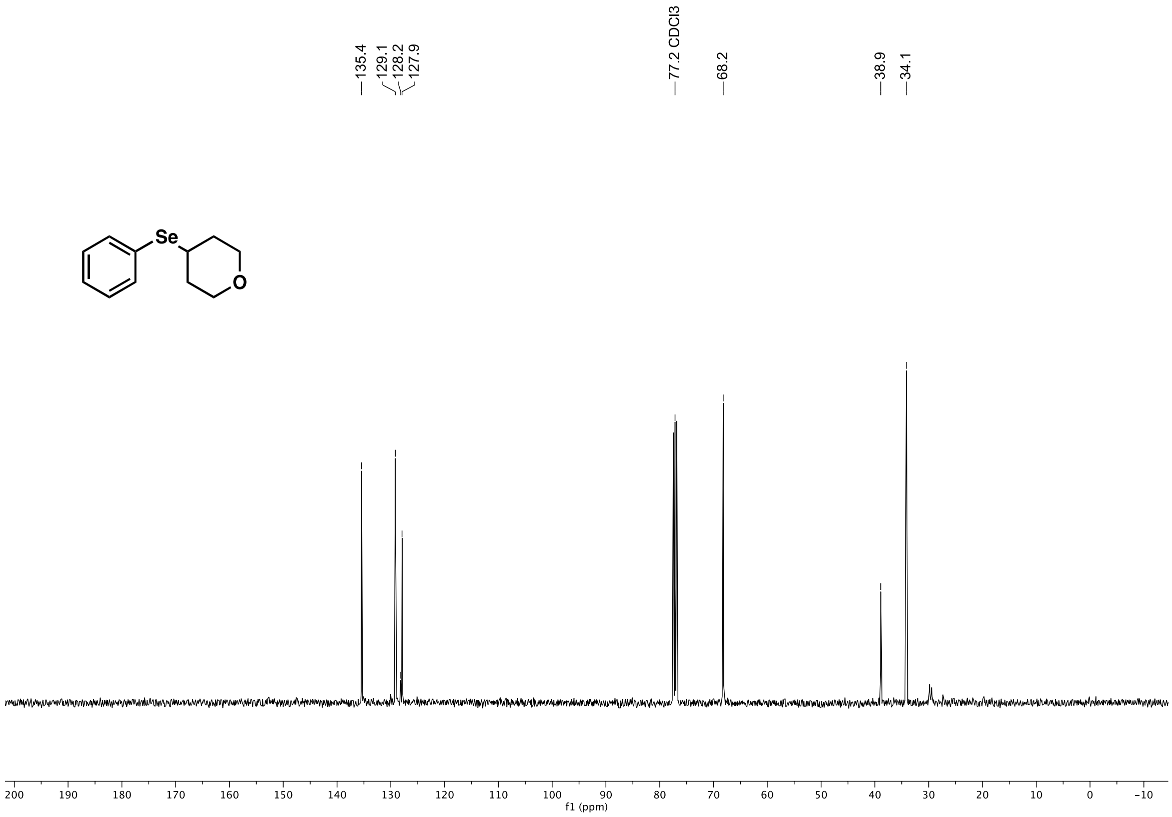

Supplementary Figure 126. ^{13}C NMR Spectra of product **45**

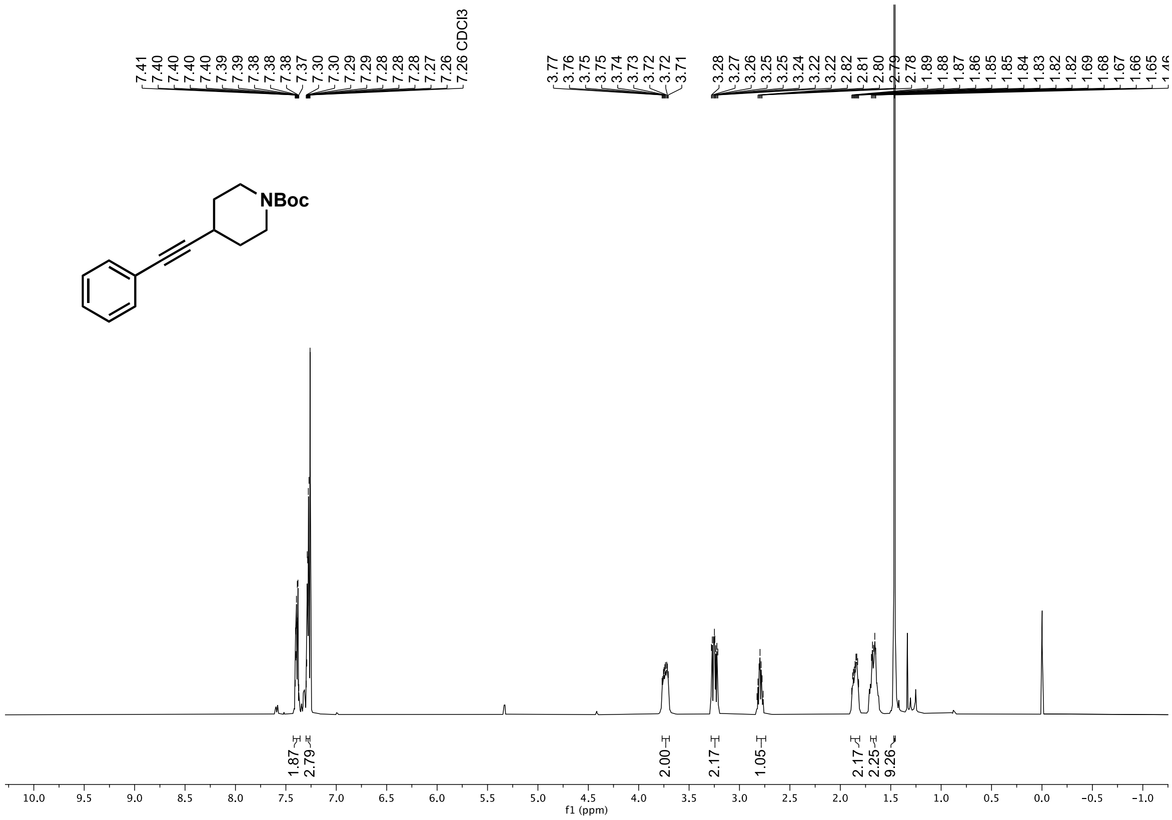

Supplementary Figure 127. ^{1}H NMR Spectra of product **46**

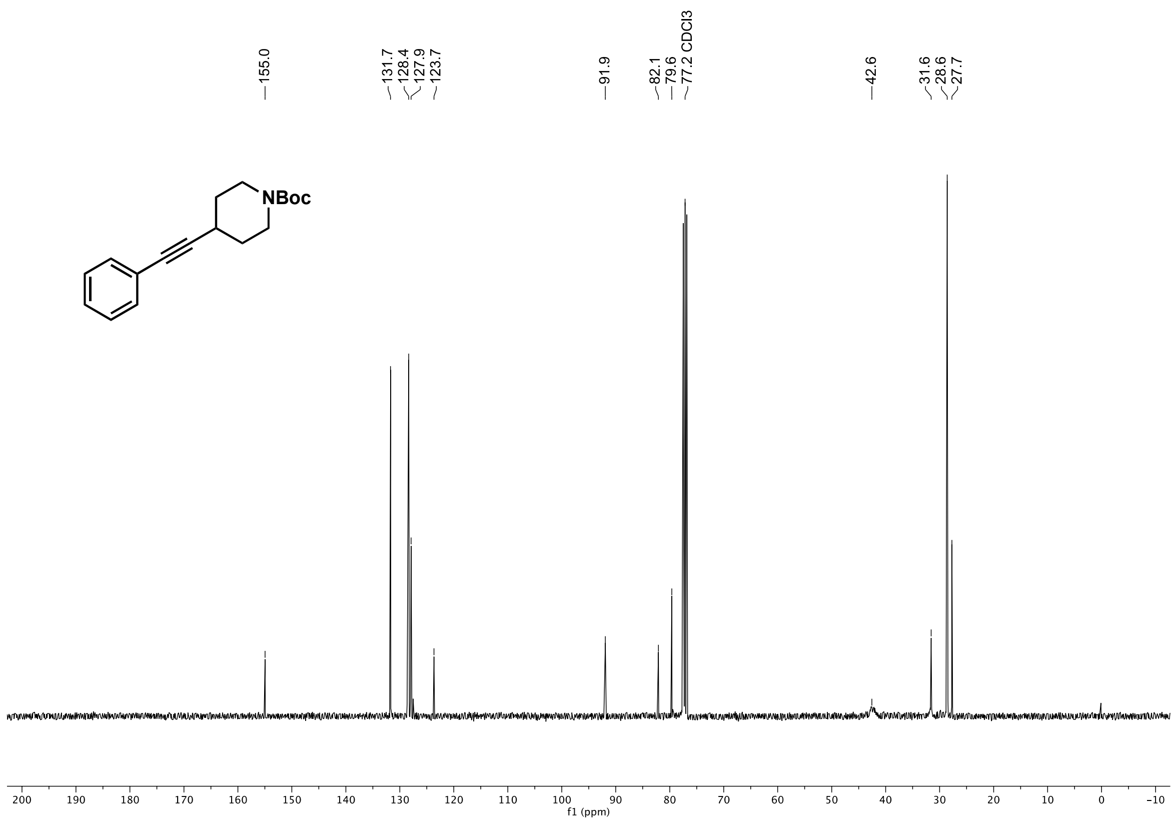

Supplementary Figure 128. ^{13}C NMR Spectra of product **46**

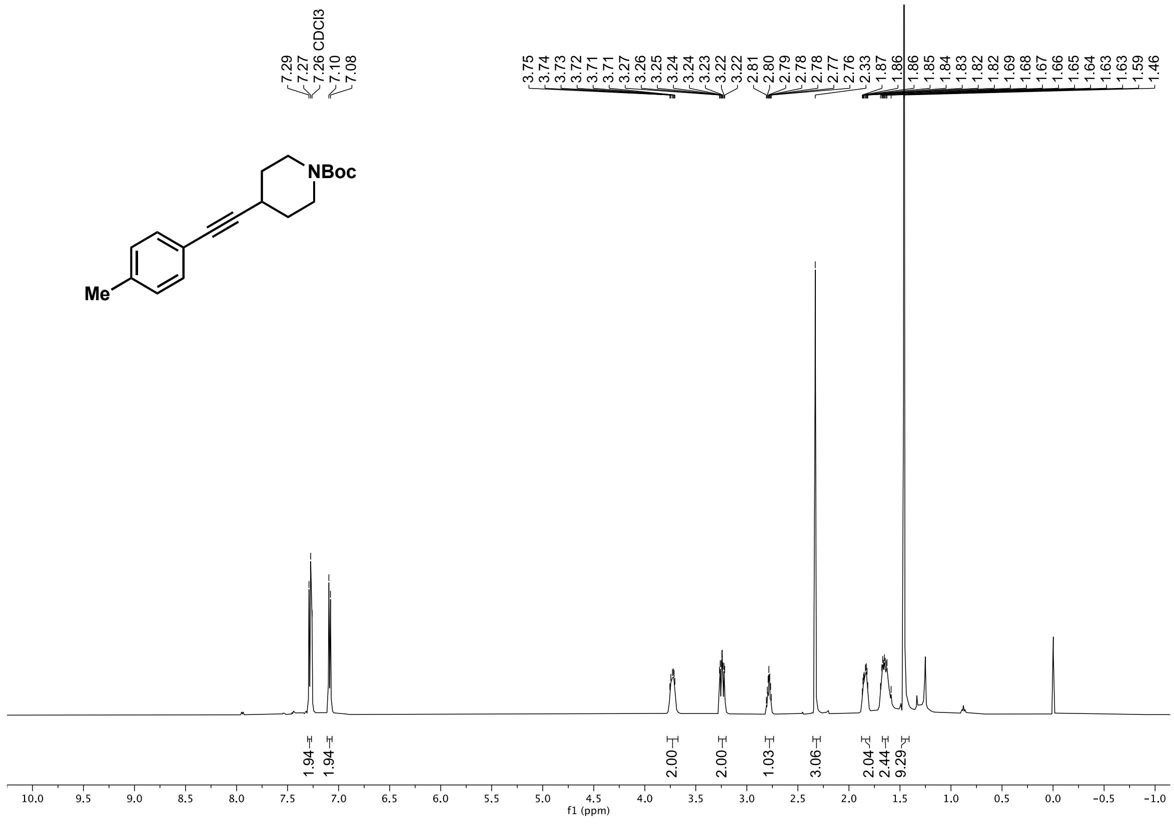

Supplementary Figure 129. ^{1}H NMR Spectra of product **47**

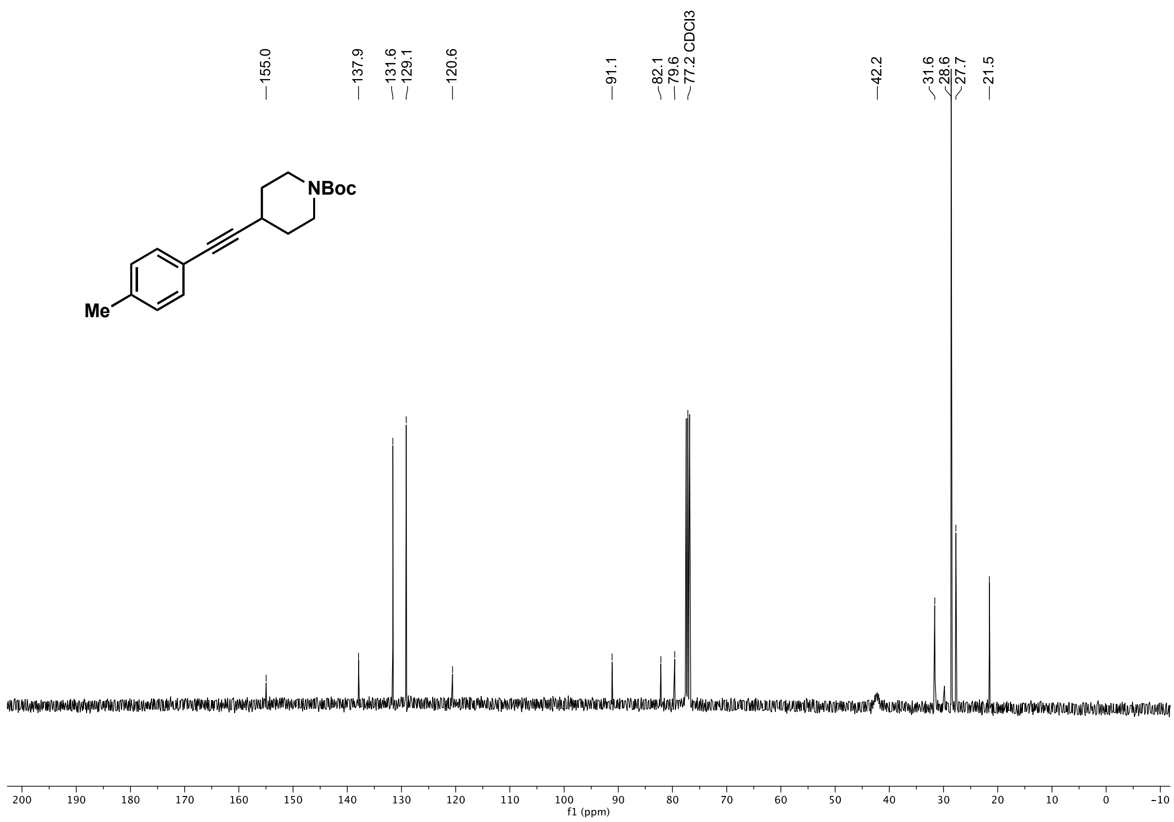

Supplementary Figure 130. ^{13}C NMR Spectra of product **47**

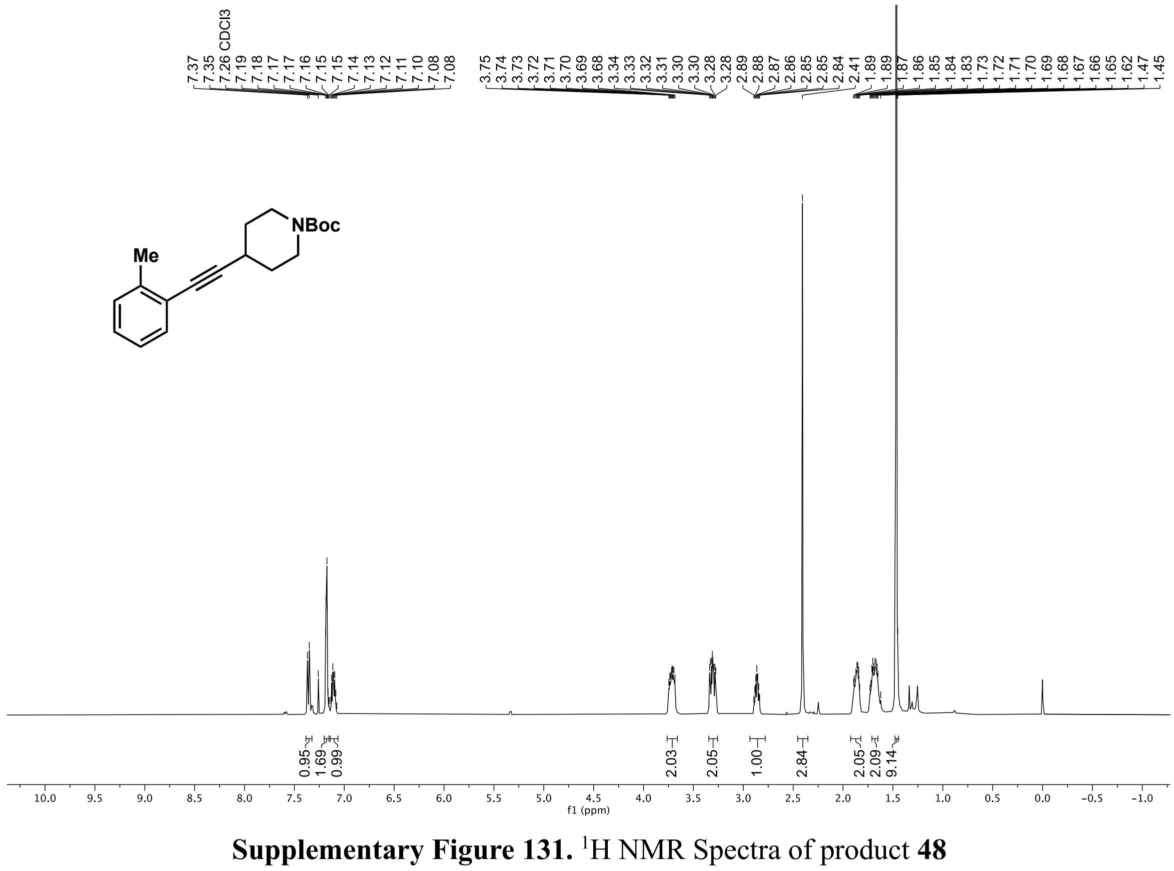

Supplementary Figure 131. ^{1}H NMR Spectra of product **48**

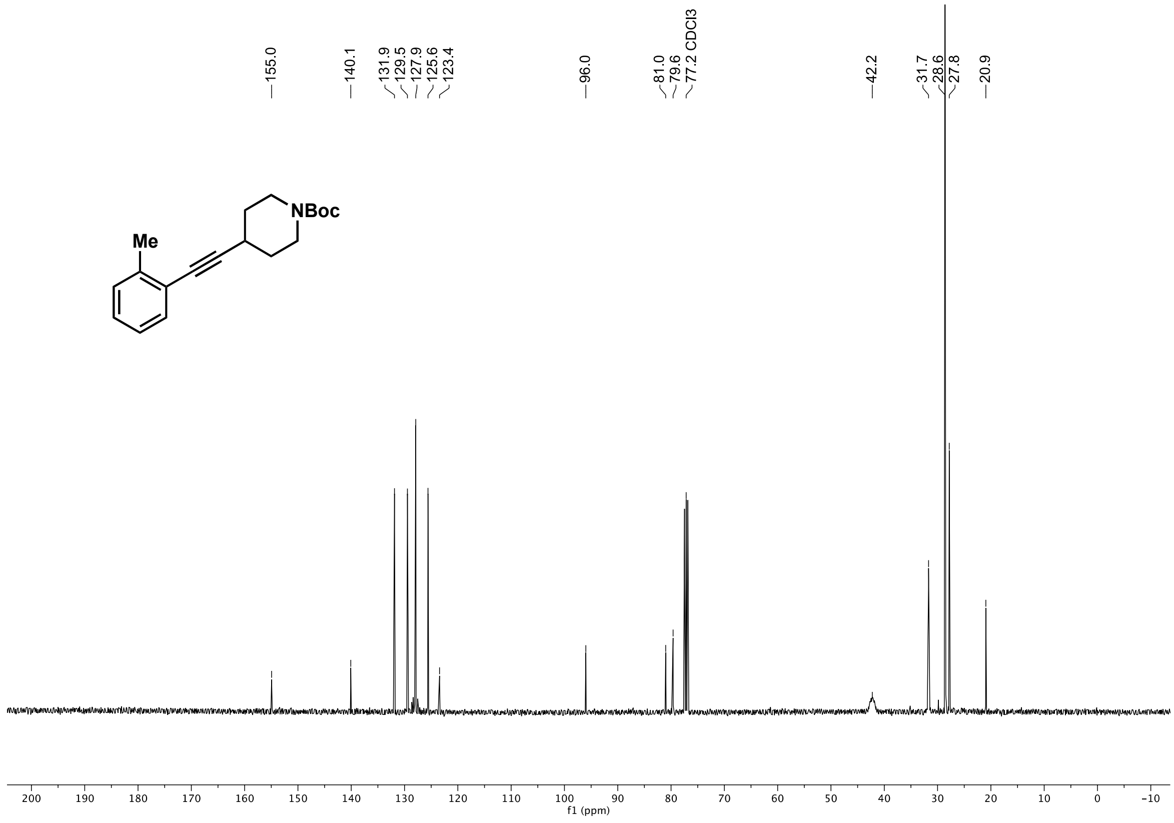

Supplementary Figure 132. ^{13}C NMR Spectra of product **48**

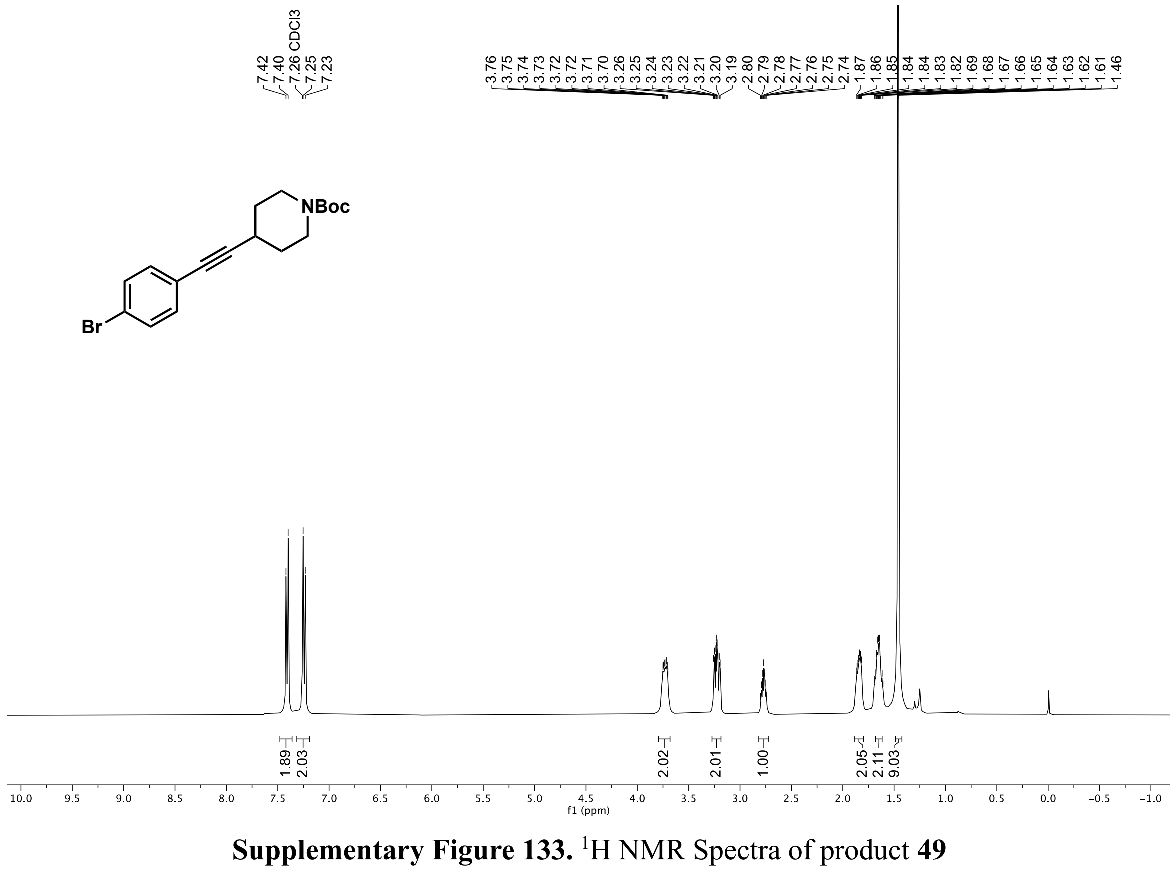

Supplementary Figure 133. ^{1}H NMR Spectra of product **49**

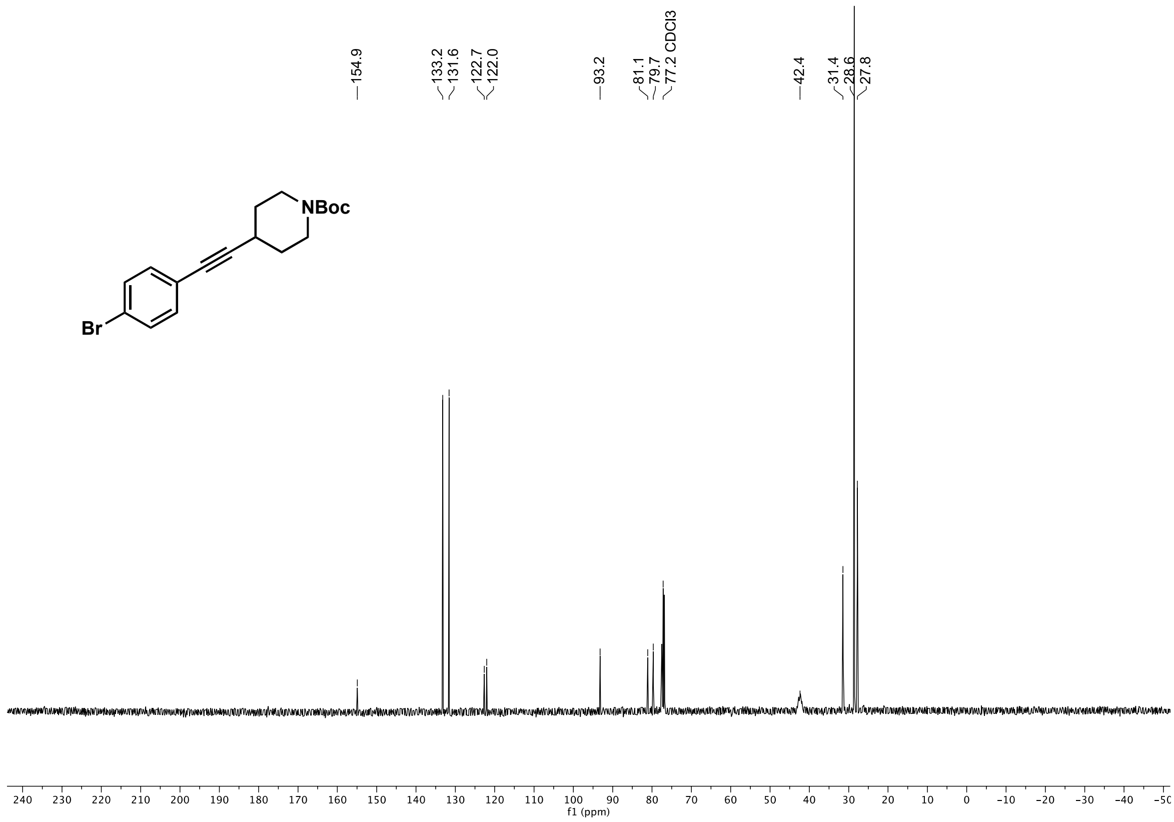

Supplementary Figure 134. ^{13}C NMR Spectra of product **49**

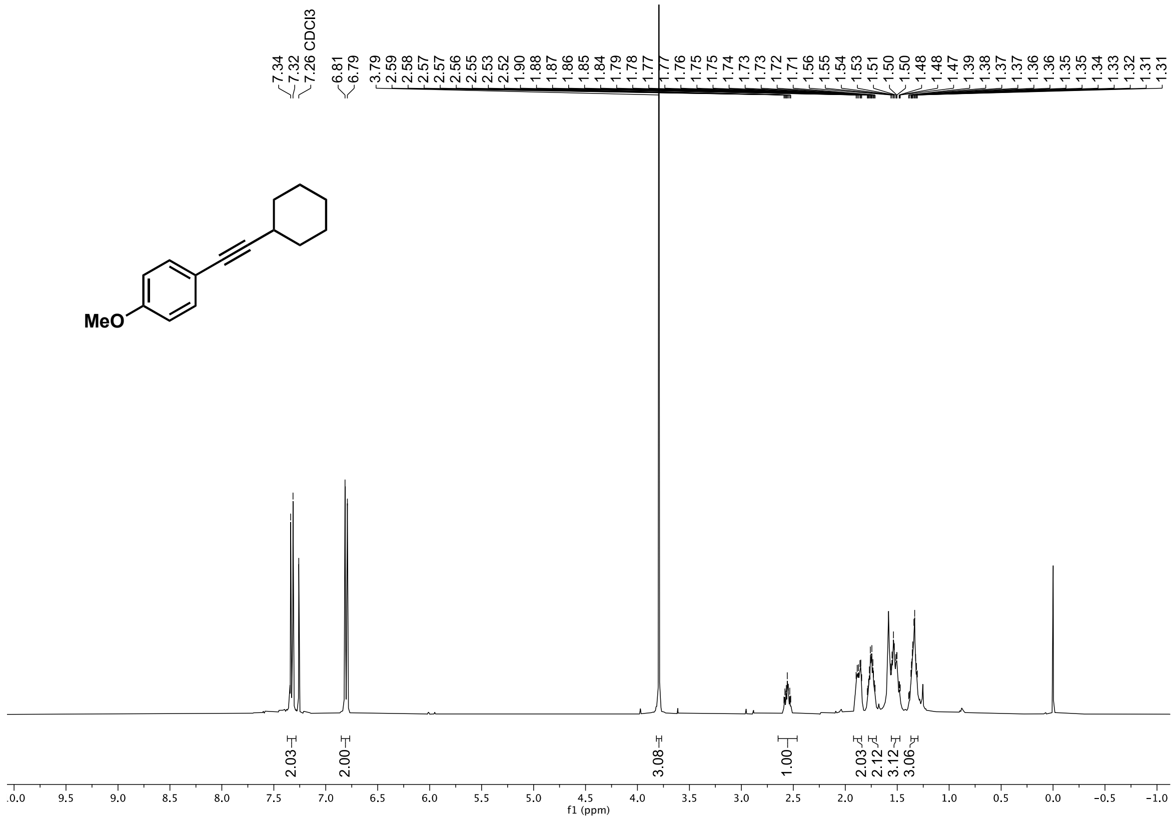

Supplementary Figure 135. ^{1}H NMR Spectra of product **50**

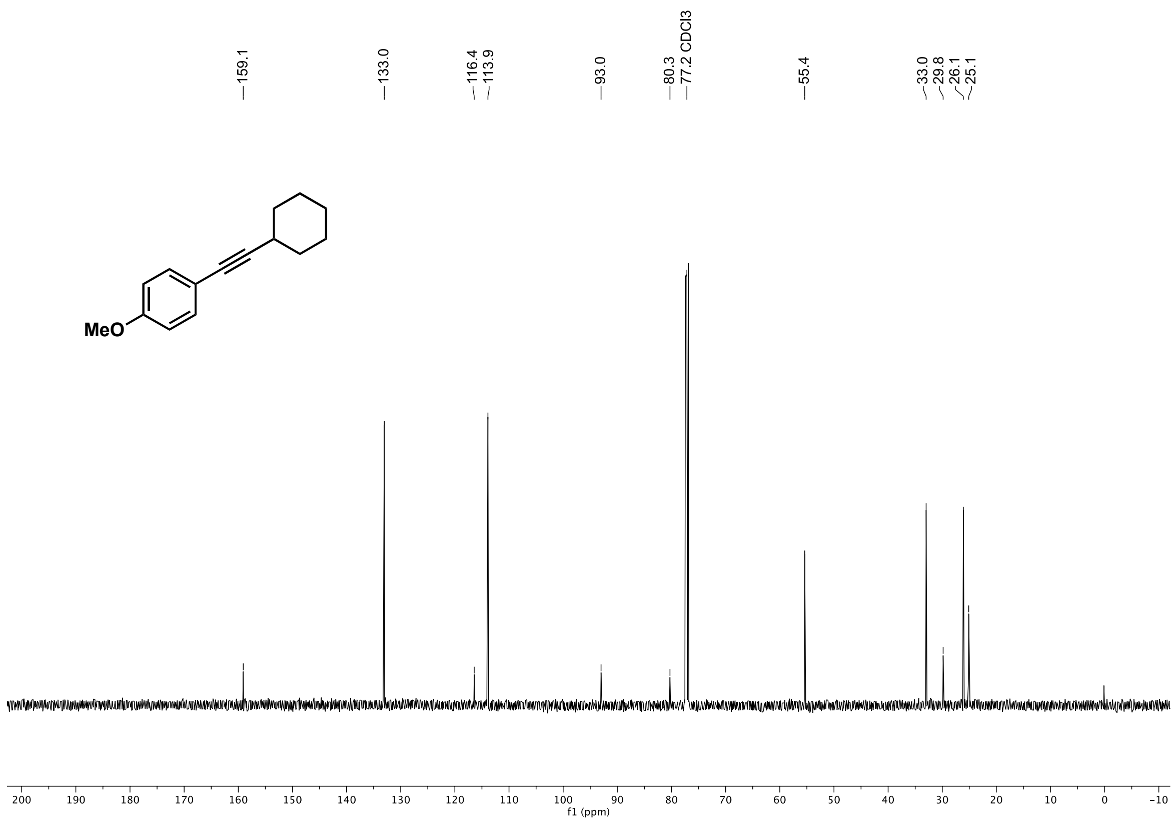

Supplementary Figure 136. ^{13}C NMR Spectra of product **50**

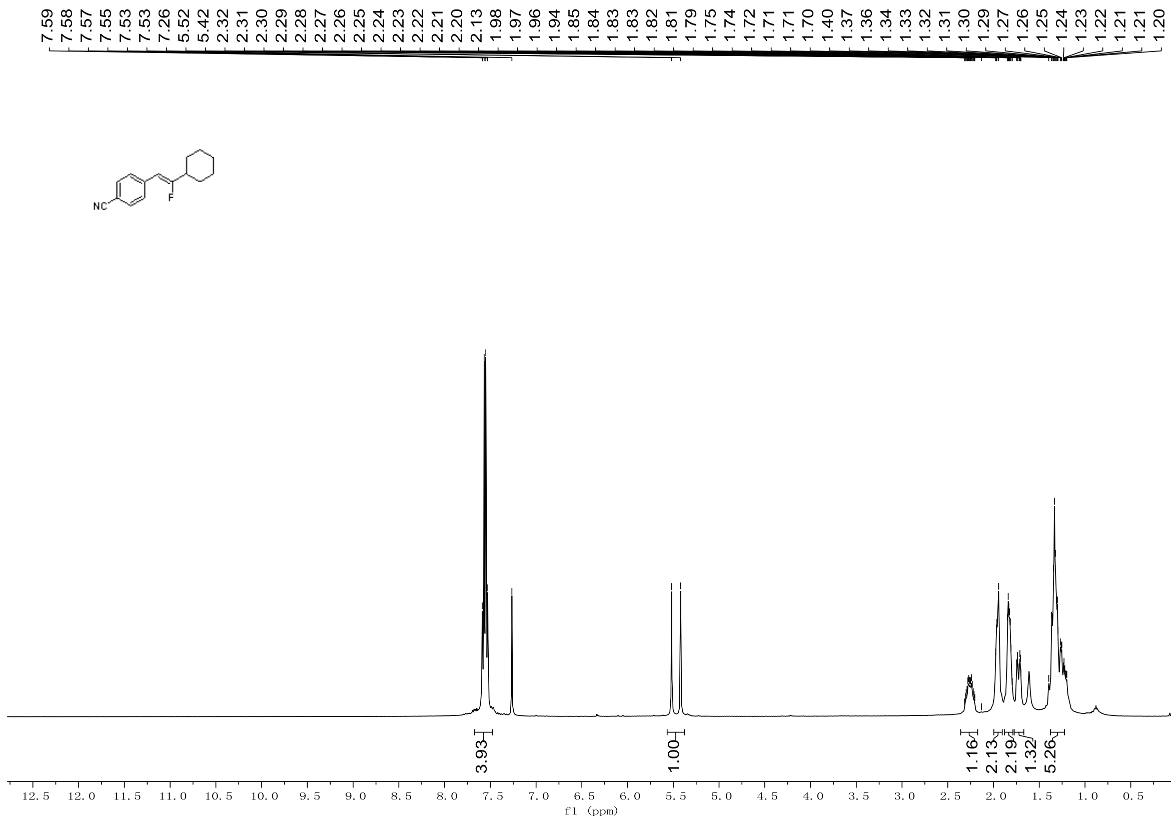

Supplementary Figure 137. ^{1}H NMR Spectra of product **52**

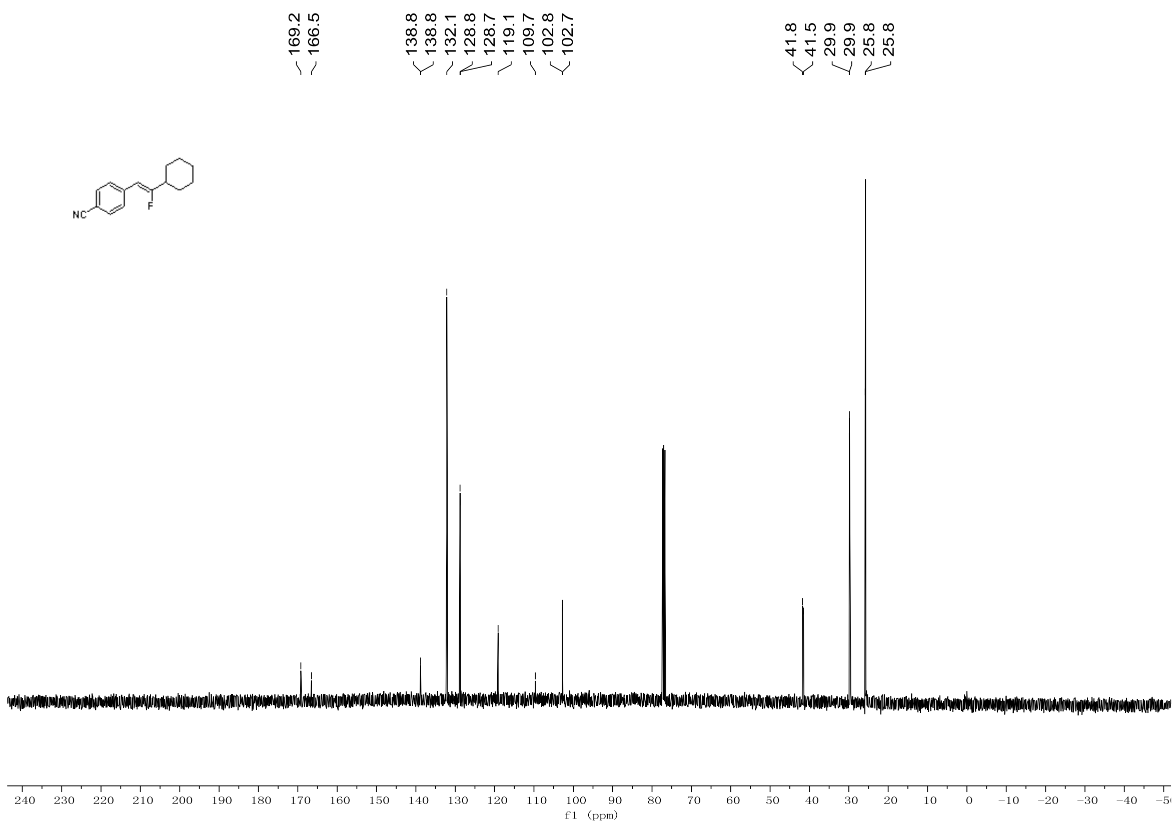

Supplementary Figure 138. ^{13}C NMR Spectra of product **52**

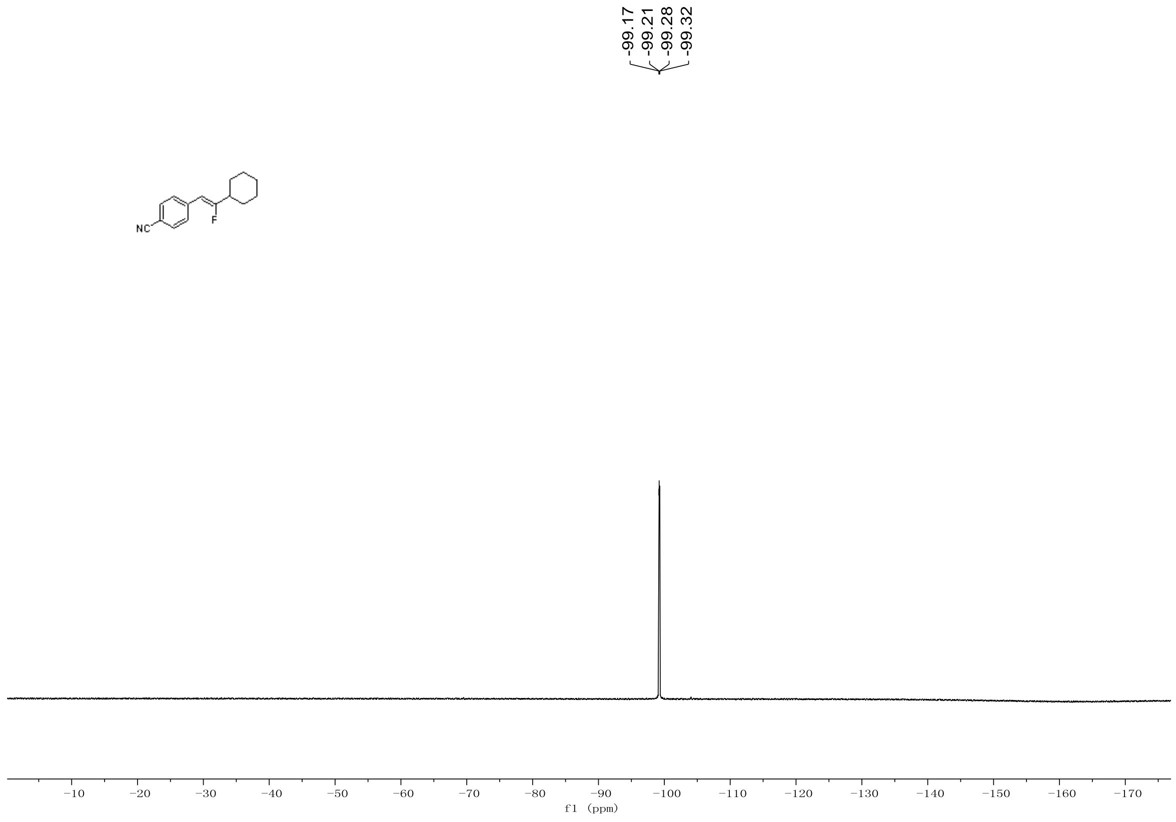

Supplementary Figure 139. ^{19}F NMR Spectra of product **52**

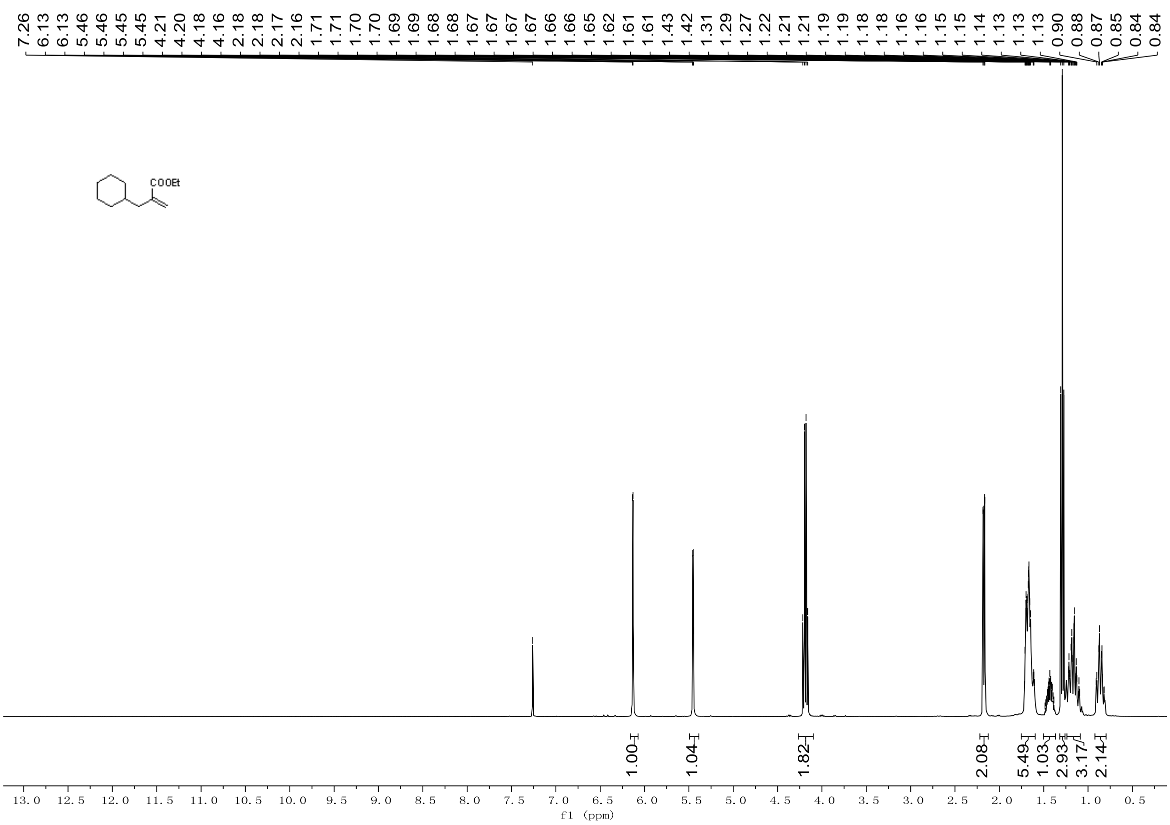

Supplementary Figure 140. ^{1}H NMR Spectra of product **54**

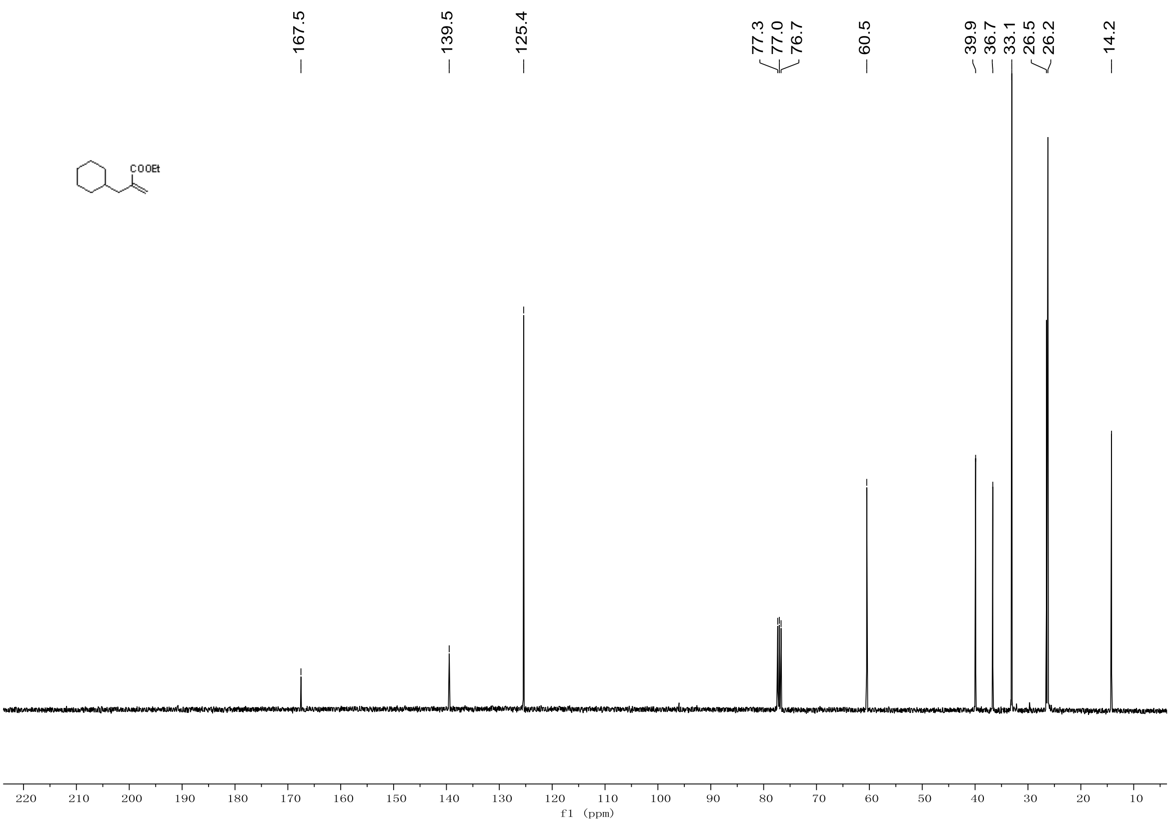

Supplementary Figure 141. ^{13}C NMR Spectra of product **54**

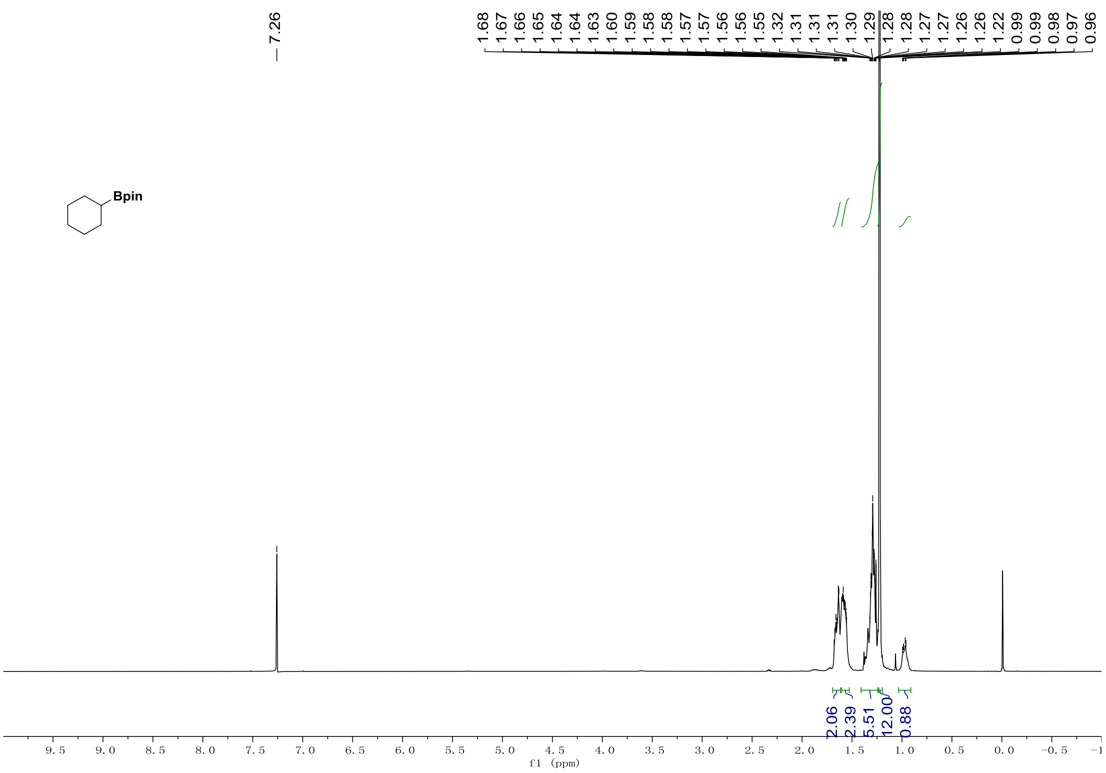

Supplementary Figure 142. ^{1}H NMR Spectra of product **55**

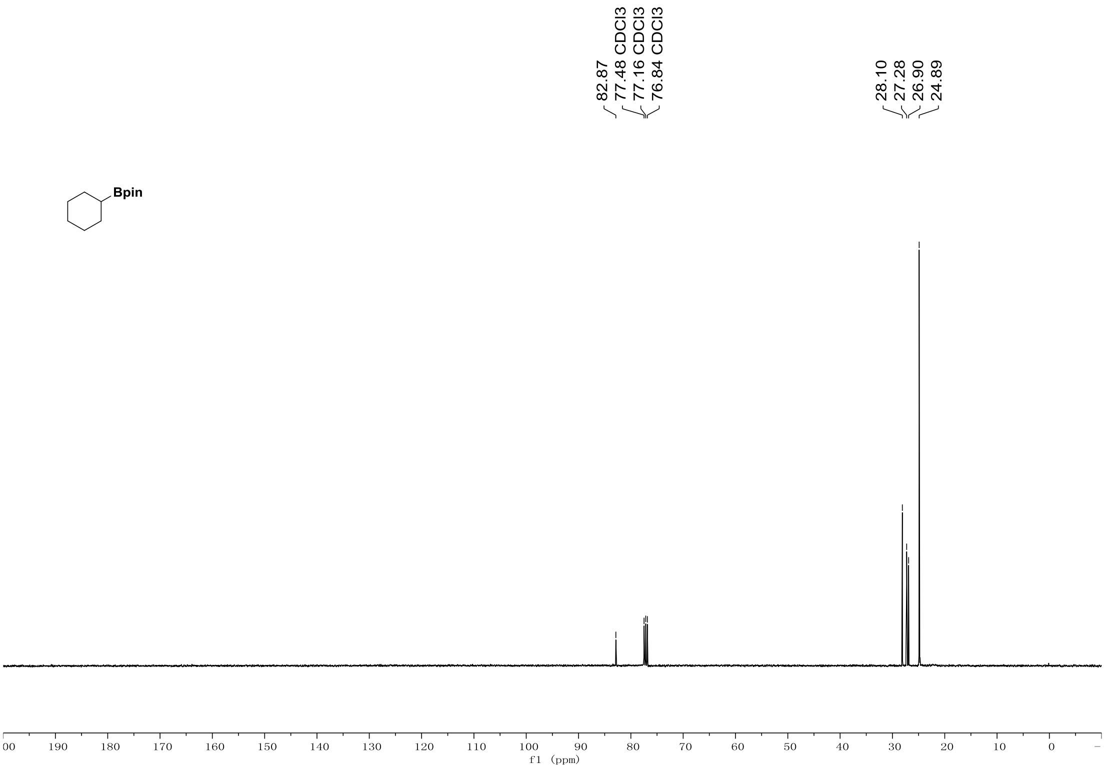

Supplementary Figure 143. ^{13}C NMR Spectra of product **55**

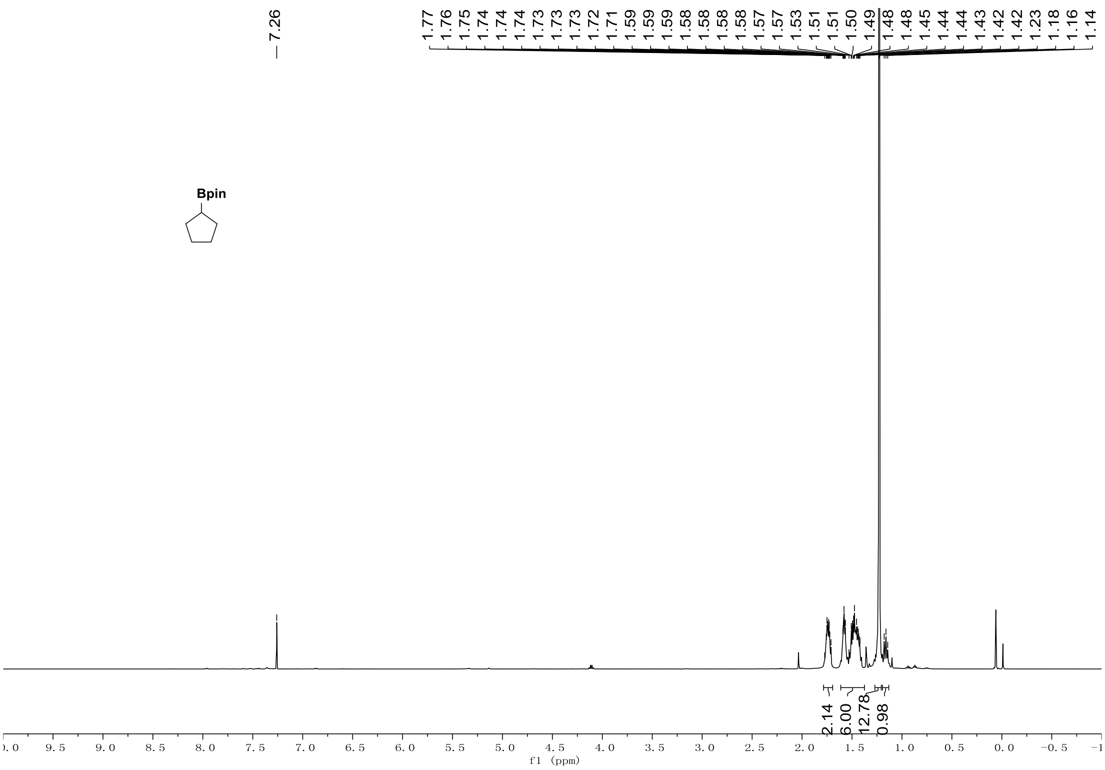

Supplementary Figure 144. ^{1}H NMR Spectra of product **56**

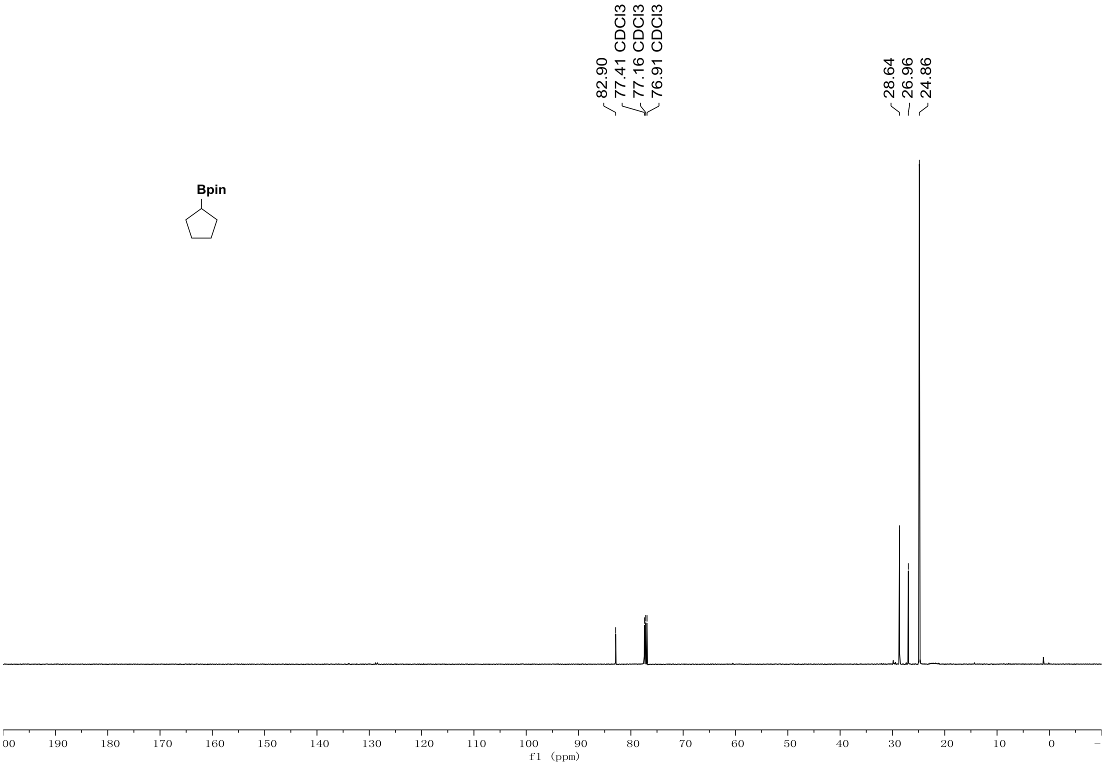

Supplementary Figure 145. ^{13}C NMR Spectra of product **56**

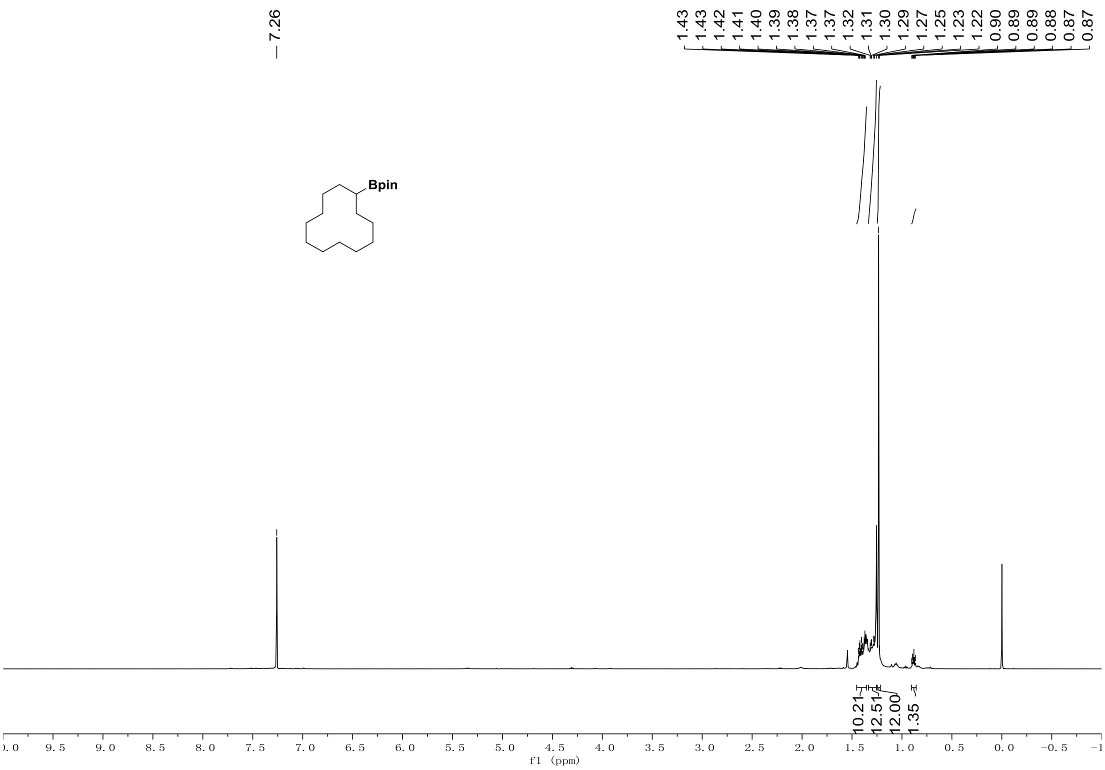

Supplementary Figure 146. ^{1}H NMR Spectra of product **57**

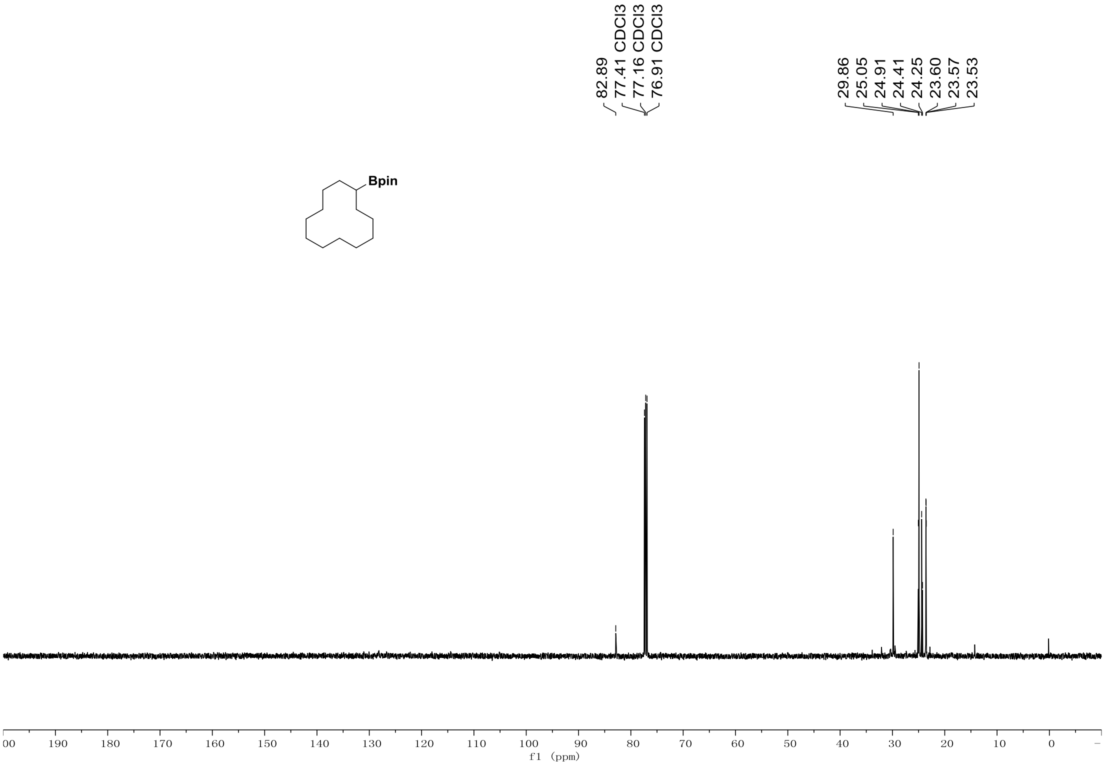

Supplementary Figure 147. ^{13}C NMR Spectra of product **57**

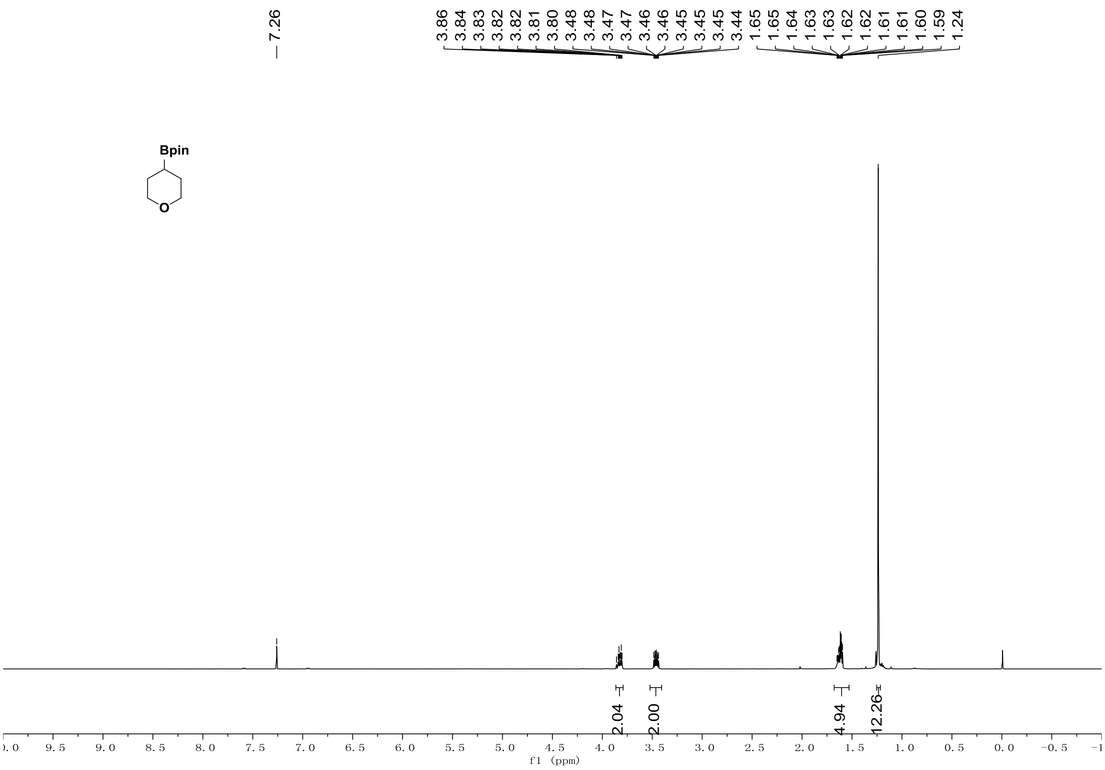

Supplementary Figure 148. ^{1}H NMR Spectra of product **58**

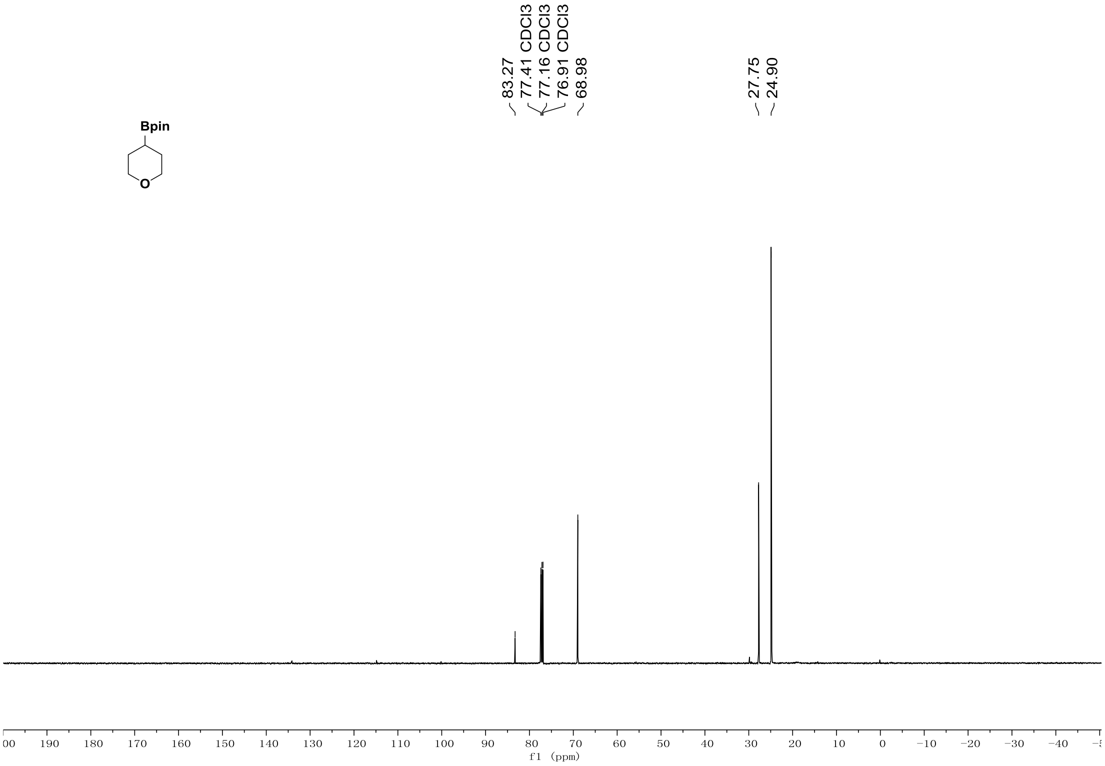

Supplementary Figure 149. ^{13}C NMR Spectra of product **58**

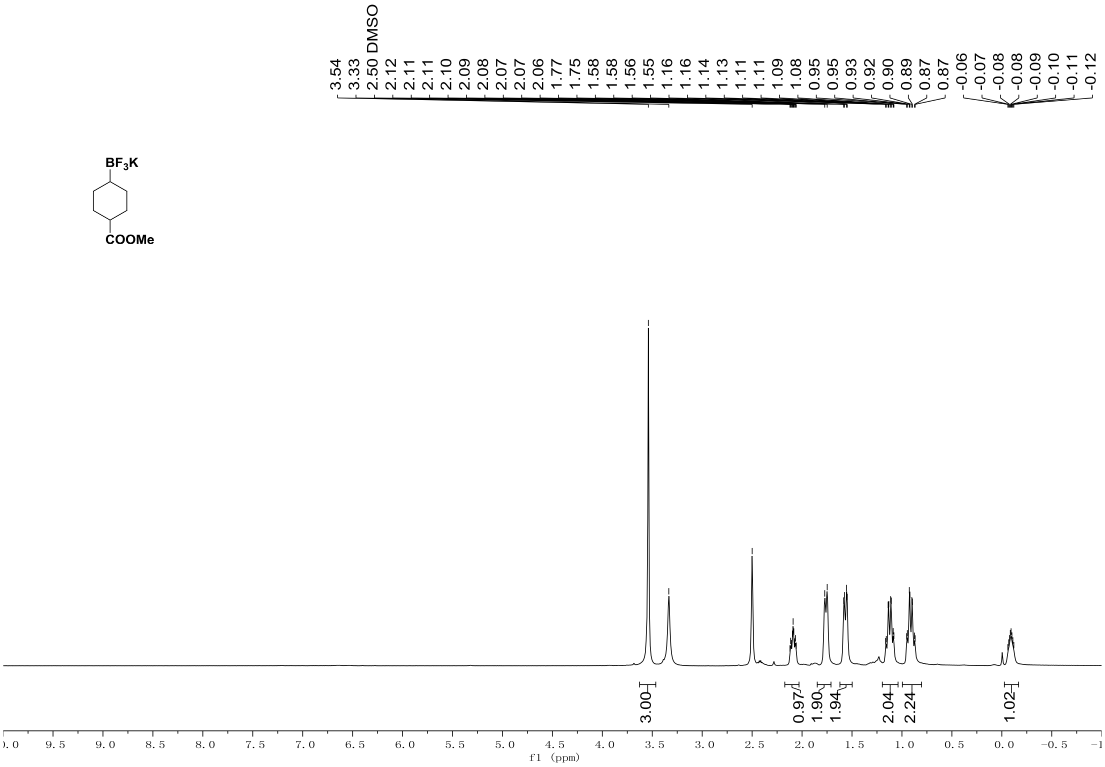

Supplementary Figure 150. ^{1}H NMR Spectra of product **59**

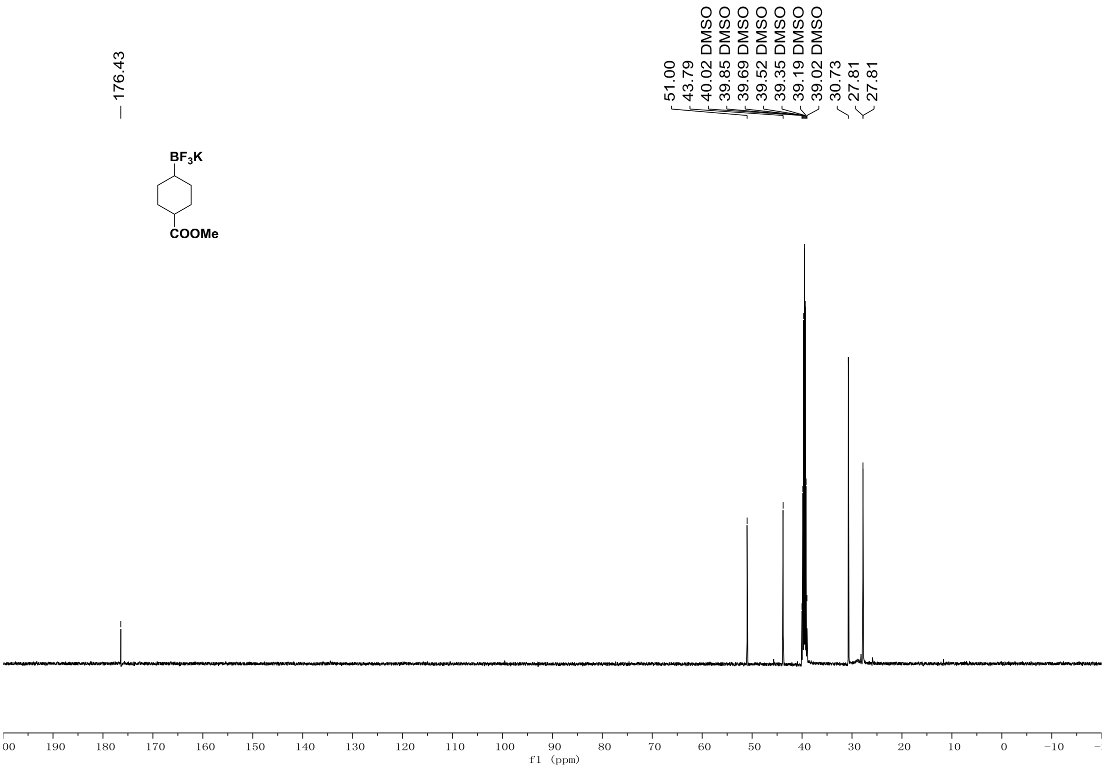

Supplementary Figure 150. ^{13}C NMR Spectra of product **59**

Supplementary Note

1. Chen, Y., et al. The coupling of alkylboronic acids with α-(trifluoromethyl)styrenes by Lewis base/photoredox dual catalysis. *Tetrahedron Lett.* **61**, 152425 (2020).
2. Lu, X. et al. Nickel-catalyzed allylic defluorinative alkylation of trifluoromethyl alkenes with reductive decarboxylation of redox-active esters. *Chem. Sci.* **10**, 809–814 (2019).
3. Kumar, G. et al. Carbon-sulphur cross coupling reactions catalyzed by nickel-based coordination polymers based on metalloligands. *Dalton Trans.* **46**, 15023–15031 (2017).
4. Guo, S., et al. Palladium-catalyzed thiolation of alkanes and ethers with arylsulfonyl hydrazides. *Chem. Commun.* **50**, 8578–8581 (2014).
5. Smaligo, A. J., et al. Dealkenylative Thiylation of C(sp^3)–C(sp^2) Bonds. *Org. Lett.* **21**, 8592–8597 (2019).
6. Tota, A. et al. Synthesis of NH-sulfoximines from sulfides by chemoselective one-pot N- and O-transfers. 5 *Chem. Commun.* **3**, 348–351 (2016).
7. Liu, X.-G. et al. Decarboxylative Negishi Coupling of Redox-Active Aliphatic Esters by Cobalt Catalysis. *Angew. Chem. Int. Ed.* **57**, 13096–13100 (2018).
8. Mao, Y. et al. Copper-catalysed photoinduced decarboxylative alkynylation: a combined experimental and computational study. *Chem. Sci.* **11**, 4939–4947 (2020).
9. Tang, S. et al. Multi-Metal-Catalyzed Oxidative Radical Alkynylation with Terminal Alkynes: A New Strategy for C(sp^3)–C(sp) Bond Formation. *J. Am. Chem. Soc.* **140**, 6006–6013 (2018).
10. Zhou, M. D. et al. Synthesis of gem-Difluoroalkenes via Zn-Mediated Decarboxylative/ Defluorinative Cross-Coupling. *Org.Lett.* **22**, 9342-9345. (2020).
11. Mahadev L.S., et al., Efficient synthesis of alkylboronic esters via magnetically recoverable copper nanoparticlecatalyzed borylation of alkyl chlorides and bromides. *Green Chem.*,**22**, 2799–2803(2020)
12. Bose, S. K. et al. *Angew. Chem. Int. Ed.* **53**, 1799–1803(2014).
13. Alexander F. et al. Photoinduced decarboxylative borylation of carboxylic acids, Science **357**, 283–286 (2017).
14. Qiang Z. et al., Decarboxylative Borylation of Stabilized and Activated CarbonRadicals, Angew. Chem. Int. Ed. **59**, 21875 –21879 (2020).
